# Supplementary material for: Olfactory Gene Families in Scopula subpunctaria and Candidates for Type-II Sex Pheromone Detection
Source: Int J Mol Sci. 2022 Dec 12;23(24):15775. doi: 10.3390/ijms232415775 (PMC9779464; doi:10.3390/ijms232415775)
Supplement: Supplementary file 1 [file ijms-23-15775-s001.zip › ijms-2042130-supplementary.pdf]

Supplementary Table S1. Sequencing yield statistics

Type	Transcript
max length (bp)	73,713
min length (bp)	201
mean length (bp)	609
N50 length (bp)	1,016

Supplementary Table S2. BLASTX results putative IRs, OBPs, SNMPs, ORs, and CSPs in *S. subpunctaria*.

Gene		ORF	FPKM FA	FPKM MA	Best Blastp Match			
**Name**	**ID**				**Species**	**E-value**	**Identity**	**Acc.number**
**OR 1**	c70447_g1	NO	2.80	2.01	odorant receptor 59 [Peridroma saucia]	1.00 E-47	54.48%	QHB15293.1
**OR 2**	c76544_g1	NO	2.60	1.2	Odorant receptor 50 [Operophtera brumata]	1.00 E-66	59.78%	KOB74670.1
**OR 3**	c132232_g1	NO	0.00	0	odorant receptor 4-like isoform X3 [Ostrinia furnacalis]	5E-67	59.49%	XP_028167120.1
**OR 4**	c85171_g1	YES	3.31	1.69	olfactory receptor 20 [Bombyx mori]	3.00 E-79	48.74%	NP_001166605.1
**OR 5**	c42080_g1	YES	0.00	7.83	Odorant Receptor 13 [Dendrolimus punctatus]	3.00 E-07	60.98%	ARO70225.1
**OR 6**	c83273_g2	YES	1.48	1.65	odorant receptor 3 [Ectropis obliqua]	9.00 E-136	82.67%	ALS03874.1
**OR 7**	c91152_g1	YES	3.74	3.25	odorant receptor 67a-like [Trichoplusia ni]	9.00 E-163	55.97%	XP_026732930.1
**OR 8**	c78758_g1	YES	3.81	2.3	Odorant Receptor 30-2 [Dendrolimus punctatus]	4.00 E-79	75.97%	ARO70510.1

**OR 9**	c96955_g2	YES	8.80	149.47	putative odorant receptor OR63 [Cydia fagiglandana]	2.00E-62	58.43%	AST36334.1
**OR 10**	c88936_g1	YES	5.81	5.16	putative odorant receptor OR45 [Athetis lepigone]	5.00E-09	71.43%	AOE48050.1
**OR 11**	c99406_g1	YES	4.93	2.51	odorant receptor 7 [Athetis dissimilis]	5.00E-114	69.67%	ALM26195.1
**OR 12**	c85181_g2	YES	1.81	2.63	putative odorant receptor OR27 [Cydia nigricana]	7.00E-123	61.54%	AST36392.1
**OR 13**	c72434_g1	NO	1.57	1.2	odorant receptor 51 [Manduca sexta]	3.00E-48	43.81%	AFL70814.1
**OR 14**	c93473_g3	YES	4.08	3.54	olfactory receptor 28 [Heortia vitessoides]	1.00E-156	54.46%	AZB49442.1
**OR 15**	c144452_g1	NO	0.00	0	odorant receptor [Semiothisa cinerearia]	2E-119	63.50%	QRF70969.1
**OR 16**	c92936_g1	YES	3.64	2.99	odorant receptor 33 [Conogethes pinicolalis]	0	75.87%	QEE82751.1
**OR 17**	c129165_g1	YES	6.24	2.35	odorant receptor [Eogystia hippophaecolus]	9.00E-09	45.95%	AOG12923.1
**OR 18**	c92338_g1	YES	7.16	3.54	odorant receptor, partial [Semiothisa cinerearia]	0	81.27%	QRF70985.1
**OR 19**	c71501_g1	YES	1.23	3.81	odorant receptor [Dendrolimus kikuchii]	1.00E-55	57.43%	AII01083.1
**OR 20**	c49697_g1	YES	0.00	0	putative odorant receptor [Peridroma saucia]	4.00E-12	41.82%	AVF19668.1
**OR 21**	c80483_g1	NO	2.25	0.31	odorant receptor 67c-like [Spodoptera litura]	2.00E-104	61.97%	XP_022824679.1
**OR 22**	c51622_g1	YES	2.20	1.51	odorant receptor [Dendrolimus kikuchii]	2.00E-68	64.17%	AII01083.1

**OR 23**	c86666_g1	YES	2.74	2.97	odorant receptor 2 [Ectropis obliqua]	2.00E-108	70.40%	ALS03873.1
**OR 24**	c97867_g1	YES	9.40	4.68	odorant receptor 4 [Bombyx mori]	9.00E-99	58.37%	XP_021205480.1
**OR 25**	c90510_g1	YES	3.41	2.02	odorant receptor [Dendrolimus kikuchii]	4.00E-124	64.23%	AII01084.1
**OR 26**	c90959_g1	NO	5.16	3.49	putative odorant receptor OR25 [Cydia fagiglandana]	1.00E-111	52.96%	AST36312.1
**OR 27**	c98196_g1	YES	6.85	3.91	odorant receptor 46a-like [Trichoplusia ni]	9.00E-91	55.52%	XP_026735047.1
**OR 28**	c97995_g2	YES	3.47	3.31	Odorant Receptor 31-2 [Dendrolimus punctatus]	2.00E-162	62.29%	ARO70511.1
**OR 29**	c20967_g1	YES	0.00	1.87	Odorant receptor 50 [Operophtera brumata]	8.00E-41	51.08%	KOB52347.1
**OR 30**	c101189_g1	YES	8.15	6.7	odorant receptor [Helicoverpa armigera]	4.00E-168	55.42%	AIG51891.1
**OR 31**	c85150_g1	NO	5.53	3.27	olfactory receptor OR22 [Planotortrix octo]	1.00E-13	32.50%	AJF23793.1
**OR 32**	c84763_g1	YES	3.66	2.91	odorant receptor 4-like [Bombyx mandarina]	3.00E-145	55.37%	XP_028028939.1
**OR 33**	c85913_g1	YES	2.56	1.37	Odorant receptor [Operophtera brumata]	3.00E-165	62.37%	KOB67926.1
**OR 34**	c90383_g2	YES	1.88	1.52	odorant receptor [Helicoverpa armigera]	0	74.77%	AIG51888.1
**OR 35**	c93606_g1	YES	4.44	4.82	Odorant Receptor 14-2 [Dendrolimus punctatus]	1.00E-153	58.37%	ARO70503.1
**OR 36**	c93823_g1	YES	2.62	2.19	Odorant Receptor 14-2 [Dendrolimus punctatus]	4.00E-124	52.33%	ARO70503.1

**OR 37**	c94915_g1	YES	2.71	2.2	odorant receptor 49a-like [Helicoverpa armigera]	0	75.63%	XP_021182925.1
**OR 38**	c96783_g1	YES	4.28	2.48	odorant receptor [Helicoverpa armigera]	2.00E-113	44.65%	AIG51896.1
**OR 39**	c96810_g1	YES	4.01	2.17	odorant receptor 94b-like [Helicoverpa armigera]	6.00E-140	60.91%	XP_021199795.1
**OR 40**	c96976_g2	YES	33.09	5	odorant receptor Or1-like [Manduca sexta]	9.00E-126	54.11%	XP_030030434.1
**OR 41**	c97235_g1	YES	5.37	4.78	odorant receptor [Helicoverpa armigera]	4.00E-69	41.30%	AIG51896.1
**OR 42**	c97403_g1	YES	15.08	3.49	odorant receptor [Helicoverpa armigera]	0	67.08%	AIG51875.1
**OR 43**	c97841_g1	YES	39.30	3.13	Olfactory receptor 34 [Manduca sexta]	3.00E-137	53.45%	CUQ99414.1
**OR 44**	c98272_g1	YES	0.04	102.28	TPA: Olfactory receptor 62 [Manduca sexta]	2.00E-178	66.08%	CUQ99298.1
**OR 45**	c82983_g3	YES	2.95	2.6	putative odorant receptor OR61 [Athetis lepigone]	0	61.94%	AOE48066.1
**OR 46**	c95547_g1	YES	145.27	209.47	odorant receptor coreceptor [Trichoplusia ni]	0	90.06%	XP_026726946.1
**OR 47**	c97124_g2	YES	4.34	2.65	Odorant receptor 50 [Operophtera brumata]	9.00E-12	70.59%	KOB74670.1
**OR 48**	c90701_g1	NO	14.06	9.9	olfactory receptor 58 [Ctenopseustis herana]	1.00E-27	52.34%	AIT69902.1
**OR 49**	c85150_g2	NO	11.58	1.96	odorant receptor 51 [Manduca sexta]	1.00E-47	39.66%	AFL70814.1
**OR 50**	c86324_g2	YES	4.62	3.05	odorant receptor 46a-like [Spodoptera litura]	0	78.17%	XP_022824662.1

**OR 51**	c95016_g3	YES	1.93	6.59	putative odorant receptor OR22 [Hedya nubiferana]	8.00E-78	34.89%	AST36260.1
**OR 52**	c95264_g1	YES	3.92	5.45	putative odorant receptor OR22 [Cydia pomonella]	5.00E-80	35.43%	AFC91723.2
**OR 53**	c97582_g1	YES	1.38	39.66	*putative odorant receptor OR22 [Hedya nubiferana]*	7E-97	38.35%	AST36260.1
**OR 54**	c97953_g2	NO	2.02	2.26	putative odorant receptor [Peridroma saucia]	4.00E-99	50.15%	AVF19632.1
**OR 55**	c97473_g1	YES	3.73	11.8	putative odorant receptor OR22 [Hedya nubiferana]	4.00E-91	39.81%	AST36260.1
**OR 56**	c83218_g1	YES	3.16	2.89	Olfactory receptor 35 [Manduca sexta]	4.00E-178	65.19%	CUQ99415.1
**OR 57**	c94541_g1	YES	4.44	2.22	putative odorant receptor [Peridroma saucia]	1E-150	0.5472	AVF19656.1
**OR 58**	c92431_g1	YES	3.43	0.68	odorant receptor 30 [Athetis dissimilis]	7.00E-126	47.36%	ALM26219.1
**OBP1**	c5357_g1	NO	0	0	odorant-binding protein 9 [Glyphodes caesalis]	2E-64	67.79%	QHI42038.1
**OBP2**	c97722_g1	YES	121.44	83.85	odorant binding protein, partial [Semiothisa cinerearia]	3E-57	76.92%	QRF70936.1
**OBP3**	c91262_g1	YES	373.02	243.5	general odorant-binding protein 72-like [Spodoptera frugiperda]	5E-71	82.03%	XP_035438151.1
**OBP4**	c81858_g1	YES	16.13	9.66	odorant binding protein [Semiothisa cinerearia]	1E-68	71.53%	QRF70928.1
**OBP5**	c98773_g1	YES	42.68	32.22	odorant binding protein [Dendrolimus kikuchii]	8E-49	48.00%	AII01008.1

**OBP6**	c91073_g1	YES	8260.58	3062	general odorant-binding protein [Semiothisa cinerearia]	7E-81	84.17%	QRF70945.1
**OBP7**	c105340_g1	YES	0	0	OBP8, partial [Ectropis grisescens]	6E-84	83.33%	UWK22035.1
**OBP8**	c86082_g1	YES	12385.1	15748	OBP6, partial [Ectropis grisescens]	2E-45	58.73%	UWK22045.1
**OBP9**	c92499_g1	YES	1763.28	933.65	odorant binding protein 13 [Apocheima cinerarius]	9E-65	69.66%	QOV03029.1
**OBP10**	c96014_g1	YES	24.19	16.1	OBP11, partial [Ectropis grisescens]	2E-118	95.26%	UWK22038.1
**OBP11**	c86352_g1	YES	10733.7	18308.2	OBP4, partial [Ectropis grisescens]	9E-84	82.19%	UWK22039.1
**OBP12**	c89262_g1	YES	51.75	31.63	odorant binding protein 6 [Apocheima cinerarius]	1E-80	76.82%	QOV03022.1
**OBP13**	c40316_g1	YES	3.37	2.54	putative odorant binding protein 3 [Ectropis obliqua]	2E-75	77.40%	ANA75017.1
**OBP14**	c38169_g2	NO	0	0	OBP4, partial [Ectropis grisescens]	8E-38	80.00%	UWK22039.1
**OBP15**	c84814_g1	YES	1.57	3.65	odorant binding protein [Athetis dissimilis]	5E-62	71.76%	QCF41950.1
**OBP16**	c89133_g1	YES	4.62	5.15	odorant binding protein 10 [Apocheima cinerarius]	2E-39	46.26%	QOV03026.1
**OBP17**	c98160_g1	YES	718.88	741.35	odorant binding protein [Semiothisa cinerearia]	3E-126	83.11%	QRF70939.1
**OBP18**	c93195_g1	YES	2337.51	1381.45	odorant binding protein, partial [Semiothisa cinerearia]	3E-72	73.51%	QRF70930.1

**OBP19**	c101141_g2	YES	21369.7	10709.3	general odorant-binding protein 2 [Agrotis ipsilon]	8E-90	93.62%	AFM36760.1
**OBP20**	c40601_g1	YES	0	0	OBP5, partial [Ectropis grisescens]	1E-40	45.89%	UWK22049.1
**OBP21**	c87421_g1	YES	5.36	13.74	odorant binding protein 10 [Apocheima cinerarius]	3E-65	66.23%	QOV03026.1
**OBP22**	c99252_g1	YES	5373.94	3095.86	odorant binding protein [Semiothisa cinerearia]	1E-61	82.84%	QRF70921.1
**OBP23**	c96085_g1	YES	107.93	88.54	odorant-binding protein 16 [Ectropis obliqua]	8E-82	91.73%	ALS03864.1
**OBP24**	c90549_g1	YES	845.42	571.52	odorant-binding protein 13 [Ectropis obliqua]	1E-82	80.82%	ALS03861.1
**OBP25**	c37612_g1	YES	0	0	pheromone binding protein 2 [Ascotis selenaria cretacea]	1E-72	73.24%	BAF64703.1
**OBP26**	c44553_g1	YES	0	0	unnamed protein product [Chrysodeixis includens]	6E-112	67.26%	CAD0201614.1
**OBP27**	c101109_g1	YES	2048.82	13067.7	pheromone binding protein [Semiothisa cinerearia]	2E-88	78.82%	QRF70941.1
**OBP28**	c146633_g1	NO	0	0	pheromone binding protein [Semiothisa cinerearia]	2E-30	86.89%	QRF70942.1
**OBP29**	c89101_g1	YES	2176.44	2555.54	pheromone binding protein [Semiothisa cinerearia]	9E-81	79.01%	QRF70943.1
**OBP30**	c93684_g1	NO	24.75	15.58	odorant-binding protein 34 [Adelphocoris lineolatus]	1E-11	60.00%	AXS78225.1
**OBP31**	c173626_g1	NO	0.00	0	odorant binding protein 8 [Apocheima cinerarius]	2E-41	52.86%	QOV03024.1

**OBP32**	c153732_g1	YES	0.00	0	odorant-binding protein 9 [Glyphodes caesalis]	1E-65	77.86%	QHI42038.1
**OBP33**	c94389_g1	YES	237.96	110.19	odorant-binding protein 4 [Cnaphalocrocis medinalis]	2E-115	66.00%	ALT31634.1
**OBP34**	c90778_g1	YES	39.22	41.77	odorant-binding protein 5 [Ectropis obliqua]	1E-55	58.22%	ALS03853.1
**OBP35**	c91709_g1	NO	0.77	0.95	odorant-binding protein 17 [Ectropis obliqua]	1.00E-31	78.67%	ALS03865.1
**OBP36**	c86364_g1	YES	105.18	54.84	odorant-binding protein 18 [Ectropis obliqua]	2.00E-41	47.26%	ALS03866.1
**OBP37**	c91913_g1	YES	10.46	9.09	odorant-binding protein 21 [Ectropis obliqua]	4.00E-60	67.44%	ALS03869.1
**OBP38**	c112322_g1	NO	0.00	1.32	odorant-binding protein 11 [Glyphodes caesalis]	5.00E-23	51.65%	QHI42040.1
**OBP39**	c74464_g1	YES	5.54	3.46	Odorant Binding Protein 38 [Dendrolimus punctatus]	1.00E-42	65.62%	ARO70197.1
**OBP40**	c146967_g1	NO	0.00	0	general odorant-binding protein 1-like [Trichoplusia ni]	6.00E-17	36.75%	XP_026738088.1
**IR1**	c100254_g1	YES	6.78	7.16	ionotropic receptor [Ostrinia furnacalis]	0	47.41%	BAR64803.1
**IR2**	c103282_g1	YES	27.37	20.36	ionotropic receptor [Ostrinia furnacalis]	0	75.30%	BAR64797.1
**IR3**	c103593_g1	YES	8.42	5.41	ionotropic receptor 75d [Conogethes pinicolalis]	2.00E-119	70.14%	QEE82787.1
**IR4**	c104376_g1	YES	52.26	23.2	ionotropic receptor [Eogystia hippophaecolus]	0	75.90%	AOG12845.1

**IR5**	c96640_g2	NO	13.10	11.52	ionotropic receptor [Eogystia hippophaecolus]	4.00E-133	68.09%	AOG12846.1
**IR6**	c82586_g1	NO	1.58	2.47	ionotropic receptor [Helicoverpa armigera]	8.00E-108	62.85%	AIG51919.1
**IR7**	c86876_g1	NO	1.49	0.95	ionotropic receptor 75a-like [Trichoplusia ni]	2.00E-51	45.75%	XP_026739905.1
**IR8**	c97080_g1	YES	6.01	5.86	ionotropic receptor IR64a [Cnaphalocrocis medinalis]	0	52.40%	APY22699.1
**IR9**	c104709_g1	YES	17.23	12.1	antennal ionotropic receptor 93a-2 [Dendrolimus punctatus]	0	77.68%	ARO70542.1
**IR10**	c74425_g1	NO	1.25	0.96	Ionotropic receptor [Operophtera brumata]	5.00E-148	61.63%	KOB72397.1
**IR11**	c85146_g1	NO	2.94	1.59	putative ionotropic receptor IR1.2 [Athetis lepigone]	7.00E-35	36.63%	AOE48004.1
**IR12**	c94321_g1	NO	3.23	1.28	ionotropic receptor 75a [Manduca sexta]	########	55.70%	XP_037298953.1
**IR13**	c96856_g1	NO	24.76	16.16	ionotropic receptor 93a [Bombyx mori]	5.00E-53	62.18%	XP_021203225.1
**IR14**	c99229_g1	YES	6.38	4.49	ionotropic receptor [Eogystia hippophaecolus]	0	55.61%	AOG12849.1
**IR15**	c104365_g1	YES	6.64	6.22	Ionotropic receptor [Operophtera brumata]	0	55.87%	KOB72673.1
**IR16**	c97874_g1	NO	2.77	30.15	ionotropic receptor 75a-like [Trichoplusia ni]	4.00E-167	65.98%	XP_026739905.1
**IR17**	c100995_g1	YES	76.23	39.24	ionotropic receptor 76b [Heortia vitessoides]	0	66.67%	AZB49414.1
**IR18**	c30936_g1	YES	1.85	0	ionotropic receptor 64a [Conogethes pinicolalis]	5.00E-45	60.87%	QEE82786.1

**IR19**	c96007_g1	YES	7.20	3.44	ionotropic receptor 1 [Conogethes punctiferalis]	0	81.27%	ARO76464.1
**IR20**	c94900_g1	NO	6.08	5.86	ionotropic receptor [Eogystia hippophaecolus]	7.00E-103	50.00%	AOG12846.1
**IR21**	c100026_g1	YES	5.60	4.33	ionotropic receptor [Eogystia hippophaecolus]	0	56.32%	AOG12846.1
**IR22**	c102739_g1	YES	5.84	3.97	ionotropic receptor 75q2 [Ectropis obliqua]	0	82.38%	ANA75034.2
**IR23**	c89009_g1	YES	1.47	1.49	antennal ionotropic receptor 68a-2 [Dendrolimus punctatus]	0	76.74%	ARO70548.1
**IR24**	c101405_g1	YES	64.31	53.73	putative ionotropic receptor IR25a [Cydia fagiglandana]	0	94.38%	AST36358.1
**IR25**	c97011_g1	YES	2.11	2.1	ionotropic receptor [Dendrolimus kikuchii]	0	62.03%	AII01128.1
**IR26**	c93917_g1	YES	72.46	1.49	ionotropic receptor 40a [Spodoptera litura]	0	82.41%	XP_022834254.1
**CSP1**	c36178_g1	YES	0	0	chemosensory protein 5 [Ectropis obliqua]	2E-64	76.12%	ALS03830.1
**CSP2**	c36822_g1	YES	0	0	chemosensory protein 20 [Ectropis obliqua]	3E-52	88.04%	ALS03845.1
**CSP3**	c73160_g2	YES	16800	15105.5	chemosensory protein [Semiothisa cinerearia]	8E-61	70.87%	QRF70953.1
**CSP4**	c62076_g1	YES	1.52	1.46	chemosensory protein 22 [Apocheima cinerarius]	3E-51	80.95%	UNG39416.1
**CSP5**	c78954_g1	YES	5500.89	13398.4	chemosensory protein csp2 [Helopeltis theivora]	4E-41	60.91%	QCX43082.1

**CSP6**	c95000_g1	YES	206.75	126.15	chemosensory protein 16 [Apocheima cinerarius]	4E-53	80.65%	UNG39410.1
**CSP7**	c85738_g1	YES	892.27	950.71	chemosensory protein [Semiothisa cinerearia]	6E-48	78.57%	QRF70949.1
**CSP8**	c96652_g1	YES	69.74	83.3	chemosensory protein [Semiothisa cinerearia]	3E-40	66.07%	QRF70954.1
**CSP9**	c42077_g1	YES	0.61	3.99	chemosensory protein 2 [Apocheima cinerarius]	1E-54	82.86%	UNG39396.1
**CSP10**	c87487_g1	YES	72.5	83.12	chemosensory protein 3 [Apocheima cinerarius]	1E-42	60.17%	UNG39397.1
**CSP11**	c72957_g1	YES	3948.04	3230.06	chemosensory protein 11 [Ectropis obliqua]	3E-20	42.16%	ALS03836.1
**CSP12**	c88811_g1	YES	2754.02	3243.12	chemosensory protein [Semiothisa cinerearia]	4E-74	88.37%	QRF70950.1
**CSP13**	c77911_g1	YES	2433.26	3051.17	chemosensory protein 24 [Cnaphalocrocis medinalis]	3E-56	69.53%	ALT31606.1
**CSP14**	c90769_g1	YES	25.28	12.08	chemosensory protein 8 [Apocheima cinerarius]	6E-60	88.43%	UNG39402.1
**CSP15**	c34905_g1	YES	0	0	chemosensory protein [Semiothisa cinerearia]	9E-40	59.84%	QRF70953.1
**CSP16**	c89154_g1	YES	893.33	1237.05	chemosensory protein 6 [Apocheima cinerarius]	4E-42	79.12%	UNG39400.1
**CSP17**	c88833_g1	YES	22.02	11.49	chemosensory protein 7 [Apocheima cinerarius]	8E-50	84.55%	UNG39401.1

**CSP18**	c98382_g1	YES	33.07	34.96	chemosensory protein 5 [Ectropis obliqua]	1E-143	80.79%	ALS03844.1
**CSP19**	c127389_g1	YES	0	0	chemosensory protein 2 [Ectropis obliqua]	5E-32	48.06%	ALS03871.1
**CSP20**	c132300_g1	YES	0.00	0	chemosensory protein 11 [Ectropis obliqua]	1.00E-16	38.61%	ALS03836.1
**CSP21**	c179234_g1	NO	0.00	0	chemosensory protein 7 [Ectropis obliqua]	2.00E-46	80.68%	ALS03832.1
**CSP22**	c74821_g1	YES	1.29	1.99	putative chemosensory protein 1 [Ectropis obliqua]	3.00E-71	89.08%	ANA75019.1
**CSP23**	c80360_g1	YES	4.24	3.74	chemosensory protein 1 [Ectropis obliqua]	1.00E-50	63.93%	ALS03827.1
**CSP24**	c80581_g1	YES	1.82	1.98	chemosensory protein 21 [Ectropis obliqua]	1.00E-61	72.66%	ALS03846.1
**SNMP1**	c101718_g1	YES	168.55	283.33	sensory neuron membrane protein 1 [Ectropis obliqua]	0	72.52%	AKN78948.1
**SNMP2**	c103761_g1	YES	843.07	1365.34	sensory neuron membrane protein 2 [Ectropis obliqua]	0	84.08%	AKN78949.1

Table S3. qPCR primers for tissue expression profiling.

**Primer name**	**Sequence**
CSP19-F	CAACGCTGTGACTACTGCT
CSP19-R	TCTTCAGAACATCGTATTCAGAGG
CSP20-F	CAGTTAATGCGGGTATCCTAAGA
CSP20-R	GGCGTACATTCTACACATCCA
CSP21-F	CGAGGCTGCTGAAGAAATACT
CSP21-R	CATTGTTTTGAAAGCGTTGAAGG
CSP22-F	AACTACAATACTGACGCCATCAT
CSP22-R	CAACTTGCTTCTGTTTAGGACTG
CSP23-F	TGACGACAACCACTACGATAC
CSP23-R	GCACTTAGCACAGCCATTC
CSP24-F	GGCAAGGAACTCAAGTCTCA
CSP24-R	CTCTCCAGTAAGTTGGCTCATT
OBP30-F	CTCAGCGGAGTGCCAAAC
OBP30-R	CTGCGGTAGTATTGATCTCTAAGC
OBP31-F	GTCGGCACCATATTATCCTGAT
OBP31-R	AGGTGTTGTATCTTGGAAGCAT
OBP32-F	GGTAATGTGCTATATCGCTTGC
OBP32-R	TTGTAGTTGTTCTGTATTGGCATG
OBP33-F	GCCTTCCATAGACCCTTACAAAC
OBP33-R	TCCTTTCATTCCCTCCACATAGA
OBP34-F	GTCTTCGCTTGTGCTCTCA
OBP34-R	TGTTCGGTTCTCTGCATAAGTT
OBP35-F	TGGTAAGATTATCCTGTCCTGTCA
OBP35-R	CTCGTCGCCTTCCTTGAATATC
OBP36-F	TAGACCCGTGCTTTATTGCTT
OBP36-R	TTTCGTCGTTCACTGATTTGC
OBP37-F	AGTGTCTCTTGGCTTGTGTT
OBP37-R	TCTCGTAGGGCTGTCGTTA
OBP38-F	CGGATGACGCAAATTGTATGAG
OBP38-R	ATCTTCTTAGGTACGCAGTTAGC
OBP39-F	GGTCCTACACCGCAATCC
OBP39-R	GTCCTCTTGTCGTTCTCCTG
OBP40-F	ACGCTGTTGCCTTCTTGT
OBP40-R	CATTCTTCTGTGACTGGACTCA
OR1-F	CTCTCAGAAGACCATGAAGTTGT
OR1-R	GTAGTGTAGAACCTCAATGTCGTA
OR2-F	GGACGCCAGTTCAGTGTT
OR2-R	ATAGGCTGAGTAGACCGACTT
OR3-F	GTGTCTCTGCTGTGCTTCT
OR3-R	CAAGGTTTCTGGGCTCTGAT

OR4-F	GCGTCCTACATCATCAGCATA
OR4-R	GCCGTTTCGTATTTAACTTCCTT
OR5-F	CCGACGATTTGGAAGACATTTAC
OR5-R	GCCTGGTATCTGTGAAGCAT
OR6-F	TAACGACTTAGAGAACGCCTTC
OR6-R	CAGCATACCAACACAGCATTC
OR7-F	ACTTGGCAACGATAAGCAGTA
OR7-R	CAACAACAACAGTGGTCATCAG
OR8-F	CTCCACTCGGTGCTGTATG
OR8-R	CTGCGAAGTAGGAATACGACAT
OR9-F	CATTCGTTCCTCTCCTTCCTAC
OR9-R	CAACAAGTCTTCCAAATTCCAAGT
OR10-F	ACTGACATGCGTGATGGTATT
OR10-R	CGAATTGAACCTCTCCTCCTTAT
OR11-F	TCAAGCACTTACTTCTCGTTCA
OR11-R	TCAATCACTTCCATACAAGGTCTG
OR12-F	TCCTTGCTATCTCCAGTTATGTTC
OR12-R	TGTGTAAGACCTCGTTGCTATG
OR13-F	GGGCTAAACTGGGATAGAATGT
OR13-R	GATGATGCTCACTGCTGGT
OR14-F	ACTATTACCTGTTGGACTGGATC
OR14-R	ACACTTCTAACTGTGCTATTGTCA
OR15-F	TTCAAATGACAGCAGAAGCAATC
OR15-R	CCAAGGCACTCCGTACAC
OR16-F	CATATCCAGGTTTACAGTCAGTCA
OR16-R	TTCAAGCACAAGTTCGTTAAGATG
OR17-F	GGTCATTGCTGGTGCCTAT
OR17-R	AGCCATCTTTATCTGTTCAGTCTT
OR18-F	ATGCTTGGAGTCAATGGAGTT
OR18-R	GTGGCGGAAGTAAGTTGGTA
OR19-F	TTGCTGCCTCTGATCTGTC
OR19-R	GTTCAACCAAGTCTCCTAATCTCT
OR20-F	GCCAACAGAGCAGCGTAT
OR20-R	AAATCGAAGACAAGGACCAGTT
OR21-F	CTGATGATAAGGTCCAAGAAGCC
OR21-R	CGGTGCGTAAGAGTGTGAAAT
OR22-F	TCTAAGCCACGCAATGAATCT
OR22-R	CTCGCACCATCGGCATAT
OR23-F	GGATTAGGACCATTAACACAGACT
OR23-R	CAGCAGTGCCTGATAGTTGA
OR24-F	TCTGCTTCTGTGGCTTCTG
OR24-R	GTGATAGATGGCGTGTGAGAT
OR25-F	TCGTCAGCAACAGTTCACA

OR25-R	GGTCGGGATGGAGAAACAAT
OR26-F	CTTGGACTTGTGCTGTTGAAC
OR26-R	CCAGGCTGACGAGTATGC
OR27-F	GGCTGTGGAACTCCGTAAT
OR27-R	CCTTGGTAGCAGTAGAGGAATAC
OR28-F	CTCTGCTCTATGCTGCTACC
OR28-R	TACGCCGCTGTTGATACG
OR29-F	CTGGCACACACTTACCTGAA
OR29-R	TTTCTTCGCTGAGGGACATC
OR30-F	ACACGCTATTCCACTTTCTTCT
OR30-R	TGCCAGGTAACGATTCTCTTC
OR31-F	GTTTGAGTATCCCAGAAGCAAGAT
OR31-R	GCTGACCACACTTTACACATAAGA
OR32-F	CGTGGCAGTCGTATCATCA
OR32-R	GAAGTCAGGCAGCATGGT
OR33-F	GGACTTATACTGCCGTCATCTAC
OR33-R	ATTCTGATCTCGGTATCCATTCTG
OR34-F	TAACCTACTCCTACGGCTTCA
OR34-R	AAGAACGCAAGGGATGTGAT
OR35-F	AAGTTATGGATAGCAAGGAGTTCA
OR35-R	TCGTCATCGTCGCAATGTTA
OR36-F	TTCATCAGTGTTGGTCAGAAGT
OR36-R	GAAGACGGAATTGGTTACGAATG
OR37-F	TTGTGCGTCACTGCGTAT
OR37-R	CCTGTCGGAGATACCAATAACTT
OR38-F	TGTGGAATGCTGGAGATGTC
OR38-R	CTGGTTTCTGTGCTTGTGTTATAG
OR39-F	CGTGGCGACATCAGTGTA
OR39-R	GAGGAAGGTGTTAAGCGAGAG
OR40-F	ACCGTAGCAATGATATTCCAAGT
OR40-R	CGCAGTGAGTACGATGATGT
OR41-F	TCTTTGGTGTTTAGTGGGATGT
OR41-R	ATGCTGTGATAACGGTAGTGAAT
OR42-F	TGGAGCGGCATTCTTCTG
OR42-R	AACGGTTGTGGTGGTGTT
OR43-F	GCTGCTGGTGATGATGGT
OR43-R	ACTGTGAAGAAACTGTACGAAGAT
OR44-F	AAGGTGGGCGATGAAGTAAG
OR44-R	GTTGCCAGTCAGAAGAAGGT
OR45-F	AATACTCTGCGGCGTTGAT
OR45-R	CAGGAGCATCGTCAGTCTAC
OR46-F	CCTTACGCTCCTGGCTTAC
OR46-R	CCGCTTCCATTACCGATGA

OR47-F	GCAGGACTACAGAACACATACA
OR47-R	CAATGGTAAACGGCTCTCTCA
OR48-F	TACTCTTCAGGCTGGCATTACT
OR48-R	GGACTGATACGACAAGCTCATTAG
OR49-F	CGAATTGAGGCAGATGATATACCA
OR49-R	GCACTCCCGCTTTCACATTA
OR50-F	CCTTCATCGCTTCGGCTAT
OR50-R	ATCCATTGCTCGTATTGCTCTAT
OR51-F	CATACGGACTTTCTATACCACTGT
OR51-R	GCATAGCACCATAAGGACCAT
OR52-F	CAAGGACTGCTGTTCTACTGT
OR52-R	TGCTCATATCAACGAGGTTACTT
OR53-F	CTTATTCTCATCTCTGGCGTTCT
OR53-R	AAGAGCCCTTTATCTCCAATGTT
OR54-F	ACTTGCGTGCTCATTCCA
OR54-R	CCCATTTGCTCCGACTTGA
OR55-F	CCATCACATCGCTCAGGAAT
OR55-R	GTAGTCAAAGGGCATCCAGAA
OR56-F	GTCTGGTTCAACTGCTTCTCT
OR56-R	CGCTCCTGTCTGCTTCTAC
OR57-F	TCAATCTGTCCTCGGCTACTA
OR57-R	GCTATACCCTCACTCGCTTC
OR58-F	CGAGTGAGAAGTTCCGTCAA
OR58-R	AGACCGTGCTAATACTGAATGG
SNMP1-F	ACCGCTACACAGTCAACATT
SNMP1-R	TTCAGGCAGTTCATCAGATTCA
SNMP2-F	CGAACTACGCAACATTCCC
SNMP2-R	ATAGCGAGCAGCAACCAA
IR1-F	GGAATGATAGCAGCCGTGAT
IR1-R	TAGCCCTTAGCATCTCTTGTTG
IR2-F	TTAGCAGCAACCGCACTT
IR2-R	ACGACTTTCAGCATCATCAGAT
IR3-F	GGAGACCGAGCCTACCTTA
IR3-R	TCATTCCGCTGTGCTTCTT
IR4-F	CGCAGATTTCGCATTCATTCA
IR4-R	TGTTCCAGTATCCTCTCCTTCT
IR5-F	AGGCTCAGAGGCGATGTT
IR5-R	GACCACGAGGCATCATAATCC
IR6-F	TTGATACTCGCTCTTGGTTACAT
IR6-R	GGTTCGTTGTCAGCATAGGA
IR7-F	AGTGAGCAATAACGGATCAGATT
IR7-R	TGAGAGTCAAGTTGTGGAAGTT
IR8-F	AGCAGATAGATTACACGCAGTC

IR8-R	CAGCACCACAGCAGATAAGA
IR9-F	CAAGAGTGCGATTCAGACAATG
IR9-R	GAGTTCTTTAGGGCGTGCTAT
IR10-F	TCTTACACCTGGGTAGTTCCT
IR10-R	ATCTGTTGCGTGGTGAATCT
IR11-F	TGCTGATATTACGTTGGCTTCT
IR11-R	CTCCAAGACCCTATATCTGTTACG
IR12-F	CACACTAGCCCACAGTCAAT
IR12-R	TATACAAGCCTCTCAACAAGCAT
IR13-F	GGAAGGGAGTTCAGAGACAAG
IR13-R	TACTGACGAGGTTGGTGAGA
IR14-F	TGGGCTTAGAAATACTTGTTGCTA
IR14-R	ACGATTCCTCAATATCATCCACTT
IR15-F	GTAGACCACATAGTAGCGTAACC
IR15-R	CTTACAAGGATGCCATACCAGAT
IR16-F	GCCAAGGTCGCAAGATGA
IR16-R	CCTGGTGTAGCCGATGTC
IR17-F	GCGACCTTCTGATGACTTACA
IR17-R	GCGTCCTGCTTGCTTACT
IR18-F	CAGCGTAGGGAAACAGACAT
IR18-R	GGATAAATAACCACCAGGGAACT
IR19-F	ATGTTCGGTTGGCTCTGTT
IR19-R	CTCTCGCTGCTTATCTGCTT
IR20-F	TGCCAAAGGATACAGTAGATACAC
IR20-R	GTCCCACTGATTCAGAACCAT
IR21-F	GAGAAGCGTTAGTAGACATGACA
IR21-R	GCATAAGACCTGACTGTTGGA
IR22-F	AACGCAGGACGGAAATACTATT
IR22-R	GGAGGTGAAGAACATTGGAGAT
IR23-F	ATGGAATGCTACTCGTTGTATCTT
IR23-R	CTTGGGACTGGATTGGCTAAT
IR24-F	TCAGATGGTAGGAGACGAGTT
IR24-R	GCTTGTTAAGGAGTTGGAGGAT
IR25-F	ACTCTTCGCATTCCACTCG
IR25-R	TTGAAAGCCACCCGCATAA
IR26-F	AGTGAGGCTTGTGCTGTC
IR26-R	GCTATGGCGGAATATCTCGTAT
GAPDH-F	CGATGCTGCTGCTGGTAT
GAPDH-R	CTTGGTCTGGATGTACTTGATGA
GTPBP-F	CGAGCGAAGGAAGTGGATT
GTPBP-R	GGAACAGAATTACCGACGAGT
ACTIN-F	CCTCCACCATCAAGATCAAGAT
ACTIN-R	GCCAGACTCGTCGTATTCC

Supplementary file S4. The amino acid sequences used in phylogenetic analyses.
>SsubCSP1
MLSLYLLSIFILVHVYCEEEYTNKYDGVDLEEILANERLLSGYVKCLLDLGPCTPDAKELK
KNLPDAIENDCEKCTKRQREGADQVMHYIIDHRPDDWLKLEEKYNEDGSYKRKYIASKQ
PEDNKSAEKPEDNTEKSEENKKS*
>SsubCSP2MKLAIVALCLVAAVLADDKYKDISDDFDITEVLDNERLLNSYAKCLLNKGPCT
PEVKQVKDKLPEALETRCAKCTDKQKQMGKQLAREVKKHHPGLWKDLVAMYDPEGKH
QQAFQDFLASQ*
>SsubCSP3
MKAIVCICFVGIIAVALARPNEEKYTTKYDDVNVDEIISNERLRKPYVECVLDHGRCAPDA
KELKSHIQEALENNCGKCNEKQQGAVRKVIKYLINNKEDEWKQLVAKYDPENKYSKKYE
HELRDVKA*
>SsubCSP4
MQIIPLTLLMCACVMHTAISAPQLTDAQLEQTLNDKTTMQKHLRCALGEGPCDPMGRRLR
TLAPLVLRGACPQCSPQETHHIRRTLAYVQRNYPWEWARIVRQYG*
>SsubCSP5
MKAIVCVCFIAVVAVALARPNDDEKYTDKYDNVNLDEILVNDRLRGPYLKCMLESGPCAP
DGKELKSHIKEALETHCAKCNKKQEDGVRKVIKYLINKQKDEWDQLVAKYDPDNKYSK
KYETELKEAEA*
>SsubCSP6
MKTILVLCALVAVVVCRPEETYSTQYDNFDAHELAGNVRLLQNYAKCFLDKGPCTAEGTD
FKKTIPDAVKTKCAKCNPKQRELIRIVVKALQDKLPTYWDELVKKEDPEGLYKDDFNAFI
KATD*
>SsubCSP7
MNWVILSAVLAMAAFVAAADSDQYTDRYDELNVEQIMANRRLLVPYMNCVLDKGKCTA
EGKELKAHIQDAMQTGCSKCTEKQRQKARKAVKQIKENEKDFWEQIKSKYDPGNKFKET
YEAFLAAEN*
>SsubCSP8
MKAVILCVLAVAAISLARPSNNDTYPSKFDNINLDQILGNRRLLMPYVDCVNGENKKCTA
EGRELKAHLGEALETNCAKCTDAQRKGARRVIAHLINNEPQAWNQLKAKYDPSGKYSKK
YEAELAKVKG*
>SsubCSP9
MNAILFLTVVVALVAPLVLCYDEIYDKMDVDKIIADEALFDAYINCMLNKGPCDVEHSTEF
KKLLPEVIATSCAKCTPIQKKHVKKTVKAIGEKKPAELIEFKKLYDPNGEHEKEFTAFVMSP
DVD*
>SsubCSP10
MKSFCIFVLALVVVCSAEQYSAENDDFDIDKAVEDDATLKSMVDCFTDKDSCTPLYAALK
EDMPDAIERGCDKCTDAQKHMFKVYLDALKKRFPAENEYYRQKFDPNNKYFATLEAAVS
KY*
>SsubCSP11
MVSDNQDTIISAFSETSIQRRYKMKSIILFCTVLAVALVGGEDLLKEEDALFVQDETAFQTL
YACLMDKGPCEGKMATYKDMIPKLLKTNCESCSPKDKELFEKFSKIAADKHPQEFKELM
DKLTPKAQ*
>SsubCSP12

MKLLLVIALACMAAVAYGKPATKYTDKWDNINVDEILESERLMKGYVDCLLDRGRCTPD
GKALKDTLPDALENECSKCTEKQKNGSDKVLKHLINKRPDFWKELAVKYDPDNKYQAR
YKDKIESVKGKA*
>SsubCSP13
MKYLLVLACLAFGVFAEDSKYTDKFDDINIEEILGNERLLMNYHECLMSRGKCTNEGKEL
KANMQEAIETGCAKCTEKQQKGCYEVIEYLIKNKLDMWKELCEKYDPEGKWRKKYEDR
ARANGIVIPDN*
>SsubCSP14
MRAFIVLACLVAVALAADKYNSKYDNFDVDTLINNDRLLNAYVNCFLDKGRCTPEGGEF
KKALPEAIETTCGKCSDKQKTNIRRVIKAIQKKHATQWVDLVNKNDPTGAHRDRFEKFIQ
GS*
>SsubCSP15
MKATLCICLALVAVVLARPNEEKYTEKYDNIDVDQILHNHRSFIGYVACVTDKGKCTPEG
KTLKDILKDALETDCIKCNEKQKATTRKVIKYMINEKQAQWEEVAAKYDPDNIYRKKYE
DELREIKA*
>SsubCSP16
MLGIVVFLVLIVNFVTSDFYSSKYDDFDVQPLIENDRIMLSYTKCFLDQGPCTPDAKDFRK
VIPEALETSCGKCTNKQKQLIRKVIGAVMDRHPEAWKELTDKYDPEAKFKESFDKFIAEKE
*
>SsubCSP17
MQISYALVLFTLAAACTAQTQPQQRPQVSDTALEDALNDKRFIQRQLKCALGEAPCDPIG
KRLKTLAPLVLRGSCPQCSPQETKQIQKTLAYVQRNFPQQWTKIVRQYAG*
>SsubCSP18
MRAVLTLCVVCVCAVMCQDTQDMSAMPKYDSRYDYLDVDAIFTNKRLIRNYVDCLINSN
RCTPEGKALKRLLPEALRTKCVRCTERQKRTAVKIIKRLKYEYPDEWAKLSQRWDPTGDF
TRYFEEFLAKEHFNTIPGSGVVLPLSTGTKDPSLQPPAPTSPIPASEPTPRPTSGPVVPVTVQS
TSPRPVILNRFGDDGELMMGSPSSAAGTTRPTVPVTMRISTVRPYMQTRMPPSIGVASNAI
PTRFPLRPNDLPPPYSTAITLIDQIGYKIIRTTELVTDLLRNTVRAVVG*
>SsubCSP19
MKSFCIVISSLLIACAADYYTSDQSIDVEALVTDMTMMQSFVDCLTGKGECNAVTTAVQK
EFPEALSTACAKCTASQKHKVKIFLASFNKNFPSEYDVLKKNLDPESKMFPAMEAAVAEY*
>SsubCSP20MKLFIYFYAAALASIAVNAGILREENELLIRDEKLIQTLNDCLLENIPCSESFVQ
YKAIFMVYLKTGCVECTPEVKNLFTKHRDIVAQLYPEKNSDMYARLTL*
>SsubCSP21
QEKYYDKKYDYYEIDYFVNNSRLLKKYLDCFLTQGPCTPIGRVFKEVLPEVIITACNKCTP
LQKRFTKKAFNAFKTMLPEMHEELKKKYDPQNVYYDSFEKAIS
>SsubCSP22
MQSWSLIVFALTVVVASAQQYPNRYENYNTDAIIQNERILLAYYKCVMDKGPCTKDGKNF
KRVLPETLTTACARCSPKQKQVVRKMLLGIRAKSEPRFIELLDKYDPEQANREALYKFLVT
GV*
>SsubCSP23
MKLLILIAMVVLASARPDDNHYDTKYDNFNIEEVIENKRLMCSYGDCFNAAGKCTPEGN
DIKKWIPEGSQNGCAKCTAKQKTLVAKFIKGLQTVCPDRWETLSKKADPEGKHQDELKKF
LEENS*

>SsubCSP24
MRTATVLCLLATVAFAAAKPGASQYTDRYDNVDLDSILKNKRLLVPYIRCALEQGKCTAE
GKELKSHIKEALETYCEKCTNVQRDGTRRVIGHLINNEPTYWRDLSAKYDPSHKYVVRYE
NELRQIKA*
>SsubOR1
KKPFYFREKVRRIASTSLACDALVVPESRFSKNLQKRLRTVKKRAIVFWVVMMGNGVVY
LMRPFVTPGRHLMEDTFMYYGLEPMHETPNYEIAITITIIGVMFACFSVVNVTAIVIINVGY
VEAQMLSWSEELLHLWDDVKHSVTRTRTTKAMTDPTEYAVRQTTVNKLVKSHLRAVIRG
HARNLDLLRQIEDVYRGAFFLEFLILIIGLIAELLGGLENTYIEIPFSLIPVVVDCLTGQRLAD
ASLIFERAVFECKWENFDKSNKKLILLMLQNSQKTMKLSAGGLAVLNFSCLMFVFRLVYS
AYTTLRFYTTSTSS*
>SsubOR2
LMMMMMCLFLTSAKHLTSKAKVLTLKMSYRDNFLQLCLYFKVCSRRKVREIVGQCLAC
DALVIPGSRFSVILMKTLRVVKKRAMVFWILIIGNGVVYTLKPILSPGRHLQEDIFVIYGLEP
MFESPNYEIAFLIITIGVFVTVYLPANITAFVIIICGYIEAQMLTWSEELINLWDDAEKQYIKE
VTDENINDSRETKSEVINELVRQRLKTIIIGHSKNITVLRQVEDVFRTAIAVEFSLLVVGLIAE
LLGGLENTYLEIPYALMQVAMDCLTGQRLMDASSVFERAVYACHWEALDQDNMRLVLV
MLQNSQRTMTLTAGGVTTLSFCSLMAVIKSVYSAYTTLRSTMK*
>SsubOR3
IQQIFSVYLTTKHSSNSNKFFSLFSAIIVCLNIFGTETLFYSLCTYMEMHFRILCHQLGNIQAR
TTTQETLKAGVRRHQLLIQLVEKTEELFSRPVLFHFVSSSLLICLSAFNITVAKEIGSVVAFV
TFLLLSLSQVSLLCFFGDKLMRSSTEVSGAVYRSPWYDFDVNAKKAVLIIIIRAQKPCKLTA
SNFADLNLRAFTTVLSRSWSLFALLKTVYN*
>SsubOR4
MRFPNKTAAHYYKTSFIVFFLGSPNFWIEDLEVSKTFYKVFRHISRFLNGILACFIVFELGSF
LTQKNLTKKQETDLMIYGISHPLLMCYTLILSRHDEDIKKLFRHILRLKSFYNDKEIEEIML
KKSKRDSTALALSCVLSLVLHTISALMQYMKSEGTFTTVITSWPLVDDPSRVAHAARIVNN
LMWWVFVSRVIASYIISIVLVTSVSHQYMNLQSYFYSIERVFHNTSLGQREKEVKYETAFK
LGLKFHANTIECVSQFRHICNGIFSAQIVFVMSMLVFLMTQMMKSERTMVNAAATAITATS
VLIATGFYLWNAGDITVEAGRLPTAMYCSGWHRCRGAAAVRVRRLLMIAMTHAQKPVEI
KGLGMITLSYRCYLSLVKSSYSVFSVIY*
>SsubOR5
MEMPNYDDIFKLIKFNIWLIGIPYGNFNFTWRYYFFFFSLFLMIIEEAGFLISKVNSVNMLEL
TQLAPCTCVGLLSVLKIMAISLKKQKIFELSESLSHLYGNITIESESGPVIRSQLILLKKLVRY
YFVLNMMLTSVYNFSSPVIIFCNYLKSGEVSFVLSYQVLLPFEINSWWLWGIVYVHSVLSG
FVCTVYFTTVDVLYCALTTHICANFVSISNKVKLVDKTNVAMFQDLIKNHQKVLKLSDDL
EDIYTAPNLFNVLVGSVMICALGFNLAMGDASQIPGCILFLASVLLQILIMSVYGENMITES
TGIGDAAYTCKWYEMDEKTKKVILTLMIRSKKPAKLTAYKFSIISYASFCTILSTSWSYFTIL
RTVYTPPESY*
>SsubOR6
MITSTVEQAKSEIDDSLKLSVFCMRRIGLSFENPKGTAAYFKQQLMFVLSVCGICYHVFSEI
VYIGLTLSNSPRVEDVVPLFHTFGYGALSIAKVFVLWYKKDVFTKLLDELAEIWPLDPLNE
EDQIIKQNSLDALGIAHRWYFTVNVLGVWFYNVTPIVVYFYEMWRTGEAVIGFVWVSWY
PFDKHQPVAHVLVYIFEVFAGQTCVWIMVCTDLLFSGMASHIGLLLRLLQHRLECVGTTD

QTDEEHYKEIVENIKLHQRLIKYCNDLENAFTLSNFVNVVLSSVNICCVVFVIVLLEPFMV
VSSKLFLGSALIQIGMLCWYADDIFYANAGVALAAYKGNWYETNPRCRRALLFLVKRAQ
KPIAFTAMNFTNITLVTYSSILYRSYSYFALLYTMYSEN*
>SsubOR7
MEPKSFSSLKNNFFRDLDYLTNKISKIFVYPFVGRSKNVIRCYNIMRICLFITIFQLTASLLIS
KFKDWFEIINIAPNLGVCILIFIKYTKIHTSQQVYDNFLTHFKIGLWNIIDMDSEKQRDIFERY
TRISKMILLFQYYYMIVLVAIVISFPRIIMFLNTNILGNDKQYLYPFNGWYPFDKVKWYNV
VYVWESLMTTVVVVLFGFANMIHLSFTRHICMELTILGTTMEDLISREDRKNITQRIFAKET
HHIIRNKLKKIIKTHQILVGMVDDFEKVLGSGMLLIYIFGSVFICLTILTALMVDDLYMSLRY
FCFFLSLLIEVFIQCIIGQMLIDHSESLEQAIYFSSWSYADRETRQMLLIFLIRAQRPLKLTAK
GYLVMNLSTFAGVCSLSYQFFNLLRTVYT*
>SsubOR8
MTLDSEQLYLNRAKFVMKLLGVWVPPDKETLLQKIYRNFMITLQYLFLVFQLVYIIQVWG
DLEAVSQASYLLFTQACLCFKVTVFLVNKNMQKELLKQMKSNIFSAQSVLHEKILKVQAT
RIKRFLFAFMISSQLTCGMWALKPLFDDAGSRNFPFDMWMPVGPEKSPQYHLGYAFQLLTI
CMSAYMYFGVDSVALSSVIFCCAQIEIIKDKILSIETVWDKKRQHEVTLTRSKNYMKLVECI
KHHQAVVSFIQLVEDANHTYLLFQLTGSVGLICMSALRILVVEWKSMQFFSILTYLSVMISQ
LFVCCWCGHELTATSEELHSVLYECSWYEQSVKFQKDLVFAMMRMRRPLVLKAGHYTPL
LRQTFVAILRMSYSYFAVLNQTQEK*
>SsubOR9
MKLHQIRRRFMAYFNKEDFDPSQGIVDPLMYHSMFISFAKLFNVMDYENRPWWVYLSTI
CTAISASIAVTLFIIALTHYFQIRSVHLITEAGTYLLYMGYKLTLVFSIHNGKKHYYYFLQAIQ
EDYKYICTRGQKYRLKFFENQLLTFRACILVAVFTLVMGSGVGLCACIAGALTRQLPYPYW
LFDYNIRSSPSYEILFILSVLFTFFHTYQYIFNMQSQVLWIREITSKADIITWNLEDLLVDVQP
AKNKEEEAFYTALIKHRMREIISMHHSMILLMEHYASVFKKCLLYEQMAAAPTICLTAFCF
AEKFEDGEFEITMLLLCTAAIGVVFVPSYLCTHLLEKVQSVGDACYDIPFWNAGPVIRPYV
VLVMQRSMRQLPTVAAGFEDISLATYSRLLTRSYSCFNMLRQADFNFT*
>SsubOR10
MEDAIIPEFKPFRETYKMNTYALCVGMIYPNPKTNRWRLAAIPAVLLTATPLAVTIFIDMYF
SWLDRDIVNIIRHSTVVGPFLGGFFKMILMYYKRVEAKQILDEIDRDYESYNHLPPDYKQI
AGQAVRTSLVYCERTWVIIVLTCVMVFPSMAVVLNLYNFMFKPEPTKYMIHDLNKPFADK
EERFNSPYFEIYFVYEFYCSILYVINFIGYDGFFGLAINHACLKMDLYCKALEDALKGPHYV
MHEAIVAVIQDQNRLFQYVNWIQDTFNIWLGIIFLATMIQICTCLYHITEGFGFDLRYMIFVT
GAVIHIYLPCKYAAKLKAMSAETGTKIYSCGWENVPSQSIRKMILFMVARGQVPIVITAFN
MMVFDMELFVSIMQSSYSMFTLLRS*
>SsubOR11
QKTKNRITIKLTFKMSDTALLFDQSLQKLNILFILSGMKIKYQTRTPFDYIKDRWLYIFNFIW
VLSVLIPCVCYVILAAAQGKDFVEVTSLAPCLTFSTLGQIKSIFHLINESEVFKLINSLKELEI
KENKRKHSVEKEKIIKEDMHFLNRVLNVLYFFNCCMVLVFGLTPFILMAVKYIRTHEFEML
LPYFDFFVFIPYEVKYWPFAYIQQVWSECIILLQLGAADYLFFTCCSFIKIQFRLLQRDFEKII
TKSKSIDTDVVVDEQFKEKFVELVKWHQDLIEASNKLEIIYSKSNLFNFLSSSLVICLTGFN
VTISNDFVVIMTYLTFLFMALLQVFFICFFADMLMTASIEVSDAVYNCRWYDAQLVEVKH
LLLVQTRAQTPCTLTALGFADVNLRAFMRVLSTSWSYFALLQTLYGSD*
>SsubOR12

MEFLKKFEDPDYPLLGPCLKGLKIMTMWQKDRLALYSFSNYIIHIIGTLFVVSQYIELWLIR
DNLELALRNLSVTMLSTICVVKSISFILWGKYWNALIKYVTGLEKQQTSQKDTMTASVIG
DYTKYSRNVTYLYCCLVIGTAVTVVLTPLFTYLSSSNEHRYLIINGTAPYPEILSSWLPFDRT
RGWGYWFSIIEHSAICAYGGGVVAVFDSIAVVLMSFFNGQLRLLRKNCARLFGEGNEVVS
YDEAVKRITECHRHHVSLVKYTSVLNSLLSPVMFLYVVICSLMICASAIQLTQEGTGAMQR
IWIAEYLVALIAQLFLYCWHSNEVLHMSNKVEEGVYASMWWSQNVRIRRSVLLLGGQLR
NPIMFTAGPFTVLTVPTFVAILKGSYSYYTLLSKKEN*
>SsubOR13
NLLKISMDLQSFIKHPKDQLFYKTLAFVMGLLALNSKHWQWFEMPKNLLYRVQRMITLY
APPFAWLSQVVYLVLNSSKHEFQSLSMMYSIMTVTFVVMVRVCIAQTKNYKTAMDDFMT
NIHLYNYYITMGRDQYAKKVLIATEKLVRWSYIYLLFFCYTCYTGFEIVPIIHNIQNIESVNN
ITTRLEIIVHMWLPFDYEFNFTNWIIVQVVMVYCGFIAISVVAMFDTLNLTFIYHLIGHIKIF
KHRIKTLFNDDMTQDEIKRNLIDISRHYTFITLTLRNIENAFGLNVANIYLHNLVGGGLFIYQ
LSTSGTEIGTIFTYGLSIPLYMGILVIISFALENICTTADDIPDLVYGLNWDRMSISNQKKVLM
VLCQVQPALIFTAAFNIQTGMQPAVSIIKATFSFYVMIKSRV*
>SsubOR14
MSPKQIDCFKVNTKFWKFLGMWPGENSSSYYKYYSLIFVLIVFVIYNVLFTINFYFLPRQL
DLFIEELLFYFTELAILSKALTFVFMPDKIEDIFDALECEMFQPKTTDGLKIVQDAKKFITRY
YKILAVVSYTSNITHVMSPLIAHILLPVGLDLPICSYLFIPENIKKHIIYPLYFYQSFGMHFQM
LVNVNTDSFLLGLMILTIAQLEVLDYNLRRITEDVESIDNSLEREKALKLRLQKCILHFSEIH
KYE*
>SsubOROR15
GGGTCNYNRTVNMVFLQVTSKQVGPLDTPYLKLMMNTLIFLGFWPGQVTGKKRWLTRN
TKIMFIRYLSFTILSIWYIKNNLRKLSFLDMGPNYINTLLLFVCVSRVPIPGTSTYKVMTKNF
FNRIHLFYYKDQSDFSMKTYREVEKYSSFFCKLLHLEVSAGVFLFNLTPLYKSYRNGMFN
EIKPANATYEHSVYFAMPFDYIHNFKGYMAIFFLNWFNSVDVAFIAGIVDLYVYVIIFNVW
GHLKILKENLRTIPRPQTLNNGINMPLSWYNEEESRNIRNLLIESIEHYKTIKE*
>SsubOR16
MDETFTTFHRVLSVVGISIFAKEKWQSKRWLLLQIFNFVVGILCFIFTTGFVTNNSSNLLLFI
QGACIWTTGAIMTISLGICLYFRKDFKEFLTEMAFKDHMLEMPLIKYVLKMERGQRLMEL
KEMVEESQEKLLKYTRILLKTYVASVFLCATLYLCTPIYEMVQKQDDSLRLLAFDMWFPW
SLENYGVYIASFILHAYCGYLCCIAYPGLQSVIILLVGQIIRQLRILTFILLHLNELVLEITKNE
ERIWQPCCTTVLTQCIDHFIKIKRFSNKLNIICRPFYLTLILVAIMLVCMCSVKIAISSKLSPDT
MKYYVHEFCFILVVLMFCLLGQQVDNECARLQNAVTEKWYIYDRRHKKDILIFSMALSQ
RMPIYIFGTISLSLPTFTWFLKTGMSFFTLVMSVLEEQ*
>SsubOR17
MKLISAKQFLKHTQTPGARHEIDEIFASVLIFHQIFGTQVLDIDWSWRKIRLKLLCSILLLFY
VFVGTIDFMQSTDDFLLFSEAIFTLIIIHQFIFQLLPFVFYRSTFRKVYFLAKTSLFPVIKEISP
ERSVRFLALLENVIKMLFFSIVTPTLLYVLPALWSYFYGERCNLSPSTSILMPMTSPTFEIGM
FLHTIFFIMVAGGTMTVSMWFVVLTLFLTEACDSAVTVLKTKHDKEDSYAKSLNKTLAKF
YSIHVKLMKYLNNLNAMYKWSLLVPMISVYTLTCVFLLVMTEEINCKLAIVVFLEFVCISA
LNMLGELTKTKTEQIKMAFLNFDWMHMRLKDRKNYFIIMCYLNKNFGVRTAIGVELSLLT
LSSIYKGSYQAYAVFNSMN*
>SsubOR18

MIKQFLESLEDPDRPLMGPNYWILKRVGLLLPTNKLGKFFFIMIHEIATYFVISQYMELYVI
RSNLDLVLTNLKISMLSVVCVFKANLFVFWQKKWKEVFEYVDEADKFERENCDETKAKII
NSYTKYCRGITCFYWILVFTTFLTTTGQPLMRYLTSPTYRQNYHNGTEEFPHIFSSHMPFDK
NHAPGCWITVVWHVLICAYGATVVASYDSSVVVIMVFFGGKLDLLRQRCKEMLGVNGV
GVSDEEAAAVIRELHKIHVMLIKHSRLFNSLNSPVMFFYIVMCSLMLCASAYQLTSATNAA
QKLLMAEYLIFGIAQLFVFCWHSNDVLLKNAKVTLGPYESQWWTASLKQRKDVVLLSG
QLRISNIFSAGPFADLTLATFITILKGAYSYYTLLRK*

>SsubOR19

MELIKKIWRKTTRTKALDDASGDMEIVFFEDVYRAAYVAGLSTTDRTVPYLIYSSLVKVMI
STVVCGEIWYTVSQASSLDEIAACVNVIVIQLNTLFKFKNMITNKELYLKLAKSMENSYFD
TTTPKRKELVKYWATTHAKYVKMLLALGNCTLVAWHLYPLVDEIDYNLMLSIRLPFRFNT
PLCYLFTYVIVGIAFTYTALGVMVTDLVMQAHLLPLICQLSVLADCFENIIQDCSATIGNPR
GDSTLNNDFIRLYLKRLGDLVEQHKLILNYSKTLKAILSFPMLGQLAASGTLMCFIGFQAA
TTILDNIVKCLMSLLYLGYNMFGLYIICRWCEELTNQSQNVGEAVYCSGWQSGMVMVSG
VRSTILMVIARANKPVVFTAGGMYNLSLQSFSTLVKASYSALTVLLRFRHG*

>SsubOR20

MELDFDNIYKIPVKAMIYSRSHPSIKRDRKWLLQFISYYIPTSFVFLVAVYDTFFLESNDFNA
ICVNGVLAIIHVVISFEYIIMLWKQNVLMGLMTLMKKDYEKAKYLTVCEKEIMNKYANRA
AYFCKQWLRISICCYCIFPIKSVVLQTYFYMIGEPKLVLVFDLELPGVIGENKENIFVHLTMY
VLMVIYGLYAMCVYVAFVPWGPIFLLHACGQLEIVEKKIEVLFKGDKEVEKKLKDIIKQIQ
YVYSFVEQISLAFRVNYELTLKASTIGVPITCFQITQEFQKGVVSLEFIAITIGCILISSAPCIYS
DILMEKGEDVRIAMYSSGWESVYNGSVRKTIGIVLMRALKPVAIKTPFRTMCLEGLTDLFQ
QSYAIFNMINATWDK*

>SsubOR21

VHNYLQSGELSYVLSFQVLLPFEVKSWFVWAIVYFHSVASAYICTFYFATVDVLYCLLTTHI
CANFTSISNQVKQADKTNVSMFKSIIKDHQYILNLAEDLEDIFTTPNLFNVFVGSIMICALG
FNLAMGDATQIPGCILFLVSVLLQILMMSVFGENIITESTKVGDAAYTCKWYEMDEKTKK
VVLTLMIRSKKPSILTAYKYCVINYESFCTILSTSWSYFTLLRTVYTPPEP*

>SsubOR22

MGFLHRLWNKLTYTKALENSSGKLEILFFESVYRVVYLKGLSSSDSNWPYLLYSFFVKLMI
ALLICGELYFFTESHSMDEIVASVNASLIQFLTIYRYKNMMDHKDIYRQLATSMESPHFDTT
TPKRKQLVEAYAKENEKYLKLLLALGNCALAAWIIYPLVDEIDYNLVVGVRLPFNYNTPV
LYPIAYFVVALAFVQIAHFVMVTDVIMQAHLIHLLCQFAVLADCFENTLVDCQEGFEGIHK
YYLINNKNFAEKYLERLGNLVEQHKLILSHAMNLRNTLSRPMLGQLASSSTLICFIGYQIIT
TITDNYAQGIMSLFYLGYNGFELYIICRWCEEITIKSQKIGEAIYFSSWESGVTKLPGVGSTV
GLVIARARKPLVFSAGGMYQLSLSSFSSLVKTSYSALTVLLRFRQ*

>SsubOR23

HFEEFNMILIERLKLAYYKIKNRLRESTIDIILSIVNIIPNLAGFNIRGKKMAAPFWILHLSLL
LYVYGVGNLVYQLTTADGAEDFIKSYVNVSLLTLIFNMSWWWLTKRSLLLETLQMVEESD
VQARNIDSLREKHKKMLYRIKVIILIFYSTNYTNAALVYLPNRTNVDNEYCMALCVGLGPL
TQTPNRQICSAILFIQECTIMAVVLNYQALLLFLISHTSTMYHLLSEELMSFNIYTNLEENQT
LVRQRLPIIISRHTAILSTCNKLRSLYSLPIGVDFGSNAICMSLFFYLPLQEWLKYMPILVYCF
LSFFLYCFLCQRLTNASEAFERAVYSCGWENFRIKEKKLVYVMLRQAQQPVILLAADIIPV
NMYTFATTLQAMFKFVTVVKF*

>SsubOR24
MSPFWKKVFPKKTKNKPADGDYTCRRSDFDATFFIPRKILRLITIKLTHDITPFASKCWSWY
YWFEFANVFMAMILEILSMIQTASGGSFEDAVKIFRMMPCFGMVVLGLLKSFSMVRHRFV
YENVVRELGDMWHGGRVSEGEWTIIESAKKQLLFVMKGYYWCNNALLVSFLSPPLFEAC
KRMLGYENEMLLQFFYWLPFDPLQPVYYDVLLVLQTWHALIVIWSNLSGDLLFFAFLSHI
TAQFDVLSLRIQSFINVPVDNQLIEEYPLSKLCVKHSLNEHYHLDTDWEDKLQKDLGEIIL
RHKTLIRLSKDVEDLFTFALFVNFINSSIIICFCGFCCVVVEKWNDFAYKSFLLTALSQTFLIC
WYGQKLLDSSMGISHAIYHSGWYKAPRKVKSSLIIMLHRSQKQVRVTTYGFSVVSLESYT
KIIKSAWSYFTLLLNTYKQ*
>SsubOR25
MGFLIKNVNRSVSVSLTALKLVGFWAPQNLGKTERIVYSCYAAFSFMFLLGIYLIIQVVDLF
LIWGDLPLMTGAAFLLFTNLAQAAKIFNIILRRKVVMSIIDGANEALMGVTNEEAKEIVKS
CDKETSMQQILYFGLTSVTALGWACSAEKNQLPLRAWYPYDTSQTPAYQLTYVHQVLALF
VAAYLNVAKDSLVSALVAQCRCRLRLLGHALRTLAADLEAPRQQQFTAAQEQVISGRLRS
CVLQHQSALEAVLQLQDCFSIPTFAQFTVSLIIICVTAFQLVSQTGNIVRLLSMGTYLLNMC
FQVFIYCYQGNQLSEESSEIAGAAYECPWYLCSVRLRRALLVVMVRSRRVARLTAGGFTTL
SLASFMAIIKASYSLFTLLQQVEEKK*
>SsubOR26
LFRSDILDRFRITRDFFELNIKYLLLVGLWPDENWSKVQKMCYKIYQNTLHCFSIIFLVITGV
KTYQIRHDVLIFLANLDKSIVAYNYFFKVILFLLRREEFKKLTDDIIWSGDRVMEKNRNRMI
NHLVVVTVFNSGLVLIFSLVGLYNGEMSIEAWMPFDAKKNKMNLALSAQILAVLFCIPFSY
RAIAMQGIVCSILIYLCDQLESLQEAVKGFEFSKADERESRQSFKKIVKKHVRLMRYSRAM
ARMFSEYFLIQNLAVTMELCLNAMMITMVGTSQKSMLAGFLAFLGLVLLNAYIYCKLGN
ELIDQSQGMAFAAYSSAWTSWPLDLQKDILIIIRASQRPFQMSAGGMAAISMPTFGAALYN
GYSIFAVLNDVVEN*
>SsubOR27
MKTILKNSIWPLTLSLNLLKLFGFLVPGKHSSRQKVILKLYGVFWYIYINGIYIIIQAGDLFQ
VWGDLSLMVSTSFLLFTNVAFGMKLINLMLRGQAVQDIIDDGEGELSSEERDEGVQIVRSC
NRETSQHLYLYVFLSAVTGIGWVASTEKNSLPLRAWYPYDTSTSPAYQLTLFHQISALYCG
ASLNVSLDTLVTSLISICRCRLRLLNLSLVNLCDGLLKEVNSGLETQEKIPLIRLKSCVCRHQ
AVLKSANEIQRCFSEPILAQFTVSMVIICVTAYQLAVELRNTPKNVVSIIGMTAYLLCMILQV
FLYCYQGNQLMIESTETASAAYTCPWYLRSLRFRRALLVIMIRSKRDARLTAGGLTTLSLTC
FTAIVKASYTFFTVLQRIEDIEK*
>SsubOR28
MKAKVRTTINSEKIHNYMHYIELPYKMAACWDWYPNSQKESHLIINNVYLCLVLFVISKV
AFGLAVYLYTEWLDIMTSLDQIADSLPLVVSLAIVVYYAIYREDLYQLMTYMNVNFKYHS
ARGLTNMTMQQSYDAAKKFARIYTACTMFSVTLYAVLPFILNSDPLQNWIYMDITRSPFIQ
LTFLQTLLSQWFVGLAMGQFGVFFAANSVLICGQLDILCCSLRNVRYTAMLRNGVHYATL
EASQPDIQLDERHSYVYNTSEMTDSSYHYDKKMVNNFTSTGIKFDMYDSAYDEATVSAL
RDCAQLCQVIYKYKDMFELFVSPLMVLRVVQVTMYLCTLLYAATLKFDMVTVEYLAAVA
LDIFVYCYYGNQIILQAERVSTAAYQSAWHTTGVRPRRVLVNILLATRRPVVVRAGGFLPM
DLHTFVVIIKTSFSYYTLLVNVNEK*
>SsubOR29
MWNKVQTFGPKYCDLPTMISNINALLRVLTLNVDSRVTVPIPTASYLGMSFCTVCYYYAY

QFSMLWFVFCKMNQSGALRAMLVFGSLSVSSHVAMIKMWYMFVYRSKVRCLVDQCLE
WNALTVPGTRFSKNMSAALRIVKRRAILFWICVNTIAVFYTTTPAFLPGTHLPEDLLVFYGL
EPTFETPYYQLSYVIMLVCVIFICYIVTTITAFVIVILGYIEAQMMSLSEEILCIWDDSVQDST
DFQINIISIFPKNQEIFDDNIKRKINSRVRKRLVDIMQRHSQTITLLKNVNTFFNIPNALMFVF
LGISLIAELLGGLENTYLQIPFTFLQVAIDCFIGQKIMDTSVTFEKSVYACKWENFDKDNMK
IVLLLLQNSQRTLTVTAGGMVVMNFSCMMAIVKFVYSSYTTLRSTLKS*
>SsubOR30
MDYVRTIKNFVSKTDYDFGRPDITLANFHPQLYITLATQGIFFGQRDSYWRFCVPCFCTSLS
IVGLTLQCMVLYMGVTTEDYSAACEAFCYLVLIVSTITIYAGFLLNKNRILSQTEEMAQDF
GEICNLGPRYRESFMEGQLLVWKLFWGWSLFVLSICNSFLLYPCLLLMHQSLVATQDENT
KRPLVFPVWLPNDDPHRTPNYEMLFTIEVWYCLIFDVCFGGYVYTLFHFLLHYYTKMDLII
LDFKVIFDGLDETVADLPRDDPRRIAVQMVLNKRLKRIVTWHLSVFRAVKTVSSVFRPSLV
YQLMLSPVAICLIAYLVAQNLERGEIDIPFTMLGAGAVLQLWIPCYLGTLLRNKGFAVGDA
CWNCGWQETPLGRMIRTDIVMIIKRSQLPIFIKYTGQPVIQLETFSAVMSTSYSYFNMLRQS
AK*
>SsubOR31
SCNMADEIYLRNPSQQLFYRRVAWVMGTLGFNSHYWSWFEYPRSKIYRLQGKAVIIFLPVI
IISMILYLCMYYKDYEFIILASMFSVLPFTMCLMCKVWSAQTSSYKAAHLDFMDKIHLYSF
FTQNFPANKH
>SsubOR32
MEVLKDYPKEFAKLFRPSFNYLFRINMNFLAKDEGLLRKYWRYSYIIPCLLMINVTLGWN
LAKMAARGKIFEIAYLLPPCLVSIQATLKAMVLIPKTSFLSNIIIELRTLWRITGLNENQKSLR
NSLLKKVNFCNGVTYWVSIIGTTQYLITPLVETLVRRLVLNQDCDLLLPLECSYPFKVTEN
WFVYLAVYGFQAYSMYLCVFAYTGSELIMITVCALVGVEFTLLCEDLRNIKLCKKRSSLNE
ILEDDEANTEGLSIKDIVMRHQRLCVLSKHLNEAFNQMIFVDLMFVGLTTCFFRYAAQFSR
GPTYMINNYVAVVSSLLYVLYLCYYGELLMRASSRIGDVAYHTLWYEGDNRHQKIIMFIIM
RSQKPCCLTSMKYAPVTLNLFTKVMSSTWSYFSLMNTVYGEN*
>SsubOR33
MTSTRQSDIFKYNFVFWKILGLWSGRKPNKNYKYYSCAYIVIFLMIYNILLTLNLAFTPREI
EYLIREVIFYFTEIAVTSKVFMIIIMRDKILEAFKLIDGKCLDGDDEISKEIIETTNAGYKTYR
KYFAIISHAAYSSQVVVPIILHFIFHTKLDLPICQYYFLTDELRNRYFIFWFIYQSWGMYGH
MVYNVTIDSFIAGLLLNAVIQLRILNAKLSNLRLSDQEKKLSIEIQEKIQMKRLRVCLVRYD
LVLKYCETIQSIMNVTMFVIFGMSSLIICVVLCGLILPSSTEFFVFMVGYLFAMTLQIFVPGY
LGTLLNYESRELVFAAYCTEWIPRSESFKRSIRVFVGRANRPIQILGLKMFPLSLATFTSIVKS
AYSFFTLITSFQE*
>SsubOR34
MPDSIRRPRRYFGFHYILLRCTGLGWWHHPDENDTRNFPGWYLYYSIATQIIWVAGFVGL
ETIDPFVGEKELDSFMFSLSFVITHNLTIIKLYLFFFRNKEIQEIVRTIEIELHDYYQNENTNR
KTIKICKTFTYAFIFFGWLTIGNSNTYGAIQDFHWKALLKRTNDTSQIPPRTLAQPIYIPWDY
QKDQSYIPTFILELVGLLWTGHIVMGIDTFIGSVILHMSSQFVILQEAIKTAYDRTMTKLLEG
AVRNADENLPSDSADISLMNLEENNERIVRAFYTVEQIESALQTTLVNCIRQHQVLVSCVE
KFSITYSYGFMTQLLSSMAAICVVMVQVSQDASSFKSLRLITSLAFFAAMIIQLAIQCFTGN
ELTLQAGRVSGAVMECKWELMPPRLRSLMLVLMMRAQRPIRLSAAGFSHMDNDCFLAIM
KAAYSYYAVLSQKQV*

>SsubOR35
MDEPLLIDQSIKKLEILYRFAGINIRSGEQKSRCLKSRMIYIFNFLWLFVDYVGSIVWLVDAF
RTGRDFLELTELTPCLSLSTLGNFKGLFQVMNEKHAHKIIDNLRDMEKQGRNRSQSSMKD
EISQKNKKFLEVVCNLLNGLFGSVMTGFNLTCLLIIAVKYYQTKELELILPILILYPFDPYQL
KVWPFVFLHQVWSEYVVIINIRAADSFFFACCIYISIQFQLLRLEFEDIIPDDGNCNEKVMD
SKEFKNKLSDLIKWHQKLIESADLLEIMYSKSTLLNFLSSSLIICLTGFNIATMTNDTMFTLK
FASFLFTGLLQVFFLCYFADLLMVSSIGISDAVYNSRWYMAEAKIGKKLLLVLTRAQRPCK
LTACGFADVNLNSFMRVLSSSWSYFALLQTMYAE*
>SsubOR36
MNDNTLFDITLERLVKLFRFAGIYITRANEERTTYNSFKFRLYYILHFFANNYNLIASIIWLIV
GLKNGQDFLECTYTIPCLTINILAQFKGILLIKHEEKIEKIIAKLKNLNSLEKQRKQTSERDEI
VKSEEKFLNIVMKTLSIFYAILITGFSLSPLILIVLTYVETKEVQLVLPFFGIYGYDPLQSMIW
PFIYIHQCWSEVAVVSTTCASDYFFFTCCTYIRNQFRLLQHDFEYIIPEDKNTKQFVEDETFK
KHFNELVKWHHDMIELVDILEIIYSKSTLLNFFSSSLIICLTGFNVTIVHDVANMVIFLSFLFV
SLVQVFLLCFFADLLMKSSMELGDGVYNSKFYLAETKISRKLLFVIQRSQKPCKITASGYT
DVNLNSFMRVLSTSWSYFALLQTMYGN*
>SsubOR37
MLKQILSKLENPEQPLLGPNIKALKFWGLLLPDNKLGKIFYLFMHIAVTIFTATEYVDVWF
VKYDLNLLLNNLKITMLATMSVCKVSTFLRWQERWKSIINYVTRADLYQRNTKDGDKIA
LITQSTRYCRKITYLYWSLMYTTVLIVMTQPVYKYFSSAGYRENVKNGTGTYIEVVSSWV
PFSKTSLPGYLAASVYQSYAAIYGGGWITSFDTNAVVIMVFFKTELELLRIDSAGIFGTVAS
PVTDRVALKKLKDCHRRHVELVKFARIFDDCLSPIMLLYMFVCSVMLCVTAYQITIETSPM
QRFLTTEYLVFGVAQLFIYCWHGNDVLYASQNLIRGPYESYWYLRQDSRKNLFILIAQYKK
RIVFSAGPFAQLTVETFINILKGAYSYYTLLSQSQMQ*
>SsubOR38
MGAARKYQYTPNETTKIFQKLNTVLYLCGLTDFWIEDVKLPAGFVKLYNFLSIFIELLMFSF
MMLEVGAFFTQSNLSDKQAADRLVFGTSHPILYIYCLSLIYYKNDAREVLYQLAVVLKDIY
NDGKVEKQMLSKLKFYLMAYVTSCGLCMVFYGASALIQYIRLGSPFTTVITAWPDVTDES
TIAHVSRVSFYIIWWIYFTRVSASYILILSMFIFLSFQYRNLQSYFSNLNKVFDMPMEQFDM
ETQFLLGFKTGVKMHSDTLWCKQQCQKIYSIIFSAQLLQTVAELVSMMAQMMNSERTVV
NLAAVLSTTCGSLISTGFLMWNAGDVTVEASDLPSAIYSSGWQNVHRSSVQARQLATLAI
TQAQKPVVFMALGFVPISYETYLTIVKSSYSVFSVVY*
>SsubOR39
MSEAGSCIRAHVAVLRSCGFLRAAAPNHAHAAYRALVLAVTTLYLAQECAYAYQERADM
EAVARVMFLLLCHVTSLVKQFVFHTDAARIDALITSLDGPRFSAQSDAARILLEQRARGAT
RFVRAYAGTAVVTCTMWAVFPALLRARGDQVLFPFWVGVEYQRAPRFAAVLLYSYYVTT
LVGIANTTMDAFIATILSQCKTQLTILRMNFEDLPERALAVAGTAAGEGEARERYERALDA
LFRDCVEHYHQICQTAKLLQDIFGSAILVQFGIGGWILCMAAYKIVSLEVASVEFASMLLFL
CCILTELFLYCYWGNEVTEESALVATSVYSMNWTRASVRFRRALLVVLVRARRPLQPAAG
RVVPLSLNTFLKIIKSSYTFYAVLRQTK*
>SsubOR40
MEIFIENHNSVLWLCLNILRWGGFLVPDELTGFTKKVFPFYSLLWIMFIFGKRQQRWTGHT
RIVLSQVVALIQGWGDVREMTSISFLLFTMSLFAAKVVNMKLRKETIQTIIDEGEYELRNE
NRVEGKEIIKDMNKEAICLLLMYSVAVSLTLLGWVVSTEGNRLPFQAWYPYDTSTTPAFQL

TFVFQIAGVVLVSLMHVSMDQVVTTSAAVCRCRLQLLNLSLRTLCNNIPITDKLITTEEEEL
VKSRLRRCVRQHLGVLQSAAQIQECFSLTILAQLLVSIVIICATAYQLAEESNNNTIWVMSM
ASYTVAMIFQVYFYCSEAHKLSEESAVVADAVYECPWYACSVPLRRSLLVVMSRSRNIIVL
TASGFSTLSLNCFVSIIKASYTFYTVLKQMEN*
>SsubOR41
MESNRNDFPNKTTKFFYKLCLFTYYTGMPTFWMKPLPVQSTKINIPFRIYSYITTALLYVFV
LLEITSIFTQENLTPKQKSNQMVFIMSHPMLWSYCVSFSRHADNVRKILVDILKLKLVYND
LEVEKKMMKKAKFFSLVFSGMCVTCVLFYMLDVFIQVIKKEGTFTTVITAWPLVDDTSTS
ANVLRVVWNMIWWTLVLRVLSVYAFVFSLASSLSHQFLDLQSYFLSLNQIFENNQWNQR
HKELNYEAAYKTGIKLHSDTLNCVELTKSICSWLFSGQIIFNVIMLVILLAQFLVRNKNQ*
>SsubOR42
MDVDSCSTFDTLRPHFDALARSGYFKIVLRNCSPTKLALHHAYRIVIWTLVVSYNLQHLIR
VVQVRHSTDQLVDTLFILLTTLNTFGKQATFNIRGVRVDNVICITNGPLFSATNRYHVELIK
RETMLMSRLLKLYHGAAFFCGVLWSAYPIINRAVGQPDDFTGYIPFNTTTTVTFALAHAY
MSIVITLQAYGNVTMDCTIVAFYSQAKTQIQMLRHNFEQLVDGVKINGRLIERHNKQNVY
KDNDDDRILLQRRFVRCVEHYKQIIGYVKEVESIFGEAMVVQFLVMAWVLCMTVYKIVG
LSIHSAEFVPMAMYLACMLAQLFIYCYFGTQLKCESELINQAVYSSDWLSLSPRFRRQLIL
MMQVCSRPLAPRIAYVVPMSLETYIAVLKSSYTLFTFLDRN*
>SsubOR43
MKLFIDNSINVLWLCLNLLRWGGFLVPDELTPLQKKIHPFYFSFWITFVLSFMASQITAMIK
GWGDVDEMTAISFILFTNFTFSVKTINVKLQKKAIQAIIDEGDYEIKNENREEGKRIIESMN
KETARLLMLYTVNVSISLVGWLAGTGKRELPFHAWYPYDTTKTPAFQLTFALQMAIMSFV
ALMHVSMDQVMTTSTAVCRTRLQLVNLSLRTLCHDITITNKLLTPDSEEVVQSRLLRCVLQ
HLAALQSAAQLQHCFTTTILAQFTVSVIIICVTAYQLAAASSHSIIWLISTTSYLVGTVWQVF
FYCNEAHKLSEESTLVADAAYDSPWYAWPAPLRRTLLVMMVRSRRPAEISAGGFATLSLTC
FVSIVKSSYSFFTVLKQTEE*
>SsubOR44
MEIQTDPKPEKKYKGFEETYPLCAYSLAFGFLYPNRTNVVKRMIIVTLIIALNGAQLFWFIS
YTLKCLFILDIYNFARNITLGIITLLFFIKTYYAIYASYKFAPLLDSMSRDYLKANDLDDDYR
EIYDKYIRMSKVGEIAWLCIPTALSAQFPIYAGICMSVESMQTDDYTRRMVHHMDLKYVE
HIQSESPFFQCMFAYNCVQCLVMVPNYTGVDGSFCIATTHLRMKLKLMTLKVKRAFTDAR
NLKELKELLKNAIRDHQLALDFHAQLQDMYGTWLFTIFLLTSFMISFNLYQLYLLGHIDVT
YTIFLMVGVLHIYMPCYYSSALTKVGDEVSSDLYCVPWEIAADKSITKTLISMTVRSQKDL
LLTGNGLVIFNMELFKTILQTSYSFFTLISA*
>SsubOR45
MALQKLDSLTEKLEDPDHPLLGPTIKGLYLFGLWQTGSKLWTVAYNIAHFWTIIFVCTQFID
IYTVRHDFNKVLNNLSISVVTIVCVVKAFSYILQQKDWRALVSEISKEEIKQLNILDPAITK
QMNAYKKYTRIVTYLYWMLVFNTNLLMIMTPLLKYASSKTYRDEIRNEVEPLPQIVCSWF
PFDNTKMPGYICGVCFHVIMGNLGCGVLAVYDMNAVAIMCYLKGQMSILKEECRNIFEET
VSGDVALDKIRECHRHHTALVKQYKMFNSLLSPTMFSYVLVCSSSICCSVIQLNMTEATKS
QKFWVIQYSAALILQLFLFCWHGNEVAIETKQVDRGVYESDWWKGDRKLRRLTMLLAA
KLNIPLILRAGPFTALSIPAFLNILKGSYSFYTLFSQMQTEK*
>SsubOR46
MMAKPKTVGLVSDLMPNIKLMQLSGHFLFNYYNESASMSTLLRKIYAGVHAFLISVHFVL

MGINMAQYSDEVNELTANTITVLFFAHTIIKLVFFAFNSKSFYRTLAVWNQSNSHPLFTESD
ARYHQLSLSKMRKLLYMLCFMTAASVVSWVTLTFFGESVRLITSKETNETLTEPAPRLPLK
AWYPFDAMSGSMYVFAFVFQIYWLIFSMAIANLMDVMFCSWLIFACEQLQHLKAIMKPL
MELSASLDTYRPNTAELFRVSSTDKSEKIPDTTDADIRGIYSTQQDFGMTLRGAGGRLQNF
ATNPSNPNGLTQKQEMLARSAIKYWVERHKHVVRLVASIGDTYGTALLFHMLVSTITLTLL
AYQATKINGINVYAFSTIGYLSYTLGQVFHFCIFGNRLIEESSSVMEAAYSCQWYDGSEEA
KTFVQIVCQQCQKAMSISGAKFFTVSLDLFASVLGAVVTYFMVLVQLK*
>SsubOR47
MRLQKMLESVSKFGLRYYDFPTMLSNITFFLRTLSLNVDPRNKTRIPIVYTVLVIAFATSYF
YVYVVSMIWFVFWRCMETGDVLSALIIFSLGTASQIGPIKIIFMFYHRTKLTHLVGQCLDSC
ALTESGSRLSMNMMKSLRFVKRRAIIFWCVIMGNGFLYILKPIVLPGRHLMEDLFVLLGLE
PMFETPNYQIAFILTAMGICVTVYFPSNATACQIVISGYIEAQMLAWSEELINLWGDAQKFY
SKYNTVPTDDTDNFTNSKVITVNKFVNKRLKQIIEAHATNLSLLRKTEDVFKGAIAVEFSL
LMVGLIAELLAGLQNTYMEVPFALMQVGMDCFIGQRITDASKMFERAVYHCNWENFKD
NMKLVVSMLMTSQRTMTLSAGGITKLNLVSLLAIIKMIYSAYTTLSATMS*
>SsubOR48
KVERDLAHVGHLGAMGLSAFCIILASGLFMCIAGDVTVEASQLCTAMYSSGWHYCQGSC
SVRVRKLLMISMIYARRLVVIKGLGLMSLSYQSYVSLVKSTYSVFSLVLQQTK*
>SsubOR49
FNITTKLELGLYLWQPFDNEYNIYGYIVSQFIVIYCCIVGTLIWVTFDSINLAYIFHLTGHIHL
FKCDLEMWQNVKFSDDEWERNLKDLTITYTFITKTFKNAENAYGMNVVSIYGQNLAQGG
LLLYLIMTSGSDMGVLLTYGLMIPIYMGTLVFLSVTLENVRIEADDIPNLVYALPWESMSV
RNQKLVLAILFQVQPTLEYIAAFNVKAGVQPAIAIMKTTFSYYVMIKSSKEM*
>SsubOR50
MFEQFKEKLAFLSPILPYGVIEPWDDLNPKLYHAIHIYWLKFYGMWYNSYSPKSFMYWFQ
QLYTIIVLWLVCFLPGIGEVVYLLRRRDNIGDVAEGLYLFLSEMYTYVKVAVFWYNRNKVI
AMLSYLHCPEFKPKEKEHENLYRKSINTARFVMSNYSAMCVGAVSVGMIIPLTENFDILPT
NVEYPHINVYNSPVYGILYIHHVYYKPATCIIDGAMDTILAAFIASAIGQIEILAFNLRNFDT
LAERQRNRAIRAMDNEFQSKEFYMKEVLKDCIKHHNCIIRYVSMIESAFSVASALQFMLSV
MVLCLVGIQFLSIENPTSHPMQIVWMAIYLTCMLIEVFILCWFGDELIWKSMDLSLAAYEG
PWLKIDRKTSMIVIIFLERCKRPMKVTAGKIFTLSLDSYTVLINWSYKAFAVMRNMKK*
>SsubOR51
MYSENFINHPKYQLFYKVLAFALGILAMNSECWHWYTMPKHFLYRFQRIFVLIAAPLAPV
SQVIYLFLYSGDHEFQSLSMIYSIVLVALTVTIKIGIAQTKNYKTTMEDFMAKIHLHNYYLT
NQDNYVLKTIIQTEKSVRLSCFYHLVFCYTCYVGFEFVPIVHNFQNNESMKNVTTRLEIIV
HMWLPFDYEFNFTNWLIVQITMLYCGFLSISVVAMFDTVNLTCIYHLIGHINIFKNRIKILFN
AENTEEDIKNNLIEISRYYTFLRQTLNSMEGAFGMNVASIYLHNLVGGGLLIYQLLSSGTNP
GTIFTYGLSIPLYMGILVIISFALENICKTADEIPDLVYCINWERMSISNQKMVLMVLCQVQP
ALKFMAAFNIETGMQPAVSIMKTTFSFYVMIKSFM*
>SsubOR52
MDNHTFTKHPKDQRLYRSLAYMMGLLSLNSLYWQWVKTPKSLFYRIQGMITLYASPCAW
ISQVIYLVLNSNEHEFEALSMMYSIMLVTCVEMLRVFVAQTKHYKTVMEDFMTNIHLYNY
YLSMDRDEYVKKTLIATEKSVRWSYYYLATFCYSCYAGFEIVPIIRNFQNNESMSNITTRLE
IVAHLWLPFDYEYNFNGWLIVQGLLFYCGMIVVSVIAVFDTVNLIFIYHLTGHIKIFKYRIQ

KLFNDDMTQEEIKSNLVDMSKYYTFIIHTLKNTEQAFGLNVATIYLHNLIGGGLFIYQLSTS
GTNAGTLLTYGLMIPLYMGILVIISFALEIMCISADNIPDLVYSLNWDKMSPSNQKIFLMLLS
QVQPSLEFTAAFNIKTGMQPAVSIIKTTFSFYVMIKSQM*
>SsubOR53
MKLKVKDDDYSDYLKHPKEQKFYKYMAFAMGLFALRTETWYWYHRPASKFYTMQQWI
VVIFVPITCFSQVFYLYTHYKEHEFVAIAAMLVIMPIGFVVCIKTLGAQTQNYKIAMKAFM
DNIHLYNYSKNTYSEHKANYVFKKVIQAEKYTRWTFYAFMTVVLICCVAWNTVPLIHNLQ
NNHSIHNITKRLEVCIHFWQPFDYEFNFYAYLYIQVLGIYCGGVAVIIMVLFDTINFSFIYHL
VGHLKIIKFDLKTGFTKEMTDQQIKQKLVDITKYHCFITDTVKVVEAAFGFNIVAVYAQNLI
QGALLFYQMLTLGGEAEVILTYGLMIPIMMGVLILISGVLENVRIQADEIPTLLYALPWESM
SVANQKILLHILFQVQPTLEIKGSFNIKTGVQPAVNVIKTMFSYYVMIKSNRM*
>SsubOR54
SFRNMVVLKKASLVSTITFVVRCAGLWHIERKYKKHPLAVFAMFCYFGSYIFQMIALILFK
NNIELEFEVFSVISFCGMGILKLLSLYLNSELWLGLLARMKDLETMQLKGDENSSIAEYES
DSESDNTKVAKLITNYTTKFKRTATILYKIYGFTGVVFILSPFIEYAFRLFNGIKLEKYPHILPS
WNPFDNIHFLCYLFIVLCEVVAAIYCVCVHIAFDATVIGVMIFICGQFSLLVYQSERIGGRG
KMCSVSKKRDLRAHSRIVTSHKVHIQLIRCTNELDTLLKNILGFYFFLATLTLCTVAMRLKS
EQMGAMQLVSLLQYMCATLTQLFIYCSYGD
>SsubOR55
MAAEPETSAIISQKDRYYLQYPAEHTFYKVIGFIMGMFGLNSEYWGWFKRPIGRVPYIAY
MIQRYIVIIFVPITSFSQVFYLYMHYSEHEFAALAVMFSIMPVAFVVAVKSAAAQTPSYREA
NTAFMQKIHLYNYMNDTNFSEHKRHHIAQELVKVEKYTRRTCIMMMASVLTCFLAWNIV
PLVHNIQNTDNIHNVTTRLEVCVHFWMPFDYEFNFYTYLFIQILGFYCGGVAVILMVLFDT
VNFAFIYHLVGHIQIIKYDLKYGFRDVKMSDLELKTKLVKLTHYYCFITNTFEMVTAAFGL
NVASIYAQNLVQGGLLLYQIMTTGRNIETLLTFGLMIPIFVGILLLVSLILENVRIQAEDIPAM
VYDLPWESMSVSNQKILLMILNQVQPTLEFVAAFNIKTGVQPAINIIKTTFSYYVMIKSSKE
LEE*
>SsubOR56
SDTIGRVQVKMQRFIKQLSFLEDPRHPCLGPHLRCLTFLGIWHPIMSKLTRFKRAFIYFTIFF
LASQYVKCMITLNVDSLKLILQYVPFHMGFVKTCFFQKDYKGWERLIEFLSKKELHQISK
KDEKQDAIISDYINRSRKVTYFFFALAFFSNFSIFTEPYLKNQISENGTSVYVYIFDGFTPFAR
EPPGYYFSMLIQTIVGHMMSACVVSWDMLVVSIMIFFAGQLRMSRLYCKRVVDLKSIQRT
KRNIAECHQFHINLIKYHKEFNDLISPVMFIYLIVICTSLGVCIIQIAEIEDDIPTLVAGCLYIG
ACLVQLLLFYWHANEVTVESELVRYSTFKSNWVEADRSVQKQVALLSLSTRKRLTFKAGP
FNEMSLSTFIGILRSSYSFYTLLKGTN*
>SsubOR57
MESLPEEYVEPLLPCFGLLKNCNVRFFDDRALFWRVLPYFYMLPSVVAYHIALTIYLIEGC
KRSMDLDELVYVVPVYVVCVQSIFKAAIVIPKKKDIKSIILRLGNLWRTQELSEVQISKKNV
LLKRLKFCYAAFYWMNIIGSWQYILAPILETVVRIFILGQDFEWLFPFGCYLPFDPRSNWM
VYLVTYFFEGYSMITIIYFYLGSEFLMIILCSHLSTEFTLLQEDIQHIKPGKQSISHFNESPGYS
SDIELYVNEKTELTALIRRHQILIGLFQDLNEVFNKMIFVNLSSATISICFFGFSAKVSHDAVS
LINNFIGVLASMLPIFNLCYYAEMLKEASEGIADAAYKNLWYNGNKSYVKMIIFILKRSQK
PCCLTSLKYNPISLATFTTVMSTTWSYFSLASSLYDENT*
>SsubOR58

MTLKNWCFENKFKIPSTAMYLIRTHPFIPRSPVWLMQVISLTAVNACCLVFLCNSIFLHDAK
SGNYADASKNFAMLIICFNSNIKYFTMLNFRKSIAGLIKIIDRDYDLAKNFNEEERHIILNYS
DKANKVCKFWIYVAILTASIFPVKAFYLMTYYLFKGEPQLVPMFDFTFPYFIEKYKPATWA
FCGIFGLCFFFDSYAFFVYLGFEPLIPIFTLHTCGQLELMSLKVRKVIAESENEREMKNKFK
CINEKLQELYSFINVLQKCFMVLMEFNMKTSTFIIPCNAFQIAQEFRVKGVISVEFILLLIASL
IHFLTPCYFSDILMESSEKFRQAIYSCGWENCRYTSIRKTILFMLTRSSKPFSISTVFYFISLDT
FSTMCRQAYAIFNLMNAAWT*
>SsubIR1
MELVKCLMLIILNLGRVIGLNSAVPMIIDVVRYYDRPTAVLLKVCWKMSDKINLATEFMHT
DDARTIQFIEDISSIERFKAEDLVTVLDLNCPDNGNFLAGASKMNKFEKPYRWILHSSEENV
DVVPLEISNLSILVDSEVIISYNVSNNETLLRKVYRIDSASDYIIEKFATWTVENGLDLSAEA
KQTTLTSRRNLRGKRIGISIVSVANSTETELYNLNNFVDDDLAKSSFHNILPLYDFINATSKL
LFTDTWGYYINNSWNGMIGQIMQGDADFCGTIILMRLNRLDILSFLTQPIPITARFVFREPP
LSLQNNLFYLPFRTTVWMCTGGLIVALVFVIYITAKWEVERNGTESDDPTILRPNVSDIAIF
VAGAVVQQGGNSQLKGSLGRVVTFIMFLIFLFLYTSYSANIVALLQSSSDQIRTLSDLLNSK
LELGVEDQPYTRYYFTKATDHVRKAIYENKVALTGYKPNFMSIEEGVKRLQNIDAPFAFH
MNLGIGYKFVEEIFFEHEKCGLREIEYIQNAVPWVCCKKQSPYKEIYKIGLSRIKEHGISDRI
NRLIYTRKPACTSRGGNFGSVNMMDFHPVLLCLLYGMIAAVMLLILEILVHKRKEKLKSK
REKSTRDAKGYDSDGVWNEIADDF*
>SsubIR2
MFFFVFCTNMIIINIIHIVSIFVSVFAYDVQFYPSQSLLDGVSVSNVYKKRNQNENKQDEHN
SGQKWRKFENIADSLPKKNVTKRAVDPAFYGYPKTREELWHEHFLNESAAFDQTPSLIKL
LHNLTLTYLKDCLPVILYDSQVKSKESYLFQNLLKGFPVSLVHGYITDKGQLAEPELLVPV
KECLHYIIFLTDVRVSAKILGKQSESKVVVVARSSQWAVQEFLAGSLSRMFVNLLVIGHSFK
DGDDNSLEAPYILYTHKLYTDGLGASLPVVLTSWSHGKYSRNVNLFPPKMTKGYAGHRFE
VTAANQPPFVFKTIKTDLDGGNPRIIWDGIELRLLKMMAERNNFSIEIKEPQELHLGPGPAV
MKEIVSGRADIGVAGMYQTIERFKEMDLAFSHSQDCAVFVTLMSTALPRYRAILGPFHWH
VWVALTFTYLFGMFPLAFSDKHTLRHLINNSGEVENMFWYVFGTFTNCFTFVGKNSWSK
TDKLTTRLLVGWYWIFTIIITSCYTGSIIAFVTLPIFPETVDTIDQLLDGFYRIGTLDRGGWEK
WFINSSDYKTNKLMKKLELVASVEDGIRNTTKAFFWPHAFLGSKAELEYLVQANFSATKS
KRAMLHISNECFVPFGVSLGFPKNSLYTAKLSSDVRRMFQSGLIDKIVDEVRWEMQRSTT
GKLLAAGSGILKIPSAEEKGLTLDDTQGMFLLLAVGFLLAATALVSEWMGGFTRRCRFLCR
QKKEREQQATSREELVSTTGDDEIKVISDDAESRIHFDTGITSAGSRDTLEGQVINVTAESI
MVHENLDADGWESRRSSSVDLDKEVNEIFERDRIQKGLVSNDKTDVNIEESHTTVSNNPF
NDHHKNDC*
>SsubIR3
MELFSFIATFFVSKKISLLTAFVCWPPDETASLWRKAAEAGLRLKIFTDFSTSLEMPAYDFY
REGVLLDADCLEAEEILQKASASRAFNHRYSWLLLHNSSLDSSTITDLLADINILPDADVI
WSSPESSADVYKVKTGCPIIVTPIDIERTTGLKEMEMTWNRWPTAVSRRKHLNNVSLKAAT
VVTQPQYFKGWTDLTNRQIDTFPKLTYPIMMLLAEDLHFRYSRTSLLCCRVVHRYFKR*TV
AIWLMCHYRYNLRQVDLYGEERNGSFDGVSGLLQREEIEVGVTSMFLRADRMRVAHFSA
ETVELKYVTVID*HKYYKQLGTKLSYINAKCGRGAFLFRQPSQSAVSNVFLLPFARGVWA
ATGGVLLLCASLLALATWRLRATHSDLEQVSLAECFSFAIGTICQQGFHITPGAVPVRVVM
VSALISSLFLFTAYSAKIVALLQTPSSAIQTIEQLTRSPLALGVQETTYKRVYFAESQEPATQR

LYQRKLLPLGDRAYLSVVDGVARLRTGLFAFQVEQSSGYEIISKTFSEFEKCGLQEIQAFKL
PMVAVPIKKHSGMRDLFAARLRWQREAGLMNRWRQRWMAAKPRCDAGAAGAISVGLA
DVLPALQVWAAGAAIAVILLAVEIAVHKLQNRRKKLFQEILSHKMYLN*
>SsubIR4
MELLFLSVLFISLGSVVSEISLRFVFIVEVHERDLSGAIGRALKEVESSSPGVRVDDDIVILD
RENEDQSYRMLCSAISKGVSAIIDVSWSPWEPAGEVPGAALVRSTLGAQQLLSALDQYLA
ERNTTDAALIVPTEADVDKTLYRLLGASDTRVWTHAGLTRDSARALRDMRPEPAAYALVG
SAAFVMDSYARAVKEKLVRRPYRWNLVFTDYPGPNLDLSSLVLPASILYVDEGECCKLAG
GKGECVCPSESRRPQAILTALIQYLVTTFSKLDRDLPLTPKLSCEDPRSDLNTTRILYQQFSE
DSLDNETLFYWDEDRYSLELRSHFHLSMYEPEEGLQPTASWSAAEQFRLLPGVTLQPLRM
FFRIGTSPAVPWTMPKLDPVSGEPVVTEDGQPEYEGYCIDLIARLAELMEFDYEIVTPKTG
GFGRKLANGTWDGVVGDLMRGETDIAVGALTMTAEREEVIDFVPPYFEQTGILIVIRKPTP
KTSLFKFMTVLRTEVWLSIVAALVCTGLMLWLLDKYSPYSATNNPTAYNYPCRKFTLKES
FWFALTSFTPQGGGEAPKALSGRTLVAAYWLFVVLMLATFTANLAAFLTVERMQTPVSSL
EQLARQSRINYTVVEGSTIHQYFINMKFAEDTLYRVWKEITLNATSDQAQYRVWDYPIREQ
YGHILLAINASGPVPDAKTGFEQVNEHTDADFAFIHDSAEIKYEITRNCNLTEVGEVFAEQP
YAVAVQQGSRLQEELSRALLVLQKERILEQLGSKYWNETARQSCPDADESEGITLESLGGV
FIATLFGLGLAMVTLAWEVFYYKRKERNKVQALDNVEKPPAFEGEKKKGLEKISESVARL
RKREKRAKNKLVTIGKNFVPAAEKAGVSYINVYPKTEYRP*
>SsubIR5
LGALYSWYEEFLYLDFSAAGIRSAVTCVAPSPRLLSSWTMPLMPFTWNMWYGVLITFLFA
SVGLAIAQSSLDGIFMTVFGVMIAQSQKNAEVSWRVRSVTGWLLMAGLILVSAYGGGLAS
TFTVPKYEQSIDTIQNLVDRNMEWGATHDAWIFSLTLSNEPLVKQLVSQFKTYSAEELKRK
SFTRSMAFSIEKLPAGYFAIGDYLTQEALVDFTIMLENFYYEQCVVMMRKSSAFTEKMSAL
VGRLHQSGLLLIWETQVALTHLNYKVQQEVKLSRLRGDVGFKPLDLGNVEGVFIFYAIGV
VLSILFFVAEWIMMPRGH*
>SsubIR6
KHCFLNSWVCSLGFCAYLRPRKQSLRMFFVFFNIYCILVVTAYQTKLIDVLKNPTFDYQIK
TIDELVESGMSFGGFEELHDLFFNSSDPFDKYIYDHWVNVDDISMAMIDVTVHRNFSLLCS
RLELAHISAVVPELSDNYGNFRYYAFTMNMFSVPMEMVALRGFGFLDKISEGLALFKQAG
INEAIRKHFRSFNSRRRARLLRSLLWEASEINSLSLQHLQGGFLILALGYISGTLVFALEILLN
TEFIRHKLGSRNPMLTTNRKK*
>SsubIR7
MKQLFLLMMIGAVFAINSDGIKLAKDYFSYKRIEYVCYFSCESQYKNLQVVKELMQGINA
RTSVIQTDRHSDLPMEMFLYQRIASVGVLLDGTCDNSTFVLEQASKAKLFDATHAWLVFM
PDYYSTELVHEQFQNFNLSIDTDLAVVSNNGSDYIITDVFNFGKIQNNSLEIASLGSWRPDD
GLNIYLQQYKYYHRWNFHNLTLRAISVVRRFYTSDGK*
>SsubIR8
MFLQRYFLNYLFIKDVVVIIDIFKNKNVQSGGIWHCNDAGTTLQMFKMFSENNLRFSNMR
VDENKNTVPLLGLGKVGIVVKTSCDGWINVFDSVDDKYAFKIPFLWLIITKNLTETTKTLS
MYNIELDSDVIISYKQENGYTLFESYHTGYKNGQFIVNKVGNWNGSLNLVEKDRKDLRG
LVLKTTVVILESQKVVNKTFIGYLEEEQPDLIKVDPVHKFKGYAVLKYFRDMFNISYEFQR
TKSWGYPKNGTFDGLVGALQRKEADIGGSPLFIKADRLHAVDYAAEAWVTKPNFIFRHPK
HLGGFHTIYTRPLADSVWWCVISLMVLSAVVLGLTFHLKIRVYRPLRLSGNVDVSTSLAF

MLIWSAVCQQGMTIRRSSTSMKIAMFTIFLLGVILYQFYNAMIVSTLLREEPKNIKTLKDLL
HSNLEAGVEDILYNKDFFKRTTDPLAREIYLKKVVHTDSPKYFSAEQGMAMVKKGGFAF
HVDSAVAYRIIRRTFSEKELCDLQDVNVFAPYVALAVAKDSPYKEIMAYGMRKLKESGILS
RLYSLWDESRPACVRTPDASVFSVSILEFSTALMVLVLGYGAALFVLAAEHLATRISRRHLI
EFRH*
>SsubIR9
MRLWQIFVVIGAFGLVNGEDFPSLITANASIAVVLDHRFLGEKYQAVLDELKDYIKELARV
ELKHGGVVVHYYSWTSISLKKGFLAVFSIASCEDTWELYSRTQEEELLLFALTEVDCPRLP
VREAITVTYAAAGEEVPQLLLDLRTSNAFKWKAAVILHDDTLSRDMISRVVQSLTSQIDDD
SATPVSVTVFKMKHEINEYLRRKEMHRVLSKLPVKYIGENFIAIVTTDVMTTMADTAREL
LMSHSSAQWLYVISDTNIHKGNISGLITALHEGENVAYIYNVTDDAPECMNGLMCYSREM
MNAFISALDAAVQDEFDVAAQVSDEEWEAIRPDKLQRRNTLLKHMQQHIFVNSRCGNCS
TWRVLAADTWGATYSNIYEASVGDPPKNGTNNSDTAGVITNIDLLSVGFWRPFDAVSFTD
VLFPHVEHGFRGKELPIVTYHNPPWTILKRNESGAVVSYSGLVFDIIDQLAKSKNFTVKIIPT
SNVKHAFNDTTIDMIQSKSAIQTMAAVAKGQAALAAACFTVLSDPFPGINYTIPVSTQPYS
FMIARPKELSRALLFLLPFTTDTWLCLGFAVLLMGPTLYTIHRMSPYYEAMEITRQGGLATI
HNCLWYIYGALLQQGGMYLPHADSGRLVVGTWWLVVLVVVTTYSGNLVAFLTFPKQEIP
VTTVAELLENSAFYTWSISKGSYLEMELKVRDFF*
>SsubIR10
LTRIHEKVEYTVEVKSPQSEIDVCGNYNENLGIMHRDIFEARPLGEYFIIIVDTYKDFPYLVS
RLIRSRSWNPSAKFIILLFNFATNLINKQHAENMLSCLFKYNAINIAVIVPEEKNVRKAIIYS
WRPYDPPKYCGYYNETARNRLVIENTCDKGILKNGYSVFGDRIPRDMNGCVLYIIALERQP
FVSRDIHDANIEKILINEIVKRYRLTTSYEIINSFRGEREKSGHWGGALNELVMKKGQLLLG
GIFPDFDVHEDFDCSIPYLADSYTWVVPRAYLSPPWIALVIIFQNSVWYSVIAGFSTCALFW
RILGEISRDSPRNRSLKHCF
>SsubIR11
MGMRQDIVEFSANYFKERNVPFVCLITCENEAWDVKFTNIMSSSFISVSKLNTLNATFQDI
EQCLRQFFTGIGVLMESHCRNTEQILYVASRNRWLDLMHVWLIFDETDDNDSKNISSGYVI
ENSKKSSKVMNILYNLNISIDADITLASRNDDQFKLTEVFNFGKLQGGNFFVTDIGSWRPK
NDNK
>SsubIR12
QFWKITRRQLFVTKIGSWRPNNDNKELYQWFKGCKFYRRYDFGQLRLRVISVATSKTFDP
EALTSLRPVPGVSTITRTTNMVLKEVAEIHNMKFIHSRTDKWLGSFDSASNASGAVATSLYL
QQQDLTSLFRYFESVFGKITVIHPAVSFLETRYYYRIPTKGPGKFENQFLRPLSAGTWWCLL
AVSAMCTVIMVFAARLEARPSSFQYALFSVTASVCQQFFEDDTYDMAVSRISAARKLTMF
VTGTACVLFYNYYTSSVVSWLLNGPPPSINSLQELLESPLELIFEDVGYTHSWFTVPGYYF
NKRNVEIEGVIRQGLQSKKRSTPLFIPLEQGIAMVKAGGYAYLTEAAHANSLISATFDKSEL
CELGTLISIEKSLLYPAVQYNSPYKEFMTWTMTRLLERGIISCIQRHTSPQSISCEGSSPRALA
LGGAAPAFILLAIGYLASTAIMLVERLVYNMQRRSRK*
>SsubIR13
FQKSEEPKYTALLKGAQLSAVAGGMEGSSETSKYEQAARTKGPISSQQLHQVRSERHAVF
DWRLRLTNLVSIDAMDTGSCDFSLSTEEFLSEKLAMIVPAGSPYLAVINKEINRMHKAGLIT
KWLSVYSARRSRCSPSAAAAQEVDNHTVNLSDMQGSFFVLFLGFFSASSVLLLEWLYNR
KKRQREQIVIKPYVE*

>SsubIR14
MLISAVVAFFRYKVVRSIFVVACWNSFEQNQFVREISKHGFAATFACDPAALDKVHEHGLQ
GVLYFKQFDDSVLEKVKRLHFSNRYYVWLIVGDGVPAELDRVRYDAEIALIRSKAPKSEM
YCLDDIHVNPSGRATAHPWASWMHTTGLHVMYERERIHRRLDFKKYPLRIAAPLGQYSD
EWYHGPYADYIVDETLTELDAGIRAGYAASSLLIDFLNATEVIETVELWGSTIHNSSMLLKL
YYGDSELAGGVLRVLHDRLNIVDYVMPVWPFNVGFTYLAERESSSNMFLEPFTASVWWT
SLGIGLVLAVALRVTARSQEEKDGSLYAVLATFLQQDASAVPKGLSGRWTFTVLSVCAMLV
HAYYTSAIVSALMSAGSGGPDSLRSLADSKYAIASEDYEYMRYLFFDVKTTWDDLEYLKR
KKKTSKFYQVLERGVELIQDGSTAFHSEYNQLFPHLRMFSDDQICKLQHVDTVPEVMSWI
TCTKEGQYTDMFRVAGTWLQEVGLGKRMVTRLQALKPTCRAQLLAERVSFGDVALLMG
LTVFGALLSLAILGLEILVAKNSDRENGKRLLSAGAGTGDIKVDDIEES*
>SsubIR15
MVTGEQAINITVLSSNMIPMEVIYELSETAAKIALQNFDWRFMTMVYFNSTLYFGVEQFL
KSYRKSVVIGRSTFYPKVVGKTRQYVLFGRDLTNIGNALEWMRNHEFDNTGKYIIICQSV
DINECNEDIAIQTLWEYKITNVIYLKSVENEAAAFTFFPLLNSSCIYDEPVRLESLKPCLHQE
SCKLFPMKLSNFNTCPLIVSAFLQVPFMSIKNGTPSGSDGDLLRVIAEKLNGSLKMMTPRL
GNGWGKLEKDGTWTGSLADIYNDFANISMTSAALTLSRFSNFQLSTAYNSAYVAWITHSA
TMIPSSLKLSYPFQENARIALGASFAIVVVCSLIFRSKFYYNLSRKLKLGRPHSSVTFYSWM
ICVGLGIPTTRLPKKTALLNIVLLWIWYGILVRTFYQVTLISAMKSNYYYEEFDSIDDAIEEE
YPFGGGAALKDYYIDYPLVYDNWKDVDTLDIIPTLMKISQGMQFVLAMNTLTARTIIWKQ
KIKVHMLPQKVVTSPCVLFFKKYSPLADPVNIVLSRLHETGFSDKFFKDYAPIVVHKTEDS
LESIKLEHYTGCYALLIIGWTVSFLLFVVEIVFDKIFRPNIVYNYKN*
>SsubIR16
LLERPKVFEPRLLSEPGFTPGVAAMTKITSNLMNLLKEHHNFRFRYTIADRWIGSPVRNSTL
AVTNSLFWREQDVTSTSARIFPTWLEWVDICFPPTTDIQTKFYYLMVERGVGEYENRFLTP
MSVGVWWSTLGACALCAAVLAAAARLEAQPQPELYAFFSVAAALFQQDFDSGSQGEKIS
SQGRKMTLLVVGLTSMLLYNYYTSSVVSWLLSAAPPAIASLDQLVASDLELVFEDIGYTRS
WLDTPGFYYYSGFVNPVEDQLREVKVTKAKRTVKPLQPVKIGIDLMRTGAYAYHTEPYTA
SQAIAPFFSDEELCSLGALQIMPPAHLYVMVQKRSPYKEFFVWRYIRNGSMMRLAERGHL
AATRRRFAAAMPACSGRKPRALSLGQAAPAFLALLQLATLALFVLLAERAVCRSRRRLAF
RQ*
>SsubIR17
MAGVELIISAVCNATFCGTVYDNPITELQLTKKQGDLIKLTEEINGKHLKIATYDNRPLSWA
EKDDNGTLVGRGVAFAIVDILQERFNFTYDVVVPEQNFEMGGRGPTSSILGLLNSSQVDM
AAAFLPKIAKFINIVSYSSELDEGTWTMMLKRPTASAAGSGLMAPFNSFVWYLTLAAVICY
GPCITFLTRIRTKLIRDERYLSLSPSFWFVYSAFIKQGTSLAPEANTTRILFATWWMFIILLSA
FYTANLTAFLTLSKFTLDIETPEDLYKKNYRWVSTEGGSVQYIVKNPMEKLYFLNKMILSG
RAEFRNFPSDVDYLPLVDGGAVLVKEVSAIDNLMYGDYLQKARDGVSEADRCTYVVAPK
SFMKRPRAFAFPHGSPLQPLFDTVFIYIVQAGIIDYLLHKDLPSTKICPLDLQSKDRQLRNS
DLLMTYMIMVIGLCAAAAVFIGEVLLKRYVNTKVSKQDAVKPKVKRTKKNSKRLRFDDD
GRPPPPYDSLFGNTKYNLNENSTRKMINGREYYEYKSSNGDKRLIPVRAPSAFLYY*
>SsubIR18
MFIQRYLLNFLVLRDIAVLVDLFRHKNVQDGVVYHCYNTGPALQMFKIFNKNDVRITSKR
VTHKTFIHTKVGVVVNTSCKGWLKAFDLVHSEHRFKSFFVWIVITENLSDTIDVLSPYRIEF

DSDVTIISKLDDIYILNEIYHTGFNRNGQLIVNEVGRWNESLHMDVKSRKDLKGLELKTTV
VIPQVQKIVNNTFVKYLEEEQPDLMKVDPMHKFKSYTALKYFRDMFNITYDFQRSDSWG
YPTNGTFDGMVGALQRRETDIGGSPLFIRPDRLNAVDFAAETWLTRPSFIFRHPKFPGGYLS
IYTRPLTAHVWYCVILIMTMAAIILGVLLNLKIIKVPGDDEDAST
>SsubIR19
MPSKLLFIFFAHYVYLSSSEYSLLMTTGNSGQIAKTAECVLKLSAKYFVEKKALSGSIVIINI
NSYTSTTQRLLLQTIHGGIKYSIMVKDSFYPHANASHFPEKAKNYMLILEEKSELTRNILQL
NKLPTWNPLAKAIVFYKVKENEDGEATAIELINVLREYKLLKTIIFIYDDVKEEVVSYTWR
PYSDTNCGGECDSVYILDKCKDNVVYQFEPQADMFPADMQGCPLVTYAVVSEPYVMPPI
RKLNNSTYNDTYEFRKGGEINLVKIISEFTNMSLIVRMSNHEENWGTIDWNGTSTGAYNV
LRNDEATLVIGNIEVTRTIRKWFDPTVSYTQDEMTWCVPRAGQASTWNNLVIIFQWSTWV
ATIVSVITLGLVFHALYYKEHDGKVTKWPTNSLLMTLSMLLGWGASFQPKTATFRILMFG
WLCFSINMGISYESFLRSFLMHPRFEKQISSERDLIQSGIPFGGREIYRSYFEANNASSFYLY
RKYNSTTFSEGIRRAALKRNFVVVSSRRQAAYQDQKLGRGAPLIYCFPESDNLYKYGVVL
LARKWFPMLARFNNIIRSVSENGLINKWNEELLIHTVSAEGTGTIEPLSIQHLLGAFMFIGF
MYAASLIIFLGELVYGWFQKRQKTWRPANKKIFRIIK*
>SsubIR20
MSFFHLISPLLRTPALQILLQSIINQFLFNSYCITLVSDKPMDFNFNLSFIYVIPKSTVEELKDQ
LLEVSEGGCSDYIVNMRDQNIFVAALDMVSKAGSVRRSDRKIIFLPTSESPSGKNQASNLF
SMKETNFVPNILIILPKDTVDTHCVLYDLVTHKFVGSNETMHQPMVLNQWDSCTEKFKTS
ANLFPHDMKNLFGKIVRVATFTYKPYALLDLDKAVIPTGRDGLELRIVEEFCRF
>SsubIR21
MSLFHLISPSLQAPAVNLLLQSILNQFLVDSYCVTLVSDEPIDFNLNLTFRYVTPKTTVDDLK
NQLLIGSEQGCSDYVVNMRDRMMFMTAFDLVSKAGSVRRSDRKIIFLPVPEDFNCKGEGP
DLFSMVETDYVANLVMILPTEEHHQSNCETYDLVTHRFTGSILEMRQSIILNQWDSCSNKF
KSNNSLFPHDLNNLFGKIVKVGTFSYSPYNMLDLDPAVTPMGRDGMEIRIVEEFCRWVNC
TIELIREDEHQWGEVFENGTGNGVIGSVVEDRADMGISGLYSWYEEFIYLDFSTPSIRSSVT
CVAPSPRLLARWAMPLMPFNWRMWCAVIVSFFFASIAFVLVKGCNSDKAFLTVFGIMISQS
QKSAELNWRVRSVTGWLLVAGFILVSAYGGGLASTLTVPKYEPSIDTMQDLVDRNMEWG
ATHDAWVFSLTLSDEPLIKRVINLYQTYTEEELEKKSFENKIAFGIEKLPGGHFAIGNYLTRE
ALVDMTVMVEDFYYGEVIVVLRKSSVYTEKLSVLIGRLQQSGLMLAWETQIALKHLNLK
VQQEVKMSRQRKTLGMVKPLGSEDVEGVFILYAIGAVISILIFMVEVLCNNSKFKTITV*
>SsubIR22
MEQPIAMQFVTNQTAKFYSREQNVVFITDLACPHVIEFLNESSARTLFRTPFRWILISDKKG
QKVKGSVIPEAIAHLDIFPDAEVTLAFRKDNVSYDLYLIYKVSSGSKWKTELFGIWNVQH
GLQKSLKSNEATILRRLDLDGFEIKICYVLTDNDSINHLSDEVNDHIDTITKVSFPTTNHLLD
FLNAGRKYYFANTWGYNVNGSWNGMTGYLVREEVEIGGSPMFFTSERIPIVDYISSPTPTR
SKFVFRQPKLSYENNLFLLSFRWTVWYSSVALVFLMFLALLIVAFWEWRKPGPNKESKEY
DAGILRASAGEIVILIFGAACQQGSPVELKGSLGRVVMLILFLALMFLYTSYSANIVALLQS
SSSQIRTLDDLLHSRIAFGVHDTVFNRYYFSTATEPVRKAIYEKKVAPPGVKPRFITMEEGV
KRMRKGLFAFHMETGVGYKFVGKFFEEGEKCGLKEIQYLQVIDPWLAVRKHTPYKEMFK
IGMKRIQEHGLQSRENRLLYEKRPKCSGRESNFVSVSMVDCYPALLVLSYGSIAALCLLAV
ENIIYRRHILRDRFKLRRYREN*
>SsubIR23

MFKFIAVTISAVICTVGADIPAIYREPPPKDLQYVLIDSLAVLTHHHDVSCAAVICDTVYLNV
FDGFLFRRTARVPYVMTVVEEREDLLSPNFYTLESLRAARKDGCNVYIILLANGYQAARL
LRFGDRHRIIDTRAKFIILHDFRLFHSELHYLWKRIVNVIFLKYHMQITGVDKSKAWFDLTT
VPFPNPIRGVLVPRRVDIWRNGKFHYNRALFKDKTSNLNGETLNVVYLGHVPSVVITESG
NDSKKIGGIESEILNTLAQKMNFIPKLYQPPNAELHKWGHKQPNGSFSGLLGEMVNGHAD
MALGNLQYNPYHLELTDLSIPYTSQCWTFVTPEALTDNSWKTLILPFKLYMWVAVLLVLLI
TGTIFYGLATYYKTLQTYKKDYRRSAKTKLDDKQDNTEDEKPVGLYLFGEIINSILYTYGM
LLVVSLPRLPTGWSIRLLTGWYWLYCILLVVSYRASMTAILANPVPRVTIDTLRELVDSKVT
CGGWGSETKQFFVNSLDEIGQKIGEKFETIDDPMEAADKVAQGIYAYYDNRDFLKYISVK
RKNSLVMQSIEEKLDNSTNVTTVVAAKTETERNLHIMTDCVVHIPISIGFHKNSPLKPLADI
YMKRVVEVGLVEKWLNDAMRPIQALETNEEEIKALMNLRKLYGAFIALGIGYGLSAIALV
GELIHWHFVVKRDPKFDKYAVDLYYMNKNKKQ*

>SsubIR24

MSVLYVVILLLYSFVQVTVSQTTQNINVLLINEENNAMAEKSFDVAKEYVRRNPSLGLAV
DPVIVVGNRSDAKAFLENVCRKYNDMLSAKKTPHVVLDFTMTGVGSETIKSFTAALGLPT
ISGSFGQAGDLRQWRNLNANQSKFLLQVMPPADILPEAIRAIVTKQDITNAAIIFDEFFVMD
HKYKSLLQNIPTRHVITPVKSFNKDEIKTQLRSLRELDIVNFFVVGSLKTIKNVLDAADEN
QYFGRKTAWFALSLDKGDISCGCKDATIVYQRPTPDATSRDRLGKIKTTYSMNGEPEITAA
FYFDLSLRTFLAVKSLLDSGKWPNDMKYITCDDYDGNNTPNRTLDLKTAFHEVKETPTYA
PFFIPEDDPMNGRSYMEFSTDLSAVTVKDGASIGSKALGTWKAGLSNPLSLSDPENMSDY
SAQLVYRIVTVEQEPFILRDDAAPKGFKGYCIDLIEEIRQIVKFDYEITLSPDGNFGTMDEN
GNWNGIIKELMEKRADIGLTSLSVMAERENVVDFTVPYYDLVGITIMMKLPRNPTSLFKFL
TVLENDVWLSILGAYFFTSLLMWVFDKWSPYSYQNNREKYKDDEEKREFTLKECLWFC
MTSLTPQGGGEAPKNLSGRLLAATWWLFGFIIIASYTANLAAFLTVSRLDTPIESLDDLSKQ
YKIQYAPLNGSAAMTYFERMAHIEVRFYEIWKDMSLNDSLSDVERAKLAVWDYPVSDKY
SKMWQAMREAGLPNSIEEAVQRVRDSKSSSEGFAWLGDATDVRYHVLTSCDLQMVGDEF
SRKPYAIAVQQGSPLKDQFNNAILQLLNKRKLEKLKETWWNNNPNAKKCEKQDDQSDGI
SIQNIGGVFIVIFMGIGLACITLGVEYWWYKWRKRPVIGDVTQVEPSKTTINNTDIKGDGFI
FRSRNLGLSTLKPKF*

>SsubIR25

MKQKMKYLTVLWFLFSKLNASYARVDLGTSFITSFILNEQKPTFVVLCDLCWRENEKVLL
ANEISKLGSRVSTSLKNEVKYQDHDLLFLLDLDCPRATQVIHKASSKGLFKRPYRWLVLTN
STEDDKLELLRNSPLLTDSELVLAERRDEAYFMTELHKPSLNHSLVVTPRGHYNGSFVDVR
PRRELFRRRMDLMGEPLTMANVIQDSNFSKYHLEDGLDFRNDPIAKVSWMNAKIAFLML
NATPRYIFSYRWGYKIDGQWSGMINDLNTGRADIGTTCVVSNIDRLSVVAYTDTLAPFKV
RFIFRQPPLSYVANIYALPFSSKLWVGTAMCAAISALAICISARWELRFGRHTSQLDGGISD
AVFWTMSAVSQQGSHMEPRRTPGRIMEFVVFTALMALFAAYCANIVVLLQAPSQSIKTLA
QLANSKMTIAANDVDYNHFIFKQYKDPVHVAIYKKIEPDKKKPQYYDLNEGVERIRRGLF
AFHSIEEQIYRRIEETFHASEKCDLTDIDFMNAFNPYAPMKKDSPYLELMRVAFKRLRETG
MISALTRRHHVSKPVCAEKGSAFSSVGLSDLKPLLALMGIGFGASVAVLMMEIAVFHMDQ
KIKKRSLE*

>SsubIR26

LINLITVIMRKVLLLFCFLGTVAGFFDIQEVTSDRMVRLPKDFTVAVKDIAEGLPTKAVTVV
RGNSTDIGPEDVFELLCLLSEHNIQVINLEINTQENKELYYAYLKKALDISDERTSLILCEPY

ECELILTEITERNLIHRSLLYIFYWPYGRVSDKFLATMKEAIRVAVITNPRESVFRIYYNQATP
NRLHHLALVNWWSGRMYKSPVLPSTDRVYSDFQGRVFEVPVLHAPPWHFVRFNNDSTIN
VTGGRDDKLLSLIAHKLNFRYRYYDPPDRSQGSSIAGNGTFKGTLGLIWKRKADFFIGDVT
MTWERLQAVEFSFLTLADSGAFLTHAPAKLSETLAIIRPFRWEVWPLVFATLLITGPALWIVI
AAPSLWQQRKRDQLGLLSTCCWFTTTLFLRQSSSKEPSSTHKARLVSVLISLGATYVIGDM
YSANLTSLLARPARERPIGTLQALEEAMKDYGYDLVVESHSSSLAILENGTGVYGRLAKL
MRRQKIQKVRNVEVGVRLVLSRKRVAVLGGRETLYYDTERFGSHNFHLSEKLYTRYSAIA
LQIGCPYLETFNNVVMTLFEAGILAKMTTDEYKNLPEQSRRSQPVTESDKQASEVTGDSA
SGSQPQSESKGLEPVSLRMLRGAFCLLGIGYTLAALALTVEIQLHRRSKLRNKLEPTETKK
SSKSN

>SsubOBP1
MPKNDVTEDQVGDIDKGKFLEERNVMCYIACVYSMSQAVKNNKIVPEAMIKQVDMMFP
PEMKDPVKDAIESCKSVGKKYKDICEASFWTAKCIYDYDPANFVFA-

>SsubOBP2MSRLFLLLIQVGVFVSCVCGVPKKTVHITPELAGKILIALQKCQPETGIDSDAI
VMIYDGKYRDDQQFKDFVFCCYKTTGFLRNDGFLNEDKAIKAFRNEPLIEEGIRRCGPLR
GKDPKESLFKFYKCFVDTTPITITL*

>SsubOBP3
MMLIKIIEFLVILAVSEAMTMKQIKNTGKMMRKSCQPKNNVEDEKIDPINDGVFIEEKEVK
CYIACIMKMANTIKNGKLNFDAAIKQADLLLPDEIKEPAKEAIGACRKIADGHKDICDASF
YVTKCIHDHNPSVFFFP*

>SsubOBP4
MNPSIVLCLIFSVAGINAGTVHLSGAQKEKATQTALDCIKETGVKPEVIAELKKGHLTEDE
GLKKFTLCFFQKAGIIGHDGKLNVGAALSKLPPGVDKAEAAKVLEECKNKKGKDAADTA
YQVFKCYHAGAKTHIVL*

>SsubOBP5
MLKGVIIFVFALVGCQADSPAPSQGVYCGFMPKNLLTCMGMPSLIGEDVIRRCSSSKGPCD
KYTCLFTQSGWLKGGLVDKKKVSSYFDTFAASHPSWTGAITHMKAACLGDKTLPAQGVL
INCPAYDTMHCALTSFVKNVSPSQWNPSAECQTTRENPGAGNPVRLDDCFSSQVPNDSSH
TLPRSA*

>SsubOBP6
MGGRLVWAVLLVASLRRGVRADVNVMKDVTLGFGQALEKCRDESQLSQDQMEEFFHFW
REDFRFEHRELGCAIRCMSRYFNLLTDSGRMHHQNTHKFIESFPNGAVLARQMVELIHAC
EQQHDAEADHCWRILRVAECFKASCQQRGIAPSMEIMMAEFIMDSE-

>SsubOBP7
MTTATLSLFLVLIAFGNCGKDKPVFSEEIKEIIQTVHDACVGKTGASEEDITNCENGIFKEDP
KLKCYMFCLLEEASLADEDGVVDYDMLVSLIPEEYLDRTSKMIFGCKHLDTPDKDKCQS
AFDVHKCSYEKDPDLYFLF*

>SsubOBP8
MAKVHRVVFLCVVAAAFSSVSALSDEDKETIKGFMVPLLAECGKEHDITMEDVIAAKKA
NDGDALNPCFYACFFKKIGVITEEGRYAPDVAKEGHKKFIHDDELVSKLDSVTDKCSSIND
QTVTDGAEGCEISKLLVNCLKENKDEFSLFDD*

>SsubOBP9
MTNFAVLCLLISLTSVTILVTCSKMSTETSTEAPQDSKTIDNTKVMVNSKDIESKAMLNDSN
AKAEIDLMSAMTMCNESFRIEMSFLIALNESGSFPDETDKTPKCFLRCVLQTLEIASMDDG

KIDPERVSDIFAGERDGENIMEIATFCSQRDEECRCEMAYNFLKCLFSMEIEKNNKKSRT-
>SsubOBP10
MVRKFSGLLCCLCVFGISLSDSAISADSEQRCKNPPTAPQKIERVITLCQDEIKLSILREALD
VIKEEHTMPTQRKRNKRDVPFTHDEKRIAGCLLQCVYRKVKAVDGYGFPTLEGLVGLYSD
GVNERGYFMAVLEASRECLMRNHDKFSRTVPMDNGRNCDISFDIFECISDRIGEYCGNAG
L*
>SsubOBP11
MEKYWFILIVAVIIVGDCDAMTKEQLKKTGKMLRKQCVTKVGVEEDKISQIEKGKFIEEK
DVMCYVACIYQMTQIVKNNKLSYEAALKQVDLMYPAEMKASIKTSIENCKDVAKKYKD
VCEASYFTAKCIYDDNPKDFIFA*
>SsubOBP12
MLRSVILFGVCAALTPYLVSAMTAEQKAKIHEHFEIVGMKCIKDNPINEDDIMNLRAKQVP
TGPHAPCFLACVMKDIGVMNDQGTLQKESALELAKKIFENEEELKLIEDYLHSCAPINKET
VSDGEKGCERAVLALKCMIDNASQFGFEI*
>SsubOBP13
MKILFSVILLTSVYADLLSQARNKGATLKPISACCDIPELGDPKPLLECSNPKLPGPCNDIQC
VFEKSGFLIDKNTLNKEAYSNHLRQWSENHKEWSEAVEKAIKDCVDIKDLRQYLTNPCKA
YDVFTCTGIAMLKKCPESAWKC-
>SsubOBP14
TQSMFVSTRGWKKSVYSFIDGKVLVHINCRCYNSWGLRCDDQRTAEKNWQDVKKTMCH
QSRRRRRENITN
>SsubOBP15
MTEEEMKVEFTKLIMKCTKDHPVEMSELMLLQQLVVPKKKETKCLLACAYKTEGVMTA
AGLYDIDHAYKIAELTKNGSEKRLENAKKMADICVKVNDVAVTDGEKGCERAGLIFKCTL
ENAPKFGFKI*
>SsubOBP16
MRYFETMVLAYVLLFGVLSMVSAITDEELADMRATAVPYLMACGSENGITMEQVKAAKE
SRNPDALNPCFIACFFKKSGVIDENGLFDIESTMKRTRKYTHDEEYLKNIDEIGRTCMAVN
DSPVSDGTKGCERAKLLLHCFWKRRSVFTQLLS*
>SsubOBP17
MISTVLLLTLLPVLAKCAGEGNIRLMEEDVAAALKTCAESDTSSPMVQRQKRYADNAPRI
DDGGKIESNQYSHERRNITDMKDQMLVLNATESDYDGYGSGNMGEKYIKTMPKSAAGG
SYPEVDANGMNRTRRSEPLFTKPDNDQCLSQCVFANLQVVDTRGIPREAELWNKVQSSVT
SQQSRAALKDQISACFLELQSEAEDNGCSYSNKLERCLMLRFSDRKPSGAQSKDNGQ*
>SsubOBP18
MEARSHQCNTMITFGCLLLLTVAFQVGTSQEQGPPHHGPPPGWGNHKCSGPPPDVKDPQ
NCCQAKPMYTEEEMTECGFQKLDDENRQGPPRPPDCSKQQCLLKKKNLLKDDDTVDYD
ATKQHMNDWVEANPEFKSAAEAATSVCMGEGGPPGPPKICEANRLMFCVGAVIFSNCPT
WQDTDDCKRLQEHMAECRGPN*
>SsubOBP19
MKYVLVAVLAAGLCGLVAGTAEVMSHVTAHFGKALTECRDESGLSAEVLEEFQHFWRED
FEVVHRELGCAIICMSNKFSLLQEDSRMHHVNMHDYVKSFPNGQVLSAKLVELIHNCEKQ
YDTITDDCDRVVKVAACFKRDAKTEGIAPEVAMIEAVMEKY*
>SsubOBP20

MFRHICMSFALWAAFLTAVMADDEMKEYRDSMIDATMEACLKEFDITKEEFSDARMTKNI
SNIDPCFIACFMKGRGFINEKAIFDDSVGLALAEKMIKNPEKIAKLKSISNICKSVNDEKYS
DGEKGCEISARLLQCLLDNNSHAV-
>SsubOBP21
MSKSCVYILGVIALTLGAVSAATEEEFKAIAEAARPILDSCGQEFGITREQIKAAKEAATGD
ALDPCFHACFFKKVGFIDANGMFDSNAAVEKNKKYFKSDDDVAKIELVGKTCTSVNDET
VSDGEKGCERAKLLVKCFIKEKATFTPFQSL*
>SsubOBP22
MRQLRWLFVLAVAMAAPPPMDEDMAELAAIVRSSCGSESGVDLGLVERVNAGTTLMED
AKLKCYIACVMETAGMMSDEEVDVEAVLALLDDNTRSRNEKSLRGCGTQKGADKCDTA
WKTQRCWQNANPGDYYLI*
>SsubOBP23
MKTFLVIAVCLVVAQALSNEQKEKLKKHKTECLTETKPDEQLVNKLKTGDFKTDNEPLKK
YALCMLIKSELMTKDGKFKKDVALAKVPNAADKPAVEKVIDACLANKGNSPHQTAWNY
VKCYHEKDPKHPIFL*
>SsubOBP24
MLKIFSCIIVSMASRLEILWLFSLCIFILLFVESNAMTRQQLKNSGKLMKKTCMPKNDVTEE
EVGDIEKGKFVEDRRVMCYVACIYSMTQIVKNNKLSYEAVIKQVDMMFPPEMRTAVKAA
AENCKDIAKKYKDDICEASYRTAKCMYDFDAENFVFP*
>SsubOBP25
MIEPKLLILVLLVSVISKVELSQDVMKNLSINFAKPMEICKKEMDLPDSAIKEFGNFWKDG
YVLSDRQTGCAIMCLSAKLDLLDQEYNLHHGNAQEFAKKHGADEAMAKQLVDIIHGCSE
STPPNADACAKTLTVAQCFKNKIHELDWAPNADVIMAEVLAEV-
>SsubOBP26
MKGCKVEAIKHDLSKNKQSLTIRCSVRLTGDYTLDGQIIILPVKGDGKFEIDIRDIVIKAATE
LETVKGADGKDHWHIKSWKHNYLTKGGARFDFKNLFNGNKVLSGPVLDFINSNWPEVM
KEIAPPIVYAIVEGVVNSVEELYQAVPSDELALA-
>SsubOBP27
MAGVARFSLLWRLLALFGTFLAQVEDRECSQEVMHNMTKGFARVLEDCKKQENVGDHI
MQDFANFWHEEYDLVNREMGCVIMCMASKLELLDEDMKMHHGNAQEFAKAHGADDA
LAKQLVDMVHECEKASEAVDDRCARALDTAKCFRTKIHGLKWAPSMRVIMEEVMAAM*
>SsubOBP28
SQTVMKDMTVNFGKALEACKKELSLPDTIFEDFYNFWKVDYDLKHRTTGCAIHCLSSKL
DLVDPEGNLHHGNAHEFAKTHGADDAMAQKLVDLIHNCEKSTPDNADICLKILDVAKCF
KVEIHKLDWAPNMDMILGEVLAEI*
>SsubOBP29
MTPNNTYGVHKCFIIATLWEKFPFEDWKMEWKHVLVVVVLVAVVRRSEAADAMKLLAS
GFLTVLDECKKELNIDDNIITDLYHYWKQEFALLHRETGCAIICMSKKLELLSEDGNLHHG
NTKEFAMKHGAADDIATQMISIIHACEAKGEGIEDDCWRILEVAKCFRVGIHEIHWEPKVD
VIVTEVLTEI*
>SsubOBP30
RTSPRLNGVPQRSAKRLAIMPAPAPYARMTASRRKCLTTPATPATPCPDLLRDQYYRSQQE
QKREQNAESKKNKKRSQRTSGQMPIS*
>SsubOBP31

MIIIPLVALCSILIPAITTAPPNRQVFVFPKEVSDHIYSTIKQCAGSENKFIELMTIVREGKVRE
DDEFKKFTFCSYHETGYSSDEGVVNADKSAPYYPDPAVILPILKKCEEKRGSTPVETTFEFF
KCFQDTTPFLIGF*
>SsubOBP32
MYFLKFLIIFMVVFGYAMGMTRQQLKKTLGIMKNQCLTKTGATEDKVVNIDQGTFLEERE
VMCYIACIYKSIQVVKKDKLDGELISKQIDLLYPQDMKESTKNGVAKCMPIQNNYKDMCE
GIFYATRCLYESDPKTFVFA*
>SsubOBP33
MSRVVYSVVCLAALFVSCYGAIDLSKYLKVCDRNSVDINDCMLEAVQKGIQTMVDGIPD
LGVPSIDPYKQKELKVEYKRNQIQAKMHMKDIYVEGMKGATVHDARVRADDDRLHIEID
LTAPKISVRGEYKGEGQYNALKINATGTFSTVMSDLVWTWKLDGVPEKNGTDTFMRITSF
YMRPDVGNMVSKLSNDNPDSKELTELGTRFMNENWRVLYRELLPYAQANWNKSGVRIA
NKIFLKIPYDQIFPIST*
>SsubOBP34
MLKPLGFFCILSIIVVGDVQAGKLDNVKQDYENILKECTDQTPLTQADMDALANDQKTD
NVKCVFACALKKTGMMDDKGKFSAEGIRKSTQKYLSDDPELMQRTEQFIETCKTVNDAP
VTDGEKGCDRALLIYDCTVKEAPNFKLF*
>SsubOBP35
ENRSGKIILSCQLQKSGRQATFKHGLGFLHNIFVVIKVTSIFQMDNKGMMSEKKMKETAK
KIFKEGDEMFTKVEELMSRCIHVNDAETSDGEKGCDRAKLAFECFIKHATELGLDVEL*
>SsubOBP36
MFRHVCVGFALWAAFLTAVMADDEMKEYRDSMIDATMEACLKEFDLTKEEFSDARMTK
NISNIDPCFIACFMKGRGFINEKAIFDDSVGLALAEKMIKNPEKIAKLKSISDICKSVNDEKY
SDGEKGCEISARLLQCLLDNNSHMV*
>SsubOBP37
MRVERRDGVSSNLYSIAPRVEGRTDMEVKGWFVSLAITCNKEYSVDPGELAMLQQHDIA
DSTSGNAKCLLACVYRTANMMDDKGNYDLDRTNAWAATEFNDSPTRLESAKSLFDLCKQ
VNEETVGDGEKGCERAFLLTKCLVANAPKHGFVAH*
>SsubOBP38
LTIQNKYISYPDCNFGLPFFVHINPRTSPGLTKCKSDDANCMRETAKAMIPIFAKGLPEYNV
PSLDPLYIKTINASEGNLLFVLSDVTATGLANCVPKKIQRDPANSKM
>SsubOBP39
MLRQALFLVAFALVKCEITEKSRAVESGKMMGLDTVHTVGKKNAEKFVKIDKDTIITHNL
KLEKRGKTSTSSPKSKEKEPDWSYTAIPEEVSQYVEQFKKNMTECLKEVQENDKRTVKRL
SPKVESPVHGECLIACVLKRNAVIENNKVNKG*
>SsubOBP40
LVIAKKQSSNKRRKCNHTFDFETLLQTSFNQDIIETFLKYWREDTLLEDPNMGCVVMWVL
RKLNLIQSDAKLNLDNAVAFLSAQGSDEYTSGVLADIYSLCLKSIDLSPVTEECHFGLALA
KCFRAGIFMIHWEPDFNASEEYDEVDDHIK*
>SsubSNMP1
MQLPKHLKYTAISGGAFLFGILFGWVMFPAILKHQLKKEMALSKKTDVRQMWETIPFPLD
FKIYFFNYTNVEEIQNGAIPIVKEIGPYHFEEWKKKVEVEDHEEDDTITYKKHDLFLFKPEL
SGPGLTGEETIVIPHLLILGPLVAISKTNAAMMGMVGKAVHGIFDKPTDVFMRTNPLDILFR
GFMINCARTEFAPKAVCTAFKKEAMSVFQFEPNNQIRFSFFGSRNNSVDPHVVTVKRGIKN

VMEVGSVVAIDGNTEMKFWKDSCNEYAGTDGTVFPPFLTEKDRLQSFSTDMCRPFRPWF
QKKTSYRGIKTNRYTVNIGDFANDPELNCLCDAPDKCPPKGLMNLMNCLKAPMYASLPH
FLDCDPSLLNNVKGLNPNVDEHSIEIDFEPISGTPMVAKQRIQFNIELMKTEKYDLFKNLPN
TMAPLFWIEEGLALNKTFVNMLKYQLFYPKRAVTVIRWWLVSFGGLGVLVGAAVHFKDD
VMRLATSGEKNKVTNVKPDEEEQKDISVIGMGEAPAKINM*
>SsubSNMP2
MLAKKAKLFFTCSFVFLIVAIILAVWGFPKIVSKQIQKNIQIDSSSQMYDKWLKMPMPMDF
KVYLFNVTNVDEVNAGEKPKLQEIGPYVYKEYRERNVLGYGPNDTVKYMLKKHFEFDQ
EASGALTEQDEVTVINFSYMAAILTVHEMMPGFVPMVNKALEQFFTNLTDPFLRVRVRDL
FFDGVFLNCEGDNSALGLVCGQIKSQTPPTMRPTEDGKGFYFSMFSHMNRSEIGPYEMVR
GTENIQELGHIVSYKDKDNLPNWGDQYCGMINGSDSSIFPPIDEANVPEKLYSFEPDICRSL
YASLVGKTTMFNMSAYYYEIHESALAAKSANPNNRCFCRKNWSANHDGCLIMGLLNLAP
CQGAPAIASLPHYYLASEELLEYFAEGIEPNKTKHNTFVYLDPVTGVVLSGVKRLQFNIEL
RNIPTVPQLQAVRTGLFPLLWLEEGATLPPSIQAELQASHRLLSYVEIGRWLLLAIAVLATLA
AGVLFARAANLIPRHNNSVSFILSNSNGHKG*The amino acid sequences of putative IRs, OBPs, ORs in other insects in phylogenetic tree are listed below:
>EgriORco
MMAKSKSVGLVSDMMPNIRLMQWAGHFLFNYYDENSGMNMLLRKVYACVHAFLISLHF
IFMCINMTQYSGEVNEFTANTITVLFFAHTLIKLVVFAFNSKNFYRTLAVWNQSNSHPLFTE
SDARYHQQALTKMRKLLYMICAVTGGAVISWVTITFFGESVRMITNKETNETLTEPAPRLP
LKAWYPFDAMSGSMYIFAFVFQTYWLIFSLGIANLLDLMFCSWLIFACEQLKHLKAIMKP
LMELSASLDTYRPNTAELFRISSSLNSEKMPDTTDADIRGIYATQQDFGMTLRGAGGRLQN
FVANPNNPNGLSQKQEMLARSSIKYWVERHKHVVRLVASIGDTYGTALLFHMLVSTITLTL
LAYQATKINGMNVYAFSTIGYLSYTLGQVFHFCIFGNKLIEESSSIMEAAYSCQWYDGSEE
AKTFVQIVCQQCQKAMSISGAKFFTVSLDLFASVLGAVVTYFMVLVQLK-
>EgriOR1
MDDQLECYVSKSLAAMLQVSRLAGLAPLEFEKKGDKWRIRVSRSWSYYGYVIITAIIITVF
AALLMDLQLDPRESVRMPSSTRRVVWITDVGCMLLLSATSVYSAPGLMRAMIATVNHIEK
INFDLNIRQYPMAFNKKQIWFFFVWTAVTIILTTVDHVVLLLMEDTKKTPTQSTISDMYMI
YYCCYWILQLRQLNFVITTLQVMYCLRRINDCLKNLLRELADRDEHSFISLFNTKLSKATK
KKQLKFKVDISVTTDFKKFQPEGTHKTAYYQDAIRRLGLAFGNTCDVVRHVDNAHGLVV
LMMLGSFLLHLVCTPYYLITGMFEFSSEENWTAHLVYNAVQFVWCFYHTVNLLMVIEPCH
LTQIEIETTCELVSHLMRCTDSVHDPLAMELEIFFRHLFLNQASYSAMHVTSISRSLVATILG
SVTTYLVIIIQL-
>EgriOR2
MSLICSQLKVSFKKIKNIFTESSFESLIAIVNFVPNLAGFSIRRKKIAAPFWILHLSLLLYVYG
VGCLVYQIKVADGAEDFIKNFVNVSLLILIFNISWWWLNKRPLLLETLQLIENSDVEARTTE
ENLREKHNKMLHRIKIIVLIFYMTNCTNAACIYLPNRINVSNEYSMALCVGLEPITVTPNG
QICSALLCIQELTIMIAVLNYQTLMLFIISHTATMYHMLAEELMSFNVYTNLAENQALVKER
LPIFIKRHSMILSTCDNLKSLYSLSLGVDFGSNAICICLFFYLSLQEMVKYMPILIYCVLAFFL
YCFLCQKLMTASEVFERAVYSCGWENFALNEKKMIYVMLRQAQKPVIILAADIVPVNMY
TFATTLQTLFKFVTVVKL-
>EgriOR3
MSSTTLEQAKNEIDMTLNLSIFCMRRIGLSFDAPKSTTAYLKQQLMLVLSVCGICYHVFSEI

VYIGLTLSNSPRVEDVVPLFHTFGYGALSIAKVSVLWYKKHVFIKLLNELAEIWPLDPLNQ
EDKTIKDNSLGALRMAHRWYFTVNVLGVWFYNLTPIAIYLYELWKGEDPTIGFVWVSWY
PFDKHQPIAHVAVYIFEVFAGQTCVWIMVCTDLLFSGMASHIGLLLRLLQHRLECVGTIDQ
TDEEHYQEIVDNIKLHQRLIKYCNDLENAFTLSNFVNVVLSSVNICCVVFVIVLLEPFMAV
SNKLFLGSALIQIGMLCWYADDIYYANLDVSISAYKGNWYVTNPRGRRALLFLIKRAQKPI
AFTAMNFTDITLVTYSSILYRSYSYFALLYTMYSES-
>EgriOR4
MKLISGGFSTHARTAGERHELDRILSPILFLQHIFAFQVLIPEWSWRNNAAKLICMLVLVLY
AFFGTIDFMHSTTDFSLFADAIFTLIIIFQFVLQVLFFIYNRETFSEVYVMAKESMFCMIEDV
SPERSVFLMKLFRNIVKAIYVVLVIPTLQYLFPVFWHYINGRRVTLSPSTSILMPMTSPTFEI
GLYLHTLFFVMVSIGVLIVNMWFVFLTCFFCEACDCAVRVLRVEEDGGTLYADRLNEALR
TFYILHVKIIQYLKTLNKMYKWPLLVPILGILSFTCVFLLVSTEKINWKLAMVVFQGFVELS
AYNVLGEKIKTKAEEIKTALIEFEWTSLRLRDRRSYCIVVCYANVGAGVRTALGADLSLLT
LSSVYKGSYQTYAVFNTMHH-
>EgriOR5
MIETLRKQLSFLAPVLPYGVIEPWDDLNPKLYHAIHIYWLKFYGMWYNNYSPKCVLYWL
QLLYTLIVLWLVCFFPGIGEVVYLLRRRDNIGDVAEGLYLFLSEMYTYVKVAVFWWNRKK
VITILRYMHCAEFKPKEKEHEVIYRKSIKTARFVMSYYSAMCVGAVSVGMIIPLTENFDILP
TNVEYPHIDVYKSPVYEILYIHHVYYKPATCIIDGAMDTILAAFIASVIGQIEILSFNLRNFD
MMAERKRNRAIRLMTNEGTHSKEFFMKTVLKDCIKHHNCIIRYVSMIESAFSVASALQFM
LSVMVLCLVGIQFLSIENPTSHPMQIVWMAIYLTCMLIEVFILCWFGDELIWKSMDLRLAA
FQSPWLKIDRSTCMFVIIFLERCKRPLKVTAGKIFTLSLDTYTVLINWSYKAFAVMRNMKK
-
>EgriOR6
MQSLHKFPNKTARFFFKLCLICYYFGMPNFWVASLPFPKRFDSIYRKYSRVITIFIYAFILTEL
TSLFTQNNLTQKQESNQMVFIMSHPMLWSYSVSFCRQENKVRKILTDIMKLKTVYNDLDV
EKRMMKKSKYYSTAYCGMCVLSMVMYTFDAVVQVLNKEGTFTTVITVWPLVDDMSIYA
NLCRFLWNVLWWSMMIRVLSVYAFVFSITSCMSHQFTDLQSYFYSLERIFRNEGMDQRQK
EYQYIAAYKIGIKLHADTIKCVKLNKSICGWIFSGQIIFNITMLVILMTQLLKAERSLMHVG
AIGIAAICIVWGTGLCMCNAGDVTVEAAKLGNAMYLSGWENCQGKSSVTARKLVLTSMV
FAQQPVVIKGLGIMMLSYESYVSLVKTTYSIFSLVFQQLQ-
>EgriOR7
MSDNISDRPRRYFGFHYILLRCTGLGWWHHPDENDTTNFPGWYLYYSIATQIIWVAGFVG
LETIDPFVGEKELDSFMFSLSFVITHNLTIIKLYLFFFRNKEIQDVVRTIEIELHDYYQNEATN
RRTIKICKTFTFAFIFFGWLTIGNSNTYGAIQDIHWKALVTTSNDSTQLPPRSLAQPIYIPWD
YQKDRSYIPTFILELVGLLWTGHIVMGIDTFIGSVILHMSSQFTILKEAIETAYDRTMIELRD
GTAYDADENLPGHGTDIFLINLEENNERIVRAFYTEKQIESALQTTLVNCIRQHQVLVGCVE
KFSTTYSYGFMTQLLSSMAAICVVMVQVSQDASSFKSLRLITSLAFFAAMITQLAIQCFTG
NELTVQAGRISEAVMQCKWERMSPRLRSLMLLMMMRAQRPLRLSAAGFSHMDNACFLA
IMKAAYSYYAVLSQKQV-
>EgriOR8
MIKLFLESLEDPQRPLMAPNYWILNKVGFVLPENKLGRIIYIMMHEIVTFFVITQYMELYVI
RSNLDLVLTNLKISMLSVVCIVKVNLFIFWQQKWKQVIDYVTEADKFERDNRNEPRGKIID
AYTTYCRRVTYFYWALVFTTFLTTTGQPLMRYISSSTFRENLRNGKEDFPHIFSSWMPFDK

YHSPGCWITVVWHVLLCAYGAAVMACFDSSVVVIMVFFGGKLDLLRERCKEMLGVNGV
GISDAEAAAVIKQLHKIHVMLIKHSRLFNSLLSPVMFVYVVMCSLMLCASAYQLTSATNA
AQKLLMAEYLIFGIAQLFVFCWHSNDVLIKNENVPLGPYESQWWTATLEQRKNIVIFTGQ
LRIKNMFSAGPFANLTLPTFINILKGAYSYYTILRK-
>EgriOR9
MSFVRSFQDFLSKKYYNFDKPDITLLNFHPQLYMILASQGVFFGKKDSYLRFIIPIFCIGVNII
AAGLEICNISLGVLNSDYNMACESLCYIIILGSCLTVYIGSLSNQQQIYDQIQDMEIDFKQIC
SFGPRHREPFLAGQLIVWKLCIIWTVWLMICGGMYLMYPTLILLYQSLIATQDENIKRPLVF
PMWFPHDDPYRTPNYEALFALESLYTVVYVTSFGGYVYALFHFLLHYYIMMDMIILDFQA
MFEGLDESVAVLPKTDPRRTAVQRTLNQRLGRIVNWHVSVYRAIKRASIIFRPTMIYIVRLT
SAVICLVAYQIVEKFEHGVFEILFTFLGASATLQLWIPCYLGTILRNKGFAVGEACWQSGWH
ETRLGRMIATEIVLIIYQAQVPIIIKYTGQPGFQLETFSAMMSAAYSYFNMLRQAAISMKE-
>EgriOR10
MTSWIRKVFRKKVEKPSEGVYICNRSDFESTFVIPKKILRWITIRLTHDISPSTSKWWDAFY
WFEFSNVSLAGVLEILSMLQTARGGSFQDAVEIFRMMPCFGMVVLGLIKSFSLVYYRPVY
ENLVHELGAMWHDQTLSLREYSIIDGAKKQLLFVMKGYYWCNNALLVSFLSPPVIESFKR
MLGYKNQMLLQFFYWLPFDPFQPVYVDILLFIQTWHALIVIWSNICGDLLFCAFLSHITTQ
FDVLALKIQSFIYVATDKQLIDTYPLSELGKENFQNEMQSQNNMEDKMLKDLSEIILRHKT
LIRLSGDVEGLFTFALFMNFMNSSIIICFCGFCCVVVEKWNEFAYKSFLLTALSQTFLICWY
GQKLLDSSEGVSHAIYNSGWYMASRKIKASLFIMLQRSQKKVRVTTYGFSVVSLDSYTKII
KTAWSYFTLLLNTYKQ-
>EgriOR11
MNKEDLSNWCFKNVFKTVTTTMNIVRIYPFTVKNRLWYIQALFFMIVNATCLIILLNSVFQ
HEIRSGNYMDASKNFAIIILCFNSNFKYYLMLYYRDSFAGLISTIDKDYELAKEFNEDEKNI
VVSYGKKGINVCKLWVFVGPGTGLIFPAKAFYLMLYYYFKNEAQLVPMFDMTFPGFIETY
KSTFLGYIGTFALCFVFDSFATCIFTGFEPLIAIFTSHISAQLELLGLNIRKAITESVNEEDMKL
KLRSINIKWQQLFRFLGHVQNNYMLLMELNMKGTTLLLPCGVFLIVQDYLNNNFNVEYIF
LVIAGLVHFLVPCYFCTLMMESSVRLKYAIYSCGWESCRSAGVRKTVLLMLIRTTKHFNIQ
TVFYSISLDTFSTMCRHAYSLFNLMIASFT-
>EgriOR12
MIKILSLLKFLEDPRYPCLGPHLRLLSFLGVWHPETKSNKTRLKLVVFYFSVAFFVSQYIKC
LIKFDVDSLKLILQYAPFHMGVAKTCFFQISYKGWERLIRFISSEELHQISKKDEKHDRIISA
YIKRSRKVTYFFFALAFFSNFSIFSEPYQKNQISENGTNIYVYIFDGYTPFAREPPGYYCSMF
IQTVVGHIMSACVVSWDMLVVSIMIFFAGQLRISRLICERSVNSKTPENSHQNIAKCHRFH
VTLIQYQKEFNDLISPVMFLYLIVICTNLGVCIVQIAEIEDDITTLISGCLFIGACLIQLLLFYW
HANEVTLESEKVRYSTFKSNWADANKDIQKKIFLLSLTTTKRLYFKAGPFNEMSLSTFIGIL
RSSYSLYTLMKGTN-
>EgriOR13
MTALDLEYKKTFSRYTALMKFIGIDFTNLGNLDCSKWRLCVYNIIFVPFICGQLSLVTRITA
DNFLEGAQCIPVSVMLILGVIKMATALVKRAEVKRMLQEIGELWPKNLRQSEEKSYIMRS
WLRRMGFLLNAFFVYAVVNLALFQVIPLLITIYNMHAGVGEYVFPFQLIDLWTVNSTATYL
ASYVWELASTVPCQLCLYLPFDLLIVTITSNVALLLRLLQVDLTNSIESQRDHGSFARIVGIV
KDHQKLLRISDQLSSIFGLVILVHVALSAVVICFFGFLTVVFGGVAETIANLLTVWTAVITIFL
LSLAGQLLCDASVGVADAAYQSMWYDSDLKVKRLVLFIIIRSQQPCYLSALGFSNMTLRS

FSKIMSTAWTYFSLLVQMYEE-
>EgriOR14
MKLLLKNTIWSLTLSLNLLKICGFLVPGKYSEQKKTFLKVYGIFWFIYTNGVYIIVQAGDL
LQIWGNLTLMVSTSFLLFTNVAFAMKIINVMLRQGAVQDIIDNGERELAAQETDAQIQIVK
RCDRETSRHLYLYSFLSVLAVLGWAASAEKSGLPLRAWYPYDTSKSPLYEITYFHQVSALL
MGATLNVSLDTLVTSLVAICRYRLKLLNLSLNNLCDGMLVERNKELRGGDSIALPRLVACI
RRHQAVLGSAGEIQRCFTEPILAQFTVSMVIICVTAYQLAVELHTSHINVVRVVGMIAYLLC
MILQVLLYCYQGHQLLIESSEIASAAYSCPWYLCSLSFRRALLVVMVRAKRDTRLTAGGFT
TLSLTCFTAIVKASYTFFTVLQRVEDRQK-
>EgriOR15
MSRLDILEDLNHPLMGPTLKGLKLWKIWQPNHSSSGFFHKYSVHIIGTLFVVTQYVELWLI
KDNLELALRNLSVTMLSTICVVKALSFVFWHNNWSNLINYVSTLEKEQLSKNDFKTTSVI
ATYTKYSRRITYFYWCLVTATVFTVILAPLFGFISSDEHRYLIRNGSAPNPEILSSWLPFNRT
RGIGYWISIVEHSAICVYGGGVVANFDSNAVVLMSFFNGQLKLLSKNCARLFGETDESISS
EEAAKRIMECHNHHVGLIKYSGMLNSLLSPVMFLYVIICSLMICASAIQLTMEGTGTMQRI
WIAEYLAALIAQLFLYCWHSNEVLHMSNKAEDGVYVSAWWSRDVLVRRSVLLLGGQLR
RSVVFKAGPFTDLTVATFVAILKGAYSYYTLLSKKED-
>EgriOR16
MKQKGRPNTEKIPKYMDYIELPLKLVACWDWFPKPAKEIHVLINNVYLCLVLFVISKIALG
LTVHLYTEWQDIMSSLDKLADSLPLVVSLAIVSYCAIYRKDLYDLMNYMNENFKYHSARG
LTNMTMQESYDTAKKFARIYTACTMFSVTMYAVLPFVLHQDPLQNWMYMDITRSPFIELT
FLCTLISQWFVGLAMGQFGVFFAANAILICGQLGVLCCSLRNVRFTAQLRAGARHAALAA
QHPEILLDERHSYIYTTSRMAESEYHYDTRDVNNFRDTATKLDMYASELDAGTEDALRDC
ARLCQVLDTYKRSFEKFVSPLLALRVVQVTLYLCTLLYAATLKFDMVTVEYLAAVALDIFV
YCYYGNQIILQAGRVSLAAYQSWWHTSGGRPRRAVLTLLLASLRPVVVRAGGFLPMDLR
TFLVIIKTSFSYYTLLVNVNEK-
>EgriOR17
MEDTSPFNKTITQLEALFRFAGIHIRIGITNTTDRFKARCLYFLHSFAANYNLFCNIIWCVNG
MMNGKDFLGIAISAPCIIISVLAQFKGFLLIKHEDKAKILIDCLKKLEDKYKHRPRNQEKKE
MVNSEVKFLNFVLKTLNIFYFILVAGFSISPLVLILVEYVKTKIFVPTLPFFALYGYDPFQRHI
WPFIYIFQCWSELAVVLNICSADYLFFTCCTYIRIQFKLLEHDLQNIIPTEKHNYKMFVDNE
NFKQNFNEIIKRHQDLMESANILEIIYSKSTLLNFFSSSLIICLTGFNLTILHDGATISTFLSFLF
LCLVQVYFVCFFADLLMTSSMGVGNGVYNSQMYLAEAKIGKNMVMILNRSQKPCKITAS
GYADVNLKSFMRVLSTAYSYFALLRTMFDD-
>EgriOR18
MFESIQKFGLSYCDFPTLLSNVTFFLRTLSLNVDPRNQTRTPILYTAVDIIFTASYCYVYLVS
MFWFIFWRCTETGDVLLALIIFSLGTSSQIGLQKIVFMFYHRLKLTDLARQCLDSYEAVAPG
SRFMINLTNTLRTVKKRAIVFWSVIMGNGCLYILKPLILPGKHLMEDDFIFLGLEPMFETPN
YQIAFTLTAMGDIVTVYFPSNATAFQIVISGYIEAQILAWSEELLNLWEDANKYYAEYRTIE
RNGTNSNLEHCKNQKDIIINDYIRNRLKYIIKTHATNLNMFKGLEEAFQGVIAVEFGLLVVG
LIAELLAGLKNTYMEVPFGLMQVGMDCFIGQKVADASLGFERAAYACNWERFNQVNMK
VIVTILMVSQRTLTLSAGGITTLNFVSLLAIFKMIYSTYTTFSAMIT-
>EgriOR19
MVSSLNSENLYLNRAKLVMKFLGVWVPPDVEGFLPKLYRVFMITLQYLFLLFQIVYIFQV

WGDLEAVSQASYLLFTQACLCFKVTVFLVNKNMQKELLQQMKSQIFSAQSLLHQKILKK
QAARIKRFLLAFMISSQLTCGMWALKPLFDDAGSRKFPFDMWMPIAPEQSPQYYFGYAFQ
LLTICMSAYMYFGVDSVALSSVIFCCAQIEIIKDKILSIDRVWDKKVRAINKARSENYKKLV
ECIQHHQAVVTFIKLVEDANHTYLLFQLTGSVGLICMSALRILVVEWKSMQFVSIATYLSV
MISQLFVCCWCGNELTATSEELHTTLYECIWYEQSLQFQKALAFAMLRVRRPMLLRAGHY
VPLLRQTFVSILRMSYSYFAVLNQAQSK-
>EgriOR20
MKEPRRFKPFRETFTKVTFALCVGMIYPNPRAARGQVIGITLQLATAAPFTAMILIDMYYS
WLDRDIINIIRHSTVIGPFLGGFFKMLLMFYYRDRAKQIMDEIDSDYESYNHLPDEFRRLAS
ESVHNSIVYCERIRVIIVSTCVMVFPGLAVVQMVYEFSFASEPHLYMIHDMNKPFASNIEDR
FESPYFEIIFVYTFYCSLLYVVNFIGFDGFFGLTIHHACLKMDLYCKAMDEAMKVPADVIH
KKIVSVIRDQNRMFEFVKNIQEAFNIWLGIIFLATMIQICTCLYHITEGYGFDLRYTIFVIGAV
IHIYLPCKYAAKLKAMSAETATKIYSCGWENVPSHSIRKMVLLMVARAQTPLEITAMDMV
TFDMELFLSIMQTSYSMFTLLRS-
>EgriOR21
MKVIKKIWKKITHTNALDQASGELEIAFFEDVYRVAYAAGLSRSDRSVMYLIYSTVVKVM
IGLLICGEVWYVSTATLSLDEFAASVNVIVIQTITLYKVKNMINYRDFYQKLAKSMENSNF
DITTTKRRQLMNFWAKSHEKYVKLLLGIGNCTLAAWHLYPLVDDLDYNLMLGVRLPYCF
ETPIRYFFTYLIVGIAFNCVSHFVMVTDLVMQAHLVPLICELSVLADCFENIVCDCCRVTGA
HREDLTSNNDFIREYLRRLGALVEQHKLILNYSMNFKLILSGPMLGQLAASGTLICFVGYQ
AITNILDNITKCLMSLFYLGYNMFGLYIICRWCEEITIQSQNIGEAVYCSGWESGLTLLPGVR
STILLVIARANKPLVFTAGGMYNLSLQSFTTLVKTSYSALTVLLRFRHE-
>EgriOR22
MEVLKDYPKEFAKLFRPSFNYLLRINMKFLAKDEGFVRKYWPHSYIMPCLLMNYVTLSW
NLAKTVARGEIFEIAYLVPPFLVSTQGILKAIVLAPKIELLSTIIEELGELWRTTGLTKKQIKTR
TTLLRRMNLCNGITYWIGIFGTGQYLLTPLAETLVRRLLLKQEFGLLFPLQCFYPFDPTASW
AVYLATYGFQCYGMLIGVAIYCGAELLMITLCALIGVEFTLLGEDMNRALQCDRSWDNEG
VGENDDSTDDNNEGLSIKEVIERHQRLCTLSKQLDEAFNQMVFLNLLFVGLITCFFTYSAQ
FSRGPSYKINNYVAVISSLLYSLYMCYYGDLLTTASLFQSFRIGNTAYESLWFDNDTQYQKII
LIIIMRSQKPCCLTSMKYAAVSMNMFTKVISTTWSYLSLMNQVYYEN-
>EgriOR23
MKIFIDNCHGTLWFCLNVIRWVGFLVPDKLTGIKKMLYPFYFFFWFMFMVGIYVITQTGRL
IQVWGNISDMTNASYLLFTNLAFTAKIINVVLRRNTIQAIIDQGEEVLRNETREDGKKIIHK
ASMDNRKLIYFYASVTMTTVFGWTIGVKNKQLPLQAWYPYDTSLSPAYEFTYAHQCIALV
ILAVVNAGLDQLVTSLVSVCRARLKLVNLSLKTLTEGLDIQQEMLTQDQEKVFAERLHDC
VKHHRSALESAAQIQSCFSEPTLIQFTVVLIIICATAYQLALEANNNIIRIVSMSTYLMCMIFE
VFIYCYQGSHLTVESSEVVQCVYECPWYRCSVPLRRALLMMMVRASKVLELTAAGFTTL
SLRSFMSIIKASYSLFSVLKQMEQ-
>EgriOR24
MRLQDYKRKFWEYVNREEFDKIRGHVDPIEYHHLFVKSANFYNLVDDDNTLSWKNILPV
ITYISAIAGGIMFFISFLHNLINFQIHLSTECGTYVLIMTYKLFMRITMDNGKKHYHNLLQA
LHDDFDYISSTAGKKYRLRFFENQKLTFKVCVYVALFLGSIGSGMLLCAIFAVIVTVYFYSG
EDSKRPLPYPFWFFDYDLHVSPAYEIVFAAAFVFTFFHTIQFIFLEQSQMIWIREMTCRADVI
NWNLEDMLIDISPAKKYEERQYNDALIRHRIRNIIIMHQAMYDLLEGYAAVFKKCLHFEQI

FATPIICLTAYGCAEKLDNGEFALKLFLLCTSAVTVAFIPCYLCTHLLEKVQSVGDTCWNIPF
WNAGPVVRAYLVLVMQRSKRPLPIVAAGFEDISLESYSRMMSRAYSCFNMLRQADFNYS-
>EgriOR25
MAKKLYLTAHPSSQSFYRVLALVMGVLGLNSERWPWFAAPRAVAYRVHRAAMPAVVPFC
VGTQVLYLVVYYDELPFETLCMIITICPIALIILSKYYIAQTESFKIIMEDFMTKLHIYYTYET
EREDYIQKALIAAEQFSRWSCFYYMFFCYVCYASFETVPIIHNLQKIESYSNITFRLDIIVHM
WLPFDYEFNFVNWAIVQSIMFYIGFIAITYIVLFDTVNMAIIYHLIGQINIFKHRITLKFNSKN
NNTQETIKKYLIETSQDYLYITKTVKKIEAAFGVNVATIYIHNLFSGGVFLYQIMTSSDTETF
LTYGMLIPIYSGILIAISIALENIRISAEDIPDLIYAINWQNWSISNQKMLLMLLVQVQPTLEF
EAAFKIKTGVQPALSIIKTTFSLYVMIKQTV-
>EgriOR26
MYLQDEEEEGEVVTRVTVDKIAEKKNTLKVEKTLLFDQSIRKLNVLFKFSGMNIKNKINT
PLDTLKYRWLYIANFVWVFSAVIVCFYIIIEGISLGKNFIEVTSVAPCLTFSILGMFKSLFYLA
NETEVFNLIKSLRELEEKEHERNTSVDKENIIKEDRRFLNAVINVLYFLNCSMIAVFDMTPL
LLMAVKYVKTQEFDMLLPYLDVFSFAPYEFKYWVIAYIHQIWSECVVLLEMGAADYLFFT
CCTYIKIQFRLLKHDFERIIATEDSEAFSDVGFQAKFTELIKCHQALIESSSKLETIYCKSNLL
NFLSSSLVICLTGFNVTIVDDIDIIVTFLTFLFMALMQVFFLCFFADMLMSASVEVSDAVYN
CRWYLSRPAVVKDLLLVQTRAQKPCKLTALGFADVNLRAFMTVLSTSWSYFALLQTIYGN
V-
>EgriOR27
MLKVLSKLEDPRQPLLGPNVKALKFWGLLLPNNVFLKAFYLFMHFAVTVFTATEYVDVW
FVKSDLNLLLNNLKITMLATVSVSKVTTFLYWQDYWKSVIEYVTRADLNQRNIRDRNKID
MINRSTEYCRKITYFYWCLMYTTVLIVMTQPMYKYFSSESYRENVRNGTGVYLEVVSSW
VPFSKTTIPGYLAASVYQSYAAIYGGGWITSFDTNAMVIMVFFKAELELLRIDTADIFGTM
AKPVDDAEAIERLKDCHRRHVDLVKFARIFDSCLSPIMLLYMFVCSVMLCVTAYQITIETSP
MQRFLTTEYLVFGVAQLFMYCWHGNDVLFASQNLMRGTFESLWYERGIQYRKDLFILTA
QYMKKIVFSAGPFAKLTVETFLSILKGAYSYYTLLSQSQIH-
>EgriOR28
MLSSIREGEKISLKDEYYIQHPAKQTFYKVIAYIMGIYGLNSEYWSWFSRPSDPMHHKAYM
LQRYIVIIFVPITSASQTLYLILNYKEFEFSELSVMLAIMPVGYVVTVKSASAQTETYRQVM
KSFMEKIHLHNYLDVEIFNEQKRDFVARELVRAEKRTRQTLIFVMTLVLVCLFSWNTVPIL
HNVKHNDDIHNITKRLEVCVHFWMPFDYEYNFKGYLFVLIIGFYCGFVACIIMNLFDTINF
AFIFHLVGHIGIIKYDLTHGFTEKGLTEEQLKTRLVKINRYYCFITETFHAVTAAYGLNVASI
YAQNLVQGGLLLYQFMSAGKDLDTLLTFGLMIPIFMGLLVLVSLTLENVRIQADDIPALVY
DLPWESMSVSNQKMVLLILNQVQPTLEFLAAFNIKTGVQPAINIFKTTFSYYVMIKSKDE-
>EgriOR29
MKLLIDNSIHVLWICLNLLRWVGLFVPDKLRGIKKLLFPIYFTLWFMFIVGMYAICQSIALF
RAQGDVSEITGIIFILFTSMAFVAKIINVNLRKEAVQAIIDEVDYELKQEHGAEGKQILKSM
NKEKIGLIILYTIDATITLFGWIATAINNNELLFRSWLPYDVTNFPAYVLTFIHQVCAITGLAL
MNVSLDHLVLLLTAGCRCRLQLVNLALRTLCRDITLTNKLLTLEEEDIVRQRLRQCVIQHQ
ATLESAAQIQANFSVTIFGQFAVSVLIICATAYQLAYETSNSMLWRIPMASYFIGMVLQVYF
YCSEAHKLTEESSLIADAVYACPWYLCSVPTRRALLIVMVRARRVTVLSAGGFATLSLTCF
VSIIRASYSFFTVLKQMDE-
>EgriOR30

MGFVIKNANRSISISLTALKLVGFWAPQGLSENQKIMYNCYGVFSFMFLLGIYLVIQVVDLF
LIWGDLPLMTGTAFLLFTNMAQAAKIINLVLRKRRVQAIVDEANECLLGVTAEEAKEIVKS
CDKETSLQQLLYFGLTVVTTLGWATSAEKNKLPLRAWYPYDTSSSPAYQLTYVHQVGALF
VAAYLNVAKDTLVSALVAQSRCRLRLLGAELRSLADDGREPHHRPLSAAQERAAWASLRG
SVARHRAVLAAARALQACFSVPTFAQFTVSLVIICVTAFQLVSQTGNTVRLLSMGTYLLNM
CFQVFIYCYQGNQLSEESGEIAGAAYTCPWYRCSVRLRRALLVVMVRARRPARLTAGGFT
TLSLASFMAIIKASYSLFTLLQQVEEKK-
>EgriOR31
MLFLKWKNEFWNYVNRTDFYNNREQIDPYQYHSLFAKFAILFGVMDNSKKSIWAYLTRIA
AIATGIGYGIIFFISAINHAKFFNLSNLTENVTFMLFTIYEIIMISCIMGGENNFHNLLQVMKT
DFSYVYTHGQTYRERFFKNQLLTFKGSVFLLIITLSTSVSMIICFAASWIIALYKMQDGDKR
PLMFQVSLFDYDLHATPIYEIMFFVSISFVVIYTYLYAFLMVTYVVWIRQITCKADIIVWNL
EDLLVDVVPAKNQEQKNYYSSLIKYRMREIITMHNDMYKQLEQFAAVFKMCIFYEQVTSA
PVICMTAFCTFMQFQKGERGGFLLILCIGCILVTFVPNFLCTHLVEKVESVGDACWDIPFW
NAEPMLRVYLVMMMQRSKRQLPVSAPGFARVSLETYANTLSRAYSCFQMLRQVDITG-
>EgriOR32
MEMPNYEDLLRQIKLNFWLIGIPYGDFKITWRYYILFVTVVLMAIAETGFLVSKVNKVNM
LEITELAPCTCIGILSCLKIAAIVLKRRKIFELTENLKSLYGGIEITSETGLQIRSQIITLKKFVR
YFFILNVMLISVYNFSSPVIIVYFYLKHGEAPFILPYQVLLPFEIKSWMTWAAVYTHSISSGFI
CVLYFTTTDVLYFVLTTQICANFKTINSKVKGVNTSNVGTVHSIIKDHQYILKLSEDLEEIFT
GPNLFNVVVGSVIICALGFNLAMGELKQVPGCVLFLTSVLLQILMMSVYGENMITESTKV
GAAAYKCKWYDFDEKTKKVILTLMIRSNKPARLTAYKFSIICYGSFCTILSTSWSYFTILRTV
YTPSDSL-
>EgriOR33
MDFNFDQFFKIPVRASKINSCHPEVKRDKKWLLYFIPTFSIFTSVFLLMLYNISLNLKNNDF
NQTCKNGVLSVVYCVVTFQLFVMIWKQDIILNLIISVKKDFDSAKSMSLNEQEILYEYAQK
GMHVCTQWLIISCCASGIFILKNLILLSYQYIIGEPKFVPFYDLIYPEIIERNKENLIVYLATYA
LLLFYGLYSTCMYTAFVPLGPIFMLHLCGQLEIVKKKIEDVFKGGDETQERLKEIILQLQCI
YDFAEQILLAFRVNYELTLKATTIILPITCYEVLEALDNGEVSLEFIVFIIGGVLISSSPCYYSD
LLMEKSEDVRMAVYSSGWESIYETDDYETARKISKTVKIIMMRALRPVVVRTLFRTVCLE
ALTDLFQQSYTIFNLMNAMWK-
>EgriOR34
MLSSLRKFGLAYCDFPTMMDNVAFFLRTLSLNVDPRDQTRIPVFYTAIGILFSLSYCYVYVI
SMLWFVFWRCTETGDMLSALIIFSLGTASQIGPFKMIFMFYHRVKLSSLVGQCLDSFALAE
TESRFLTNLTKSLRCVKRRAIMFWCVIIGNGFLYALKPIILPGRHLMEDLYILLGLEPMFETP
NYQIAFILTFMGICVAVYFPSNATVFQIVLIGYVESQMLAWSEELLNLYDDANKHFIQLKTE
QTININSSPNEIDAVTNKYINTRLKSIIKGHATNLNLLIELEKVFKEAMAVEFSLLMVGLVAE
LLAGLQNTYMEVPFALMQVGMDCLTGQKIIDASIIFERAVYHCKWECFNKSEMKLVVMM
LRSSQKTLTLSAGGITKLNFVSLLAIIKMIYSAYTTLSATMN-
>EgriOR35
MSEAKMSSKKQSDIFKLNFMFWKIFGLWSGRNPNKNYKYYSAIYIFITLVIYNILLTLNLAL
TPREIEYLIREVIFYFTEITVTSKVFMVIIMRDRILEAFNLMDGIELAGDDEISKKIIEETQAD
FNVFWKIFAAISHGAYSSQVFVPIVLYLIFHTQLNLPISEYYFLTDDLRERYFVFWFIYQAW
GMYGHMIYNVTVDSFIAGMLLNAVMQLKILNAKLINLKLGEQEYKLCKDTQEKIQMKRL

RKCLIRYDTVLKFCNTIQLTLDVTMFIVFGMSSIIICVVLCGLLLPSSTETFVFMVGYLCAM
TMQIFVPGYLGTLLGYESQELAFAAYSTEWLPRSESFKRSIRLFVVRANKPIQITGLKMFPL
SLSTFTSIVKTAYSFFTLIRSFQE-
>EgriOR36
MKNYKILKTYCRKIYLVGSGNFWFEDNKIGDDKSLFYRLCSSLLFFLYAFMTVLEIMAAVK
GHFPEDEKRDSVTFAVSHTIVMIKMFSVITSKERIKVLNKRMIEICERYEEEKLMADKYRN
MKINVVAYFVTVYGSLMCFVVEGLRKLLEGTHFVTVVTYYPTYEDNSTPANIFRLVATLIF
SVMMMNMIISVDSFTMVYLIMYKYKFITMRRYFEKLRENFDELNQRGFKEEAAENLINGL
IEGIIMHNEVLKLSKEIDKAFGLVVALQVLLSSGTAVSLLLQFALSDQLTFVASMKIIFFVFAL
FFLLGLFLCNSGEITYQASLLSDAIFYCGWHLCPPRVGRRNIRKMVHLAMMQAQRPLVMK
AFKMLELTYGTFLQVVRSTYSVFALFYAQNK-
>EgriOR37
MSRKLKKKFWTYVDRSEFNHMYEPIDPAEYHSLFTRLARLYDLVDDDKKSLVAYVTPFIL
LICGLTNATVFLISSVHHIQVFQMYYLTDCVTFLAIMCYQILISTSLMRGKKNYHKLQQAIR
HDFQYICTRGQKYREKFFENQLLTFRGCVGLFISTILTGSSMTVSFFISLTVAIIKGTKRPVM
FPFWLFDHDLEVSPAYECMLAYSLFIVGTYTFLYSFVMVTYVLWFREITCKADIINWNLED
LLADVQPAKTKEEDEFYTSLIKHRMSEIIEMHQSLLKLIEDYAEVFKKCLFYEQVTSSPAIC
MTAYCVSEQYKVGEIEWFLLTLCFGIMIVSFVPNFLCTHLLEKVESIGDACWDIPFWNAGK
VVRPYLVLMMQRSKRPLPITAPGFANISLQTYSTILTRAYSCFNMLRQADFSG-
>EgriOR38
MRQIDCFKINVTYWKFLGIWPGETRSQFYKYYGIVFMTLFVITYFILLALNFYFLPRHLDV
FIDEMLFFFTEVAIVSKVLSVYFMYDKIKSIFDILECDVFQPQASDALNIIVNAKKFIRRYFTI
VAVISYLSNLTHILSPLVAHIFLAVKLELPVCSYSFISDNFKDMLIYPLYLYQSFGMHCHMLV
NVNTDSFILGIMILAIAQLRVLDYNLRHLTDCKGPNDDLSEKEKVVKCRLRKCISHFVEID
KFCSLIEDTFGKCLFVQFSSSSCIICAILFRFTLPAPAAYFIFLTTYMAAMLVQILMPAWFGTR
ITETSSSLSEAIYSCEWIPRSQPFRSSVVMFVERMKKPLSITAWKIFPLSLTTFTKIMNTAYSF
FTLLRNMQALDS-
>EgriOR39
MDINSCSTFSTLRPHFDALARVGYFKIVLRERTPARLRLHYVFRIIIWTLVISYNFQHLIKVV
QVRHSTEQLVDTLFILLTTLNTLGKQLAMNLRRQRVDDIIRITNDHHVELIKHEAVLMSRL
LKLYHGAAVTCGILWSSYPVVNRVLGQPDQFTGYIPFDTTMSLAFYLAHAYMSIAITLQAY
GNVTMDCMIVAFYSQAKTQIQMLRLNLEQLVDGVKINDRLIGRHNKRIYGDNEDRILLQR
RFVRCVEHYKQIIGYVNEVGAIFGEAMAWQFFVMAWVICMTVYKIVGLSLLSAEFVSMA
MYLGCMLAQLFIYCYFGTQLKYESELVNQAVYNSDWLSLSPRFRRQLLLMMQVCSRPVA
PRIAHVVPMSLETYISVLKSSYTLFTFLDRQ-
>EgriOR40
MSILKYWWKKMSYTKALENSTGKLETLFFETVYRVVYITGHSTCEKHLVYILYSWFVKL
MILLLLLCGEIYFFSQPHSMDEIVAAINASLIQLLTVYRYKNMMVNKDMYKKLAISMESPHF
DVSSSTRQQLVDKFSKANEKYLKLLLAIGNAALAAWIIYPLVDEVDYNLVVGVRVPFEYK
TSILYPVVYFLVMLAFVQIAHFVMITDVIMQAHLIHLLCQFAVLADCFENILIDCQEGLEGI
HTNQLVYNRNFAEKYKTRLGNLVEQHKLILSHTMNLRNTLSGPMLGQLASSSTLICFIGYQ
IITMITDNYAQGIMSLFYLGYNTFELYIICRWCEEITIKSHKIGESIYSSGWETMSTLPGVGST
VKLVIARALKPLVFTAGGMYQLSLSSFATLVKTSYSALTVLLRFRH-
>EgriOR41

PKETTLVSTIFFVVKCTGLWRVEKQYTKHPIAIFAIFCYFGTYVFQTVALFLSKDHIEQLFEV
FSVISFCGMGILKLYSLSCRSKLWLSLLEKMKDLEQPNDRKEIKECHIATEYESDNEANSFE
VDNLTKIYNKKFKKAATILSRIYCFTGIVFILSPFIEFTFGWIRGVESNGYPHILPSWNPFDSV
HFLCYILVIICETVAATYCVCIHIAFDLTVIGVMIFVCGQFSSLVYQSERVGGAGKVCYLSKK
RDVRAHSRIIHCHQVHVLLIKIVGELDMLLKNILGFYFFLATLTLCTVAVRLKTEEMSAMQ
LASLLQYMCATLTQLYIYCSYGDAVYNESSISAGQGPFGAAVWCLSPRIRREVALLGFGM
MKHRRLHAGPFNTVDLPSFIQIVRTAYSYYAVLGSKSK-
>EgriOR42
MKVFIENCHGTLWFCLNVIRWVGFLVPDELTGIKKRIYPFYALFWFIFIVGIYVITQAGCLIQ
VWGNISEMTNASFLLCTNLALTMKICNVFLRRKAIQRIIDNGEEILRNETREEGKKIIQSAS
MDNRKLIYFYSSVIVITIFGWAIATEKNQLPLQAWYPYDTTKLPAYQLTYLHQCIAVALLAA
VDTSLDLVVASSISVCRARLKLVNLSLRTLDHDMNLMDEMLTPAQAKVFSDRLRDCIEHH
QSALASACQIQRCFSEAMLAQFTVTLASICSTAFQLAVEPNNSILRLISMATYLMCIIIQVFIY
CYQGNLLIIESYEVASAVFECPWYLCPVSLRRSLLVMMVRSSKVPELTAGGFITLSLNSFMA
IIKASYSLFTVVQQMEQ-
>EgriOR43
MDETYRAFDRVLSVAGIPIFAKENWQSKGWLIYQIFNFFIGMLCFIFTTGFVSTNSSDLLLFI
QGACIWTTGAIMTISLGICLYFRNDFRQFLNEMAFTDSILDMPLITFVLQSDRGEKLMELK
NMVEETQAKLLKYTRILLKTYVASVFLCATLYLCTPIYEMVQRKDENLRLLAFDMWFPW
SMENFRVYIASFILHAYCGYLCCIAYPGLQCTIILLAGQIIRQLSILNFILLHLNELALEITSTD
KGNWQLCCTSILSQCIDHYGKIKRFSNKLNIICRPFYLTLILVAIMLVCMCSVKIAISNKLSP
DTVKYYVHEFCFILVVLMFCLLGQQVANECEKLRNAVTEKWYIFDRSHKRNILIFSMALS
QQMPIYIFGTISLSLPTFTWFIKTGMSFFTLVMSVLDEVE-
>EgriOR44
MVLFKNMKKRFKSFITKDDFNNIPRIDPLEYHSIFIKFAKIFAVMDYGKKTMWMHPLSIFLL
LVAVISAMMALVSLGHHIRRLHIYNITECATYLFLMLYQMVMILAMGRGRKNYGNLLQSL
KDDYTYIYTRGQKYKKQFFENQLLTFQGSVATLIINVVTSSIMTICYAISTIIALATGTVTHK
RPLMFQLWIFNFNLRVSPVYEVLFVFSMMNIIVYAFLYSFMMLSYVIWIRQITSKADIIIWN
LQDLLIDITPTHNLEKKEYYNTLIKHRMREIIQMHHAMYGLLHDYADVYKKCLLYEQTTV
APGICMTAYCFSEKYNQTGEFAGFLLMLCIGCIAVSFIPNYLCTHLLEKVESVGQACWETPF
WNAGPVSKGYLVLIMLRSKRRLPVTAPGFQNISLETYAMILTRAYSWLNMLRQADIDLQQ
D-
>EgriOR45
LMVNEKNVHNLIENLRNLERQNRMTMQSDEKIKIIQEEKKFLDLILKTFNILHLLLIITFNL
CPLVIIAVKYFRKGEVELILPFLVLYPFDPYQIKVWPFIYVHQFWSACVVCLCISSCDLFFFT
CCTYIRAHFRLLQQDLKHVIKGDGN
>EgriOR46
NFLTVMTTNNFSFLKERFVFETNYVTNIGSKVFIYPFSGRSKTTVFCFILLCISLCITIIQLTIT
LIVSKFEDWFEIINIAPNLGVCILILIKYVKIHGNSEKYDKIITHFRNEVWDVIKPECDNHRRI
LKKYIDIIIIIIRFEYYYMVALCGVISLF
>EgriOR47
CFHVIMGVLGCGVIAVYDMNAVAIMSYLKGQMAILKGKCRNIFKEEFPVARETVVDNIKE
CHRHHSVLVETGLQNRRNIGLSSSQPLWWCNCF-
>EgriOR48

GMSSTSLGFGGNGITKSEKYWVIQFTTALVVQLFLVCWHGNEVIIEAKTVDRGVYESDW
WKGDKDLRKLALLLAAKLNAPMVLHAGPFTTLSVPAFVVVLKGSYSFYTLFSQIQNDK-
>EgriOR49
HGRVRRAGDDEATAGGASEVAAGGASEAAAGGASVAPHVRVLRACGFLRRRGARAHGA
YRAGTLALTGVYLVQECVYAAQERADMEELARVMFLLLCHVTSLAKQLVLARAAARVE
RLLRALGAPACARAVRGAGG
>EgriOR50
SFFVSKMEEVLLFDQTILQIEFLFRYIGINIRSGEQTVSRQLKCRIIFFLNLFWLSCDSLGAIY
WFIDAVRTQKEFLEITYIAPCISLTFMALFK
>EgriOR51
RARALLAAHARSAARFARAYAGAAVLTCTLWAVFPLLSHARGERVRFPLWVPFEYDSTPR
FAAVLLYTYYVTTLVGIANTTMDAFIATVLDHCTTQLTILRMNFEDLPERALALAAGGAAG
GAAYERALGRLFVDCLAHYDQICQTTKLVQDIFGTAILIQFGIGGWILCMAAYKIVSLPVLS
VEFASMTLFICCILTELFLYCYYGNEVTVESSRVAQAVYSMRWRRTPLRFRRALLVVMERA
RRPLRPAAGLVIPLSLDTFLKIIKSSYTFYAVLRQTK-
>EgriOR52ISMLVFLMTQMMKSERTMVNAAATAITATAVLISTGFFVWNAGDITVEADKLS
TAMYCSGWHNCHGIGAIRVRRLVSMAMTHAQKPVVIKGLGILTFSYRSYLSLVKTSYSVF
SVLY-
>EgriOR53
NVNLWYPFDGWYPFDKVKWYNIVYVWESLMTTVVIVMYVCANMLHISYTIYISMELVIL
GSTVEDLITSEDIRNISNMLHVVETHYRIKKNLKTVIKRHQSLIHFVSDLDYVLGFSNLLIY
LFGSVFLCLTILTALVVDDLYNSLRYFSFFFSLLFEVFLQCTMGQLLSDHSKILEKAIYFSNW
FYADQETKRMLLVFLIRTQKPLTLGAKGFLIMNFSTFGRICSLSYQFFNVLRTAYS-
>EgriOR54
CYYGNLVEFESRSINDSVYESDWLSLSPPFRRLLLIAMCRWSCPIQPRVSRIIPLSLSTFVQIV
KSSYTLYTVLVVSAPPKTE
>EgriOR55
LYGGMQVPQGCGPNSTRPAMLVFAVMVNPDILKANGKNAASATDREALVSLLCDFVEAM
NPGLVLTRNPLILKDRDLAGELKDVWLAVQAKREREKGMSQDVMYKVYDIDGIGGDEQE
DDKTYARNKHNEDSPSKGDKKPVKSSKQILLNAGQKSEFDSGMNNCQLNQNPNRDKTG
NTNADTTYCTPVYGQIVSSRENHNQ
>EgriOR56
RFPRRWKRGKLVLLRKEGRPADEPSGYRPIVLLDEVGKLLERIIAARLAKHLDRVGPNLSE
RQFGFRRGRSTIDAIRLVNDLYKDVSSSSGGVLL
>EgriOR57
SKRSSTMARSFRSSRASRSPLLSLQGLTMHNTPSRLKPRSLSLAWFNANGSFPDQRDLISHF
LSDHQVDILLVNETFLKPSKRDHKIANYSLVRNDRTTGPRGGTRIYYKRALHCIRLDTPPLT
HLEASVCRLSMTGHASITIASCYHSCNKELLKSDLEALLSLGNSVILAGDFNSKHLIWGCN
YTNPRGKTLHDLSSKLGFDVLPPVFPTHYHFNNDCRPDILDVALLKGVTLRLGSLEV-
>EgriOR58
GRIMDNNAPSRLKPRSLSLAFYNANGGLHDDVRGQRDLIQTFVSDHQVDVMLVNETFLK
PCKRDPRIANYLLVRNDRTTGPKGGTLIYYKRALHCIPIPTPPLIDLEASVCRLSMTGHAPIT
IASCYLSGSKTLLRSDIEALLSLGNSVILAGDFNSKHLVWGCNRTTSRGNSLLSLSEELGFD
VLPPASPTYFPDQHGRRPDVLDLALLKGVTLRLGSIEVLHELDS

>BmorOR1
MLLSFKDDSRSPNIQKPQNFQYMKILRFNLKIICAWPEKQLNEIRSLGHSIHRVILPIQSVVC
LACGILYIHFHFNEIPFFILASTFITVMMNLVTCSRTALVMLFERYLVLTGRFITVMHLFNFQ
KNSDYAYKLCTFVNRMSHFYTLYVLFSMFMGLGLFNLLPLYNNYVSGAFSDPYGPNVTFF
HSVYFAFPFDYSHNFRGYIIMALFNSYVSVTCSIGLVMFDLLMCLMVMHVWGHLKILSHN
LINFPRPKASHVITTPNGPTNVETYTEEESKEVFARLRECIKHYGTVDDFANDMSETFGVIL
LVYYGFHQVSLCMLLLECSDLSTKAMLRYGPLTLIMIQQLIQISIIFELLGSVADRIPDAVYQ
LPWECMDVKNRRVVYGFLRRTQNPVRFKAMGILDVGVQTMASILKTSISYFVMLRTVAT
>BmorOR2
MMTKVKTQGLVTDLMPCIRLLQAAGHFLFNYHADTSGMNMLLRKIYSSAHAVLIVVHYI
CMGINMAQYKDEVNELTANTITVLFFAHSIIKLAFFAFNSKSFYRTLAVWNQSNSHPLFTES
DARYHQISLSKMRRLLYFICGMTVFSVISWVTLTFFGESVRMIASKETNETLTEPAPRLPLK
AWYPFKTMSGGGYVFAFIYQIYFLLFSMALANLLDVIFCSWLIFACEQLQHLKAIMKPLME
LSAALDTYRPNTAELFRVSSTDKTEKVPDAVDMDIRGIYSTQQDFGMTLRGAGGKLQNFN
AENNPNGLTAKQEMLARSAIKYWVERHKHVVRLVASIGDTYGTALLFHMLVSTITLTLLA
YQATKINGINVYAFSTIGYLVYTLGQVFHFCIFGNRLIEESSSVMEAAYSCQWYDGSEEAK
TFVQIVCQQCQKAMTISGAKFFNVSLDLFASVLGAVVTYFMVLIQLK
>BmorOR3
MIFVDDAVIGIKDPREYRHLRVLRTSLRLLGAWPGHYLGEETGSKYECAPMFLLMFIKIAC
LYLTIVYLRNNADVLGFFELGHVYLTIFMTFVTLSRGFSLTWNPNYHKVVKKFITEMHLLY
FKDNSEYAMKTHRRVHKISHFYTVFLKVQMIAGLTLFNVIPMYNNYRQGNYASDRPANIT
YDLSIYYETFDILNTPNGYIFICVFNWFASYICCSFFCSFDLILSLMISTVSGHFRILIHNLLTF
PLPEAITASKKFVDKHRCNGNRSEFVLEEAKLYSPAEMWQVTDRLRQCIDYHRKLVEFTG
DISEAFGPMLFVYYLFHQVSGCLLLLECSQLNTAALVRYGVLTVVLYQQLIQLSVIVESVG
TVTGRLKDAVYEVPWEYMDTSNRKTVAIFLMNVQEPLHVNALGLAKVGVQSMAAILKT
SFSYFTFLRTVSE
>BmorOR4
MFKIIKNIIVENDALKQVEKPQEFQYMKWVQYHLKYIDGWPNMDMNKKNVSKIRFHKR
HLLVVEQTITFLSQMFYIVKNYGKLSFFEIGHSYITALMTIVIFSRSVVTALGRYRKIARYFV
SSLHLYHYKDISEYALQTHLLVHRLSHYYTVYLISLVVTGMLLFNITPLYNNISSGVFNSPRP
ENMTFQHAVYLGLPFDYTTDIKGYFVVFILNWHLSHIAASYFCTFDLFLSLLILHLWGHLRI
ILNNLKTFPKPYTNNSMYTEEENQVVLLKLQECIRYHNFIISFTVMMSNVYDVVIIVYYLF
HQVTGCLLLLQCSTLDWESLSRYGPLTLIIFQQLIQVSMIFEILGFLSDKLPNAVYSIPWEAM
NVTNRKLVQVLLQKSQKPIQFKAMNMMSVGVQTMASIIKTSISYFIMLRTIARD
>BmorOR5
MLLYYPNTQVKEKVNNVEEFTYIKFLKSFCKIMDFWPEREEKNSKTRIFRLRYILVLQFCF
TLVAGVLYLTNSVGKQTFYDLGHTIITVLMNVVSLSRLILRCFKKYDVVGQQFINKIHLYH
YRNDSEYAMKIHTVVHKISHNMTYIFSFCIIFGTVTFNLTPIFNNIGSDAYKNPRPDNVTLQ
QCVYYALPFDYTGNFKWYLLVAIFNVQKTFFCTSLFILFELSLSLMIICLWGHLRIFIHNLNH
IPAPRNSFEYTKEERQEVDDTLKKCIQHHTLIIGFVRIMSETYGLAVLIYYAFQQVVGCLLL
LQCSQMELKTVTRFGFLTLVLNQQLIQISVIFELLGYMSDKLQDAVYCVPWEYMDTSHRK
MVYMMFRQSQIPLQLKAMNMLSIGVKTMVSILKTSVTYYLILKTVTTD
>BmorOR6
MKEEYYLQHPRTQLFYKVLAHVSTIESTIDLTWWGYTFPKYVGWFYHLQCNVVRLFGKC

VVVSQILFIILNYQTIDKSVFIIAITITPLGALVGIKAESAKAECYVNLMKNFMDKVHIHSIY
RKNENNEFVKKKVIQIERVSRFTAYFLVILIAINCLSWMLKPTLHNIKHFEEIMNKSMEFQY
YIYFWTPLDYKYNLRDYIIIHTLCIYLGATAVTVIVTFDIFNFIAVFHVVAHIQILKNNVKSN
WSDDFNESEKKGYLVSILEYHAYIIRIFGEVQSAFGLNVASNYLQNLIEDGLFLYQIMNGEK
ENVLMYGLMIILYLGGLIFLSIVLEEIRRQNYDLCEYVYALPWEGMSLENQKIFVVFLQRT
QPDLEFETVCGMKAGVKPAFSIVKSMFSYYVMINSRF
>BmorOR7
MLLYHPNTQVEEKVNNVEEFTYMKFLKSFCKIMDFWPEREEKNSKTRIFRLRYILVLQFCF
TLVAGVLYLKNNFGKKTFYDLGHTIITVVMNVVSVSRLILRCFKKYDVVGQQFINKIHLYH
FRNDSEYSMKTYKAVHKISNNMTYIFSFSIFVCVVTFNLNPVFNNIGSGAYKNPRPDNVTL
QQCVYYALPFDYTGDFKWYMLVAIFNVQKTFFCTSLFILFDLLLSMMIIHLWGHIRIFIHNL
NHIPAPRNSLEYTREERQEVDNTLKKCIQHHTLIIGFVRIMSETYGLAVLIYYAFQQVVGCL
LLLQCSRLDLKTITRFGFLTTMVNQQLIQISVIFELLGYMNDKLQEAVYCVPWEYMDTSHR
KMVYMMFRQSQIPLQLKAMNMLSIGVKTMASILKTSVTYYLMLKTITANEA
>BmorOR8
MSLSTRCLLKDFCKYVYYAGAGNFWYEDIYKETVPYKMYVVISFFTYTVMIFLENLAALF
GKLPEVEKNSAVMFAAIHNIVLTKMFLLLYHKRSISKLNCEMAAVGENLEEASIMRRQFRK
MRLGTALYFISVYLSLVAYGVESARRTIVEGAPFYTVVTYLPDYDNTTVLASFLRIFFYITW
LYMMLPMMSADCMPIAHLITMTYKFVTLCRHFDQIREKFQINVKIMAKTEATEILKLGFIE
GIKMHQKLMYLADEIHRVFGIIMALQVCESSAVAVLLLLRLALSPHLDLTNAFMTYTFVCS
LFLLLALNLWNAGELTYQASLLSNAMFYSGWYFCDFEKDWCRDIRRLVLIGCAQAQKPLI
LKAFGVLDLSYETFVSVARMTYSVFAVFYKRGD
>BmorOR9
MVARRPLQFHQGRNVDNVEDFKYVKWLRNHLKTVDAWPVHSKSKRKIQKRYVLPIFSA
ACFISQTVYLKNGIGTLSFVVLVHSYICFLINGSCLCRGILIATERYKRLATCYLKTVHLFHH
KNRSEHAMKIHVIVHRLSHYYTIYLISLVFVGMVLFNFMPIYNNINSGAFKSPRPENVTFQ
HAMYLALPFDYTTNIKGYFVVFILNWYISLVTTSHFCTFDLFISLMIIHLWGHIKILMCSLED
IEGFVPGSSFKFTIEQNRKIYLILQECIRHHQFTIDFTNEMSSTFGLVILFYYFFYQVSGCLLL
LACSQMDIESLSRFGPMTFILFQQLIQLSIVFELISSLSENLPNAVYNVPWEFMDKNNRKMI
QVLLLQSQKLIQFKATSMMNVGVQAMATILKTSVSYFIMLRTMYQEH
>BmorOR10
MAFFIKNKMFGLTITLNTLSWAGLIMRDQYTKTQRIIVRVYAWLVFLYLFVAAACVQIADII
DIWGDINLMAETALLLFMEFAVISKLLTLLLRYDRIMEIINGTEEILYFENGLEGQRIIASVDK
ETTRFLQFNSAFVVLSTTFWFMGEHSSTFFIRAKYPFNDLKSPGYEFALIHQCMMMVFTG
YTVFNINIFFASVVAGCRCRLNLVALSIRNICINIPVNKKNLITPEEEKIVKERLHCAISQHKC
ALNAAKDIKNCISEFLLVQFTVSIIIICTTAYQLAVNKAIGNIQKTSMVGYILGASLEVFLFCF
QGEFLRNASEEIADAAYECPWYTLTQPLKRTLLIIMMRAQSPVILTAGGFIDLSIREFMGILK
ASYSFFTVLQHVNE
>BmorOR11
MDEHSHFETSLNKIKVLFKYSGMNLENTVTNTYEFLNHRWVYILNHAWTLAAVTFICIGIS
NGQNFIEMTCIAPCVAMTVLAVSKSFFHYINENAVKSLLENLIELERTDFERTKSVQRTEIVA
TEKQLLNMVINVLYVLNCSMILVFDMTPLIIIAIKYWTTNKFVRLLPYLDIFVFVPYKFEYW
VMAYILQIWAECIVLLFIGAADCLFFTCCTYIRIHFRLLQYDFERLTSSRRESDGLRDDEDFR
ETYTNLVKRHQGLIESSSILEMIYSKSTLSNFVLSSLVICLSAFNVTVVNDVTIVMTYLIFLA

MSLMQVYFLCFFDMLMSASEEVGNAVYNCSWYTEKASTGKDLLFTITRAQKPCELTAAH
FAYVNLKAFMRVSFTSASITTLPTI
>BmorOR12
MTRITDVFSLNFIFWKFLGLWGKSAPSKYNMAYTVFYLFASLFVYDIFLTLNLIHTPRKLET
LVRETMFYFNHLVAVTKILMMFIMRKKILVIFDLLDCEEFKPNDENSQEIMKRKTDFYYIY
WRIVAVTSNLSCFMLVIGPLIKMLIWKIELGLPVCKFYFMSDELRNKYFVIWYIYQSFGIYN
QMVNNLNLDTFNCGMLWMAVGQLQILKTKFVNLKLNDFENGLDLKSRDDMQIERLRKY
LTHYEIILKYCAIVQDILNITIFVQLGMSSIVICVGLCGFVAMPSNTETAIFMFSYLTTMTMQI
FVPSWMGTQISFECGELMSAAYSCEWIPRSKLFKRSLILFVERAKTPVRITGLKIFTLSLDTF
TSIMKTTYSFFTLIRQLQVDEVN
>BmorOR13
MAPKQIDCFEINWKFWKFLGIWSENKPHRYYKYYSKIFITFFVILYDVLYTINFYFVPRQLD
LIIGEMLFYLTELSVLSKVFTFIIMRHKLKIIFEILESDAFQTDTEEELKILHRAKVFIKRYWKI
VALVSITANLTHISSPLLKNLIFKVELVLPVCSYSFLSESFLKTFEYPLYFYQIVGIHFHMLYN
LNIDTYFLGLMILIIAQLDILNVKFRNLKSGKDHTQLNESIMGLNKNLDHYNEIERFCSLVQ
NIFSFTLFVQFSMASCIICVCLFSFTLSVPVEYYIFLATYMFIMIIQIMVPCWFGSRIMDKSIL
LSSAIYNCDWTSNSKDFKINMRLFVERANKPLSITGGKMFSLSLATFTSIMNSAYSFFTLLR
YIQTRE
>BmorOR14
MSNYIFKPFHETYRIITFTMIAAMIYPNPATEKRRLIYIGLMLLSVIPLAFMIVTEMYEFFMA
SDLNNTIRHSTVIGPFIGGFVKVALMYYKRRQANELVSEINRDHLAYNGLKGEDREIAASSI
RNCQIYCELGWTLIVMSCGLSFPVIAILLKIHSFTFKLDSTKHMIHDINNPFTDDPEDRFESP
FFEIMFVYTFFSSFIYIINYVGYDGFFGLCINHACLKMKLYCRALEDAMRSDSRRHEKIVAV
IEEQRRTYEYIALIQDTFNIWLGLIYVATMIQMCTCMYHIVQSFNIDVRYIIFVISIIHIYLPCR
YAANLKCMAAETPTLIYCCGWESVSDLRIKRMMPFMVARSQVIVEITAFNMFAFDMELFV
WIMKTSYSMFTLMRS
>BmorOR15
MMTLVYQTDIFKPNVFFWKMFGIWADRKSSKTYKYYSFVFLFITLIMYNSLLAINLLYTPL
KIELLIREVIFCFTEITVSTKVLMILFKRNKILDAFDLLNKNEFRGNSEESSAIIQKNNSAYKT
YWKLYAILSNFAYSSQVLGPLIVKLIWKTKLELPICNYYFLNEELRHDFFSGWYIYQSFGM
YGHMMYNVNIDTFISGLLMMAVTQLKIIQTKLLSLKLNPRERKMDRGLMNITEVLKLNEI
LKHYELVLKYCSTVQSILDVAMFVQFGVASAIICVAMCGLIMVRSSTETLLFMVTYLFAMT
LQIFVPAWMGTQLHFQSQELVFAAYNSEWIPRCQSFKRSIIIFVERAKIPITITGLKMFPLSLA
TFTSIMKTAYSFFTLIRNMQALQEE
>BmorOR16
MSFNSEDLYLNRAKFVMKYLGVWVPPENENFARKFYKIFMMSLQHLFLFFQIIYIVEVWG
DLEAVSQASYLLFTQACLCFKITVFQINMNKLKELLKQMNGYVFQPKNINQQNIIKVQATR
IKRLLFAFMISSQLTCGMWALKPLFDDVGSRKFPFDMWMPVSPERSPHYHLGYSFQLVTIC
MSAYMYFGVDSVAFSSVIFGCAQIGVIKDKIMSIKPLGIYRNHKTYTKISRYNRKTLIECVK
HHQAVISFTELVEDTYNSYLLFQLVGSVGIICMSALRILVVDWRSVQFFSILCYLSVMISQLF
VCCWCGHELSATSEELHTILYNCAWYDQDVKFKRDLNFMMARARRPILLRAGYYISLSR
QSFVSILRMSYSYFAVLDQTNK
>BmorOR17
MREDKMEINNSQKFYTKMIFRYLYSVGLGDWWYQHEDRSDSHRKLYCLWAVISNAYIFL

NICNELLANFRKDLTDVEKNDAIQFSFAHPLIFAKIASFFFNRKKIREVFGRLLEENRSVYSC
GELEKESMKQIKRYSLAFIGVSYMTLVMSTIDGLRAHFKEGIPIRTEVTYYPSPSNSGVIVNI
LRFLVEFHWWYIVSVMVAIDSLAVASFVFVTFKFKLLQRYFKDMGLTVRRDQSNMTDEAL
ADKFRRDFIVGVKLHENALWCAENVQKAFGWVYSVQVFETVALLVMCLVKLVTTNHNM
IFLLANFAFMLCVIILNGSYMMPAGDVTYEASEVPTSIFLCGWELVRQTDLRFLVVVAIQRS
QVPVIMKAFGIMTLSYSNFIAVSLFKFYVQFQINLF
>BmorOR18
MGDRMVTRGHFFDFNIKYLFYVGLWPSNEAKRIEKIAYKIYEYQLHVLSLIFLVTTGIGTY
KNHKDIIALLTNLDKTLVAYNFVFKVIVFVYKREELRKLIEQIVQSGDQITEDRKALMAKL
VIVLTGISTVIITAFSCLALFEGEMTIDAWMPFDPMKSKMNLFAASQILAATFVVPCGYRAF
AMLGIVCSLILYLRDQLVDLQNKIRDLRFATGNVEKLRDDFKLIVKKHVRLLGYSKVIEMI
FKEYFFIQNMAVTAELCLNAMMVSVVGLEQKTLAASFLAFLSVALLNAYIYCYLGNELIV
QSEGIAMAAYESSWILWPVDMQKDLLIVITAAQKPMKLSAGGMAVLSVQTYSQTLYNGY
SIFAVLNDIVN
>BmorOR19
MHEFVINVQNETTKLYDQLNIILYILGLQGIWVDEIKLSRRFHVFFKVVTFILHIMCGMFAG
LQFFAIFTQNSLNSQQKSDVIVIGISNPMAYIFCINFIRNRNEIKDLFYHLAVVLKIYYNDVEI
EKSMVNKIKSYLSTYVFASITILVSNGIIAFFQTINSDEPFLGIITAWPDKTDTSKTASYARIGF
YLFWCIHFFRISTVFAVIVCILISIKYQYKILCSYFESLNKIFDDETSSHEVKEAEFENAFCNGI
KIHTQIIWCVRRCQIMCRTVFSANIMLDTFVLVILMLAMVNSENDFYGLCSQMSSVLVTV
VLMAFFMWTAGDINVQASQLPDAIYGSGWYNCRGKSSARIRSLVTISMNKAQQPILMWA
LGFVELSHKNFVAIIKSAYSVFSVFY
>BmorOR20
MIQASKYPNSKTKELFRKIAHIAYICGLPNFWIEELNLPKSFIRVYDKIVRIFNVATYFFLGIE
IAAHFTQHHLTNKQKFDLLLYSISHPILNGYGVIVSRQVGNVKKVLLDLIVNLKVKYNDPV
IEEAMIKISMTYSVSFITNCVLSMLTYTFDALLMVYKKGVTFNVIITAWPDVEDTTTEASIG
RIGFHIFWWLFVTRPFAVYVLVINLTTCLSHQYMNLQSYFFHLEDIFKENLSQNEKEAKYE
AEYKIGVMLHANTLRCTRRCHMVWNGVMSGQIIFNISLIVIIMAQMMNSDRTLVNTFGTV
LTASAILISTGFFMWNAGDVTVQASRLATAMYCSGWQNCRGKSSVSIRNMVMNTIAVAQR
PLVLRGLGVIDLSYQSYLSIVKASYTVFSVIY
>BmorOR21
MNKNHYILKTYCDKIFLVGSGNFWYQKTESRNDKTLLYKIYSCVLFFTYGFMTVLEIMAA
MMGDFPEDEKRDSVTFATSHTVVMIKFISIIKNKELLKTLNRKMMMICEAHEEQTLMDEM
YRTVKINVVAYCVAVYGSATFYVFEGLRKFYNGSHFVTIVTYYPSNDDDTLAATIVRIATTL
VLLMMLLTMIISVDTYTMAYLIMYKYKFITLRHYFKRLRENVDELVAAGKARLAAEKLA
QGLVEGIKMHNELLSLSKDIDKAFGTVMALQLCQSSGSAVSLLLQIALSDQLTFTMGMKIF
FFLAAMYLLLALFLCNAGEITYQVCTSIV
>BmorOR22
MNKNHYILKTYCDKIFLVGSGNFWHQKTESRNDKTLLYKIYSCVLFFTYGFMTVLEIMAA
TMGDFPEDEKRDSVTFATSHTVVMIKFISIIKNKELLKTLNRKMMMICEAHEEQTLMDEM
YRTVKINVVAYCVAVYGSATFYVFEGLRKFYNGSHFVTIVTYYPSNDDDTMLASIVRIATT
LVLLMMLLSMIISVDTYTMAYLIMYKYKFITLRHYFKRLRENVDELVAAGKARLAAEKLA
QGLVEGIKMHNELLSLSKDIHKAFGTVMALQLCQSSGSAVSLLLQIALSDQLTFTMGMKIF
FFLAAMYLLLALFLCNAGEITYQASLLSDEIFYCGWHKCNSPVLSTQRNIRDIVLIAILRAQ

SPLVMKAFKMVELTYATFILVVRSTYSVFALFYAQNK
>BmorOR23
MSLSGSSVFRHLFLLRCCGFCRLSRSSTARRGLSVAHEVYRALTLTLTVVYLLQECIYAYQE
RTDMDKLSRVMFLLLCHITSVAKQLVFYLDADRIDCLIATLDDPSYNEMSHQRLLVDASR
WASRFVWAYSGCAVVTCTLWIVFPIIYHVQGQTVEFPFWIQIDYTKSSMFVVVLLYSYYVT
TLVGIANTTMDAFMATILGQCKTQFTILRIKFETLPTRAKQALRCDSEQNYDEVLMRLFHD
CLKHYQKIVDTTSSLQSIFSSAILIQFGIGAWILCMAAYKIVNLSVLSIEFASMILFISCILTELF
LYCYYGNEVSTESDRLVTSIYSMEWVGARLGFQRGLLVLLERARRPVRPAAGLVIPLSLQT
FLKIIKSSYTFYAVLRQTK
>BmorOR24
MPEELFLDRSIKKIESYFRWMGINIRSGDNNNKKDVFKIRCIYFINFVLLNTDVLGAIFWFR
SGLEQGKTFTEVTYNAPCLTFSFLANFKMLSLIFYEKTVHELIAALQKLEIKHFLRQNCAEE
LKMLKDEKNFLHAVFKGSKIVNYASILTFGCSPLVLIASNYYKTGRMDYLLPLIVLYPFDV
DNITVWPIIYVRQIWSVITAVIGVCATDYLFYTFCVYISTQFRLLGHSIERVVPNNGLSVRTR
LNGNLRMKFVENLKWHQELIRAASLLEQIYTKSTLYNFVTSSVIICLTGFNVAVVEDFAVIL
SFLFFLFMSLLQIILLCFFGDKLMKSSTNISDAVYNSKWYLTEKNVGKVLLMVQIRSQRAC
RLTAYGFAEVNLRAFMKILSTAWSYFALLQSLYSSHE
>BmorOR25
MFEKALRSANFYMRVIGIPTDIRDGNRTLMERLRNRWFYCINFLWLNTDVAGEITWFVKG
LLSGSSTLIENTYLIPCLTLCILGNVKTFFTIKYANHIIDLVAILKDLEIKNNAARKNETEIVKE
RLKFLTTSNKFLLFVIGTGIIAFGIGPLMLTASIYFSSGDMKLKLPFLIWYPFDSSDIRYWPFV
YVHQVWSACIACCAVYGPDCFYFTSCTFIHIHFIHLQNDITNVIVESSRARRNGLYRGCHQ
AFLELTNRHKDLIRCVNLLEIIYSKSTLVNVVSSSLLICVTGFNVMAIDFLPLIAPFTSFLALG
LVQTYLLCYYGDTIMCSSTEVSDAVYNSTWYGTNISQMRDYLFVMKRAQKPCKLTAYGF
SDVNLRTFSRILSTAWSYFALLITIYRGNGQQ
>BmorOR26
MSNYVFKPFHETYRIITFTMIAAMIYPNPATEKRRLIYIGLMLLSVIPLAFMIVTEMYEFFM
ASDLNNTIRHSTVIGPFIGGFVKVALMYYKRRQANELVSEINRDHLAYNGLKGEDREIAAS
SIRNCQIYCELGWTLIVMSCGLSFPVIAILLNIHSFTFKFDSTKHMIHDINNPFTDDPEDRFES
PFFEIMFVYTFFSSFIYIINYVGYDGFFGLCINHACLKMKLYCRALEDAMRSESRRHEKIVA
VIEEQRRTYEYIALIQDTFNIWLGLIYVATMIQMCTCMYHIVQSFNIDVRYIIFVISIIHIYLPC
RYAANLKCMAAETPTLIYCCGWESVSDLRIKRMMPFMVARSQVIVEITAFNMFAFDMELF
VWIMKTSYSMFTLMRS
>BmorOR27
MPSSFFLPNLENPDYPSLGPTLKGLKYWGMWQSGGIKRILYNSIHAFATFFVITQYVELWII
RNNVELALRNLSVTMLSTVCVVKAGTFVCWQKYWSGIIGFVSNLEKEQLSKNDAATQAA
IVKYIKYSRRVTYFYWSLVTATVFTVILAPLVGFLSSPERELIANGTLPYPEIMSSWVPFDRS
RGFGYWVTALVHTLICFYGGGVVANYDSNAVVLMSFFAGQMKLLSINCSRLFDDGNEVIS
NNEAMKRIKECHYHHVFSTIFNSLMSPVLFLYVIICSLMLCASAVQLTTDGTSNMQRIWISE
YLMALIAQLFLYCWHSNQVLYMALEDRLGGLFEACLESGRFPSKWKTGRLVLLRKDGRP
ADSPAGYRPIVLLDEAGKMLERIVAARIVRHLTETAPDLSAE
>BmorOR28
MHTLALVFALLYPSNCNIIKRAIGITLIIALSGGQLFWCMTYTFNVCVLILNYSGFDGSFCIA
SIRLCMKLKLVVYKVQKAFAESKSVSELKHQLNDAIKDNLDALKFHEQIQNVFFIALVGR

RAYGPPDGEWLPSPMDFSNTRGRTKPLSTVYEPWLFLIFLLTFLII
>BmorOR29
MFDFLQNLEDSERPLLGPNFWLINKTGLLLPKTNFGKLAYILVHEIVTFFVVTQYVELYVIR
SDLDLVLTNLKISMLSIVCIVKVNTFVFWQTSWREVLEYVNEADKFERNQTDETRGKMIE
TYTKYCRRLTYFYWSLVFTTFLTTTNTPLMRYWSSPIFRENLRNGTEDFPHIFSSWMPFDK
NHSPGSYCTIVWHVLLCAYGAAIMAAYDTCIVVIMVFFGEKLNLLRERCKKMLANDLYN
HAFVIGQLHDIHVQLIKQSRLFNSLLSPVMFLYILMCSLMLCASAYQLTSATSTAQKLLMA
EYLIFGIAQLFVFCWHGNDVLFKNANVSLGPYESNWWSSSPRVRADVLLLCGQLRVRHV
FTAGPFADLTLSTFIKILKGAYSYYTLLRK
>BmorOR30
MSVSNLKFEVLFKPTTMSLHMNRSHPSIKRNKIWLLQFISLMTLTAFCATGLITSLLFHDLK
FGKYMEASKNGTIAMLSFTTTFKYSLLLYLQKSLNRLIAKIDMDYEIAKGLTPQEKVTVLN
YAKKGVIVSKFWLFTAFAITFCFPLKAFIIMGYRFIIKDEFRLEPMFDMTYPEPIESYKTSFPV
YFILFVVCFLFGCYASSLYVAFDPLVPIFVLHACGQLDLLSVRITKLFSDTKNPRIIAKELKVI
IIKLQELYSFVNFIKVNFSILYEYNMKITTISMPLSAFQVVESLRRGEFNIEFTYFFFGCILHFF
MPCYYSNLLMERSENFRFAIYSCGWENHNDKNIRQMLLFMLTRAAEPLGIATVFTNISLDT
FAEVNTFDTVLLA
>BmorOR32
MTTRHAEPCAEAPRLSPAAGGMVGLAPVPQPSSNEMLVQERGRPPGLEGEYVANRPFRKS
LRGSQEEPRANGKSENVRFLINSHILHCGLRFNETNCHTHYIAKVAIFCFIVTYMLQVMEL
YWSKGDQEKLFECFSILSFCGMGVMKLVILRVYHQRWRFLLNQVSILENRHLDPGPLSYD
SDNDNDDNEIVTFITKYTDKFKRTSSILIKMYASTLVIYVLSPFVEYIFRQFRGDLNIAYPHIL
PAWTPLDEFSVTGYLIMVSFETVACIYCVFVHVAFDLTCVGLMIFACGQFYLLRYRSERIGG
KGRICRLLKSTEVRAHYRIVFCHGIHVLLVQLIEELDRLIKHILGVYFFLATLTLCSVAVRLK
TEDMSITQLVNLLQYMCGTLTQLFLYCKYGDSVYNEADEPYGLPDACLESGRFPKQWKT
GRLVLLRKERRPADSPAGYRPIVLLDEAGKLLERVVAARIVQHLTGVGPDLSAEQFGFREG
RSTIDAVMRVRALSDEAVGRGGVALAVSLDIANAFNTLSWSVIAGALQYHGVPAYLRRLIG
SYLEDRSVVCTGHGGTVLRFPVQRGVPQGSVL
>BmorOR33
MELNFDKIFKIAIISQKFSGTYPYTKRDKKWATHFILMHGELTIICMLFIYNIIEFDLKAADY
SQMCRNMCLSFVYLVITLLYINMLYYQSKLKMLIETMKAEYELAKTMSEEEQNVILEYAK
KGRWLCRAWAILTTCGMAQFFLKSIVCTIYSAIQGNFRIVQYYEVICPEVIERHRNNPVIFIT
LYFCTFFYSLYTSALYTSVLPLGPIFLLHGCAKLEIVRLNIKNLFDNDDYVVQERLKKTVLQ
MQDIYCYSHEINECFQILYEFLLKATSLVLPITIFAVIQALGRGQFIPEFFAFIFGAFMVGTTPC
YYSNMLMEK
>BmorOR34
MELNFDKIFRIAIISQKFSGTYPYTKRDKKWATHFILMHGELTIICMLFIYNIIEFDLKAANYS
QMCRNMCLSFLYMVITLLYINMLYYQSKLKMLIETMKAEYEIAKTMSEEEQNVILEYAKK
GRWLCRAWAILTTCGMAQFFLKSIVCTIYSAIQGNFRIVQYYEVICPEVIERHRNNPVIFITM
YFCTFFYSLYTSALYTSVLPLGPIFLLHGCAKLEIVRLNIKNLFDNDDYVVQERLKKTVLQ
MQDIYW
>BmorOR35
MVVLSLGISSEIGTLKFFYTFIYIKKVQRIVREYLECDHMVVPGSRFADNVLKTMRNVKKR
AILYWVVVIGNGVVYVTKPLFMSGRHHMEDRYIVYGLEPMFESPNYEVAYFLMMFGLCFI

CYPPANVTVFLIVVVGYTEAQMIALGEEMLRIWEDAVAHYNNKYHTVGALTNSSEKNKII
NQYVKFRLTEIIKMHTTNIQLLRQVEFVFRSAIAMGYVFLVLGLIAELLGGLENTYLQIPFA
LIQVLVDCYTGQKVMDASSLFEQAVYDCKWENFDKSNMKTVLLILQNSQKSMRLSVGGI
TVLGFSCMMSVMKSIYSAYATLRTTMS
>BmorOR36
MVFNSKKNIISLFSLLEDSRHPSVGPHLRLLSLTGIWYPNSKTNITLLKRACFYVIVLFFVSQ
YLKCIIKFKIDSLQLILEYAPFHMGIVKTCFFQKDYNVWQDLVSFISKTERDQIAKKDPKSIK
TIQSYISRNRKITYSFWALAFIANIGVFSKPYQNNQSDVNGTVTYNHLFDGYTPFSEEPPGY
YFSMGIETILGHVVSFYVLGWDTLVVSIMIFFAGQMQMSRLQCSRMINGSPERTHKNIIKC
HKFHTDLIKYQKQFNSLISPVMFVYLFVSSINLSVCIVQIAEIEDDFATVLSSFIFLLACLIQL
LLFYWHSNEVTVQSELVSYSTFESNWTSTQNKLQKEVALLGLTTSKTLVFTAGSFNHMTL
ATFISIIRASYSFYALLNSTKY
>BmorOR37
MELGCSRHLKLPCSLHPIGISKHGNTLSELLIYFPAIPKITYAILAVLLTVYYYIYLCSITWFV
FVRCPQTGDLAAASIVFSLGVSSEIGAIKLFIMYVYRAKLRDITGEYLQCEADMAPGRLRA
RVGRSLRTVRRRAFVYWLVLVVNAFAYDLMPAFLPGRHLSEDVFVIYGFEPMFESPNFEIA
STLMGVSVVFICYTAGSISAFLIVIVGYSEATMLALSDEISCVWDDACASECQQPNDFIRAR
LGKIVAIHTKQIRLIREVEVVFRGALAGGFACVAFGLIAALLGGLENTFLQLPFCVIQISVDC
FVGQRLRDANVAFETAVYNCKWEYFDKSNMKTVLLILQNSQKTMGLTAGGVAALDFTSL
MTIFKSVY
>BmorOR38
MVVFSLGISSEIGSTKFFNTIIYIKELRKLFKDYLLYDATCPAQGRLRLHLLTTLRYVKRRAII
YWLVIIGNGFIFAIKPLLVEGRHLAQDDLVLIGLEPMRQSPNYEIAYAIMTMGVCFICYPPAH
VTMFLIIIVGYTEAQMLALSEELKHLWNDAIEHYEKHSRTEREADAAMKSKILNSFVNFRL
VQIIKSHSTNVNLIGRVENVFRGSLAVGYVFLIVGLIAELLGGLENTYLQVPFALIQVAIDCF
IGQRVNDANIDFEKAVYDCKWENFDKRNMKIVLLLLQNAQKTVSLSAGGIAKLNFSCFMS
VIKSIYSAYTTLRTTMK
>BmorOR39
MLWSVFSYFTRADDVLAGIVIFSLGVSSEIGLVKLCFMYANIDKIQKITEGYLKSDAASAR
NSRFSKNILHTMQSVKKRGVIFWLVIISNGVVYLVKPIVTPGRHFMEDQFIILGLEPKYETP
NYEIGFFMMAVGVCVTCYLPANITAYLITVAGYSEAQFLALGHELANLWPDAQLHCRAM
NLSQSVNEQANEYVKMRLRELVKIHSTNVNLLRDIEGAFRGAIAVEFLLLIVGLIAELLGG
LENTYMQVPFALIQVSVDCLTGQRVMDANLALERAVYDCRWEEFDASNRRVVLLLLQNA
QKVATLSAGGIATLNFSCLMAVIKSIYSAYTTLRTTMK
>BmorOR40
MTGAGAGTFRTGAGPGRGDGVARRGESGETTTLGRDAFAALGCFGAADGSTARARFFPR
VTVLNPSEVPGSGLAADSNSISDSESEPELDAAQDAIDAGAGVGGDIGESRARTVFGIQGH
DASDSALRMHNNVAIYAKTTMSGNSQLTFATAATIFLKNASGPNGVAIGTDYAICVVSLSLF
FCYRFTELVEDTYNSYLLFQLVGSVGIICMSALRILVVDWRSVQFFSILCYLSVMISQLFVC
CWCGHELSATSEELHTILYNCAWYDKDVKFKRDLIFMMARARRPILLRAGYYIGLSRQSF
VSVSIPRIRFNAILVI
>BmorOR41
MMGNSTDLFLDRTKRILNFFAMWRSFEKPIPLKVYMAFIMTTQYLFLIFEIIYIVNVWGDM
AEVSEASILLFTQASVCYKMTAFISKTNNFVILLGLIESEIFSAQTELHEKILILKARKIKRLC

MFFLVNAVTTCSLWAVIPLLDISSKMLPFKIWMPVSTGESPHYELGYLYQMITIYISAXLFIS
VDSVPLSMIMFGCAQLEIIMDKIGKVKSWSLDQQPMQKQEVLNSNYELLVECVRRYQSV
VRFIELTEKTYHANIFFQLSGSVFIICNIGFRIAIVDSNSLQFYSMLTYLVTMLSQLFQYCWC
GHELTIRGEELRETLYQSPWHEQDIRFRKVLIITMERMKRPIIFKAGHYIPLSRPTFVAILRCS
YSYFAVLNRVRNE
>BmorOR42
MDIPKFEELLKQIKMNFWLMGIPFDNPKIQIRYYVLLLPLSLMLIEEIAFFGSRMSSENFLEL
TQLAPCICIGVLSVLKILALTAKRQKIYELTQNLECLHKIILNDTRKTELVRKNLVLIKFITKY
FFVLNAVLIFVYNFSSPVIIAYNYIVSNEVQFVLPYAVLLPFKTDSWIPWLIVYVYSIFCGFTC
VLYYATVDVLYCVMTSLVCNNFSLISFKLQKVNRNTAHLLKEVVKEQQYVLKLAEDLENI
FTAPNLFNVLIGSVEICALGFNLMIGDLTQIPGCILFLSSVLLQILIMSVFGENLISESSRIAEA
AFLCKWYEMDQKSKKTILTIMIRSHKPKKLTAYKFSVISYGSFSKIISTSWSYFTILRTMYTP
PGTKFQDDL
>BmorOR44
MYTYFKVLVFWLNKDKVISLQKILHCKEFKPKEPEHKEIIRKSIRKARFVMTSYATMCVGA
VSVGIILPLTENFDILPTNVEYPFFDVYKNPTYAYLYLHHIYYKPATCIIDGVMDTILAAFVA
SAIGQIEILAFNLRNFDVLAERRRKRAISGNKYIGKYTNLYFTKRILKECILLHNSIIRYVSVI
ESAFSLASALQFMLSVMVLCLIGIQFLSIENPTSHPMQMVWMAIYLTCMLIEVFILCWFGN
ELIWKSNDLRQAAFDGPWRNLNRKTCMFIIIFMERCKRPMRLSAGKIFTLSLDTYTVLINW
AYKAFAVMRNMKK
>BmorOR45
MKVLDNVNHAVKVTMNCCRLYGLFVSDDLTKRQLIIMRAFSLMLYLFFVGFFITTQSALII
TMWGDLNLMTNVGLVLGTHLTLSAKVFTLHYKEKEITNVIYKNEVRLRAETREQGKYIIS
EYPCNTTKSPAHEIILAHQGIAVILTATLEIAIVLLMTSIVAVCRCRLKLVGLSFETICDDLPSN
IMNKLTADEQVIVAKRVRENVIEHQAVLECINDIQDCFSSAMLVHIAISTMIICATAYQLAVE
KSLDLTQRMTMASFLGGMSTEIFLFCYQGGHLSIDSMEVATAVYSCPWYTFPTSLKRSLLVI
MIRAQQPALLTAGGFAPLLLDTFVSIMKASYSFFTVLQNASE
>BmorOR46
MAFFIRNKMLGLTITLNTLSWAGLIMRDQYTKTQRIIVRVYGWLVFLYLFVAATYVQIADLI
DIWGDLDLMAETSLLLFMELAVISKILTLIFKYDKIMEIINGTEDILCSENRLEGQKIIASIDK
ETTRFFQYYTSSVIFTTFFWFLGEHSSTFFIRAKYPFNELKSPGYEFALIHQCMMMVFTGYF
EFNINIFFASVVAGCRCRLKLVALSLRNICINIPVNKKNLITPEEEKLITERLHCAISQHKYAL
DAAEDVKHCLSKVLLVQLTVSIVIICTTAYQMAVNKSTDTIQKLSMAGYLLGASFEVFLFC
FQGQSLSNASEDIADAVYECPWYTLTQPLKRTLLIIMMRAQSPAILTAGGFVTLDITEYMAV
LTGHGGFGDFLHRTGAEPMAECHHCGCDLDTVQHTLLVCPAWKGWRRDLVVKIGNDLSS
VLWHRCSAATSRGRRCLTSASAPSRRRRRGA
>BmorOR47
MKLVFDNFISALKVTLNWSRYIGIFIPDELTGRRQKLLVQAYSVFMYYLFIGFFITTQIISFIL
VWGDLNLMTDVGLVLGTNLALSAKIAVFFFKREDLANILKKNDDTLRSETRAEGKKIISET
SCESARVGTTTLPISAVVTILETLELISQASLLITVDIIMLSMIAVCRCRVKLVGLSLQTICDD
LPCNVKNKLTSDEEVIVAKRIREYVIEHQAILDCISELQNHFSPALLVQLLTSVVIICVTAYQL
AVEKSSDLLRKFTMASFLFAMSTEMFTFGYQGGHLSHDSMEVATAAYSCPWYTFPTSLKR
SLLVIMIRAQQPALLTAGGFTTLSLETFVTIMKASYSFFTVLQEATD
>BmorOR49

MLTCFATIFSAVNQTGYIVLFINLLAHELGHFYVITDVLNGIFEKNDADRDPVFIDRKLKFC
AKHYQYLLKFHNEIKNLYKIIFGAHFLMMTIVLVTTLQTMNSWDIRNTVLTAVTGIMPLFI
YCFGGELLITAGMDMSTAIYQCGWEKMGVKQAKVVSVILCLSQRPLCLTAANVFVMNRE
TFGGIAQVVYKIYAVFN
>BmorOR50
PIFAAKTLKDKQHLIQNKKEVTRFARLLLTYVTVGGFIWPMSFCFRRIKDPNTVVPFYVPF
TPDNWTKLINEVTDIFNPCLTFQFFTSSVAICMVIYKLSDTYIVSLEFVFLLNFIFVLLTQMFI
YCYYGNVVSYESKYINTSLYLSDWSSASPGVRKMFLIVMPRWTRPLVVRIARVVPLSLDSF
VSVRKYKCSIEHFIYSHVKQTRARDIFSGASNKP
>BmorOR51
MDCTIVAFYSQAKTQIKMLRYDLEQLGKIDNIETKFTENIFERSSHIWKALKDEKIKIHSKL
VFCVEHYRQIVWFVKEVESIFGEAMTVQFFVMAWVICMTVYKIVGLSIYSAEFVSMGVY
LGCMLAQLFIYCYYGTQLKVESESVNTSLYCSNWLSSLPKVRRQMLIMMQYCSKPLTPRT
AYVIPMSLETYISVLKSSYSLFTLLNQKH
>BmorOR53
MALKKMLALTKGLEDPTHPLLGPTLKALSVFGLWQTGSQKSTVIYNTFHFLTFLFVITEYI
DLYTVRKELSKMLNNLSVTVLSTICMIKTLSYVCRQSHLKVLVREISELELELMKTTDKNI
VKRLRQYTVYTRAVTYVYWFLVVGINVVLLTSPLLKYASSEIYRSEIKNGTEPPPLILCSWF
PFDSARMPGYFWATMVHIIMSIQGCGVVATYDMNAVAVMSYLKGQTSILKDKCKAIFDET
ASSRDVLNRIRDCHRHHNILLRHYYMFNSLLSPIMFVYMLICSFTICCSIIQLDSSETTISQRI
WIIQYSIGQISQLFLYCWHSNEFAAKVKKKHFPLFPINLF
>BmorOR54
MGLNTIKEFFVNVKRRFQDVSIDSLLWIVNIVPSLAGFSIRSDRVSAPFWIVHWSLLVYVYA
VGNAVYQWKFANEAIDYITSFINVSLLILIGNNSWWFLANRRLLKSVLHKIEVNDELSRRS
EQSRLKHKKLLKIIKRIVLVFYMSNYVNASFIYLPNRVDVLNNYAMTPCVGMEPLTVSPNR
ELCLTILCMQEFSIMTVVLNFQALLLCFIAHTAVMFQILADEIMALNNYENLEEHQAYVKE
MLPIFVKRHSLTLSAVDNYKSLYSVPLGVNFGSNALTILLILYLPVLEWFKFIPIFVFCFMLFF
LYCFLCQKLVNASEAFETAIYCCGWENFALREMKMIYVMLHQAQKPVELLAADIVPVNM
NTFATTLQAMYKFVTVVKF
>BmorOR55
MCFLKIKQQIIDIQKHFKDYSLNGSLWIVNLLPRLMGFNLRADKVGVFFWTIYILLLVYVF
GIGIFVYLWKHVDTMSGLMKSYLNLSLILVIVNNSCWFLSKRSLLNKVLKKIHLIEDLSCES
EHALAKYRRVFKIVTHLLLASYVLFYFTEIYFMFLFRNYDLLEDYSLAPCVGLEPLSSSPNS
EICLIIVLIHEFISTTVMMSFAALFLVLIAHTAVMFLVLAEDMTKLTDLINLADHRKMIRESL
RSLIHRHSLLLQIVYELRLLYSVPLGINFISNAMSILVLLCLPIHEWPSFLHIIGYCFFAFFLYC
FLGQNVINASEKFIDAIYCCGWEHFGVAEKKLVHVMLRQAQKPVEIIALGMISVNMNTYV
EALQLIYKFVTVLKI
>BmorOR56
MKLLEKLEDPDRPLLGPNVKALKFWGLLLPESRSKKYFYLFMHFAVTVFTATEYIDVWFV
KSDLALLLNNLKITMLATVSVLKVTTFLLWQNAWRDLIGYVSRADLEQRATSDSRKLALI
NGFTGYCRKITYYYWFLMYTTVAIVTVQPIFKFFSSAAYRLDVQSGNGTYLQVVSSWIPW
DKNTLPGYLLASIYQTYAAIYGGGWITSFDTNAIVIMVFFRAELELLRIDCAALFDDEKSFG
DMAFMRRLKECHRRHTELVKHSRLFDSCLSPIMLLYMFVCSVMLCVTAYQITIETNPMER
FLMTEYLVFGVAQLFMYCWHSNDVLYASQDLSRGPYESAWWSRDVKYRKNLYILVAQFN

KVIVFSAGPFTKLTVATFIRILKGAYSYYTLLSQSQMNKT
>BmorOR57
MPSLIKNRIFGLTLTLNTLSWAGLILRDDYTKTQRIIMKVYGGLVFLYLFVFTAYVQIADLV
VIWGNIDFMTETSLILFMQLAVSAKVLTLMLKSKKIMEVTNEADAILISEKKVEGQRIIASI
DKNTTLFLKYYGFFVAFTIICWFMGENTSTFFIRSKYPFNELKSPGREFAFVHQCIVVIFTGS
FDFNVDIIIISLVAVCRCRLKLVALSLRNLCLDIPMNKRNLITSDEEKVITERLRNIISQHKRA
LDAAEAIKHYLSGALLVQLMVSIVVICTTAYQLAVKKSTTMQSLTMAGYLFGTSLEVFLFC
YQGEFLRESSEEIADAAYECPWYTLTRPLKKTLLIIMTRAQRPATLTAGGFVTLDITEYMAV
SLISNT
>BmorOR58
MKLVFDNFIFALKVTLNWCRYFGIFIPDELTGRRQKLLVQAYSVFMFMLFIGFFIITQIILFIL
VWGDLSLMTDVGLVLGTNLALSAKIAVFFFKREELASILKKNDDTLRFETREEGKKIISEY
PCDTKRSPAYEIIMIHQTIAVAVIASLAITADLLMLSMIAVCRCRVKLVGLYLQTICDDLPCN
VKNKLTSDEEVIVAKRIREYVIEHQAVLDCISELQNHFSPALLVQLLTSVVIICVTAYQLAVE
KSSDMLRKFTMASFLFGMSTEMFMFGYQGGHLSHDSMEVATAAYSCPWYTFPTSLKRSL
LVIMIRAQQPALLTAGGFTTLSLETFVTVS
>BmorOR59
MDTNPSAAGDSVAPHLRRLRQVGFCQLDPTSQSRRPILALMHRVYHRLVLAATVLYIFEQL
TYAYQARNDMERLSRVLFLMLCHLTCIAKQFVFHSDADKINQLVVGLDDALCNQPVETH
RLLLLETSRRAARLLMLYSGCAVSTCILWAVFPLLDQLRGRTVEFAFWIPIDYRHNAFQFAV
VLAYAFYSTSLVAVANTTMDAFIATVLYQCTTQLRILRMNFESLPERAYALSRKTRQDYHT
VTHELLVDCLLHYKKITETCNLLEQIFGKAILVQFGVGGWILCMAAYQIVDMEILSIEFAST
ALFMGCILTELFLYCYYGNEVTVQSGLVSESVYAMSWLSLCPRERRALVVVLERARRPLR
PAAGRVVPLTLNTYLKILKSSYSFYAVLRQTK
>BmorOR60
MVRPCRYFAIHFILLRFLGLGWWHHPHENETRNYPGLYLYYSILTQLVWVVGLVGLETIDP
FVGEKDMDRFMFSLSFVITHDLTLIKLYIFYFRNVEIQDIVRTIEIDLYRYYQNDDKIRATIRI
SRIFTAAFLFFGWVTIGNANIYGIVQDLRWKDIVKNLNETTSKPLRTLPQPIFIPWPYQEDK
HYILTFILETMGLLWTGHIVMTIDTFIASVILHMSTQFAILREAIVTAYDRTMIALSEGALQS
GVLCENSNGNEENNQIFLESFYSKEHIESVLESTLLSCIRQHQLLIGCVEKFSKTYSYGFMT
QLLSSMAGICVVMVQVSQGASSFKSVRLVTSLAFFFAMVIQLAIQCFTGNELTIQAERIAD
AVMESKWEKMPVRLRRLLLVTMMRAQRPLHLTAAGFAYIDNTCFLSILKAAYSYYAVLSQ
KQG
>BmorOR61
MARITDVFRLNFIFWKFLGIWGKSAPSKYNMAYTALYLSASLFVYDIFLTLNLIHTPRKLET
LLRETMFYFNHLVAMTKILKMFIRRKKILVIFDLLDCEEFKPSDEDSQEIMKRKNEFYYIYW
RIVAVTSNLSCFMQVVGPLIKMLIWKSELGLPVCKYYFMSDEFRNKYFVIWYIYQSFGIYN
QMVNNLNLDTFNCGMLWMAVGQLQILKTKFVNFKLNDIENSLDLKTRDDMQTERLRKY
LTHYEIILKYCATVQDILNITIFVQLGMSSIVICVGLCGFVAMPSNTETAIFMSSYLITMTMQI
FVPSWMGTQISFECGELMSAAYCCEWIPRSKLFKRSLILFVERAKTPVRITGLKIFTLSLDTF
TSIMKTTYSFFTLIRQLQVDEVN
>BmorOR62
QNVILEYAKKGRWLCRAWAILTTCGMAQFFLKSIIVCTIYSAIQGNFRIVQYYEVIYPEVIER
HRNNPVIFITMYFCTFFYSLYTSALYTSVLPLGPIFLLHGCAKLEIVRLNIKNLFDNDDYVV

QERLKKTVLQMQEIYCYSNEINECFQVIYEFLLKSSSLVLPITIFAVIQVSSLHICFIFLIPNTQ
WNVCSQNKHFLGSLEMSCRNCHSK
>BmorOR63
MKLWIRNANFTISLSLTLLRCLGFWSPDGLAGNKRLLYNCYSFVFFMFLLGIYILIQVVDMI
KIWGDLPLMTGTAFLLFTNFAHATKVINIVIRKNRIQRVIQQANAVLMGVQSEEARRIVKSC
DFETSIQLCLYFLLTFVTTVGWATSAEKHQLPLRAWYPYDTSKSPAYELTYIHQVAALLIAA
YINVAKDSLVSSLIAQCRCRLRLVGLALASLGQDLKIDYQSQLSPAQENILNLRLKTCVLEH
QTVLAAVTELQACFSKPTFAQFTVSLIIICVTAFQLVSQTGNLVRLLSMGTYLMNMIFQVFI
YCYQGNKLSVESSEIAGSVYFSPWYLGSVKLRRALLIVMVRSRRVAKLTAGGFTTLSLASF
MAIIKASYSLFTLLQQVKQKK
>BmorOR64
MGVSNGRGTVKPFLYPLVDELDYNLIVGVHLPFEYKTPSRYPLAYITVVIAFIYVSYFVMV
TDLIMQAHLLHLLCQFNVLADCFENMLNDCVKGFEGPLVSLHEYIHPLIDEFEYNLMVGL
RLPFSFDTPLRYLFTYVIVLIAFNYTAHYVMVTDLIMQSYLIPLICQYAVLADCFENILIDCS
NDYGDHARRNDIVYSRSMELRAILSRPMLGQLASSGLLICFVGYQATTSISVNIVKCLMSL
FYLGYNMFTLFVVCRWCEEITNKSLNIGNAVYCSGWESGMTVVPTVRSTILLVILRANKPI
VFTAGGMYNLSLTSYTSLVKGSYSALTFLLRIQHE
>BmorOR65
MRLGFEVSISEYLYRNIFYIYTLFHILLHFYYILHMIKLDLEAIFDDIDESVALLPHRDTRRIE
VQKILNGRMKRVVTWHISVFKAVEAVSSIYGPPLAYQVMFTSIAICLIAIQITQKLENGILDI
RFTMLGVAACLQMWIPCYLGTLLRNKAFGVGEACWNSGWHQTPLGRMIRQDIIIVLLRA
QQPVTIKFPGLQSIQLETFSSVIFNLYGYYYFLLLRWVDELTAHLVLSGYWSP
>BmorOR66
MRFGLKVYIYTLFHILLHFYYILHMIKFDLEAIFDDIDESVALLPHRDTRRIEVQKILNGRM
KRIVTWHISVFKAVEAVSSIYGPPLAYQVMFTSIAICLIAIQITQKLENGILDIRFTMLGVAAC
LQMWIPCYLGTLLRNKAFGVGEACWNSGWHQTPLGRMIRQDIIIVLLRAQQPVTIKFPGL
QSIQLETFSSVIFNLYGYYYYYCLDG
>BmorOR67
MRFGLKGGAAVVTILETLELISQGGFIETIQVTFGGQLSSMLFISACIICSTAVQILAIESPLDN
LTTVGWILVYLSLCILILFVDCYFGNTITVKCAYLPTAVFSIPWLDQPKNIQVSTLLFMAKTQ
QPVQLIAAKLVPVSLTTFTQVSYCPPLDLKCLQGGVIAHLAKD
>BmorOR68
MFTIDFHDERITSFNKNQTRKIIICVITGGRTSCESARVGTTTLPISASEDVCMTLYSCGWET
RFDLNTRKCIILMLCRALRPVSIRTIFRSVSLTTLTGVFQQAYALFNLLNAVWN
>BmorOR69
PTEITMEQAKSEIDGSLVLSSFCMKRIGLSFESPKEFSSLLRQKIMFLLSFSGICYHVFSEIVY
IGLTLTNSPRVEDVVPLFHTFGYGALSIAKVLSLWYKKDIFSQLLKELSEIWPTIPLDEDAQS
IKTRSLSALHVTQRWYFATNVLGVWFYNVTPIVKYMYRWLKEGDGQIGFVWVSWYPFD
KNGTVAHIAVYIFEIFAGLCQTCVWIMVSTDLLFSGMASHISMLLKLLQRRLQCFGTTPQT
DDVNYQEIIAIIKLHQRLIRFGYILVEWCREPGIAKHVKLLEPLPALSSKLFLVSALIQIGMLC
WYADDIFHANGDVATAAFNSGWYRTSPKCRRSLLFLIRRAQKPIAFTAMNFTNISLATYSAI
LTRSYSYFALLYTMYREN
>BmorOR70
MSESDQKFARMEILETLKLCNFCIRRVGLSFIDEVPLTVLAKLKKYFLFIFSSSMLVLMLAG

EVAYVAGQMINSASIEEFVGSYLHIAGYDTMSFGKLITIWYKRNTFKQLVEELAVIWPVSIK
DEVAAQIKRDSLSALKLRQFLYAFWNVLGVWLYNLTPIAILLYRKMRGTPGELGFVWQLL
YPFDKTQPIFHEIVYVFETFSGVISVCCMLGSDLLFMTMSSHISMLLRLLQVQIRRLATTEG
RNYESDNDCYDEIISVVKIHQRLIFSGNALYINDLENAFSVVNLINVLLSSFLQPWMEMSD
KFFLGAALTQMGIVCWYADDIYRASVGVSDAVYESGWYRTNTRCRRALLVVLQRSQKPL
FFTALKFRSITLTTYSSILTTAYSYFTLLYTSYRRN

>BmorOR71

VYKVEDYNATFTIPMAVLKLVGLRVTKNDSKYTRLFWNIFYWFEFGNLFIVTWLELINMV
QTASGGSFQDAVEIFRMMPCVGYLLLAMAKSYKIVYYRPIYENLIYELRDMWPKGCVAD
EEHAIICTALKQLNYVVQGTLTIALAIIFLSPPFVEIIKQVAGWDVPLILPFFYWFPFDPFQRI
YYETILIFQTWHDKLPLTFPGLITIWFMLCGDLLFCIFLSHISTQFDLLAVRIKRMVYVPVDK
QLIEEYPLGEYSRNFLVKEKSAIDSYTEKQWEERFQNEVTEIVLRHRALIRLSGDVEELFSF
ALLVNFFNSSIIICFCGFCCVIVEKWNEMIYKSFLTTALSQTWLLCWYGQRLLESSEGVSDA
LYNSGWYNVSKKIKGSILIMLHRAQKEVHVTTYGFSVISLSSYTTIIKTSWSYFTLLLN

>BmorOR72

MEELRGLPEDYLEPLLPCLDLIKRCNVTFFDNGTLFNRYWRYFYIIPCVVAFYIFLSAYMM
QVFEGQLELSELAYVVSVYVVSNQAIVKAIIVVLKSKEIRSIILELGQAWRTEDLSDAQINK
KKILLKRLNFVSKVFLIVYCYVGVELLMVNLCAHISTEFALLHEDLRNLTPLRNSKGKGLG
NGIRKIVRHHVKLIRLSQQLDNIFNIMIFINLSSVTFTICFFGFAAKVARTAIEKAKNFIGVIAL
IVQIFNLCYYAELLQDASSTIADAAYENLWYNGGIDHQKNLLFIIKRSQNPCSLTSMKYSPI
ALNTFTAVTLGILYSYIAWMPQFTLA

>BmorOR73YTTLEAFKPHFNALARVFVAFTDICKVFTKTVKKSLRCFRAMPITLYVGGEYR
GRITFNIQVRRSTERIVDILFILLTTLNTFGKQLAFNLRVHRIDKIIDVMNGVYFAATNRYHV
DIMKENALAMYRILRLYHVAIFTCGILWTIFPLVNRALGENVQFTAYFPFDTAESPTFEIVLA
YMSILITLQAYGNVTMDCTIVAFYSQAKTQIKM

>HarmOR6

MSFRKFLFENEAVDGINGPADYLYIKILRFTLDVIRSWPRKELGEPESASFTVFMKYFYLVL
TIATVVGSILYVVVHVTELSFLEAGLMYLIILMSFLDALTVMSLTFSEKYRVLAKDFLTKIH
LFYYKDRSKAAMEIHKKVHLISHLFSLWLLFQMLSGLSLFNLTPMYSNLAAGKYRRGGLG
NTTFEHSLYYLYPFNTSTDVIGYIVACILHWIISYLCSTWFCMFNLFISLMVFNLWGHFKILII
TLEEFPRPKSIGSLPSAYKYSQEELVEVAEKLKDCINYHRVIKNFTNRMSDVFGPMLFVYYS
FHQASGCLLLLECSQMTAQALMRYLPLTIILTQQLIQLSVIFELVGSESEKLKDAVYSVPWE
CMDTKNRKMVRFFLMNVQEPIHVKAMGIANVGVTTMAAILKTSMSYFTFLRSM

>HassOR6

MSFRKFLFENEGVDGIKGPSDYLYIKILRFTLGVVRSWPRKELGEPESAAFTVFIKYFYLILT
IATVVGSILYVVVHVTELTFLEAGLMYLIILISILDAITVMSLTFSAKYRVLAKDFLTKIHLFY
YKNRSKHAMEIHKKVHLISHLFSLWALFQMLSGLSLFNLTPMYSNLAAGKYRRGGLGNR
TFEHSLYYLYPFNTSTDVFGYVAACILHWIISYLCSTWFCMFSLFISLMVFNIWGHFKILIITL
EEFPRPKSIGNLQTAYKYSQEELVEVAERLKDCINYHREIKNFTDRMSDVFGRMLFVYYLF
HQTSGCLLLLECSQMTAQALMRYLPLTIILTQQLIQLSVIFELVGSESEKLKDAVYGVPWEY
MDTKNRRMVRFFLMNVQEPIHVKAMGIANVGVTTMAAILKTSMSYFTFLRSM

>HarmOR11

MHLAGNAVTGITGPMDYKYMKVLRFVLRIISGWPGKALGEKTLRIEGMGHAYYNTILSLV
YLALGIAYLKKNFHRFDFLELGQLYIVLLMNMLSTSRAFTLCLSQKYREVAKIFIQKIHLFY

FKEKSDFAMKIHITVHKISFISAVYLSVLLFIAACMFNLIPMYNNYSAGRFASFDNLENTTY
EQAISCLYPWNFETNFNGYLAATLSGWYGTILCGSSVSMFDLFLCLMIFNLWGHFKILIHN
LEHFPRPASEVVDAEGEERSGRTVGSEMYSQSELEEVAVLLRDCIQYHMLIYNFTNNMSD
AFGMALFIYYSFHQITGCLLLLECSQMTAAALTRYLPLTIIMFGELVLLSIIFETIGTMSEKLK
DAVYKVPWEYMDTKNRRTVLIFLIKVQEPIHVKAGGLVDVGVTTMASILKTSFSYFAFLRT
F
>HassOR11
MYAGNAVTGITGPMDYKYMKVLRFVLRIISGWPGKALGEKTLRIEGMGHAYYNTILSLVY
LALGIAYLKKNFHRFDFLELGQLYIVLLMNMLSTSRAFTLCLSQKYRQVAKIFIKKIHLFYF
KEKSDFAMKIHITVHKISFISAVYLSVLLFIAACMFNLIPMYNNYSAGRFASFDNLENTTYE
QAISCLYPWNFETNFNGYLAATLSGWYGTILCGSSVSMFDLFLCLMIFNLWGHFKILIYNL
EHFPRPASEVVDAEGEERSGRIVGSEMYSQSELEEVAVLLRDCIQYHMLIVDFTNNMSDAF
GMALFIYYSFHQITGCLLLLECSQMTAAALTRYLPLTIIMFGELVLLSIIFETIGTMSEKLKD
AVYKVPWEYMDTKNRRTVLIFLIKVQEPIHVKAGGLVDVGVTTMASILKTSFSYFAFLRTF
>HarmOR13
MKILSDGSDLEGVEKVEDIFYINLARKSMWILDSWPKTPNESVTYRYFVLALNVATLVGG
AVYLRNNTGVLSSFELGHTYITVFMNCITCSRCIMILSREYNEVMLSFVNKIHLFHHRHKS
EYAYKTHIFIHKISHFYTVYLLGLALNGLLLFNMIPFYNCYSRGMFRDVIPANATYDHSVFY
SVPFDYTTKFKGYIAMTSFNCFISYTCTSYFCVVDLTVSLVIFHLWGHMRLLTYHLANFKK
PASVLESNENTDAIKDHSYTQEELKEVFGKLREYIRHHNLILNFSSEMSNAFGPALLAYMV
FHQVSGCILLLECSQLDMKTLVRYGPLTVVIFQQLIQISVIFELLGSSNDKLIDAVYLVPWEY
MDTKNRKLVFVMLRQSQRSIDLKMMSMLTVGVQTMTAILKTSFSYFVMLKTVAEEEQ
>HassOR13
MKILSDGSDLEGVEKVEDIFYIRIARKSMWILDSWPRTPNESVIYRYFVLALNITTLVGGAV
YLRNNTGVLSSFELGHTYITVFMNCITCSRCLMILSRKYNEVMFSFVQKIHLFHHRHKSEY
AYKTHIFIHKISHFYTVYLLGLALNGLLLFNMIPFYNCYSRGMFRDVIPANATYDHSVFYS
VPFDYTTKFKGYIAMTSFNCFISYTCTSYFCVVDLTISLVIFHLWGHMRLLTYHLANFKKPA
SVLESNENTHAIKDHSYTEEELKEVFSKLREYIRHHNLILDFSSEMSSAFGPALLAYMVFH
QVSGCILLLECSQLDMKTLVRYGPLTVVIFQQLIQISVIFELLGSSNDKLIDAVYLVPWEYM
DTKNRKLVYVMLRQSQRSIDLKMMSMLTVGVQTMTAILKTSFSYFVMLKTVAEEEQ
>HarmOR14
MGGIRDFIFNLEAKEGITKPTDYPYMILCRHLLTVITCWPKEPKEGLDTRAKLKARIWVTF
QKIFHLNGCFITTIGMAMYIALHKNSMSFFELGHLYISLLMTVVIFSRVTTLCWNPEYQAVA
TDFLTKIHLFYYKDDSDFSMQTHKQVHKISHLFTLLLTGQMVAGMSLFNLTPMYNNFSTG
KYKKGGLKNSTFEHSLYFSYPFNASSDVRGYILSNIFHWIISYLCSTWFCTLDLFLSIMVFH
VWGHFKILIHDLNHFPRSLNTISFRLDQSNITLTTEMYSSRELVQVSERLNKCVEYHRRIVS
FTDKMSEVFGPMLFVYYGFHQTSGCLLLLECSQMTVEALVRYLPLTIILFQQLIQLSIIFELV
GSVSDKLKDAVYGLPWEDMDTKNRKTVAFFLMNVQEPVHVKALGLADVGVTSMTAILK
TSMSYFTFLRSK
>HassOR14
MSGIRDFIFNLEAKEGITNPTEYPYMILCRHLLTVITCWPKELKEGLDTRAKLRAKIWVTF
QKIFHLNGCFITTIGMAMYIALHKKSMSFFELGYLYISLLMTVVIFSRLTTLCWNPEYQAVA
TDFLTKIHLFYYKDDSDFSMQTHKQVHKISHLFTLLLTGQMVAGMSLFNLTPMYNNFSTG
KYKKGGLKNSTFEHSLYFSYPFNASSDVSGYILSNIFHWIISYLCSTWFCTLDLFLSIMVFH

VWGHFKILIHDLNHFPRPLSMISFRLDHSNITLTNEIYSSRELVQVSERLNKCVEYHRRIVSF
TDKMSEVFGPMLSVYYGFHQTSGCLLLLECSQVTVEALVRYLPLTIIVFQQLIQLSIIFELVG
SVSDKLKDAVYGLPWEDMDTKNRKTVAFFLMNVQEPVHVKALGLADVGVTSMTAILKT
SMSYFTFLRSK

>HarmOR14-2

MAGLLDFFFNYEANEAITTPKNYPYLIIMRISLSLIKCWPKKTTENLAAGAKMKAKVWG
MVQNVLHLAFCVLTIVGTATYVMIHKKNMTFFELGHLYITLMLSCVVFSRLATLTFNEEYQ
VVANEFLNKIHLFYYKDNSEFSMQTHKQIHRVSHLFTLYVTGQMLGGLSLFNLTPMYNNY
SAGKYSKGGLKNSTFDHSLYYSYPFDVSTDVRGYIFSNILHWFFSYIVSTWICTLDLFLSVI
VFHIWGHFKILLHDIDNFPKPSKMVSFKLENTNVTISNENYSTEELEQLADKLKKCIDYHR
EIISFTNKISEVFGPMLLAYYGFHQASGCLLLLECSQMTPEALARYLPLTLILFQQLIQLSIVF
ELVGTVSSKLNDAVYGLPWEDMDVKNRKTVAFFLLNVQEPVHVKALGLADVGVTSMTK
ILKTSMSFFTFLRSM

>HassOR14-2

MAGLRDFFFNFEANEAITTPTNYPYLIIIRRSLSVIKCWPKKTTENLEAGAKLKAKVWGMI
QNVLHLGFCAFTIFGTATYIAIHKKNMTFFELGHLYITLMISCVVVSRLLTLTFQEEYQVVV
NEFLNKIHLFYYKDNSEFSMQTHKQIHRISHLFTLYITGQLFTGLSLFNLTPMYNNYSAGK
YSKEGLKNSTFEHSLYYSYPFDVSTDVRGYIFSNILHWFFSYIVSTWFCTLDLFLSVIVFHI
WGHFKILLHDIDNFPRPSKMVSFRLENTDVTISNENYSTKELEQLAAKLKQCIDYHREIISF
TNKISDVFGPMLFAYYGFHQASGCLLLLECSQMTPEALARYLPLTLTLFQQLIQLSIVFELV
GTVSSKLNDAVYGLPWEDMDVKNRRTVAFFLLNVQEPVHVKALGLADVGVTSMTAILKT
SMSFFTFLRSM

>HarmOR15

MTGFCDFIFNYQPKDGITTPTDYPYMIIARHLLTVITMWPKTSVILQSAKTQKRAKIWLTIQ
KAFHFWLCVTSFFGGTLYILRHKKSMTFYELGHLYISLLMIVCTFSRITTLCLNDEYRVIAK
DFVTKIHLFFYKDRSDYSMETHKKVHMISHIFTLYLSGQMMLGLFLFNVTPIYNNYSAGK
YTSGGLKNSTYEHALYFSWPFNASTDFRGYVVSNILHWLLSFSCSSWFCVVDFFLSLMVF
HVWGHFKILLHDLDHFPRPANKISFILEDSYVTITDEIYSRNELNQVFDRLNKCIDYHRDIV
SFTDKMSEVFGPMLLAYYGFHQASGCLLLLECSQMTVAALVRYLPLTIILFQQLIQLSIIFEL
VGSVSDKLKDAVYGLPWEAMDTKNRRIVAFFLMNVQEPVHVKALGLADVGVTSMTAIL
KTSMSYFAFLRSK

>HarmOR16

MGLRQFLFENEAVEGINSASDYLYIKVLRFMLLIVNSWPRKEMGEPESPKFSAFVKYFYLV
VTVLASAGFILYLVKHNSELTFLETGHMYIVLLMSFNDVSRVATLTMSTTYREVARDFLTKI
HLFYYKDRSKQAMETHRAVHKIAHLFTLWLVSQMLSGLSLFNLIPMYSNYAAGRFSGEVS
KNSTFEHSMYYPYPFDTSTDIRGYSIACITHWIISYLCATWFCMFDLFLSLMVFHLWGHFKI
LNYTLNDFPRPSSEVEAAKYSDEELVEVAARLKDCILYHREIILFTDRMSNVFGPMLFLYY
MFHQASGCLLLLECSQMTAQALIRYGPLTIILTQQLIQLSVIFELVGSESDKLKHAVYGVPW
ECMDVKNRRSVVIFLANTQEPVHVKAMGVANVGVTSMAAILKTSMSYFTFLRSL

>HassOR16

MGLRQFLFENEAVEGINSASDYLHIKVLRFMLVIVNSWPRKEIGEPESPKFSEFVKYLYLVV
TFLLSAGFILYVVKHNSELTFLETGHMYIVLLMSFIDVSRVATLTMSTTYREVARDFLTKIHL
FYYKDRSKQAMATHRAVHKIAHLFTLWLVCQMLSGLSLFNLIPMYSNYAAGRFSGEVSK
NSTFEHSMYYPYPFDTSTDIRGYSIACILHWIISYLCSTWFCMFDLFLSLMVFHLWGHFKIL

NYTLNDFPRPSSKVEAKYSDEELVDVAARLKDCIVYHREIILFTDRISNVFGPMLFLYYMF
HQASGCLLLLGCSQMTAQALIRYVPLTVILTQQLIQLSVIFELVGSESDKLKHAVYGVPWE
CMDVKNRRSVAIFLANTQEPVHVKAMGVAKVGVTSMAAILKTSLSYFTFLRSL
>ObruOR1
MGSLDNKDKNSNEAVTTPMDYRYMKFLRLVLRIISGWPGKELGEKQLLMEGRGYIMYNA
TLSLLYFLLEMLYLRDHVEHLTFMELGHNYIVLLMTLLSCSRCVTLCFASKYPKVVIKFVE
VFHLCHHRNKSEYAESIFQRVQKMSGVFTVYLSCITLNTLIMFNLTPVYNNYAHGKYASM
NNLENTTYEHAIYLKFPFDAYSNFNGYMVAFTFNWYGSYLCASSVTMLDLFLSVMVFNI
WGHFKILLNILETFPRPALENAETDVGSVQFSESEQADVKALLRECVIYHRFIADFANQMS
DAFGIALFLYYMFHQVAGCLLLLECSQMNAEALLRYLPLTLMMFQQLIQVSIIFETIGSESD
KLKHAVYEIPWECMNSKNRMTALIYLMNVQKTIHVTAGGMVDVGVTTMASILKTSFSYF
AFLRTVGAE
>ObruORco
MMTKVKYQGLVSDLLPNIKLMQAAGHFLFNYHDENAGMSTILRKIYSSTHAFLISIHFVL
MVINMAKYADEVNELTANTITILFFLHTIIKMLFFAVNSKSFYRTLAVWNQSNSHPLFTESD
ARYHQLALTKMRRLLYFICVMTVVSLICWVTLTFFGESVRLISSKDTNETLTEEIPRLPLKA
WYPFDAMSGSMYIFAFVFQIYWLLFSTSISNFMDVMFCSWLIFACEQLQHLKGIMKPLME
LSASLDTYRPNTAELFRASSTEKSEKVPDPVDIDIRGIYSTQQDFGMTLRGAGGRLQTFGE
NNNPNNLTQKQEMLARSAIKYWVERHKHVVRLVGSIGDTYGTALLFHMLVSTITLTLLAY
QATKINGVNVYAFSTLGYLCYTLGQVFHFCIFGNRLIEESSSVMEAAYSCQWYDGSEEAK
TFVQIVCQQCQKALSISGAKFFTVSLDLFASVLGAVVTYFMVLVQLK
>HvirOR6
MNLRKFLFENEAVEGINSPADYLYTRILRFNLDFIRTWPRKELGEPENLAFTVFMQYFYLIL
NIVTVMGSTSYIVVRGSELSFIEAGLMYLIFLIGIVDTLTVVCLTFSEKFRVLAKDFLTKTHL
FYYKDRSKHAMEIHKKIHLISHLFSLWILFQMLSGLSLFNIIPMYSNLAAGKYRKGGLQNS
TFEHSLYYLYPFNTSTDITGYIIACILHWIISYLCSCWFCIINLFLSLLVFNLWGHFKILISTLN
EFPRPSSKSVDTQESPYKYTEEELIEVAEKLKDCINYHREIKIFTNRMSDVFGPMLFIYYAFH
QASGCLLLLECSQMTARALMRYLPLTIIMLQQLIQLSVIFELVGTESEKLKDAVYGVPWDC
MDTKNRKVVMFFLMNVQEPVHVKAMGLANVGVTTMASILKTSLSYFTFLLSQTKEE
>HvirOR11
MHLAGNAVTGITGPMDYKYMKVLRFVLRIISGWPGKALGEKTLRIEGMGHAYYNTILSLV
YLALGIAYLKKNFHRFDFLELGQLYIVLLMNMLSTSRAFTLCLSQKYREVAKIFIQKIHLFY
FKEKSDYAMKIHVIVHKISFISAVYLSVLLFIAAVMFNLIPMYNNYSAGRYSSFDNLENTTY
EQAISCLYPWNFETNFNGYLVATLSGWYGTMLCGSSVSMFDLFLCLMIFNLWGHFKILIHN
LEHFPRPASEIVDAEGAERSGRIIGSEMYSQAELEQVAVLLRECIQYHMLIFDFTNNMSDAF
GMALFIYYSFHQITGCLLLLECSQMTAAALTRYLPLTIIMFGELVLLSIIFETIGTMSEKLKD
AVYKVPWEYMDTKNRRTLLIFLIKVQEPIHVKAGGLVDVGVTTMASILKTSFSYFAFLRTF
>HvirOR13
MKILSDGSDLEGVEKVEDIFYINLARKSMWILDSWPKAFNASSKYRYFVLALNVATLIGG
AIYLRNNTGVLSSFELGHTYITVFMNCITCSRCLMILSKDYNHVMTLFVQKIHLFHHKHKS
DYAYLTHIFIHKISHFYTVYLLGLALNGLFLFNMIPFYNCYSRGMFRDVIPANATYDHAVFY
SVPFDYTTKFKGYLAMTSFNVFISYTCTSYFCVVDLTISLVIFHLWGHMRLLTYHLANFKK
PASVLESNDNNKDEIKDHSYTEEELKEVFSKLREYIQHHNLILEFSSEMSNAFGPALLAYM
VFHQVSGCILLLECSQLDTKTLVRYGPLTIVIFQQLIQISVIFELLGSSNDKLIDGVYLVPWE

YMDTKNRKLVFTMLRQSHRSINLTMMSMVTVGVQTMTAILKTSFSYFVMLKTVAEEE
>HvirOR14
MTGIRDFFFNYEAKDGVTNPTEYPYMIMSRHLLTVITCWPKKPKEGLNARAKLRAKIWVI
VQKIFHMSLCLLTTLGMAMYIGLHKKSMSFLELGHLYISLLMTVVIFSRITTLCLNPKYRA
VSTEFLTKIHLFYYKDDSEFSMQIHKQVHKISHLFTLYLTGQMIAGLSLFNLTPMYNNFSAG
KYKKGGLKNSTFEHSLYYSYPFNASSDVGGYIVSNICDWIISYLCSTWFCTLDLFLSIMVFH
VWGHFKILLHDLDHFPRPANLTTFKLDNSNITLTSEKFSSIELGQVSEKLKKCIEYHRKIVSF
TDEMSEVFGPMLFVYYGFHQTSGCLLLLECSQMTVAALVCYLPLTIMLFQQLIQLSIIFELV
GSVSDKLKDAVYSLPWEAMDIKNKKTVAIFLMNVQEPVHVKALGLAEVGVTSMTAILKT
SMSYFTFLRSK
>HvirOR15
MTGFRDFVFNYQPKDGITNPVDYPYLIIARYLLTFISMWPKKSVVYHSARAELKARIWLW
VQKFYHLLLCAVAFFGGVLYITLHKKSMTFYELGHLYISLLMMACTFSRITTLCFNDEYRV
VAKDFVTKIHLFFYKNRSDYSMQIHKKVHMISHVFTLYLSGQMMLGLFLFNVTPMYNNY
SAGKYKSGGLKNSTYEHSLYFSWPFNASTDMRGYIVSNILNWMLSYTCSSWFCVIDFFLS
LMVFHIWGHFKILLHDLDHFPRPLNKVNSVIEDSITITNEMYSQTELDQVFDRLGKCIDYH
REIVSFTDKMSEVFGPMLFAYYGFHQASGCLLLLECSQMTVAALVRYLPLTIILFQQLIQMS
IIFELVGSVTDKLRDAVYGLPWEAMDTKNRKTVAFFLMNVQEPVHVKALGLAEVGVTSM
TAILKTSMSYFAFLRSM
>HvirOR16
MGLRQFLFENEAVEGINTASDYLYIKILRFTLVIVNSWPRKEIGEPESPRLSAFAKYFYLFLT
VLAAIGSIAYVAVHNRELTFLETGHMYIVVLMSLVDVSRVATLTMSTTYREVARDFLTKIHL
FYYKDRSKHAMETHRAVHKISHLFTLWLVGQMLSGLSLFNLIPMYSNYAAGRYSGDVSK
NSTFEHSLYYSYPFDTSTDIRGYSIACVIHWVLSYLCSTWFCMFDLFLSLMVFHLWGHFKI
LINTLNDFPRPSSKVEGAQFSDEELVDVAARLKDCIVYHREITLFTDRMSNVFGPMLFVYY
SFHQASGCLLLLECSQMTAQALMRYVPLTIILTQQLIQLSVIFELVGSESDKLKHAVYGLPW
ECMDVKNRRVVLIFLANTQEPVHVKAMGVANVGVTSMAAILKTSMSYFTFLRSM
>HvirOrco
MMTKVKAQGLVSDLMPNIKLMQMAGHFLFNYHSENAGMSNLLRKIYASTHAILIFIHYAC
MGINMAKYSDEVNELTANTITVLFFAHTIIKLAFFALNSKSFYRTLAVWNQSNSHPLFTESD
ARYHQIALTKMRRLLYFICGMTVLSVISWVTLTFFGESVRMITNKETNETLTEVVPRLPLK
AWYPFNAMSGTMYIVAFAFQVYWLLFSMAIANLMDVMFCSWLIFACEQLQHLKAIMKPL
MELSASLDTYRPNTAELFRASSTEKEKIPDTVDMDIRGIYSTQQDFGMTLRGAGGRLQNF
GQQNPNPNGLTPKQEMLARSAIKYWVERHKHVVRLVASIGDTYGTALLFHMLVSTITLTL
LAYQATKINGINVYAFSTIGYLSYTLGQVFHFCIFGNRLIEESSSVMEAAYSCQWYDGSEEA
KTFVQIVCQQCQKAMSISGAKFFTVSLDLFASVLGAVVTYFMVLVQLK
>HvirOR1
WPLFSTCSYIVMGPTSLKRNMFFWIPVKKNKVDVAKPKVKNITTFQDALRATLIIGQVFSL
LPFVGVFTNVASNVKFIKTSWKCGYSLLSLIGQMFMAVLCVNKLAKSNVSLNGTSPVIFY
VTTCVTMMLFFQVARRWPALVQHISKAEDMDPNFDCSLTRKCNITCAVVLILALLEHILSL
LSAFAGASACYTGMDTYQGFVTHFYPWVFNYLPYSIVLGVITQFLHFQSTFIWNFSDLFVI
CMSYYLTSRLEQVNRKLLAAQGKYLPEIFWRATREDYCRVTQIVRKVDEVISGVVFISFAN
NLFFICLQLFNTLEDGLKGTGECTQLNSQSKLKKIVVSKSGPLGGHEAAAYFLFSLVYLLS
RSVAVSLIASQVNSASSVPAPVLYDVPSPVYCVEVQRFLDQVNGDKVALSGLQFFSVTRGL

LLTVAGTIVTYELVMFQFNSSTPSLNITSPTSATHIITTLAT
>HvirOR3
LIDDGFFSFNLKYLYFVGLWPEKTLTANQKILYKLYEYFITFLTTTFIVLASIGTYQHKHDLV
VVFCNVDKCLVVYNFFLKTIIFFIKRDKLRDLIDEIEMSGDKVTEERKKLMANYVMFITGM
TAAVIGAFSLLALLEGTMSVEAWLPFDPMESLMNQILALEVLAFCVFPGLCRAFAMQGLV
CSMIMYLCDQLTHLQTELRDLTYVKESEMAMRLKFKNAIRKHIRLMGYSGRMESIFKEYF
LVQNLAVTVELCLNAVMVTVVGVHQITLLVSFVAYLLLALVNAYVYCYLGNELIIQSQGIA
LAAYESTWTSWPVDLQKDLLIVILAAQRPLKLSAGGMALLCIQTFSQALYNGYSIFAVLND
AVN
>HvirOR4
EVTAAPVPSESGSRPSRPTHCVVGGAHAFILRISSFFGLAPLRFESRSNGFTVSISGAMCVYS
YILVTVLVICTLFGLVAEINVGVELSVRMSSRMSQVVSTCDVLVVVATAGAGVYGAPRRM
RNMLKFMENIASVDTSIGGQYSRVTERKLCGIILAILIFSVLIADDFTFYALQAKKLDREWD
VVTNYLGFYLLWFVVLILELQFAFTALSVRARFSAVNDALALTARQVSIPVEKPKTSSPLNI
YAIRVAPVDSQRSANVSLLVDTMTGREHVVIIKRTASGEPRLIVSPCDAVRRLAALHGTLCD
VVNSIDDSYGLPLVVILISTLLHLIVTPYFLIMEIIVSTNRIHFLVLQFLWCVTHMLRMIVVV
EPGHYTIAEGKRTEGLVCRLMTSAPSTGVLPSRLEIFSRQLMLQSVSYAPMGMCTLHRPLI
ASVIGAVTTYLVILIQFQRYDN
>HvirOR5
AYLDKVFLWSCLYGVFGSKRFISLIWSTLILGSLVIIEVLAIWKVIRALAGVARDMSGHRSV
TARLAGTIFYSISILSLVLVSKLYYNWRTNIAGVWGKVERSVGVKIPVDRTLKCRMTFVAG
LMTFFSIFEHAMSILSSVGLDCPPSLILKRYVLVSHGFIFMGQDYSEWFAMPLVIISTIATLL
WNFQDQVIVLISMGLTSRYRRLNECLAKVCELEKQHKDSDKKIEAVKVYTWRKIREAYV
KQAMLVRKIDVALGGIVILSCSCNFYFICLQMFLGITQGLSSDLLSLIYYVISLAWLCTRVIS
VVLAASSVNTHSKLALNHLYNYETHCYNVEVERLQDQLTKDYIALSGMGFFYLNKTILLQ
MAGAIVTYELVLIQFDDQGNDALNATKI
>HvirOR7
IEKLGVLFRFSGMNIKNKIVTPLDTIKYRWLYTLNFLVVFSAIIGSVYYVILGIKQGKNFIEV
TSVAPCLTFSILSMIKSLYHLMYEEHIQELIELLRELELRENNREKCIEKEEIIASETGFLNKVI
NVLYVLNCSMIVVFDMTPMIMIAVKYYKTNEFEMLLPYLDVFSFIPYELKYWPFAYIHQIW
SECVVLLDMAAADYLFFTCCTYIRVQFKLLQYDFERMIPDRGISKGKFYEENELRNKFTEL
LKWHQDIIYSSTILEIIYSKSTLFNFLSSSLVICLTGFNVTIVDDIVIIITFLTFLSMALMQVFFL
CFFADLMMTASLEISNAVYNCRWYSANIKVGKQILFVQTRAQEPCKLTAAGFADVNLNAF
MRVLSSAWSYFALLQTVYGGK
>HvirOR8
MVFRQIDCFKINMKFLKFLAIWPGKDFTRRYKYYTVAFLTAYFIIFMILFTINLFFLPKQLDI
FIENMVFYFTDSATLSKVMTIGFMRKKILQLFEMLESDIFQPDNAEGLAVIEKAKKFNKLY
WNIILAVSFASCASNLFPPIIAHFILGTELVLPICNYGFLSEDFRQMFGVPLYLYQGSAMMFD
MLYSVNIDTLFAGLMVLAIAQLDILGIKLRRVTDKEVLEETDSETSRQHRDNHKEAIKKIN
HCIIHYEKIHKYCSLVEDVFSITLFVQFGMASCIICICLMRFTMPAPLVYYLFLATYMFVMIL
QILVPCWFGQRIIDKSNLLAFSAYDCEWTSETRQFKSSMRIFIERAHKPLSITGGKMFCLSL
VTFTSIMNTAYSFFTLLQNVKSRK*
>HvirOR9
MVDQFQKCLKSVNLYLKFIGLHLESKDTTKTFIERSRSHRLYFAHFFSLNLEVVAQILWVLE

AVITRKSFVEITRLIPCLILTLISDFKTLSLLYYARHNNEFIVTMKSLLLNQKQLEEKETRFRE
DLIDKHVLMLTSITKKISYLIGMGLLMFALAPAFIIIPHYFKTNEVKLEMPFIAYYPFNEFDS
RIYPWVYLHQVWTACVAMIMVYGPDCFFFTCCTFIHIQFSLLNNDMERIVNEDTPRYDKT
KFKELAVRHIELMRCVNLLEKIFSKSILFNALTSSVIICVTGFNVLVVDNIVMMASFTAFLLF
GLMQIFLYCYYGDTIMRSSMQVSTSIYNSPWYNIRAADRKGFFIVIIRAQKPCELTANGFFK
INLSAFTSILSTSWSYFALLKTMYHPE
>HvirOR10
IDAVSEASYLLFTQASLCYKSTAFMVNKQSLLELLEIMDCEIFEPKSAEHEKILAAQARKIK
RLCLFFLTSATTTCTLWAMIPLFDAASKRSFPFRIWMPVTPLKSPDYELGYLYQMVSIYISA
FLFISVDSVAVSMIMFGCAQLEIIMDKIQKIKYVFESADSEEKRREIIKINNEFLVECIKQHQT
VERFIQLCEDTYHANIFFQLTGTVAIICNIGLRISIVEPNSVQFFSMLNYMVTMLSQLFLYCW
CGHELTIRSENLREWLYQCPWYEQDTKFKRALFIAMERMKKPIIFKAGHYISLSRPTFVAIL
RCSYSYFAVLNRVNTE
>HvirOR12
MMEEEPLLIDKTVKKVEFLFRWTGINIKSGTKTRMDTIKSRAVYIINFIWLNSDLAGAVVW
FFAGIANSIGFTELTYVAPCITLSFLGDLKSLYLIIREKNVDNLIQMLRDLEINERARPKSEEK
DAIIKYEHNFVTTVISVLNVFYFVLLVAFALSPVTLVALKYYTTNELELLLPFLIVYPFNPYD
IRYWPWVYLRQIWSEVVVVIDICTADYLFYTFCTYIRMQFRLLKHCIERVIPEDDGSGRLL
NIEQVRAEFVQLIKWHQDLISSANMLETVYTRSTLFNFVSSSVLICLTGFNVVAISDVAFVV
TFLSFLFMSLLQIFFLCFFGDLLMTASTEVSDAVYNCRWYLADTSFGKDLLLVQTRAQTPC
KLTASDFSDVNLKAFMKILSTAWSYFALLQTLYGAPT
>HvirOR17
MSLRSECARSVAPHVRVLRRVGFLRGAALPARPRAVRLALRGYHALALAATSTYVLQQAV
YAYQERGDMEKLSQVMFLMLCHVTCVVKQIAFHVDADRIDRLIAGLDEPLLNQCEGERG
ALLRGTARGAARLLRTYVGCAVATCVLWIVFPILNRIQGISFEFPFWTGFSYDHNVVFSLVL
LQSFYCTNLVAIGNTSMDAFMATILDQCKTQLRILRINFESLPERASALHMETGENYDTILD
ELFVDCLVHYNKITEMCAELHDVFTAPLLVQFAVGGWILCMAAYKIVSLEVLSIEFASITLFI
TCILIELFIFCYYGNEVTVESERVSQSLYSMEWRRARLAFRRSLVLVMERAKRPLRPAAGR
VIPLSLDTFVKILKSSYSFYAVLRQTK
>HvirOR18
MEMKVDVLPEKKYKGFNETFKLCAFSLAFAFLYPNRTTALRRCITITLIVTFCGGQLFWFIT
YTFKCLYTLDIYNFARNMTLAVVLVLFFIKTYYVIYATSKFAPLLDKISEDLLEANNLEEEF
QVLYDDHIKIAKVGEISWLLIPTIMSALFPIYAGALMTIESIQTDDYERRMVHDMELLFVED
IQSETPFFQCMFAYNCVQCVVLVPNYCGFDGSFCIATTHLRLKLKLMTLKVYKAFKYSKS
RQELRVRLYESIKDHQDALDFYVQLQNVYGPWLFAVFLLTSFMISFNLYQIYLLQRIDPKY
TSFGVVGVLHIYLPCRYASDLTRVSEEIPDDLYLAEWEAWADPSITKMLMFMITRAQKEMI
VTGMGLVVFNMEMFKSILQTSYSFFTLITA
>HvirOR19
MKDRDILFKYCKVMFYIGSGNCWYKEDEIGNDRSILYRVCSASLMLLYSYMAIFELIAFAF
GNFPEEEKRQALIGGAGHTVMLLKALFLTTKKLPIRSLNRKIVSICEDYEDSALMARKYKI
MKINAISYLGLVNGGVLLYIIEGLRNMLNGSHFVTVVTYYPSFEDDSMLATIVRVFNTIIFI
MIMLTIVISVDTYIVTYFIMYRYKFITLRKYFENLRNDFFTLIERKEVELATEKLANGLVEGI
KMHSSLIRLKPEIDKAFATVSAIRVFESSSLAVCLLFEISPSDQRIPIEETVKTMIFIFALFFAM
GLFLCNAGDITYQASQLTDAIFYCGWQSCPPRPRSAPNHNIRKMVLLAIMQAQRPPVMKA

FKVLELNYATYIQLVRSTYSVFTLLCAQKT
>HvirOR20
EFKPFHETYKVITFTLCIAMIFPNPRTEKWRLISIPLLIATVAPVASMIFLDMYKCWTNNDIV
NIIRHSTVVGPFLGGFFKMILMYHKRKEAKQILDEFDRDHFMFNDFSETYKDIARASIRNC
QIYSERLWALLVTTCVMTFPVMAIVLNIYNFLFKSEPTKYMIHDLEKPFSESPEERFESPYFE
LLFAYMFYAAILYVVNFTGYDGFFGLCINHACLKMELYCKALEEAMVADREEVYGRVVAV
IREQCRMFRYVDLVQETFNIWLGIIFIATMIQICTCLYHITEGYGFDIRYMIFVYGAVVHIYLP
CRYAAKLKAMSMETSNRFYCCGWEKVDDERVRKMVLFMIARAQVPNEITAFNMLAFDM
ELFLSILQTSYSMFTLLRS
>HvirOR21
MDHFSGYYKSSKTTEFLINLNQFVFIFGLPNFWVQELDISDSFRKIVGYLNKYGNWSIFGLI
LAEYGAFFTQKNLNQRQSSDLVLFMISHSIITGFRVRICHQEVEIRNVMYKLGIALKEVHN
DSEAEEQMIKRSKFFSWALILNCVISFLMYTIEAVLRVIRAGVTFTTVITVYPDVEDRSGLS
NGVRVMFYIIWCIYLTRVFAVYTLVICLTIAMSHQFKNLTSYFYSLSSIFDDDQMTQAEKEQ
EYERAFRVGIKIHSDTLNCTGDIQKICRDVFSGQIIFNITLLIVLMYQMVNSARSLTNALTLV
MVALSILLSTGFFMWNAGDITVEAKSLPTAMFSSGWEHCGRDSSVRVRKLIVIAMMQAQE
PVVLTGLGIIALSYQSYVSIVKSSYSVFSVLY
>LdisPBP1
SKEVMKQMTINFAKPMEACKQELNVPDAVMQDFFNFWKEGYQITNREAGCVILCLAKKL
ELLDQDMNLHHGKAMEFAMKHGADEAMAKQLLDIKHSCEKVITIVADDPCQTMLNLAM
CFKAEIHKLDWAPTLDVAVGELLADT
>LdisPBP2SKDVMHQMALKFGKPIKLCQQELGADDSVVKEFLDFWKDGYVMKDRQTGC
MLICMAMKLELLDSAMEIHHGSTFAFAKAHGADEAMAQQIIDIVHGCTTTYPAAETNDPC
QRAVNVAMCFKAHVHKLNWAPDVELLVADFLAESQ
>EgriOBP1
TAEVMSHVTAHFGKALSECREESGLTPEVLEEFQHFWREDFEVVHRELGCAIICMSNKFSL
LQEDSRIHHVNMHDYVKGFPNGQVLSAKMVELIHNCEQQYDDITDDCARVVKVAACFK
RDAKKEGIAPEVTMIEAVMEKY-
>EgriOBP2
SQDVMKSLTLNFGKPMEVCKKELDLPDAVTKEFLNFWREGYEVKNRLTGCAIICMSEKLE
LLDEGLKLHHGNAKDFAKKHGADDGMAQQLVDMIHSCMESTPPNTDPCMKTVDVAMC
FKLKIHDLSWNPDPDLIIAEVLAEA-
>EgriOBP3
RECSQEVMHKITKDFAYVLEDCKKQENVGDHIMQDIFNFWHEEYALVNPELGCVMLCMA
GKLDLMDGDDMHHGNAHEFAKKHGADDDLAKQLVTMIHDCEKASASIADRCARALETT
KCFRGKIHGLKWAPSMRVIMEEVMADMNV-
>EgriOBP4
DLIGQPRNNKEATLKPISTCCDIPELGDPKPLAECSNPKLPGPCNDVQCVFEKSGFLIDKST
LNKETYKAHLRQWAEKHKDWSAAVERAIEDCVEKNLRQYLDIPCTAYDVFTCTSIAMLK
KCPASSWKC-
>EgriOBP5
NAVPANKAVLCGFMPRNLLACMGNPRIISESLTAKCNAVSTCDKDKCIFTQAGWLKDNKV
DKKKVADYFDTYAADHPTWAAAVTNLKAKCLGDQDLPPQTTLVSCPAVDILHCAFISFVK
NVSPAQWVQDERCAYARQYAASCPVCPEECFAAMIPNDSCHACYSLPTTPVA-

>EgriOBP6
QEQGPPHGPPPQWANHKCAGPPPAIKNPQKCCEIQQMFTEEEMASCGINKFEEENRQGPP
KPPDCNKQECLLKSKDCLNDDGSINHKAVAEHLNNWASEEWKPAVEAAVAVCLGENEVP
GPPHICEANRLMFCIGGVIFSECPTWQDNDDCKQLKEHINECKAAKFPPPN-
>EgriOBP7
DMPACARVSHGGLMCCKKEMPPMKPPSDDTKECFKLMKKPPSCDVDMCIAKKKGLVSD
DGTIDKKKMAEDVEQLFENDPELVDSLKENCINGDLAKYGPADFCDLMKQRLCFDVQIIK
RCNEWDDTGDCSGMKDLAAECVKQFS-
>EgriOBP8
EIVRTAITLPPEIAFDIAKAIKEVCVPEDRVPDIIRMIREGETNNNTEFKKIIHCVIKEAKYMT
ADGKRINVEKAASIFPNKVLMFKILSQCDKNIVTNDPEEYCIKFYDCFQENTPYRLSF-
>EgriOBP9
MTKEQLRKTGKMLRKQCLGKVGVEEEKISQIEKGKFIEEKDVMCYIACVYQMTQIVKNN
KLSYESALKQVDLMYPADMKASVKASIENCKDVSKKYKDVCEASYWTAKCLYDDNPKD
FMFA-
>EgriOBP10
MTRQQLKNSGKLMKKTCMPKNDVTEEEVGSIEQGKFIEDRRVMCYVACVYTITQVIKNN
KLSYEAVIKQVDMMFPPEMRTAVKAAAENCKDIAKKYKDDICEASYRTAKCMYEYDAEN
FVFP-
>EgriOBP11
MTMKQLRSTGKMMRKSCQPKNNVEDEKIDPIADGVFIEEQEVKCYIACIMKMANAIKNG
KLNYEAAIKQADLLLPDEIKEPAKESITVCRKVSDQYKDICEASFHTTKCIYNNNPAAFYFP
-
>EgriOBP12
GKDKPVFTDEMNEIIQTIHDACVGNTGVSEEDITNCENGIFKEDPKLKCYMFCLLEESSLA
DEDGVVDYDMLLSLIPEEYYDRTSKMILGCKHEDAPGKDKCQSAFDVHKCSYQKDPDLY
FLF-
>EgriOBP13
GSVHLEGAQKDKAAETAMQCMKETGVKPEMVAEVKKGRLSEDEDLKKFTLCFFQKAGII
SPDGKLNVDVALSKLPAGVDKSEAEKLLNDCKTKKGKTAADTAYEVFKCYQAGTKTHIL
L-
>EgriOBP14
LSNEQKEKLKKHKTECLAETKPDEQLVNKLKTGDYKTENEPLKKYALCMLIKSELMTKD
GKFKKDVALAKVPNAADKPAVEKIIDACLANKGNTPQQTAWNYVKCYHEKDPKHPIFL-
>EgriOBP15
CKNCVVLGKAEKAMFRAHSDACLEQSQVDPRLVDTLLNGELTDDPALRKHVYCVLLKC
KVVGKDGKLLKSAVLGKLATRSDGKNASKILESCSDQSADLAPQDAAWNLFRCGYDRKA
VLFDYMPTDKQPALDA-
>EgriOBP16
APPPMDEDMAELAAMVRSSCAAESSVDLALVEKVNAGTSLMDNGALKCYIKCVMETAG
MMSDGVVDVDAVLGLLDDKTRAHNEAALRACGTKKGADDCDTAWQTQKCWQDANPN
DYYLI-
>EgriOBP17
MECGKEVGVTKEQALKAKQMKDPDVLDPCFYACFFKKSGVIDDNGMYNADAIHEKHKK

YIHDEEDLKKIEEMAKTCASVNDKTVGDGTKGCERAKLLVHCLLEHKSAFSPLE-
>EgriOBP18
AISEEDKNAIKAEMLPVLAECGKEHGVTEKDVKEAKESNNVDAINPCFIACFMKKRKIID
DEGKYAPEVAKSEHAKYIHDAELVAKLDEISDNCASVNDQAVSDGAKGCERAKLLTACLT
EHKDILTEIFKD-
>EgriOBP19
MTAEQKQKIHEHFETIGMKCMKDHHITEADITDLRAKKVPSGPEAPCFLACVMKDIGVM
DGNGLIQKETALELAKKVFEDAEELKMIEDYLHSCAHVNTEPVSDGDKGCDRAIIAMKC
MIENASQFGFEL-
>EgriOBP20
AQLTASQKSKIYGSVLSAGMECMRDFPLSLDHIQAFRNKKAPNDEVAKCFTHCLYKKLGL
MDDSGKISEKTAKAATKKVFKEGDEMFTKVEELISRCIHVNDAETSDGDKGCDRAKLAFE
CFIEHAKELDLDVDL-
>EgriOBP21
AEKLNDLKQEYDEILKECIAQNPMTAEDVESLSKDKRTYNVNCIFACALKKGGMMDDDG
NLSVEGVRKSAEAYLSDDPELLKKSELFTDACKSVNDAPVSDGKKGCDRASLIFQCSVEK
APSFQLF-
>EgriOBP22
MTEEEMKVEFTKLIMKCTKDHPVEMTELMLLQQMVVPKKKETKCLLACAYRMEGVMT
ANGLYDIDHAYKIAELSKNGDEKRLENGKKMADVCVKVNDIEVSDGEKGCERAGLIFKC
TVENAPKFGFKI-
>EgriOBP23
YGGVSVERQRSSEAGRGPSFWDELYLGKTDLEVKGWFFSLAVICNKDYTIAPEELAMMQ
DHRISDSPNAKCLMACIFKKADMMDDKGNYDLEKTNKWVETEFSDSATRLESARNLFN
MCKKVNDEPVTDGEKGCERAYLLSKCLVENSPKIGFATIE-
>EgriOBP24
DSAISADTEQRCKNPPTAPQKIERVITLCQDEIKLSILREALDVIKEEHTMPTQKKRNKREV
PFTHDEKRIAGCLLQCVYRKVKAVDGYGFPTLEGLVGLYSDGVNERGYFMAVLEASRECL
MRSHDHFSRTVPMDNGRNCDVSFDIFECISDRIGEYCGNAGL-
>EgriOBP25
DSNIMKNLSLKFGEAMSICKAELNLPDSINEDFYNFWKPDYELQHRETGCMIHCLSTKLN
LIDPEGKLHHGKAKEFAMSHGADEGMAQQLIDIIHNCENSTPQNEDGCLMVLAVAKCFKV
EIHKLNWTPSMDMVVGEVLAES-
>EgriOBP26
MCYSILRSLDTMPLLSRFTKIIVFLVFVSAAYLVVSFKPLTKEEQIKGFNTMQESVEPFRRNL
SECAMQVKASMEDIQHFLKQVPPITMEGKCLVACILKRNAIITKNKVNPEIILEANRAVYEE
DSEVGIRLKKAISECITAVDNIFEICEYASVFNDCMHMKMEHVLDKATMERRMEAMGQM
TSEPDYWNDDDDELMQLVRDEL-
>EgriOBP27
QVQENDKRTIKRLSPKQESPVHGECLIACVLKRNAVIENNKVNKENLIQLVSKFFAKDTHL
MKKLEKNLDQCIEMSAHHHNECDLAAKLNECTNDLMTSNKHKIVVNY-
>EgriOBP28
DLLSQARNKGATLKPISACCDIPELGDPKPLLECSNPKLPGPCNDIQCVFEKSGFLIDKNTL
NKEAYSNHLRQWSENHKEWSEAVEKAIKDCVDIKDLRQYLTNPCKAYDVFTCTGIAMLK

KCPESAWKC-
>EgriOBP29
AVVADVQVMKDVTLGFGQALEVCREESQLSQDVMEEFFHFWREDFKFESRAVGCALQC
MSRHFNLLTDSSRMHHENTHRFIESFPNGAVLAKQMVSLIHGCEQQHEAEPDHCWRILRV
AECFKRRCQDAGIAPSMEIIMAEFIMETEAK-
>EgriOBP30
MGTRMDNYDRMDGNNRMGRHGHDRDDSMSNHDNAHSRGREHVSSGRNDFQHDDYGR
DMSNQYGYQSSTQSSRRYKREKKTENSGQRSQFNPNSKKKTNYDDSYQSDERSGKENNT
NKDDVKKSCALHCFLEELEMTAEDGVPDRYLVTHTLTKDVTNEDLKDFLQESIDECFQML
ENENTEDKCEYSKNLLMCLSEKGRANCDDWKDDLRF-
>EgriOBP31
LVTCSKLSTETTKVTATTESSNNMKPDDNNKELDDSKAVSDTSRIAVDSKYTSNDTKALYD
EVMDVMTTCNESFRIEISYLVSLNETGSFPDETDKTPKCFLRCVLQSLEVASMDDGKIDPK
RAAEVFGDQRENIEETATLCAQRDEKCHCEMAYNFLKCLFSTKIENVEKSKT-
>EgriOBP32
KKVVHVSPDLAGKILAAVSKCQPETGLDANGLLMIYDGKYREDQQFKDFIFCCYKSTGFL
RNDGQLNQEKSIKAFNNDPLVQEGIRRCGSQQNKDAKEDMFQFYKCFVDTTPVIITL-
>EgriOBP33
AGVTDEEKKQMHDYLVSATESCSKEFGIPSEDFEKAKRNKELQSLDPCFVACILKGNGLID
DKGMFDPAKGTSIAEKFIKSPDDIAKVKKISDICSSVNDEAVNDGDKGCDRAVLLLKCLME
NKSLVV-
>EgriOBP34
LSDEDKETIKGFMVPLLAECGKEHDITMEDVIAAKKANDGDALNPCFYACFFKKIGVITEE
GRYAPDVAKEGHKKFIHDDELVSKLDSVTDKCSSINDQTVTDGAEGCEISKLLVNCLKENK
DEFSLFDD-
>EgriOBP35
DSPAPSQGVYCGFMPKNLLTCMGMPSLIGEDVIRRCSSSKGPCDKYTCLFTQSGWLKSGQ
VDKKKVSSYFDTFAASHPSWTGAITHMKAACLGDKTLPAQGVLINCPAYDTMHCALTSFV
KNVSPSQWSPSAE
>EgriOBP36
AGEGNIKLLQEEVAAALKICAESDNSNPQPQRQRRFAHKLHDGDNPQQQKQRRFDDNVRI
DDGAKESNPYSHERRNITDMKDQISVINSTDYDYDGYGSGSMGEKFVNSMPKAADGRYY
PYANGTNRTRRSEPLFSKPDNEQCLSQCVFANLQVVDSRGIPREAELWNKIQSSVTSQQSR
AALKDQTSACFQELQSEAEDNGCSYSNKLERCLMLRFSDRKPSGTQTNNEQGTKST-
>EgriOBP37
VPKKTVHITPELAGKILVALQKCQPETGIDSDAIVMIYDGKYRDDQQFKDFVFCCYKTTGF
LRNDGFLNEDKAIKAFRNEPLIEEGIRRCGPLRGKDSKESLFKFYKCFVDTTPITITL-
>EgriOBP38
FQYVVITINNLIKACFFFQMSRQQLKKTLGIMKNQCLSKTGAAEEKVANIDQGVFIEEKEV
MCYVACIYKSIQVVKKDKLDGDLISKQIDLLYPQDMKASVKNGVTKCMPVQNGYKDMC
EGIFYATKCLYEADPSTFVFA-
>EgriOBP39
SPTYFYETIEDFDDSNVKHDLFMTTHRSEELVPTRGTTEAPIEDEWKQRGEYRIRCVDELG
VGPKRVVNLLATANHKWDNTDVDLKHWMLCVLNKMNLMSKEGVFRMDVAMSKIYNK

DKDDAEILINHCLPKKKLPPYETAWQYVKCHNAAKSNFAAISVF-
>EgriOBP40
MQCCVFPAFFTKEVAKECGAMTELISADKKNNITALRRIASSKCGHWKCILEKYDLLGPE
DTLDDEKYYSHLERWSTLNPSFTEVMKNAKMHCKSEYRMYLPLKICEFYDIHGCIRNYV
KLACPVIFPTTECTEQKQFYEECREYFI-
>SlitPBP1
SQDLMAKMTKGFTRVVDDCKTELNVGDHIMQDMYNYWREDYQLINRDMGCMLLCMA
KKLDLMDDQTMHHGKTEDFAKSHGADDDVAKKLVSVIHECEQQHTGIADDCMRVLEVA
KCFRTKIHELKWAPSMEVIMEEVMTAV
>SlitPBP2
SQDVMKNLAINFAKPLDDCKKEMDLPDSVTTDFYNFWKEGYELTNRQTGCAILCLSSKLE
ILDQELNLHHGRAQEFAMKHGADEAMAKQIVDMIHTCAQSTPDVAADPCMKTLNVAKC
FKLKVHELNWAPSVELIVGEVLAEV
>SlitPBP3
SKDPMKYIASGFVKVLEECKHELNMNDHLIADLFHYWKLEYTLLNRDTGCAIICMGKKL
DLLDANGRMHHGNAQEFAKKHGAGDEVASQIVQIIHECEKKHERDDDECLRVLEVAKCF
RTGIHELNWQPNVEVIVSEVLTEI
>SlitGOBP1
DVNVMKDVTLGFGQALDKCRQESQLTEEKMEEFFHFWRDDFKFEHRELGCAIQCMSRHF
NLLTDSSRMHHENTEQFIQSFPNGEVLARQMVELIHACEKQHDHEDDHCWRILHVAECFK
QACVQRGIAPSMEMMITEFIMEAEAR
>SlitGOBP2
TAEVMSHVTAHFGKALEECREESGLSAEVLEEFQHFWRDDFEVVHRELGCAIICMSNKFS
LLQDDSRMHHVNMHDYVKSFPNGHVLSEKLVGLIHNCEKQFDSMTDDCERVVKVAACF
KVDAKAAGIAPEVAMIEAVMEKY
>SlitOBP1
ASNSNPGTPNANPGTYCGVTPDNIYRCLNNPRVVTPEVSTKCGSQFTECEKMTCIFRELK
WSKRGAIDKAKVRAYFDQYETEHPEWAQAVQHVKAFCLASELRAQGVFLNCPAYDIMQC
VLASFIKHASPSVWSTATDCAYPKAYAADCPVCPSDCYSPQIPYGSCNACYTQPRTV
>SlitOBP2
DSAISADSESRCRNPPTAPQKIERVITLCQDEIKLSILREALDVIKEEHTMPAQRRRDKREVP
FTHDEKRIAGCLLQCVYRKVKAVDGYGFPTLEGLVGLYSDGVNERGYFMAVLEASRECL
MKNHDKFSRTVPMDNGRNCDISFDIFECISDRIGEYCGTSGL
>SlitOBP3
TEKGNKIASECIKESGVKSDVLAEAKKGNLGDDPAFKEFTYCFFKKVGIVGEDGKLNRDV
AIAKLPSGVDKAEAEKLLDSCKSKTGKDAVETVYEIFKCYQHGTKSHIMFAS
>SlitOBP4
LTDEQKEKLKKHRTECLTETKVDEQLVNKLKGGDYKMDNEALKKYALCMMMKSELMT
KDGKFKKDVALAKVPNPADKPTVEKLIDACLANKGNTPHQTAWNYVKCYHEKDPKHAIF
L
>SlitOBP5
ASTKEAMTTTMSDQVNSIDVDVLAVMDMCNDSYRIDPTYLQALNESGSFIDETDKTPKCF
IRCVFENVGIVSEDGKQFNPARAAVIFAGERNGKPMEDIADMTALCATDRQETCPCDRSYK
FLRCLMSMEIERYEKS

>SlitOBP6
ESKFGEIVKRTVIATAHTCMDHVNATAKDLEHLRDEPPYPETSACIVKCLLEKVKYMRKQ
TN
>SlitOBP7
GKNKPVFSDEIKEIIQTVHDECVAKTGVAEEDITNCENGIFKEDAKLKCYMFCLLEEASLV
DDDDTVDYDMLVSLIPDEYYERTTKMIFACKHLDTPDKDRCQRAFEVHKCSYEKDPDLYF
LF
>SlitOBP8
AVDIKKYFKVCDRNAIDVNDCMAEAVRQGIATMINGIDELGVPPIDPYLQKDFRLEYKNN
QLAAKLTLKNIYVEGLKEAIVHDARLRADDDKFHLEVDLSGPRVSVRSDYYGEGQFNAL
KIVAYGQVNTTMTDLVYTWKLAGVPEKNGTETYIRIKDFYMRPDVGSLVTHFRNDNPESR
ELTDLGTRFANENWRMLYKEFLPYAQANWNRIGVRVANKLFLKVPYDQLFPTSS
>SlitOBP9
LNCRSSGGPKEAELKNIYKKCLKMQEGKNSSKGNSAQDWKEPRVQIQSNDNDGSGNRGR
GNKNNRNDMNGGRDDRFGRDDYFNGREDFPQSDEYGGDMGQYNNNYYSTTQSSRRYK
RERRPSNSGQRSQYNPNNHKISGYEDNFRSDERNTTDNNSSKETDNKSCALHCFLENLEM
TGEDGMPDRYLVTHAITKDVKNEDLRDFLQESIEECFQILDNENTEDKCEFSKKLIDLSVR
E
>SlitOBP10
MKEGNRYSHERRITNDSGDQLMVINATDDDYSGYGSGNMGEKLLTSVPRPATPSNNINKN
NINRTKRNEPLLNRPDSDQCLSQCVFANLQVVDSKGIPREAELWNKVQSSVTSQQSRSAL
HDQIQACFQELQSEAEDNGCSYSNKLERCLMLRFSDRKVDGKGNAKKSSTEQTG
>SlitOBP11
TAEEKAAFIEAVKPYVQECSKEHGVTPEDIKSAKAAGNADGINSCFLSCVYKKAEVINEKG
EYDVDKALEKLKKFVSNEDDYAKFANIGKKCASVNEKSVSDGEAGCERAALLTSCFLEH
KSEISA
>SlitOBP12
MSGDEEAGIKEALRPFVQECADEFGITEEQFEEAKKKASAADIDPCFMSCFLKKAEFFDSQ
GKFDVDSTMAFAKEHLTSEPAMKFVEAVGDECVKINDEDVSDGDKGCDRAKLLFECIAET
KKKME
>SlitOBP13
QQPVFESGPPEPWGPPQRPAHRRQFLPRIPKRCWVPPQRINVYNCCPIPTLYPDEDMQSCG
FEKTSGNTDQPQKPVFRPEGTCKEGYCVMGKFDLLFANNSVDFVKFREYLDNWAESYPE
FANAIRIAKQECAQDGGPEVPPICEPDKLFLCLTSTIFWNCKLRDGDGCAALQEHMNECK
QYYTRQMEPTMKDIEVR
>SlitOBP14
MSRQQLKNSGKMLKKNCMNKIGVTDDQVGSIDKGKFIEDRKVMCYIACIYELTNVIKNN
KLNYEASIKQIDLMYPPDVKESAKAAVEKCKDVQKKYKDICEASFYAAKCMYEYKPEDFI
FA
>SlitOBP15
MFNNCFVYSMTREQIKNSGKLIKKTCSAKNDLTEDEVKDVDKGKFIEKKDFMCYIACVY
KMGQTVKGSTINHDMMLRQVDMMFPNDMKAPVKAAIEHCRPVAKNYKDLCEASYWTA
KCIYDFDPANFMFP
>SlitOBP16

MLAEELKDCFDGSGPKDPMKCEIDLCIAKKKGFATDDGKLDIKKFEEVITKDVGSDKDLL
DEIKTNCINGDLNNYGPPEFCDFIKIKHCVTLHMMNHCSEWSDDGNCKVVKELVGKCAK
VI
>SlitOBP17
MTRQQLKNSGKLMKKSCMPKNDVTEDEVGDIEKGKFIETRNVMCYIACVYTMSQVVKN
NKLSYEAVIKQVDVMFPAEMRDAVKAAATHCKETTKKYKDLCESSYWTAKCMYDYDAQ
NFVFP
>SlitOBP18
MILXYTQKLTNMLLTKIVKFFILVATCEAMTMKQIKNTGKMMRKTCQPKNNVEDEKIDPL
SDGVFIDEKEVKCYMACIMKMANTIKNGKLNYDAAMKQADLLFPDDIKEPAKEAITACR
KVADAHKDICDASFHVTKCIYNHNPSIFYFP
>SlitOBP19
DSVSFIKKCKWDDGKCAKESGQNVIQKFAAGISEYNVGVSDPLHIEYVDASSPNMKLIVT
DVVVTGLRNCEVKKIQRFEDSSKLIVKLLCAAELNGKYDMKGQLFVIPIEGNGGLYSKVP
KIQINAEVDLNTKQGKDGKDHWIVKSWRHTFELKDKSTVKFENLFPDNEFLRTSTNELIA
QNGNDVIIEIGANLIKAIVGKIVENIKKFFIAVPIEDLSL
>SlitOBP20
AVEMDEDMAELARMVRDNCAGETGVDVALVEQVNAGAELMPDDKLKCYIKCTMETAG
MMADGEVDIEAVLALLPPSLAEHNAPALRACGTQRGADHCDTAFRTQQCWQNANKADY
FLI
>SlitOBP21
IHYLCAARAVHKIKNITNCENGIFKEDAKLKCYMFCLLEEASLVDDDDTVDYDMLVSLIPD
EYYERTTKMIFACKHLDTPDKDRCQRAFEVHKCSYEKDPDLYFLILRREQLASRDDCVAIS
GIN
>SlitOBP22
QVKKTMGIIKNQCMPKNSVTEEQVGRIEQGVFIEDRNVMCYVACIYKSLQVVKNDKLDM
ALITKQIDILYPPELKEPVKKSVAACFHSQDNYSDFCEGVFYASKCLYEKDPASFIFP
>SlitOBP26
MTAEQKALIHEHFETIGKSCNKDSTMITAEDIANLRAKKIPTGPNAPCFLACMMKQIGVM
DDNGMVQKETALEMAKAVFDDPEEIKAIEDYLHSCSHINTESVSDGAAGCERAMLAYKC
MTENASKFGFDI
>SlitOBP31
RTEIDKSAYIDMLANFTGSHQAWIRATEKVVSRCLNANNLERRSDCLINDVLACTLDVLTE
YCPYKRKSGICKHGKHDVPCQISSSKSRPKNRREICLLPELVHHDHLYECDLDALYKVERV
AVPVRHKKHVFPSLWHNCKQLETQTSCIMDKMGILNRYKFMDYFKMKDKIRQFTEDKPE
WSAMMDIYTSAYINLPMYSDHCTSERKLLNVIDTMLMTCPVSKRKNTPQCNSLFMEMVK
ATPADNQTVTKEKAXXNYEALPPRXXVSDR
>SlitOBP25
EDANSIFHAPIKPIVIDCAKEYGLSEDDIKKNRGLDGLKNLPPCFIRCVLNKLDIINDKGQY
DADSGIATIKGLMSNNEYLEKISGVLKECESVNEKSVSDGDAGCERALLGAMCYLDHKTI
VLA
>SlitOBP24
NAEDKIAIMTAVKPFVEECAKKHGVTFEALLTAKASGKIDGVEPCFYSCVYKKTEFLNSKG
EYDVDTALAKLKKYISNDDDYAKLSQVGKRCASVNSKPVGDGEAGCERGVLLTQCFLDH

KGSVPM
>SlitOBP28
RTDQEIKAWFFREGMDCNIEHPISXKEMLELKENKIPDTNNAKCFVACVFKKTGMLDSXG
MFDAENSIAMTQKDFANDPNRLESSKKLLETCKKVNDETVSDGEKGCERSVLLHKCFVE
TAPQLGIKLP
>SlitOBP29
GLTEEELKMEFTKLIMKCNKDGEVDMTELVQLQNYVVPTKQSTKCVLACAYKAAEVMN
AKGEYDIDHAYKVAEMMKNGDEKRLVNAKKMADLCVKVNELSVSDGEKGCDRAAMIF
KCTVENAPKFGFKL
>SlitOBP30
HKFSAIQIPYNVLSRDDSNYLFVNNFNVVITKNYYETNNSKQALSMDDLKQKYVDNILEC
SKQYPIDRADAEQLQNRIMPDKEPIKCLFACVYKLAGMMNDQGELSVEGVNAISRKYLA
EDPEKLQKSEEFTEACRSVNDAPVSDGTRGCDRAALIFKCTIEKSPEFNFV
>SlitOBP27
QLFNSGVVFCIIVAVFLKNASAITDELKAHIEAKFLTVGAECIKEHPLTIEDLSAFKNRVFPD
GENAGCFSACIFNKLGLFDDKGTLSHLTALENAKKVFEDQGELESIEKFLTTCAKVNDEEV
SDGEKGCERAKLAYNCFIPKTIEQLGFDL
>SlitOBP23
MAKLSCLVLCVVAASLGSIHVAKGESLRESLRPVIVACSKEHGVTDAEIQAAKEAGSPASIK
PCFIACVFKKAGFINEQGQLDLETGLKNLRQFVKDDEQYKQLEGVANKCSPVKDKAVSD
GAAGCDRGVHLAGCFLDHKTSIII
>SexiPBP1
SQELMMKMTKGFTKVVDDCKAELNAGEHIMQDMYNYWREDYQLINRDLGCMILCMAK
KLDLMEDQKMHHGKTEEFAKSHGADDEVAKKLVSIIHECEQQHAGIADDCMRVLEISKCF
RTKIHELKWAPNMEVIMEEVMTAV-
>SexiPBP2
SQDVMKNLAINFAKPLDDCKKEMDLPDSVTTDFYNFWKEGYELTNRQTGCAILCLSSKLE
ILDQELNLHHGRAQEFAMKHGADETMAKQIVDMIHTCAQSTPDVAADPCMKTLNVAKCF
KLKIHELNWAPSMELIVGEVLAEV-
>SexiPBP3
SKDAMKYITSGFVKVLEECKQELNMNDHIIADLFHFWKLEYALLSRDTGCVIICMSKKLD
LLDANGRMHHGNAQEFAKRHGAGDDVASKIVQIIHDCEKKHERDDDECLRVLEVAKCFR
TGIHDLDWQPKVEVIVSEVLTEI-
>SexiGOBP1
DVNVMKDVTLGFGQALDKCRQESQLTEEKMEEFFHFWRDDFKFEHRELGCAIQCMSRH
YNLLTDSSRMHHDNTEQFIKSFPNGEVLARQMVELIHSCEKQYDHEDDHCWRILHVADCF
KQGCVQRGIAPSMEMMMTEFIMEAEAR-
>SexiGOBP2
TAEVMGTAEVMSHVTAHFGKALEECREESGLSAEVLEEFQHFWREDFEVVHRELGCAIIC
MSNKFSLLQDDTRMHHVNMHDYVKGFPNGHVLSEKLVELIHNCEKQFDSMTDDCERVV
KVAACFKVDAKAAGIAPEVAMIEAVMEKY-
>SexiOBP1
TAEEKAALIEAVKPYIQECSKEHGVTPEDIKSAKEAGNADGINACFLRCVYNKAGVINDK
GEYDADKALEKLKKFVSNEDDYAKFAEIGKKCASVTETSVSDGEAGCERAALLTSCFLEH

KSEVHA
>SexiOBP2
NEKGNKLASECIKETGVKNELLEEAKKGIISEDPAFKAFTYCFFKKIGIVGEDGLLNRDVAI
AKLPSGVDKSEAEKLLDSCKSKTGKDAVDTVFEIFKCYQQGTKSHIMFAS
>SexiOBP3
EDANSVFQGAIKPLIAECAKEYKLSDEELLKNRGLAGLSNLPPCFIGCVLKKFDIINDKGLY
DAEAGIAKIEKLLPNNEFLDKISGVLKSCESANEKSVGDGDAGCERAVLVATCYLEHKTAV
IA
>SexiOBP4
LTEEELKMEFTKLIMKCNKDGKVDMTELVQLQNYVVPTKQTTKCVLACAYKAAEVMNA
KGEYDIDHAYKVAEMMKNGDEKRLVNAKKMADLCVKVNEQSVSDGEKGCDRAAMIFK
CTVENAPKFGFKL
>SexiOBP5
MTMKQIRNTGKMMRKTCQPKNNVEDEKIDPIAEGVFIDEKEVKCYMACIMKMANTIKN
GKLNYDAAIKQGDLLLPDDIKEPAKEAITACKKVGDAHKDICDASFHITKCIYNHNPGIFY
SP
>SexiOBP6
LLIDDLKQKYADSILQCSQQYPLDRADAELLQNKVMPDKESTKCLFACVYKVTGVMSDQ
GELSVEGVNALSQKYLADDPEKLKKSEEFTEACRTVNDAPVSDGARGCDRAALIFKCTIE
KSPDFSFV
>SexiOBP7
ASTKEAMTTTMTDQVNSIEVDVLAVMDMCNDSYRIDPTYLQALNESGSFIDETDKTPKCF
IRCVFENVGIVSEDGMQLNPARAAVIFAGERNGKPMEDIADMTALCATDRQETCPCDRSY
KFLRCLMSMEIERYEKS
>SexiOBP8
DDSNDSEPDIDLDQIVMECSEKFDIPFPQIPAALRAANAHALKPCFWSCCFMKVGVINSKG
QYDVDFTLDLAKKMLGRQDEKKVEATVKDCESVNDAPVSDGNAGCERSLLLATCLLDN
CKKNFPEIFGH
>SexiOBP9
ITDEPKAHIEAKFLTVGAECIKEHPLTIEDLSAFKSKVFPDGENAGCFSACIFNKLGLFDDK
GTLSHLTALENAKKVFEDEEELENIEKFLTTCAKVNDEEVSDGEKGCERAKLAYNCFIQNY
EQLGFDLEF
>SexiOBP10
MTREQVKKTMAIVKNQCMPKNSVTEEQVGRIEQGVFIEDRNVMCYVACVYKTFQVVKN
DKLDMALITKQIDLLYPPELKEPVKKSVAACLHSQDNYNDLCERVFYGSKCLYEKDPNSFI
FP
>SexiOBP11
MTDEQKALIHEHFEAIGKSCNKDSHIITADDIADLRARKIPSGPNAPCFLACMMKEIGVMD
DKGMVQKETALEMARAVFDDPEEVKAIEDYLHSCSHINTESVSDGAAGCERAMLAYKC
MTENASKFGFDI
>SexiOBP12
MTREQIKNSGKLIKKTCSAKNDLTEDEVKDVDKGKFIEKKDFMCYIACVYKMGQSVKGS
TINHDMMLRQVDMMFPNDMKAPVKAAIEHCRPVAKNYKDFCEASYWTAKCIYDFDPAN
FMFP

>SexiOBP13
ESLRESLRPVIVACSKEHEVTDAQIQAANDAGSPASIKPCFIACVLKKAGFISEQGEYDLDA
GLKNLRQFVKDDEQYKKLEEVAKQCASVSKKAVSDGTAGCERSALLAGCFLDHKASIII
>SexiOBP14
MAIYSLLPSARTDQEIKAWFFREGMDCNNEHPLSPKEMLELKENKIPDTNSAKCFVACVF
KKTGMLDSKGMFDADSSIAMTQKDFADDPKKLESSKKLLEACKKVNDEAVSDGEKGCD
RSVLLHKCFVETAPQLGIKLP
>SexiOBP15
MSRQQLKNSGKMLKKNCMNKIGVTEDQVGSIDKGKFIEDRKVMCYIACIYELTNVIKNN
KLNYEASIKQIDLMYPPDIKESAKAAVEKCKDVQKKYKDICEVSFYAAKCMYEFKPEDFIF
A-
>SexiOBP16
GKDKPVFSDEIKEIIQTVHDECVAKTGVAEEDITNCENGIFKEDAKLKCYMFCLLEEASLV
DDDDTVDYDMLVSLIPDEYYERTTKMIFACKHLDTPDKDRCQRAFEVHKCSYEKDPDLYF
LF-
>SexiOBP17
DVVETPTKREIKASLKPLSVCCDIPELADDRQLVKCSNPKPPGPCEDVQCIFEVSGFLTDKN
TLNKAAYRSHLQKWEKNHPGWTDAIYKAITDCVDNDPRQHLDVACKAYDVFTCTGIAM
LKKCPEAAWKC-
>SexiOBP18
APNKASGTFCGLTPNNMFKCLNNPRVLNLEAAAKCTSQVTECEKITCVFREQKWSKHGVI
DKAKIRAHFEQYETEHPEWAPAVQHVKAFCLAPELRAQGVFLNCPAYDIMQCALASFIKH
ASPSVWSTEQNCDYPKAYAADCPVCPSDCYSAAIPIGSCNACYLQPRTV-
>SexiOBP19
QQPDFQPGPPGPPGPHHKGPPPGAFPPGIPKSCWVPPREVNLFKCCPIPPLYSDEVMQSCGF
EKPSEDGPKPPKRHRRPDGTCKEGYCVMGNADLLQTNNSVDYEKFRSYLDNWAASNPDF
AEAIQIAKEDCAQDGGPAGPPVCEPDRLFFCLTSKIFWNCKLRDEDGCQALQQHMDECRQ
YYTKPKEQIEGNPERR-
>SexiOBP20
PSKNEDEKEPDWSYANFPKEVSEHVEKFKKNMTECLKEVQTIDKRPVKRLSPKMESPVH
GECLIACVLKRNGVIINGKVNKDNLISLVSKFYSKDTRLMKKLEKHLDRCIEMSVRFPGRL
HVGITTKRLHKTTLMASNKHKIMVNY-
>SexiOBP21
MYAHEKLSDIVAEQCLSQMYPKNKHIQIQESDEPCIIFCVLKKFGIISPNGVFNLEVYRKRV
QI
>SexiOBP22
ATTGCKNCIMLGKEEKAMFRAHSEACLATSRVEPRLVDAMLAGELLDEPALRKHVYCVL
LKCKLISKDGKLQKAAVLGKMAARPDAKNATKVLESCADQTGDTPEDLAWNLFRCGYD
KKALLFDYMPTNGANESDNNS-
>SexiOBP24
DSAISADSESRCRNPPTAPQKIERVITLCQDEIKLSILREALDVIKEEHTMPAQRRRDKREVP
FTHDEKRIAGCLLQCVYRKVKAVDGYGFPTLEGLVGLYSDGVNERGYFMAVLEASRECL
MKNHDKFSRTVPMDNGRNCDVSFDIFECISDRIGEYCGTSGL-
>SexiOBP26

VTEEEKEAFREAMAPIIAECSEEHGVSEADIKAAKEAASADGIKPCFLGCVMKKIEVLDSK
GLYDAETGLGKLKKFVKDDDEFAKFEDIAKKCLKVNDESVSDGEAGCDRAKLVLGCFLE
HKVEMPF-
>SexiOBP27
MYKIICLIFCVVVSLNSVHGNVEDKIAIMSAMKPIVDECAKKHGVTLEALLAAKASGKID
GIEPCFYSCVYKKTEFLNSKGEYDVDNSLVKLKSTLA
>SexiOBP28
LTDEQKEKLKKHRTECLSETKVDEQLVNKLKGGDYKTESEPLKKYALCMMMKSELMTK
EGKFKKDVALAKVPNPADKPTVEKLIDACLANKGNTPHQTAWNYVKCYHEKDPKHAIFL
-
>SexiOBP29
ASSNNNINKNNTRRTRRNEPLLNRPDSDQCLSQCVFANLQVVDSRGIPREAELWNKVQTS
VTSQQSRSALHDQIRACFQELQSEAEDNGCSYSNKLERCLMLRFSDRKVDGKGNPKKSST
EQT-
>SexiABPx
AVEMDEDMAELARMVRENCAGETGVDVALVEQVNAGAELMPDDKLKCYIKCTMETAG
MMADGEVDKEAVLAPLSHNAPALKACGTQRGADHCDTAFRTQQCWQNANRADYFLI
>SexiABP2
MSGDEEAGVRDALRPYVQECADEYGITEEQFEEAKKKASADDIDPCFMSCFLKKAEFFD
AQGKFDVDSTMAFAKEHLSSEPAMKFVEAVGDECVKINDEDVSDGDKGCDRAKLLFDCI
AETKKKMD-
>BmorOBP28
TTVLAEDSRKLVSFAPEVAKKLKVLIQECLNENGLGEDAIEVIRAGEYREDEPFQNLVYCA
YKKFGALDENNRIISQVAAASFPKDIDVVTVIESCGKEDGNTPVDQVFKYFKCFQKNSPVR
MQLY
>BmorOBP22
TQKVAVSFPKDKTTIVVEAMKSIAKTGANPNVIEVISSGKVSEDEKFKEFFYACNDIGVVNP
DGHIKVKECIELFPKETQPLVEPVIKNDKEGVNKYDTLFKYLKFQETSPVRVTLA
>BmorOBP26
DNVHLAETQKEKAKQYTSEVRESGVSTEAINAAKIGKYSKDKAFKNFVLFFNSAIFNSDG
TLNMDVALAKLPPGVNKSEAQSVLKQCKNKTGQGAADKFEIFRYYKGTKTHILF
>BmorOBP27
DNVHLTETQKEKAKQYTSEVKESGVSTEVINAAKTGQYSEDKAFKKFVLFFNSAILNSDG
TLNMDVALAKLPPGVNKSEAQSVLEQKDKTGQDAADKFEIFQYYKGTKTHILF
>BmorOBP25
DNVHLNEDEREKANWYTAEGVETGVSTEVINAAKIGKYSKDKAFKKFVLFFKSAILNSD
GTLNMVVALAKLPSGVNKSEAQSVLEQKNKTGQDAADKFAILQFHKGTKTHILF
>BmorOBP23
EDSRKLVSFAPEVAKKLKVLIQELNENGLGEDAIEVIRAGEYREDEPFQNLVYAYKFGALD
ENNRIISQVAAASFPKDIDVVTVIESGKEDGNTPVEQVFKYFKFQKNSPVRMQLY
>BmorOBP30
EDYYYDIVTRDPDDLMREKENEVRALRAFQADAEDVQVKPDLVVNLKSGDWQTEDVSL
KKWALVLMLGLMTAQGVFKMNEAMSKIPDMNDKIIAEKLIDDLSLQATTPHDAWNYIKH
HQKDPEGNFSSLNIF

>BmorOBP31
LTDEQKENLKKHRADLSETKADEQLVNKLKTGDFKTENEPLKKYALMLISQLMTKDGKF
KKDVALAKVPNAEDKLKVEKLIDALANKGNSPHQTWNYVKYHEKDPKHALFL
>BmorOBP29
TTGCKNCVILGKEERAMFRSHSDALAQSRVEPRLLESMMNGELIDDAALRKHVYVLLSC
KMIGKDGKLLKAAILGKLAARPAGRDVTKVLEAAEQPGASPEDVWNIFRGYNRKAVLFD
YMPAGGASSGNTENHP
>BmorOBP14
MSRQQLKNSGKMLKKQMGKNDVTEEEIGDIEKGKFIEQKNVMYIAIYQMTQIIKNNKISY
EASIKQIDLMYPPELKESAKASAGRKDVSKKYKDIEASYWTAKMYEDNPKDFIFA
>BmorOBP17
MTRQQLKNSGKIMKKTMPKNDVTEEEIGQIEQGKFLEQRNVMYIAIYTVTQVVKNNKLS
YDAVIKQVDVMFPAEMRPAVKAAAENCKDISKTFKDIEASYWTAKMYDFDPKNFVFP
>BmorOBP15MTKQQIKNSGKILKKAISKNDVTEDQISDIDKGKFIEDKNVMYIAVYSMSQV
VKNNKFVHDAMVKQVDMMFPTEMRDAVKASIANCRGVAKNYKDIEASFWTAKCMYEF
DPANFVFA
>BmorOBP16
MTRAQVKKTMTIMKNQMPKNGVTEDQVGKIEEGIFLENHNVMYIAVYKTIQVVKNDRL
DKDLISKQIDVLYPQEIRESTKKAVGDINLQEKYDDWEGIFRSTKLYEKDPANFIFP
>BmorOBP18
MTMKQIKNTGKMMRKSQPKNNVDDEKINPINDGVFIEENEVKYIAIMKMANTMKNGKL
NFEAAMKQADLLLPDEMKEPTKEAIVARKVADSYKDVDASFHVTKIYNHNPSVFFFP
>BmorOBP21
GKDKPVLSEEIKEIIQTVHDEVGKTGVSEEDITNCESGIFKEDVKLKYMFLLEEAGLVNDD
GTVDYEMFTSLIPEEYFDRATKMIFSKELDTPDKDKERFEVHKSYEKDPDFYFLF
>BmorOBP20
QLDDEIAELAAMVRENADESSVDLNLVEKVNAGTDLATITDGKLKYIKTMETAGMMSDG
VVDVEAVLSLLPDSLKTKNEASLKKDTQKGSDDDTYLTQIWQAANKADYFLI
>BmorOBP12
LTDEQKNKIQSKFIEIGAEIVEHPISIDDINSFKNKKFPSGVNAGFVAIFNIGLFDDKGNLSHN
SALEKAKGIFNADEEVKNLEEFLNRAKVNGEAVGDGVKGERKLAYNLIENSLEFGFNIDF
>BmorOBP11
LTEEQKAEITKSSLPLIAESKEFSVNQGDIDAAKKLGDPSGLNSFVGFMKAGIINASGLFDV
AATIEKSKKYLTSEEDLKAFEKLTETAPENDKPVSDSDKGERKLLLDFVANKGSFSVFSL
>BmorOBP13
VTPEESKAFEAFAKPVIEQQKDFGMDKESFAQKNLDEIDECLIAVVEFGITNDEKIDGDAL
KALVTKFVGNEEERNKINKIVEETEDANKSGDGTNTSTILFLLLKNGKDLWGF
>BmorOBP8
SSLDDLKMVYKNVIKEVGDYPITAADLKLIKARQIPNDDIKVFAAYKTGMMTEEGMLSVE
GIKDMSQKYLSDNPEQLRKSKEFAEASSVNDQQVSDGTKGERALIFKSTEKITNFGFEL
>BmorOBP9
VSYEQKIKIRDQLDRAGFEFKDHKITEDDIKNLRANKPATGENVPFIAVMKTGVMNDQGVI
RKGPVLELAKKVLADDKDIKKLQDYIHSSHVNSETVHDKGKGEFMQAYTMSANASKFGF
NI

>BmorOBP10
VSDEQKIKIREQIDKSGFEFKDHKITEDDIKNLRARKPATGENVPFIAVMKTGVMNDQGVI
HTEPVLQLAKKVLTDDKDIKKLQDYIHSSHVNSKTVHDKGQGEFIQTYTMSANASKFGFD
V
>BmorOBP7
AVTEEELKIEFTKLVMKTKDHPVDMSELMQLQQLIAPKKTESKLLAAYKLNGVMTSQGL
YNLEHAYKIAEMSKNGDEKRLENGKKVADIVKVNDVEVSDGEKGERALIFKTLENAPKV
FKFGSSEYNCQ
>BmorOBP19
AKTDVEIKAWFLGQAVESKDHPVTTEELRMHKHELPDSKNAKLMKVFRCNWLDSKGMY
DINAAYASSTKDFSDDKTKQENANKLFDTKSVNEENVGDGEEGDRSLLLAKLTKAAPQVS
IYYS
>BmorGOBP1
DVYVMKDVTLGFGQALEQREESQLTEEKMEEFFHFWNDDFKFEHRELGAIQMSRHFNLL
TDSSRMHHENTDKFIKSFPNGEILSQKMIDMIHTCEKKFDSEPDHWRILRVAEFKDACNKS
GLAPSMELILAEFIMESEADK
>BmorGOBP2
TAEVMSHVTAHFGKTLEEREESGLSVDILDEFKHFWSDDFDVVHRELGAIIMSNFSLMDD
DVRMHHVNMDEYIKGFPNGQVLAEKMVKLIHNEKQFDTETDDTRVVKVAAFKKDSRKE
GIAPEVAMIEAVIEKY
>BmorPBP1
SQEVMKNLSLNFGKALDEKKEMTLTDAINEDFYNFWKEGYEIKNRETGAIMLSTLNMLD
PEGNLHHGNAMEFAKKHGADETMAQQLIDIVHGEKSTPANDDKIWTLGVATFKAEIHKL
NWAPSMDVAVGEILAEV
>BmorPBP2
SRDVMTNLSIQFAKPLEAKKEMGLTETVLKDFYNFWIEDYEFTDRNTGAILMSKLELMDG
DYNLHHGKAHEFARKHGADETMAKQLVDLIHGSQSVATMPDEERTLKVAKFIAEIHKLK
WAPDVELLMAEVLNEVSWKS
>BmorPBP3
SSEAMRHIATGFIRVLDEKQELGLTDHILTDMYHFWKLDYSMMTRETGAIIMSKLDLIDGD
GKLHHGNAQAYALKHGAATEVAAKLVEVIHGEKLHESIDDQSRVLEVAKFRTGVHELHW
APKLDVIVGEVMTEI
>BmorOBP5
ESGVDVVKNLSLSFARFFLEDEERHFQPEVRLKVMTFWYSESSTWDRDVGAFLIFKMEID
NPQDPSYRTHLELLSFANSEDNKIANQMVEIFYAGENTETDPLWLEQVKYKNRINQLGLTP
TF
>BmorOBP32
YNTKLFSHSLGSEPSLSILYARDKKSDKVTNELMEMYPKNLYKYPLRIDRNDIPIIHVLKFGI
ISNDGFINIKNYYRRVQAIHRYDPRILISDVGETAQNINGMNLDHDVKKKVFNDTQLYAISY
REPEDW
>BmorOBP33
MYAHDKLSDMIADQLNEMYPRSKRLEIEESDEPIIFVLKFGIMSPTGVINLEAYRKRVQLPE
QLAQRNSINDFGSALESAEATQHKQDVKKKVFNETHLYKILLK
>BmorOBP34

SRTRGSSGTLVDFTDPKVQGHLDALVRMAQSVIKVRATPKDVRAYFTNSSPVSRSGQFAT
MLEQSDIINHGKVNRDLLVHLAGLVNGKNSRVVRKLNSVSRLLDSISGMTDRQLSTYNDL
NENMIEFAFPLDIAEEAVRKMPFHLIQPK
>BmorOBP35
GMSTHVLDFKRNMTELKEVQNNDKRPIKRLSPKQESPIHGELIAVLKNGVIQNGKVNKDN
LMALVSKFHAKETKLMKKLEKNLDRINISVKNHDESLSQLNDTNDIMASSKQKILFNY
>BmorOBP39
AISTDNEQRCKNPPTAPQKIERVITLQDEIKLSILREALDVIKEEHTMPAERKRNKREVPFTH
DEKRIAGLLQVYRVKAVDGFGFPTLEGLVGLYSDGVNERGYFMAVLEASRELMKNHDKF
SRTTPMDNGRNDVSFDIFEISDRIGEYGTSGL
>BmorOBP38
KSTEAPKSKTALFNDQDNMGYEELDMEEIMSANESFRIEYALESLNDSGSFPDETDKTPKY
IRVLETEILSENGVLNPATAALVFAGERNGKPMSDLEEMAVAADRHEKCKEKYNFVKLMY
MEIDKYEKKN
>BmorOBP36
FKPLTKDEHIERYNKMNEDIEPFRKNLTEARQVKASMADVEKFLKRIPQSNMEGKFVAILK
RNSLIKNNKLSQENLLEVNRAVYGDDSEVMSRLKTAILESKIVEDIFEIEYSVFNDMHMKM
EHILDKITMERRMEALGQMSSNPDEWSEEEDEMLKLVKDEL
>BmorOBP37
EPEKENHFTLALKKTLFSTARSMSHVNANETDLEYLRKDPPFPDKAAIIKLLEIGVVKNNK
YSKMGFLTAVSPLVFTNKKKLDHYKSVSENEKEINHDQTTEELGNEVVSIFKYAPELHFKT
>BmorOBP40
MSEFIQPSWRTQNFRLNWDNRNRLSIDISHGAATTQTPVPTTKPKALRDFMVVPQSDKTT
VFKLNIVSDKGVVDVKSFIKLLDKFTNSYPVWNSAKARVITTLRKSLIAYDGGELNNILAT
FDVLSENPLNGNNQTC
>BmorOBP41
GNIPEQPRVYCGELPNTIYSLGNPKIIQPEVSEKCNKPISEDKTRIFKESGWAKNNVIDKKK
VSDYFEQFAKDNPDWSAAVQNFKTTLSDSLKPQGVDTNPAYDIIHALISFIFASPSQWSTSE
QCVYPRQYAGACPVCPERCFAPSVPNGSCNACLALLRTP
>BmorOBP42
EDPPGLPPFLKDAPEKCKSPPRVKNPNECISEPFFKEADFIECGIEKPGSERGPPDSKQNLLK
YNLLKNDETPDIEAIKSLLDKYIEKNPSFKSSVEKAKELREDLPGPPQILANRMTLIGTVLL
MEPDEKWNTTDDCKAFKDHMTECQKYFPK
>BmorOBP43
AKATLKPISACNIPELGNPEPLAECSNPKLPGPKDIQVFESGFLTENKTLIKEAYKTHLRQW
AKEHEGWSVAVEKAISDVDKDLRQYLEFPSAYDVFTTGIAMLKKPNEHWTC
>BmorOBP44
EFIINLYFNFITEIDSCVKKYPKLFDSEFITECYNTQRKANDKERDMVARLNLLTEEDSINKD
ALLRFVEEGFKTEIDLVNAIKKKFEEDISNIGKPEMEVKYKIITSRMAEDPKWDSKGICSSA
QQKVENFMKMLS
>HarmOBP1
SDEDKAKLHEALKPLVEECMKDHEVSLDDLKAAKEAKSADGVKPCFLACVYKKAEVLN
DKGEFDADHALEKLKEFVSDEDVLAKVAEVGNTCKAVNDKAVSDGDAGCERAALLTACF
LEHKAEILV

>HarmOBP2
MSRQQLKNSGKMLKKNCMNKNQVTEDQIGSIDKGKFVEDKKVMCYIACIFEMTNVVKN
NKLNYDASIKQIDLMYPPDLKESAKAAVEKCKDVQKKYKDICEASYWTAKCMYDFKPED
FIFA
>HarmOBP3
NALEKAAIRAAVYPLIVDCAKEHGVTLEQLKAAKASHSAEGINPCFQSCVYKKTGIFNDN
GEYDVANAKTKLQKFVTDEDEYARIAEVGKTCASVNDKSVSDGAAGCERAALLTACFLE
HRAQIII
>HarmOBP4
DDETRASFRQVLGPLVMECRNEFGITEDDLKKAQQERSPDALKPCFIACVFKKFGIITSAG
KYDSDASISRIKDVVKNDDLLAKLKSVGEKCNSVNDASVSDGDAGCERAALLAKCFIEN
KSELSI
>HarmOBP5
SEEEKAAFREAIKPIVEECSKEHGVSHDELKSAKDNQNADSIKPCFLGCVYKKAEVFNSK
GEYDVDKALEKLKKFVSNDEAYAKFAEVGKKCASVNDKAVSDGDAGCERGALLTACFLE
HKAEVPL
>HarmOBP6
TEEEKEAIRAAVRPIMQECGKEHGVTLDDLKAAKAAHSADGIKPCFQSCVYKKAGIFNDN
GEYDIANAKTKLQKFVTNDEEYARIAEVGKMCASVNDKPVTDGAAGCDRAALLTACFLE
HRAQIII
>HarmOBP7
LSSEEELSIKEALHPFVVECAEEYGMTEEMFEEAKKKGSAEDIDPCFMSCFLKKTGFFDDS
GKFDAEKSISFAKEHITSESAIKFLEAGAGECVKINDEDVSDGENGCDRAKLLFDCLTELK
KKMSE
>HarmOBP8
MTMKQIRNTGKMMRKSCQPKNNVADEQIDPIAEGVFNEDKEVKCYMACIMKMANTIKN
GKLNYEAAIKQADLLLPDDIKEPAKEAITACRKVADAYKDICDASFHITKCIYTQNPGIFYF
P
>HarmOBP9
ISEEDKAAIITAIAPLAQNCGSECGLDNDDFEKYKEDGSDMDPCFKACLMTQMGVLDKEG
KYDGKGLHKAMEEADYPGDKDDAQKFLDELDRCFDAKGDNSGSDEEAKMKRADVLFR
CMQDMKEK
>HarmOBP10
LTEEELKLKFTKLIMKCNKDSEVDMQELVQLQSYVVPTKTATKCVLACAYKAASVMNAQ
GLYDIDHAYKVAEMMKNGDEKRLTNAKKMTDICVKVNDIKVSDGEKGCDRAALIFKCTV
ENAPKFGFKL
>HarmOBP12
QGVLCGPLPRRLTRCLNMAPAISGEIQDKCHESRTATECERLTCIFREYNLLDGTTVNKDRT
NAFLDNYVKQYPVWTTAVQHAKAACLGDVELKPQGIDLNCPIYDTLQCIFSSLIKHATPSQ
WSTTSECQGYRAFAAACPICPEDCFAAQVPIGSCNACLSLP
>HarmOBP13
GKEKPVFSDEIKEIIQTVHDECVAKTGVAEEDITNCENGIFKEDPKLKCYMFCLMEEASLV
DDDDAVDYDMLVSLIPEEYVDRTTKMIFSCKHLDTPDKDKCQRAFEVHKCSYEKDPDLYF
LF

>HarmOBP15
EKPSKHPMPYITSRFVKVLEECQHELKLNEHILEHLFHFWKLEYSLLGKDPGCAIICMSTK
LDLLDLYGRMHRGNAAEFAKKHAAGDEVPSKIVTIIHFCQKKHEQDGDECLQVLEVATCC
RTGLHDLNWQHQVEVIVPDVLTEI
>HarmOBP16
DVLKKRDSKGASLKPLSVCCDIPELGDPKNLEKCSNPKMPGPCDDIQCIFEASGFLIDRNT
LNADAYKNHLMKWQEEHKPWKVAVDRAIEECANNQTRQYLDFPCKAYDVFTCTGIAML
KKCPEAAWKC
>HarmOBP17
SGLTDEQKEKLKKHRSECLTETKVDEQLVNKLKGGDYKTESEPLKKYALCMMMKSELMT
KDGKFKKDVALAKVPNAADKPTVEKLIDACLANKGNTPHQTAWDYVKCYHEKDPKHAI
FL
>HarmOBP18
AANVSLPPKQNEKANQIATECMKESGLKPEVLAEAKKGHISDDEHLKKFTFCFFKKAGIV
SEDGKLNTEVALAKLPPGVDKAEAEKLLETCKGKTGKDVTDTVFEIFKCYHHGTKTHILL
GF
>HarmOBP19
QEGPGGPPGDPRQHPILSKIPRKCWAPPPGIDIYRCCPIPKLYPDEIMEQCGIKRASGEGSEE
LEKIQPGPKVPCKEGICLMQKANLLQENNSVDYTKLRSFLDQWADTNAEFTDAILTAKKIC
AQDGGPAGPPVCEQDRIFFCLTSNILWNCNLRKLDGCDILQEHMDECRQYYVQDEPEE
>HarmPBP1
SQDVIKNLSMNFAKPLEDCKKEMDLPDSVTTDFYNFWKEGYEFTNRQTGCAILCLSSKLE
LLDQELKLHHGKAQEFAKKHGADDAMAKQLVDLIHGCAQSTPDVADDPCMKTLNVAKC
FKAKIHELNWAPSMELVVGEVLAEV
>HarmPBP2
SKELLTKMTGGFTKVVDACKTELSVGDHIMQDMYNFWREEYQLVNRDLGCMIMCMTAK
LDLIGDDQKMHHGKAEEFAKSHGADEALAKQLVGLIHGCETQHQAIEDHCSRALEIAKCF
RTKIHELKWAPSMEVIMEEIMTAA
>HarmPBP3
SKDAMQYITSGFVKVLEECKHELDLNEQILADLFHFWKLEYSLLGRDTGCAIICMSKKLD
LLDANGRMHHGNAAEFAKKHGAGDEVASKIVTIIHECEKKHEQDGDECLRVLEVAKCFR
TGIHELDWQPKVEVIVSEVLTEI
>HarmGOBP1
LLADINVMKDVTLGFGQALDKCREESQLTEEKMEEFFHFWRDDFKFEHRELGCAIQCMS
RHFNLLTDSSRMLHDNAEKFIQSFPNGEVLARQMVELIHSCEKQFDHEDDHCWRILHVAE
CFKGSCVQRGIAPSMELMMTELIMEAESR
>HarmGOBP2
TAEVMSHVTAHFGKALEECREESGLSAEVLEEFQHFWREDFEVVHRELGCAIICMSNKFS
LLQDDSRMHHVNMHDYVKSFPNGHVLSEKLVELIHNCEKKYDTMTDDCDRVVKVAACF
KVDAKAAGIAPEVAMIEAVMEKY
>HyphOBP1
EQESSEQCFMFKKNVLNCCTKPLKPRIGPKPEIKECLTDKPAPNCETEKCIAKKLGYYGSD
DKIDMEALRNYIEKDLEDNQDLLDMIKKNCLNTESTDVCGFKQVHLCMLKQSIDSCQEW
KTEAPCDENKIKEL

>HyphOBP2
LSDETKTAFQEAIKPLIEECSEAHGISMDEIKAAKEAGTSSGLKPCFLGCVLKKGNILNDKG
EYDVDAALKVIKKYISDEEEFSKFAEVGKKCASINDEAVPDGAEGCERAKLLLSCIHQNKD
GIL
>HyphOBP3
GVDDDTDGMMTAVNHMMQECVQELGIPYENHKKFSWDVDYDPCFLNCFYEKHGVMNK
GKFDKNNALEFAKKYIKDETTLAQIKESNEKCNKVNDVDISDGDVSCKRA
>HyphOBP4
LTEDDLKAELTKLVMKCNSNSELDMKDLQQLQEFVTPTKTATKCVLACAYKEAQIMTKD
GLYNLEHAYKVAEKLKNGDEQRVVNGKKMADVCVKVNDEQVSDGEKGCERAALIFKCT
VDNARKFGFKL
>HyphOBP5
ATMGCKNCIILGKEEKAMFRAHSDACLPVSHVDQRLVDAMLSGELIDELPLRKHVYCVLL
KCKLISKDGKLQKYAVLGKMSAKPDAKNATKVLESCAEQTGDTPEDLAWNLFRCGYDK
KALLFKYMPTSTASGDIDNSN
>HyphOBP6
EPDHPMKKCHKLHHDMMRCCTGITTNKITQSDEFKECFEKMKPGPPSPFSCDFDDCLAKK
FGFYGEDGKLDKEALTSNIEKKYENNKEVVIAMKENCINGDISKYGPPDMCDMRKMKHC
FDNQLIKNCPEWSNEDECNGIQDLIADCIHDD
>HyphOBP7
DPEMAELARMVRDNCAGETGVDIALVEKVNGGAGLMPDAKLKCYIKCTMETAGMMSE
GEVDVEAVLAMLPPDMSAHNAPALRACGTQRGADDCETAYLTQVCWQNANKADYFLI
>HyphOBP8
MTRQQLKNSGKLMKKSCMPKNDVTEDDVGQIEQGKFLENRNVMCYIACIYTMTQVVKN
NKLSYDAVIKQVDVMFPVEMRNAVKAAATHCKDVAKKYKDLCEASYWTAKCMYDFDA
ANFVFP
>HyphOBP9
VTDEEKAAFHEAMEPIITACSAEHSVSEGDIEAAKELNYENGIKPCFIGCIMKKTELLDSNG
LYDAEKGLSKLRPFVKSDEDFAKFEAVAKKCVKVNDEPVSDGAAGCERAKLIMACAIENK
AEVKNIPLLL
>HyphOBP10
GKDKPIFSDEIKEIIQTVHDECVDKTGVSEEDITNCENGIFKEDPKLKCYMFCLMEEASLVD
DDDTVDYDMLVSLIPEEYFERTSKMIFACKHLDTPDKDKCQSAFDVHKCSYQNDPDLYFL
F
>HyphOBP11
MFPDGRNPNCFTTCVFKKIGIMDENGMLSPQEAIKKAELLLKENETHLNMDEELMRICSF
VNEPIDDGPEGCDRAKLIYECFIENGPRYGLKLHVN
>HyphOBP12
DGNIRILEDEIAVALKACTYPNDNTKQKEVIKERRRRSESLDDVSAKIDDETKQINQYNQE
KRHSGSREQIHVLNATDHDYEGYGSGNMRERLGNRSTNTNKTKRSDPLLYKADTDQCLS
QCVFANLQVVDSRGIPREAELWNKVQSSVTSQQSRSALRDQIRACFQELQTEAEDNGCSY
SNKLERCLMLRISDRKKDSQGSTEKPGS
>HyphOBP13
MSRQQMKKTGKMLKASCIKKIKVPEDMVGDIEKGVFIEDPKVMCYIACIFEMTNVIKNN

KLNYEASLKQIDLMYPPELKESAKGAAANCKDIQKKYKDICEASFWSAKCMYEFKPEDFI
FA
>HyphOBP14
ALNSNEEDEMFKNGLPYVMECLEAHGLSLEKIEENMENPPDGYYCFLDCFYKKLKILDD
QSMFNLDQVIKTMKINAKDEDDFSKIEETAKKCEKVNEVKIPDGDKNCERAKLLSECLDE
YEGVLE
>HyphOBP15
ANTMTKDLGSATTKYQEARSNEDYNLDMQRIMNECNDTFRIEMTYLESLNQTGSFSDEG
DKTPKCYIRCMLENMGIMSSEDGMLNPARAAVLFAGDRNGKAMEDLGGLTAICADRRES
CKCERAYQFMRCLMSMEIEKYESNQ
>HyphOBP16
KTDEEIRVWFFRQALECNKDHPVAKEEMQMLQEYKVPDTKNSKCLMACVFQKANYMDS
KGMFDLEGATAHVAKDYEGDATKLENSKKLMEICNKVNSETVGDGEAGCERAFLLFQCF
NENAPQLGFTIHTKA
>HyphOBP17
LNCRSDGGPKESELKNIYKKCLKMQQEVKNSTKGNSDQDWKESRSQLHSNNWERGRMG
NKENRNNRDDKMSNRGKMNTRDDVMGSNGDQMGGSNDRMGNKRDKMGGNSDRSSS
RNDRMGSSSDRIGYSDRMEGRKDRYDYTDSSHDDRFEENDYFKGREDFPQSGEYGTVSY
RRT
>HyphOBP18
KTDEEILTWLFRQAVECSAQYPITPEEMQMLQKQKMPNSKNARCLIGCVFKKAKFMDDK
GMFDEEGAIAQAKITHEGEPTRIENSKKLMEICKKVNSEPVEDGEAGCDRARLIFKCFNEN
ARKVVYLYIV
>HyphOBP19
HTVQLSQEQKDKANQHVAECIKETGIKTEVVAEAKKGQFSEDESLKKFTFCFFEKAGIVD
NEGKLNVATALAKLPAGVDKADAEKLLESCKNKTGKDAAERAFEIFKCYRQGTKSHIFLG
F
>HyphOBP20
FYCLNLNEESAVAYELKPLVSNCTEDCDIKEDLNAEKINPCFKKCLLVKIGLINENGKYNRS
EVKRQINNYTDNENEAQMILEAVDKCFKGKSGDQTEEEQINAVYECFMSF
>HyphOBP21
DSAISDNEARCRNPPTAPQKIERVITLCQDEIKLSILREALDVIKEEHTMPAQRRRDKREVPF
THDEKRIAGCLLQCVYRKVKAVDGFGFPTLEGLVGLYSDGVNERGYFMAVLEASRECLM
KNHDKFSRTIPMDNGRNCDVSFDIFECISDRIGEYCGSSGL
>HyphOBP22
ITEEELTALRLAVAPIISECAAEYEVSRNDIDAAKQNRDVDAVASCFIACGFKKIGVLDSNGL
YDAEKVSDKLKLFVQNDEDFAKFESIAQTCAKVNDEPVDDGAKGCERAKQILACAFEHR
DEMPF
>HyphOBP23
TPTPKPVLCGYTPDKLLTCLKNPHVVSQDVISSCRSDQITECDVMTCAFRKYKWLKAGKI
DKTKVVSHLNQLAKDKPEWKNVLEYAVTNCVERDLPPQGVHLGCPAYDILHCVLVSFIKK
TEAAQWDSSPICETPRQYAGACPVCPSDCFAPAIPYGSCNACYGAAPPAA
>HyphOBP24
QVSNIEELKQKYVEMILECSGDFPITNADLDLLKNKKMPDNQSIKCLFACVYKKAGMMN

DKGELWIEGVNEMTRKYLSNDLIRMKKSEEFTAACKNVNDVPVSDGAKGCERAALIFKC
TVEKAPEFDFV
>HyphOBP25
MTPEQKAQIHAHFKSIGQTCIKDNFISEDDIASLRSRKVPAGVNAPCFLACMMRQIGVMD
DSGVLQKETALEMAKSVFNEPEEIKNIEDYLHSCTSVNQEAVSDGEKGCERAMLAYKCMI
TNAPQFGLDF
>HyphOBP26
VTDEEKDVFRKAMEPIIAACSTEHSVSEGDIAAAKELHYSDNMKPCFIGCVMKKTEILDSN
GLYDAEKGLSKLRPFVKSDEDFAKFESVAKKCLKVND
>HyphOBP27
LPVYYAFMANHCTEYNEVLHVDTPNLEEAISPLEQTMIRTKMIALAIECVQSNPLTFDEIKS
FKNNVVPDKENAKCFTACLYKKMGIMDDMGMLSPARARKQAEKVFKDEEKLNNVTKIM
EDCLYVNELHEEDSKGCDRAKEAFSCLIKTGPKALSDETK
>HyphPBP1
SQDVIKQMAINFVKPLEACKKEMDIPETVIQDFYNFWKEGYELTNRQMGCAILCMSSKLE
LIDGEMNLHHGNAQEFAKKHGADDAMAKQLTDIIHNCVQTSPEAPDDPCQKTLNTVICFK
AEIHKLNWAPNPELLVGELLAETK
>HyphPBP2
SQEVMKKLTGGFAKVLESCKNELTIGDSIVQEFYNYWREDYALVNRDLGCMIICMASKLD
LLTDEMKMHHNNAHEFAKAHGADDDTAKQLVSIFRECESAPTTEDDPCLRALEFSKCFRS
RIHELKWAPSMEVVLEEVMTGVKT
>HyphPBP3
MIVFIDFSFKMEASKICLSFILLAISIRRTEPSKDGMKYISSGFVKVLEECKHELNLPDHIIGD
LFHFWKLEYDHLNRDTGCIIICMSKKLDLIDASGKLHHGNAKEFAMKHGAEEEIASKLVS
ALHGCETKNEGNADECLRALEMAKCFRTLVHDLEWSPKVEYIVEEVLTEV
> BmorIR21a
MDRRSLYGLLFIFYIISSQEIISYHSESLLKNASRNLLWNKKITSIIKEHNDFAYHYESDLHFG
NRIKNVKSKRAVDPVFHGHPKTREELWYERFLNRSSVFDQTPSLIKLIQNITLTYLNECTPV
ILYDSQIKLKESYLFQNLLRNFPVSFVHGYINEHSQLQEPKLLQPVRECLHFIIFLSDVKVSA
KVLGKQSESKVVVVARSSQWAVHEFLSSSFSRGFINLVVIGQSFKEDDDSTIESPYILYTHK
LYTDGLGASKPVVLNSWSHGKFSRNVNLFPPKMTGGYAGHRVVVAAANQPPFVFRRIKS
DLDGGNPRVVWDGIEIRLLHLLAEKNNFSIEIVEPQELHLGSGDAVAKEIAKGRADIGVAG
MYLTIDRTREMDVTFAHSQDCAVFITLMSTALPRYLLIVFIQFSIASFRLYLQNKYNFRYQAI
LGPFHWHVWVALTLTYLFGMFPLAFSDKHTLRHLINNSGEIENMFWYVFGTFTNCFTFLG
RNSWSKTDKITTRLLIGWYWIFTIIITSCYTGSIIAFVTLPMFPETVDTIHQLLAGFYRVGTL
DRGGWERWFLNSSDPNTNKLLKKLELVPNVEAGIMNTTKAFFWPYAFLGSKAELEYIVQS
NFTKTTSKRAVLHISNECFVPFGVTIGFPNNSLYTAKLNNDLRRMVQSGIVDKIVDEVRWE
MQRSSNGKLLSAVGGSLKVSAAEEKGLTLEDTQGMFLLLAAGFLIAATALISEWIGGFSKL
CRFRKKKNTLVNSSTKEDSINMPPTDSKDFKTETESVLHFCSRSTSPGSNESLDGQIINVTE
ESIEIHKQFTSEWDSRRSSSVDLEKEVKEIFERDLRRRGAALNNCQDVVLETRQSTASNNA
FGDAVK
> BmorIR25a
MRFACAFFFVLADFLGAFAEIYLRFVFIVERHEPELFRMIGEAVKHAEDLKQLALDDSIVSL
NRENEDEARGKLCSELSKGVSALIDLSWSPWEEAEHLASEAGVPIIRTALGPQPLVAAIDRY

LESRNATDAAILLESEIDVDKVLYELLGRSNTRIWVHAGLTRDSARALKTMRPDPSFYVLV
GGSGFVMETYKRAVKEKLVRRVYRWNLVFTDYLDLSALDLSSVVQPTMILQAHPEDCCRI
IASKDCTCSDSFEKTQATLNSLIAYLVDAFGRIDGTFMVKARVDCGDVHSDMSDTKEALH
RIFVEDIDNNETVFYWDQERSGLFLRHRYVLSSYKPDSGLEAVAVWSANQEYRLLPGVTL
EPLKHFFRVGTSPAVPWTLPKLDPETGDPLYNEDGQPIYEGYCVDLIQKLSEAMNFDYEIV
SPRSGGFGRRLPNGSWDGVVGDLTTGETDIAVAALTMTAEREEVIDFVAPYFEQTGILIAIR
KPIRKTSLFKFMTVLRTEVWLSIVAALVLTGFMIWLLEKYSPYSAKNNPGAYPYPCRDFTL
KESFWFALTSFTPQGGGEAPKALSGRTLVAAYWLFVVLMLATFTANLAAFLTVERMQTPV
SSLEQLARQSRINYTVVEGSSTHQYFINMKFAEDTLYRVWKEITLNATSDQAQYRVWDYPI
REQYGHILLAINASGPVADAETGFKQVNDHTDADFAFIHDSAEIKYEVTRNCNLTEVGELF
AEQPYAIAVQQGSRLQEDISRALLELQKERFLEQLASKYWNETLRQSCSDADESEGITLES
LGGVFIATLFGLGLAMITLAWEVFYYKRKEKNKVQSTKENVERPPIKSAKLGGKMAVGVA
RLRKRATKIGKKKNVTIGDSFKPSVSYISVYPKGDYRP
>BmorIR93a
MCNCKKVFFSTAVILDRQYLGDKYQTVLDELKDYIKELARVELKHGGVLVHYYSWTTISL
NKGFLAVFSIASCEDTWELFSRTEEEDLLLFALTEVDCPRLPQRSAITVTYSEPGEELPQLLL
DLRSSNAISWKSAVILHDDTLGRDMVSRVVQSLTSQIDEESARPVSVTVFKMKHEMNEYL
RRKEMHRVLSKLPVKYIGENFIAIVTSDVMTTMAEIARELLMSHTMAQWLYVISDTNAHA
SNLSGFINTLNEGENVAFIYNITENGPDCKNGLMCYSQEMMSAFISALDAAIQAEFDVAAQ
VSDEEWEAIRPSKVQRRDILLKHMQQYILAKSVCGNCTLWRALAADTWGVTYRQNDVP
EQINEHANGSTGVIEHLELMNVGIWRPIDAMTFADLLFPHVHHGFRGKELPIITYHNPPWT
FLQANESGAIVKYSGLMFDIVNQLAKNKNFTIKIMLPSHVKHEFSNETDMMHSQSARLTIA
AIAKGHAALAAAPFTVLPGPNQGINYTIPVSTQPHTFIVARPRELSRALLFLLPFTTDTWLC
LGFAVILMGPMLYIVHRLSPYYEAMEITREGGLATIHNCLWYIYGALLQQGGMYLPRADS
GRLVIGTWWLVVLVIVTTYSGNLVAFLTFPKLEAPVTTISELLKNSDAYTWSVTKGSYLEM
ELKNSEEPKYKRLIKEAELLKETGGIEGTIHAARGTLDRVRGQRHLIFDWRLRLTYLMSAD
HIATETCDFALAVEDFMEEQVAMIVPAGSPYLPVINKEINRMHKAGLISKWLSAYLPKPNR
CLKISTVTQEVSNHTVNLSDMQGSFFVLFLGFFSASTVLILEWFYNRRKRKSEEIVIKPYVE
>BmorIR75a
MPCTSLIINCLSQFDYFGIIIKLYYGRIDLYSTVDNNLLAKMYMEKKIRMSLHNLSHRKYDF
ETFMVPHNTSIGLLVDGECNHTKELYLEAAKLKLFDSTHSWLVFPDVQGPENDTEDFIDD
MFADVELYVNADIVVPSYVGDHYKLTDVYNFGRIQGNPLEKMVIGAWEQRAGLVIYNKT
FKYYQRWDFHNLTLRAVSVILEPPKDFYPEILTEMKYTPGIVRMTKIASLLLNILKEQHNFR
FNYTIRSRWIGSPERNSTPMVSNALLWGDQDIASTSGRIFPKWLDWMDIFYPPVTHLETKF
YYMIPDKGIGDYENRFLTPMSPAVWWCSLATAVVCIFGLAAAAMLERRPSPGIFAFFSVFAT
TCQQDFEDGAPLDETVSSQGRRAALLVVGLTSMLLYNYYTSSVVSWLLNAAAPSISDLDG
LMNSDFELIFEDIGYTRGWFDNPGLFYFSGFKNPKEDILRDKRVLHTKRKLPPLQPVEKGI
ELVRTGQFAYHTEPFTASQLISRTFKEQELCELGGLPIMLPANAYMYGQRRSPYKKFFVWS
LMRLLERGHIRAAEARFSGTIPPCSGRMPRSLALGQAAPAFVLLAAFTVLSVIIAMIERIWF
RYRFKTSRPTINRRSKN
>HrhoIR8a
MDFFVLFFILMIVNVIFVTSEISLRFVFILEHHDYELAEQIGNALKTVEESTPGVHLSDAVIF
LNREEDGESYRKLCSSVSTGVSMIINLSWAPWPAAEDMASSSGVPIIHTALGSQQLIKALD
DYLESRNASDAAYILESEKDVDKTLYELLGRSNVRVWVHAGLTRDSANVLKSMRPEPSFH

VVVGNKGFIMDTYRRAVKEKLVRRKYRWNLVFTDYSGADMDWSQVTLPAMVLYINPDE
CCKLMKQEKCTCPPDFQRTQSMLSYLIEYIVTSYGKLEDQQFTTKLDCNAIEMGDMNVT
KEKLLDFFNQDSTNNDSLFYWNIERSGLFLRSRFVLSYSDGSERLELVAKWSADEEYKLLP
GVTLEPLRMFFRIGTSAAIPWTLHKMDPNGQPMVTDEGDPVYEGYCIDLIQKLSEVMEFD
YEIVTPKSGSFGRKLPNGTWDGLIGDLARGETEIAVAALTMTAEREEVIDFVAPYFDQTGIII
VIRKPIRKTSLFKFMTVLRTEVWLSIIAALILTGFMLWLLDKYSPYSARNNPDAYPYPCREF
TLKESFWFALTSFTPQGGGEAPKALSGRTLVAAYWLFVVLMLATFTANLAAFLTVERMQT
PVSSLEQLARQSRINYTVVEGSSIHQYFINMKFAEDTLYRVWKEITLNATSDQAQYRVWD
YPIREQYGHILLAINASGPVPDARTGFRQVEEHLDADFAFIHDSAEIKYEVTKNCNLTEVGE
VFAEQPYAIAVQQGSRLQEDLTRALLDLQKERFFEQLNSKYWNESLRQSCPDADESEGITL
ESLGGVFIATLFGLGLSMITLAWEVFYYKRKQKTNVHDTTTSEKPRPAFVKKGKLRRRKK
TVTIGDSFKPAVDVSHITVYPKGYVP*

>HrhoIR21a

MFWYVFGTFTNCFTFVGKNSWGKTTKNTTRLLIGWYWVFTIIITSCYTGSIIAFVTLPIFPA
TVDSIKQLLSGFYRIGTLDRGGWERWFINSSDPDTNKLFKKIELVSTIQAGIKNTTKAFFFP
YAFLGSQAELEYIVQANYTKTRSKRAVLHISNECFVPFGVALSFPNNSIYTSRFSNDIRRVL
QSGILKKIVSDVKWEMQRSSSGKLLSVGSAILKSTSIEEKGLTLEDTQGMFLLLGAGFLIAA
GALLSEWMGGISRRCCVIKKKATSANSSRNLMTPFNTENEVKDDTDGFDLERKSDLDSNN
SSADSRKTLDGHIIKLTENSITVHENFNSNDWNCRRSSSVDIDQEVKEIFEKDISRRRKAFD
DRSSLQDGRLTTASKGKFGEYIP*

>HrhoIR76b

MVANGRAVFRSFTSDRDFLPTVKAGAVLVKEQTAVDHLMYFDYLTKVREGVVEEERCTY
VVAPNAFMKRTRAFAFPMNTNLTTLFDPILTYLLQSGIVDFLEHRDLPTTKICPLDLQSKDR
RLRNSDLMMTYMIMGVGLASAIAVFIIEMILKRYAVKHKLKPLKKFNSKTFTFKDDSMPPP
YDSLFGKNSKYRGSKRTVVNGREYWETKMKDGTTRLIPLRTPSALLYQ*

>HrhoIR41a

MLTSKMLSIPIEILLKIILQKYFINSYCITVVSEDSIQLKTSIPFIYAIPNDNFVDLLLNSSDIGC
SDYIVNMKNPQEFMKAFEKVTHLGLLRKSDRKILILTHSKSYNAQDKDAILKVLSMNETR
FVANILLVIQADVNEKCYIYDLITHQYVGKDDVRKPIYLNQWNSCTGFTNNVSLFPHYNM
SDLYGKTLKLACFNYEPYSLLDLDTSVDPLGRDGMEVRVMDEFCRWVNCTIELVRDDNQ
WGEIYSYENLTGVGVIGNVVKDEADVGISALYSWYEEYIALDFSTPLVRTAVTCIAPAARV
LASWELPLLPFSLHMWLGLGFTFFYASMALMIAKGFNTDKMFLTTFGMMITQVRFLNSCK
KSGGYHSPH*

>HrhoIR60a

MTMVVFLFISLLINEVSLINPNGPTAVEDFTNCITSIVKVSFKNPGLLVFVDTFFIAEAVGRIK
GNVLKQIHLNKKFSVRVVRPKNEYPVCVNLNEFNTGVVHQNQVDVIPLADYFVIIVDSYS
EFTHAASRLIRLRNWNPHGKFLILLYSFDNIYYLKQIEYIFTCLFRYNVLNVVVLVPHIRNIR
ATIIYTWEPFEPPKYCGYYNETAENRIKVADFCEKGHLKNNTTLFENVVPIDMMSCVVNIL
AIEKQPFIGKDDNVQEANIERFLINEVLSTINMKTNYIITNKSRGERFYNEWNGALKKIVSK
KFNVLLGGIFPDFDVHEDFQCSNTYLEDSYTWVVPRAHPRPPWVALTIIFHKTVWLSVLIG
FTISALSWKFLSTVSGDSTYYTSIDHCLLSTWLCILGLTTHIRPKKESLRIFFVFFNIYCIIFIT
AYQTKLFDVLTNPSFEYQIANVEELIDSGLKFGGFEELHDLFYNSSDPFDNLIGSQWVIVEN
MSNAMVDVVVHRNFSVLCSRLELTYLSATMPQLSDSIGHHKYYAFKTNVFTVPIELIAMR
GYALVEKFSEILEAFKQSGIVSGIRRHYVTFAERKRASIILKLQSQQNDVRALTIQHLQGGFL

ALVLGYVGGIIVFIVELIIKCNLVQKVLYQK*
>HrhoIR75q.2
MKAVNLLALFIITVISCLAEADLVSIIGDLIRVMNKPSSVIATLCWPQYKQLKLYYFLHRENI
SHLTTIQFLKLGHEPKNYWPSQNILFLLDLNCTNVTNHLKLSNDKNLFRSPYRWFLIGIDET
LNNSNSNINIKRQFKPFDIFPDSEVMIILFHYNSRNDSRYEVIDIYKTCKNSEDMKTKLYGN
WDATNRFQKSLNFYKPTALQRLDLGGCEIAISYVLTNNNSIHHLYDQMDDHVDTITKVNF
PTTNHLLEFLNATRKYSFTDTWGYRLNGTWNGMSGYLFRGEVEIGGSPMFVTSERISFVE
YISNPTPTSSKFVFQQPKLSYGNNIFLLSFRETVWYCATALVILIFLTLFAVTFWEWKKINDG
NMLDNRDPGILRPNVTDILILIVGALCQQGSPVQLKGSLGRIVLLVLFLALMFLYTSYSANI
VALLQSSSSQIKTLEDLLHSRMKFGVDDTVYSRYYFSIATEPIRKAIYETKVAPRGEKPRFM
SMEEGIKNMQKGLFAFHMEVGVGYKFVGKYFLEGEKCGLKEIPYLQVQDPWLAVRKNTP
YKEMFKIG*
>HrhoIR68a
MNFKSKLYKPVNADINKWGQKQSNGSFSGLIGEMVRGKADVGLGNLQYTPYHLDLMDL
SIPYTSQCWTFLTPEALSDNSWKTLILPFKLYMWITVLLVLLVTGSIFYGLAKFYLNLLEFK
DHSSIYDKHLDKQIVYDGAKPVGLYLFGEIINSILYTYGMLLVVSLPKLPTGWSIRLLTGWY
WLYCILLVVSYRASMTAILANPAPRVTLDTLKELVESKIACGGWGTQTKIFFEESLDEIGEKI
GEKFQIVNDPDEAAAKVAQGVFAYYENKHFLKYLSVKRKNSLIDTTPQDNSTANSTAVTA
KKKDERNLHIMSDCVINIPISLGFQKNSPLKPLADLYLSRIVEVGLVEKWLNDAMHPIKSL
DSQEEEIKALMNLKKLYGAFIALAIGYFLSTISLIGEFIHWYLIVKKDPNFDKYALDVYYAN
KNKRQ*
>HrhoIR75p
MKLYNLLYILLCIPTDVACSRNVDDMLLSYITMENQPTSLLAPELCWPLHHKTSFTRLLNG
VGVNVAYTMRPSRKEQYLHHITILADFSCSSASDLVLQSDEHGFFMSPYRWIFINLHQQSP
NATILDKLNILIDSNVVVVQKVDDTKYVFHEVYKIAKDYQVIKNLRAVWRAVNDTEKRN
EQTTSSIHVNLTPGNNSVSVNSKTNGEIEDLFYSTPLSCRRRNLRGHSLTMVNVITDSNETK
NHMHDRLFLHHDSISKMSYMVVRICFEMMNASENLLFTNTWGYRDKHGNWQGLIDHLL
KKKADLGTLTIFTKERTEHIDYIAMVGSTAVRFVFREPPLAYVSNIFTLPFSGAVWFAILICV
LGCALFLYITSKWEATMGSHPLQLDGSWADVLILIIGAVLQQGCTLEPRFTAGRCVTLLLFI
SLTILYAAYSANIVVLLRAPSPSVRSLQDLLSSPLKLGASDFEYNRYFFRQLNDPTRKSIYDK
KIAPKGKKPNFYNMTEGVEKIRQGLFAFHMELNPGYRLIQETYQEDEKCDLVEIDYINEID
PWLPGQKRSPYKDLFKVK*
>HrhoIR40a
MRVAVVTNPRESVFRIYYNQGTPNLLHHLTLVNWWSGRLYRSPVLPPAEKVYKDFRGREF
EIPVLHAPPWHFVKYNNDSTVNVTGGRDDKLLSLLAKKLNFRYKYYDPPERSQGSSISGN
GTFKGTLGLIWKRKAPFFIGDMTMTWERLQAVEFSFLTLADSGAFLTHAPAKLSETLAIIRP
FRWEVWPLVLATLLVTGPALWMVIAAPSLWRRQRRDQLQLFNNCCWFTTSLFLRQSSSKE
PSSTHKARLVSVVISLGATYVIGDMYSANLTSLLARPAREQPIGTLQALEEAMRDNGYELV
VERHSSSLTILQNGTGVYGRLAKLMKRQQVQRVRNVEVGVRLVLTRKRVAILGGRETLYY
DTEKFGSHNFHLSEKLYTRYSAIAMQIGCPYLETFNNVVMTLFEAGILTKMTTDEYRDLPK
LSRRSDPVTESDTEGSDAIGESTAASQTQVESTKGLEPVTLRMLRGAFCLLGIGYLLAGVS
FCIEIQIHRRRTRTKAPVPETKIKEKQKKFQRILTNIKIRFRRIAIKIYSKIDTALGP*
>CpomIR7d
SVDMPIDGELNDDLMLFNKNITAAQNLAINAAKIALNNFEWRYVTMVFHNSSILLGLTAF

MQIYRKSVIVGKGTFLHGKESSADRISQFVIFGSDLVDIMCTLDWMRKREFDNTGKFIVIC
NNCDERKAMDIFWNHKILNVVFINDSSGTSSLIGFTYSIYDNQKCVISPPEPLLDSCIHNSC
MGVYPLKLRNLHKCQIIVSTFEQVPFMSLKTGTPIGADGDLLLLIAEALNATLKVMTPHRG
AGWGQLDKDGNWLGSLADVYHDLANFSMTSAAITLTRFKAFHLSTDYHSINMAWVTHP
AVPLPGWQKLLRPFKMKARISLAVTFVLIILVAVFVKSNLWAKLSKRINTSARPQTCVLFYS
WTICMGMPATSLPSKPTFLTMFLLWMFYCFMIRTFYQTSLIHAMKDNLNYPEFENLQDILN
SGYPFGGVPALKDFYIDDPEVYNNWKSINSTEINDMMVSLSRGMKYVLAMNKVTAQSFIL
KHYGDIHIVPQMIVTSPTVLYFKKFSPMVQSLNLILDRLVEGGFTEKLYKNHASTHARKKT
DSTAPMNFEQYMGCYVVLAAGWIVSILVFICEVYCYKFSV
>CpomIR8a
MDFCCLFLAIFIFNLGCVASELSLRFVFIIESHEQDLPQLIGRALKFAEEAQPDLRVSEAIVSL
DRENEDESYRQLCSALSNSVSIIVDLSWSPWDSLEELSSTAGVPLVRARLGSQHLVRAVDE
YLESRNATDAALLMESEADVDRTLYELLGESNIRVWVHAGLTRDSARALKTMRPEPSFFV
VVGSGAFATDTYKRAVKEKLVRRDYRWNLVLTDYSNLELQPVKPAMVLQVDAAECCKV
MGQKDGCSCSQDFERKQPILSALLQLLAETYSKLDDDDFTTRVDCDNLVPENGTRSKVYR
QLAEELGASNESLFYWDGERSGIFLRSRFILSTLKPDIGPQHAAIWSADDEYKLLPGVTLEP
LRQFFRIGTAPAVPWTMPKLDSNTGEPMFNEDGEPMYEGYCIDLIQKLSESMDFDYEIITPK
TGTFGRRLANGTWDGVVGDLMRAETDIAVSALTMTAEREEVIDFVAPYFEQSGILIVIRKP
TRKTSLFKFMTVLRTEVWLSIVAALVLTGFMIWLLDKYSPYSARNNPDAYPYPCREFTLKE
SFWFALTSFTPQGGGEAPKALSGRTLVAAYWLFVVLMLATFTANLAAFLTVERMQTPVSSL
EQLARQSRINYTVVEGSTIHQYFINMKFAEDTLYRVWKEITLNATSDQSQYRVWDYPIREQ
YGHILLAINASMPVPDAKTGFRQVDEHTDADFAFIHDSAEIKYEVTLNCNLTEVGEVFAEQ
PYAIAVQQGSRLQEELSRALLDLQKERLLEQLAAKYWNETARQQCPDADESEGITLESLG
GVFIATLFGLGLAMITLAWEVFYYKRKEKNKVRQEDEETKPKKAFEKDLEKKIAGGVARL
RKRDKKEKKGQVTIGDTFKPVSEKDGVSYISVYPKTEYKP
>CpomIR21a
MRFLRTALFNYILLHYVISQEIEYYPSQASSFARKLVSEFNSEPYQHKHDLFKREAQWRKF
NNNDDTEFTKNKTQKRAVDPVFHGHPKTREELWNERIINESLAFDQTPSLISLIHNITLTYL
NDCIPIILYDSEVKSKESYLFLNLLKDFPIAYVHGYINENNELAEPKLVRATRECIHFIAFLSD
VTKSAKILGKQAESKVVIIARSSQWAVQEFLAGPQSRMFINLIVIGQSFKDGDDDTLEAPYI
LYTHKLYTDGLGASQPVVLTSWSHGKFSRQVNLFPRKMTEGYAGHRFVVAAANQPPYIFR
TIKTDADGGNPRVVWDGIEVRLLTLLSQMNNFSIEIKEPREPHLGSGESVLKEITGGRADIG
VAGIYLTSDRIRDTDMSFSHSTDCAVFVTLMSTALPRYRAILGPFHWTVWLALTLTYLFGIF
PLAFSDKHTLKHLLHNSGEIENMFWYVFGTFTNCFTFVGKNSWSKTTKITTRLLIGWYWL
FTIIITSCYTGSIIAFVTLPVFPETIDSIQQLLDGFYRVGTLDRGGWEKWFLNSSDPKTNKLL
KKLQLVGDVPSGIRNTTKTFFLLPFAFLGSRAELEYIIQSNFTKTKKSKKAQLHISNECFVPF
GVSLTFPNNSLYSSKLSGDIARILQSGLMDKIENEVKWEMQRTPSGKFLSAGSGTLKLGAI
TEKGLTLADTQGMFLLLAAGFVLAAAALISEWMGGCSRKCRPQKKEDEPSSAHSREHLIP
TPKSDVDSEIKVISDSAESRFRLNPRPDSEDSRDSLEGTIINVTKESIIIHNNYHTSNWDSRRS
SSVDIDKEVQEIFEKDEKRRRINSGTVPLKDNQREATASKGAFGDHLSDH
>CpomIR25a
MASLIILLLFLFVPDSFSQTTQNINVLLINEENNALAEKSFEVAKEYVRRNPTLGLAVDPVIV
VGNRTDAKAFLENVCRKYNDMLSAKKTPHVVLDFTMTGVGSETIKSFTAALGLPTISGSF
GQAGDLRQWRNLDANQTKFLLQVMPPADILPESIRAIVTKQDITNAAIIFDEFFVMDHKYK

SLLQNIPTRHVITPVKSFNRDEIKTQLRSLRELDIVNFFVVGSLRTIKNVLDAADENQYFGR
KTAWFALTLDKGDISCGCKDATIVYMKPTPDAKSRDRLGKIKTTYSMNGEPEITSAFYFDL
SLRTFLTVKSLLDSGKWPNDMRYISCDDYDGKNTPNRTLDLKTAFHEIKETPTYAPFFIPED
DPMNGRSYMEFNTDLSAVTVKDGASIGSRNLGSWKAGLSNPLSLTDPQNMSDYSAQLVY
RVVTVEQKPFIIRDDEAPKGFKGYCIDLIEEIRQIVKFDYEITLVPDGNFGTMDENGNWNGI
IKELVEKRADIGLTSLSVMAERENVVDFTVPYYDLVGITILMKLPRTPTSLFKFLTVLEDDV
WLSILAAYFFTSFLMWVFDKWSPYSYQNNREKYKDDEEKREFNLKECLWFCMTSLTPQG
GGEAPKNLSGRLLAATWWLFGFIIIASYTANLAAFLTVSRLDTPIESLDDLSKQYKIQYAPL
NGSAAMTYFERMAHIEVRFYEIWKEMSLNDSLSDVERAKLAVWDYPVSDKYSKMWQA
MKEAGLPNSIEEAIQRVRDSESSSEGFAWLGDATDVRYYVLTSCDLQMVGDEFSRKPYAIA
VQQGSPLKDQFNNAILQLLNKRKLEKLKENWWNNNPEAMKCEKQEDQSDGISIQNIGGV
FIVIFMGIGLACITLGVEYWWYKWRKRPIIGDVTQVEPSKTTRNNADNSTTKIGEGFTFRS
RNMGLSNFRSKF
>CpomIR41a.1
MIMPSKLFPVEILLNILINEHLQEYFCLTFVTETKLTVNIPINMSLMIIQPNNSVLAEQILDAS
EKGCSDYIIQMHEPENFMIAFEKVNHLGDIRRSVKKLIFLPVQDDMNNRSVLTNILALRET
GFVANILLVVPSLQSSGDCKVYDMITHTFVGSDEDVQKPLYLDRWDSCTGHFERGVNLFP
HNMSNLYGKTVKVAAFTYKPYVLLDLDPSLNSLGRDGMEMRIIDEFCRWVNCTVEIVRD
DEHEWGEIYENNTGVGVLGNVVEDRADIGITALYSWYDEFRVLDFSAPIIRTAITCVAPAPR
ILTSWDLPLVPFTWTMWMCLVFTFFYASFALSIAQRSTDNVFLDTFGMMITQTREDATSWR
IRSITGWMLVTGLVIDNAYSGGLASSFTVPKYEASIDTVEDLVDRKMEWGATHDAWIFSIM
LSEEPLIKSLLSQFKTYPADILRQKSFSRSMAFSIEHLPAGYFAIGEYITKEAAMDLEIMLDKI
YYEQCVVMLRKSSPYTAKLSELVGRLHQSGLMLSWETQVALKYLDFKVQLEVRLSRARK
DLEEIEPLSIKQLLGIYIFYFGGVVIALLVFFGELLSKCSKPSIVL
>CpomIR41a.2
MVKMLIPSTIYFPIEILLNTIINNYLQTSFCLTFVTETELMINLPLNMSSMRIIPNNSELVQQIL
ETSEKACTDYIIQMDEPRNFMIAFDKVNHVGDVRKSDKKLIFLPLEDEFYNPSVLTDLLSL
KETGYVPNILLITPTGQKSSDCKVYDMITHTFVGAEEQIQNPLYLDRWDCCTEVFEKEVNL
FPHDMSNLYGKKVKVGAFTYKPYVLLDLEPSLAPLGRDGIDIRFIEEFCRWINCTVEIVRPD
DGQEWGEIYENNTGIGLVGNLVEDRTEIGITSLYSWYEEYRALDFSAPIIRTAVTCIAPAPRIL
SSWDLPLVPFSWLMWMCLIATFFFASFALFVAQRSTDDIFFVTFGNMIGQSPGDSSSWRIRS
ISGWMLVTGLVIDNAYSGGLASSFTVPKYEASVDTIQDLVDRKMEWGAPVDAWLYSMILS
EEPLIKSAISQFKVYPPETLTKKSFTRSMAFSIERLPAGSFAIGEYITKEGAKNLELMVEDMY
YEQCVVMTRKSSPYTAKLTELVGRLQQSGLLLCWETQIALKYLDFKVQLEVRLSRTKKDI
DGVEPLNVKQLLGIYLLYFGGLSISIVVFIAELLIKRGKAVIVI
>CpomIR60a
GFYVQATMLKIICLLSIGVNAKVNPHGPTVVSDFSSCVSEIIDKNFAQSGLLFFVDTFNVST
PVAGIRNGIIKSVHTKLKYSVKIAIPTKKDKGICVNNDKTAIEISVKSRMDHFEATSLADYFI
LIIEDYKDFSYIASRLIRAISWNPRALFILVYFSISNSDDQNIRHAEDMLFCLFKVNVINAVVI
IPEVNNVRRANIYSWRPYAPPKYCGHYNESIRNRLIVENVCERGKIKYAKKIFESKIPSDM
MGCSLKVLALERQPFISHNPLDPNIESLLINQVAKRYNLSLRYEILNSFRGEKLFDGDWTG
ALKELTYKKGHLLLGGIFPDDEVHEDFECSSNYLADSYTWVVPRALPKPAWLALFVIFQK
TVWLTVITCFVFIALSWMVLAKLSKDPTYRTNLDHYFINTWLSNLGFCAFSRPITNSLRLFF
VFINIYCILLLTAYQTKLIDVLTNPSFEYQISTVEELVESGLKFGGSEELHDIFENSTDSIDNYF

LDGWIDIADIRDALRDVAIHRNFSLMCSRLELAYVSAIIPELSDQFGKYMYYAFPTNVFTVP
LEAVSMKGFPFMKGFSRTLTHFEQHGVNNGVIVYFGGYLLRQRALLLNKFKIEHNSRDPL
SIQTLQGGYLALMFGSVCGTFVFIVEIILNTKFVKKLKIL
>CpomIR64a
MNLTTYSALFTIFSTAEINLITDVFKHKHLHFGTIFHCSKPENAIFLQKHLKKMDLRFSTIM
MHSNASHFKQTNDSRVGIVLKTSCENWTQVFEHFNCNLFEKSLYSWLIFTDDLSSASEALS
RYPIEVDSDVAIIYRQEKSYYIYEVYNTGYFTNGRYHVEPVGYWYYKLRIKGHRRTNLDGI
VLRSAVVVTHSIGHQTFEEYISRLKPEVDSLHKLKYFTLLNYLRDMYNFSLIVQRTNSWGY
VTNGSFDGMVGTLQRGETDIGGTPVFIRADRAKFIYYVTATWPSKPCFIFRHPKHPGGFLTI
YTRPLSYNVWLCIIALLVFAGSLLCVLIKLRVTRTAGDDGDLSASLALLSIWSAVCQQGTT
VNLSANSVRLVLFFSFLFSLFVYQYYNALVVSTLLRAPPVTIRSLEDLLRSKLKAGVEDVL
YNKDYFRRTTDPIALELYSRKIASSPRPNFLPPDRGMALVKQGGYAYHADTAYSYPIIRRTF
TEREICELQEVELFPPQTMFAVMKKGSPYIKHLSYGIRKMAESGLMQRLKTIWDEPKPLCV
RTPDSSIFSVTLREFITPLLLLCLGMLAATVVFMAEHVFYRLQWKRIQFRH
>CpomIR75d
LQVQTRIFAGMELVSFALAYFAAKRLSLLTAFLCWRPEELSALCRDAQRQGMRISIADWTH
LPPLEPYATHREGMLLDVTCPDAPLVLEKASSTRAFNLRHTWLLLHNAPFNASLMEVTLD
STLVLPDADVAWVANDQFLDVYRIKHDQALITMPLGDDAARVALPAAPTRRRDLNNVYL
RSSTIISQPQFFKGWNDLTVRQIDTFPKLTWPLMHLLADDLHFRYNLIQVDLYGENRNGSF
DGLAGQLQRQEIEVGITSMFLRADRMQVLHFCSETVELRGAFMFRQPSKSAVSNVFLLPFS
RGVWIATALTLVLAAVTLALLARRPRLXXAVDASLEQLSIGEAVIFTVGTACQQGFHIVPEL
ASARVVMFCALMTALFAFTAYSAKIVAILQTPSDAIRTIDDLTNSPMTMGVQETTYKRVYF
AESTQPATQRLYRHKLLPLGDRAYLSVVDGVAAMRTGLFAFQVEEPSGYDIISKTFTEREK
CGLMQIQAFKLPMVAVPIRKHSGYKELFATRLRWQRETGLMDRTRRIWLASKPRCDANSG
GFVSVGIIDILPALHVLAAGMVASVLFLVLERSMARLKCCGVRGS*
>CpomIR75p.1
MDIWKLGAIIWLFKSHVEGRAGIGKFLTSFVDNERKPTTVVFHGICWNNSVKLHVMKELS
KAGIRSSQSMSKRTSLIDHTVLLLADLNCTGTDDLIINATQRELHRLPYRWLVLSDAPRFGR
SSLWDLFLVDSELVLATVDGAGYSMTEVYKPSPTSPAILTPRGTFHHVLTDTRPHRELFRRR
RDLMGVPLTITNTIQESNSSIYHLLQEDSLELEHDLISKNSYTLAKVAFLTLNSTPVATFTNS
FGYLQNGQWTGVIKELLEYNADIGTNVGMSQSRLKQVMFLDPLDNGRARFIFRQPALSLT
ANIFSLPFSPDVWIATGLSSFVAGVAYYLSTRLIKTRAEKGTVRDAYLLTMSALSQQGCEVQ
PRHVSARIVLWVVFTSMMALYAAYGANIVVLLQAPSTSVNSLATLAKSKLALGAADVNY
NHFLFRASSDPVRNDIAKRINSDKGPKAFYGLTEGVEKIRKGLFAFHSVVEPVYRQIDRTF
QEKEKCDLMELDYIGYAAFHVPGSKKSPYLELLRVTFKRLREVGIKSAVNFRYEARRPSCK
ESIAMFSSVGITEMRPVLIFMAYGVALSVAVTAAELLVFHANRYRLRQQRLAVQLGRI
>CpomIR75p.2
MKILFSFVIVLFLSLGKAFDDNDINMIVSFVTLDERATAVLTPYVCWSTYELTSLAKSLHDT
GISMAASLQPKRPELFLQNLVIVADLRCRRTDDFLIKASDEGFFKSPYRWLLISQDQTELNV
LDQLAMLVDSDVVIAQRRGADYQYVEVYKIVENSQLIYNTRALWRPIDKNNNTAIITYYN
KSKVVANKYGAVEDYRKSKILSTRRMDIRKHTLTMVNVITDSNDTRKHMDDRLNLHQDS
ITKMSYMVVKICFEMMNSTEKLIFTNTWGYVDKNGSWNGIIERLIKKEGDIGTLTIFTQER
LKIIDYIAMVGTTAVRFVFREPPLAYVSNIFALPFTGAVWLAVFICVLACALFLYITSKWEAT
MGIHPMQLDGSWADVLILIIGAVLQQGCTLEPRRAAGRIVTLLLFIALTILYAAYSANIVVLL

RAPSSSIRSLQDILNSPIKLGASDFSYNRYFFKKLNEPLRKEIYNKKIAPKGKKANFYTMKE
GIEKIRKGLFAFHMELNPGYRLIQETYQEDEKCDLVEIDYINEIDPWVPGQKRSPYKDLFKI
NFIKIRESGIQNCIHQRLHVGKPRCLGAVNTFSSVGIMDMYSAMLATLYGMFMAPAVLLLE
IAYKRLMVAREKRMQHNNSHSHT
>CpomIR75p.3
VRRRDLRGADVVLPTVLLHNQSLEDLPDYLHRERDTLTKVVYYMCTHLVEWVNGTKIW
NRTTSWGYLQPDGQWDGIVREMQDGRADIAGSCMMAQKERVKYVNYVLAPSKIEAMF
VFKKPALASVTNIYVLPFDIGVWVSIIVLIIISSSTLFLSSFGEDRMKNYYSKSWLQKMSDGF
FDTLCLMFQQGTAADPLSIASRQILLLGLMAFMFLYTAYSANVVALLQSPTNDINSIETLLT
SPVACGSQDVVYAWQMFGHESRPIHRLLADRKINSQGKKGFLSVEDGIRKVREGMFAFHV
EQTAGFDQIQKTFLEDEKCNLGFIKYMSTTSPFVAFSQITPIKEMLRIGANRIMEVGVQSRS
ARRLVPERPRCGASAAMFNAVRLSDVAPAFRVLLAFYALTLPILGLEILVKRREMKRGIFES
KSVDTATAEITNNIII
>CpomIR75q.1
MYTSLTIVCAIFICSSFALSISKENDIKVIVDVIQSFNKPTDVISNVCWTNIYKKKLTANLAA
ADNPRSIKFVNDIHAKDLVHPEKVTFLIDVRCKDSGGFLNKAASRKYFGRPHRWFIINTPA
NEVSVPLVIDKMHLLPDSEVYVMQLINNSYSINLIYKIKPNREWIIENYGNWSTENGLTISR
RAKKVALVMRRRNLARASIVTSMVITDNGSFADLETLRYKQIDSVTKGGFHQLTALYEFM
NASREFVFTDQWGHHVNGTWHGMVGHLADGTAELAGAILFITKERMPLIEYMSHPMESS
IKFLFREPPLSYQNNLYLLPFQASVWYCVGSFVLVLIFAMYFSAYWEAKKVADEKQKADD
TTVLVPTISDVTIFVMCAISQQGSTVELKGMLGRFMILILFLVFLFLYTAYSASIVVLLQSSS
NQIRTLTDLLNSKLELGVEDTPYNRYWFMNEKEPIRRAIYEKKIAPSGSKPKFFDLTEGILQ
LQKKPFALNCNLGVAYKVMERYFYEHEKCGLQEISYLQNNNPWQAVRKGSPYREIFKIGL
LRNAEFGLNDRTNRIMFSKKPVCSVRGGSFVSVSLVDCYPILLLLLYGMILGVMLLLVEILY
YRKMNTRP
>CpomIR75q.2
MVADVIRAMQRPSAVIAMLCWSSNLKLQLYSALEGENVTQITMMQFLKAGTVPERHAQD
QHIVFLADLDCPDIISYFQTSSLNKHFRSPFRWILIDSGNNDTSQSYIPNAVGNFDILVDSEVI
LAHHLGDGSYRLHLIYRIGNNTDWKKEFYGTWDERRRLQKQVMEGEIILRRIDLESYELPI
CYVLTDNDSINHLYDNVNDHIDTITKVNFPTTNHLLDFLNASRKYVFANTWGYRVNGTW
NGMTGYLVREEVEIGGSPMFFTSERISIVDYISSPTPTRSKFVFRQPKLSYENNLFLLSFRAS
VWYSSIALLLLLVIVLFIVTIWEWKKTRGHEDKKLEADSGILRASVVDVVLLIFGAACQQG
STVELKGSLGRIVMLILFLALMFLYTSYSANIVALLQSSSSQIKTLEDLLHSRIKFGVHDTVF
NKYYFSTATEPVRKAIYETKVAPSGSKPRFMPMDEGVKKMQKGLFAFHMETGVGYKFVG
KYFQESEKCGLKEIQYLQVIDPWLAVRKNTPYKEMFKLGTKRIQEHGLQSRENRLLYEKR
PKCSGQGGSFVSVSMVDCYPALLVLFYGAVFSVGLLFIEILTKRRNDILRKISRAKTLGVDV
EDY
>CpomIR76b
MTGLELIVSSICNATFCEVVYDNPITDTLLPAQKKELLKIAEDLNGKHLKIGTYDNYPLSW
VHTEDNGKLTGRGVAFVVLDILRERFNFTFDVVTPLKNFEIGIEGRMEDSLIGLVNSSQVD
MAAAFLPIVYKYQQFVDFSSILDKGVWMMMLQRPKESAAGSGLLAPFEIQVWYLILAAV
LSYGPCITLLTYLRSKLVRDGEKNISLTPSFWFVYGALLKQGTTLAPEANTTRILFTTWWLF
IILLSAFYTANLTAFLTLSKFTLDVEYPEDLYKKNYRWVAPEGSTVQYVVNDADENLHFLS
KMVANGRAEFRSVNADRQYLPYVMGGAVLVKEQTAIHHLMFEDYLKKTKAKVPETKRC

TYVVAPNPFMEKLRSFAFPKNSKLKLLFDPVLTYLLQSGIVTFLEFRDLPSTKICPLDLQSK
DRKLRNSDLSMTYMLMGVGLATAIAVFGGEMIIRYYVRIKIRKNRGERTRTKTVKTSKHR
RFRIQDDSHPPPYDSLFGQNSRYKMNGDSTTKIINGREYWVVGTVSGDIRFIPVRTPSAFLY
QRDK
>CpomIR87a
MLLLRTIHSGIKYSVMVKDSFYKHANASHFPEKAKNYMLILEEKSELVRNILQLNKLPTW
NPLAKAIIYYQLLPDEDGETIAKKFINELREYKLLKSIVFIYSPDDAGLISYTWAPYSDTNCG
GECDSVYILDTCKNSIVKQKNAQREMFPLNMKKCPLVTQAIISEPYVMPPVRQLTNTSYPD
AYEFQKGGEINLVKLISEFTNMSLIVRISDVPENWGLIYPNGTATGAYGILRNDSVDLVIGDI
EVTRTIRKWFHPTVSYTQDEMTWCVPKSAQASTWNNLVIIFQWTTWVATLLSIVTMGLIF
HYIYYRENDRKVTKLPTNSLLNTFSMILGWGASFKPKTATFRILIFAWLFFGMIMSISYESFL
RTFLMHPRYEKQISSETDLIQSGIPLGGRAIYRSYFETNNASSFYLYRKYISTSFSEGIKRAAL
ERNFAVVASRRQAEYQDQKLGKGEQLLYCFKEGNNLYKYGVVLLARRWFPILERFNNIIRS
VSENGLIEKWNQELFIHTVGVDGTSKVVPLGIRHLLGAFIFIGIMYAASVIVFVVELLLNVS
KKRKGNKPICRACLSSKYSSRR
>CpomIR93a
MRIWVVLICVVGVRGEEFPSLITANASIAVVLDRQYLGEQYQPLLDTLKDYIKELARVELK
HGGVVVHYYSWSTISLKKGFIAVFSIASCEDTWSLFSRAEEEELLLFALTEVDCPRLPPDSA
ITITYTDPGQELPQLLLDLRTTRAFNWKSAVILHDDTLNRDMVSRVVQSLTSQIDDEDVPTI
SVTVFKMRHEINEYLRRKEMHRVLSKLPVKHIGENFIAIVTSDVMSTMAETARDLFMSNT
QAQWLYVISDTSIRNSNLSSFVNALYEGENIAYIYNITDDREDCKNGLMCYSEEMMNAFIS
ALDSAVQEEFDVAAQVSDEEWEAIRPTKIQRRDTLLKHMQQHIAVNSVCGNCSTWQAMA
ANTWGSTYGGNVQADNVAAPDNETNEAIQKIELLQVAYWRPSDGLRFTDFLFPHIVHGFR
GKVLPIITYNNPPWTILKANESGSISSYSGLIFDIVDQLAKNKNFTLKLIFPGDMKDVLSNK
TVTNDMYSQSAKLTMMAVARKQAAFAAAAFTVLSDRNPGINYTIPVSTQSYAFIIARPREL
SRAMLFLLPFTTDTWLCLGFAVVLMGPTLYVVHRLSPYYEAMGVTRQGGLATIHNCLWYI
YGALLQQGGMYLPRADSGRLVVGTWWLVVLVVVTTYSGNLVAFLTFPKQEVPVTTVSEL
LENRAVYTWSISRGSYLEFELKNSDEPKYVSLLKGAELTSDSSGLEGNLASGSPLLSRVRQ
DRHVIIDWKLRLSYLMRAEHLATDKCDFALSAEEFLDEQVAMIVPAGSPYLPVFNKEINR
MQKAGLITKWLSAYLPKRDRCWKTSSVTQEVDNHTVNLSDMQGSFFVLFLGFFSASSVL
LMEWFYHRRKSQKEDVAIKPYVE
>CpomIR1
MTMWRLLFLVASAASLPTDWPHMAVDYFQHKHVKYVAHLSCKDAAEIKGVLRLLMNEG
IRAAVGLIDQGPMNIMPLLYQYEASVGVLVDGDCINTRDILNNASESMMFDDTHFWLVM
NDNCSMGFVEDTFLDLKLSVDADVVVASYCGDIYQLTDVFNFGRVQGNVLETRELGAWT
SERGLEIVLQGFKYYNRWDFHNLTLRAVSVIRNSSKEFHEGMLYEPGFTVGVAAMTKISSQ
LLNLLKEMHNFRFNYTIVGRWIGTPERNSTKAMSNMLLWRDQDISSTCTRLFSNWLDWM
DPFFPSVTELETKFYYTISEKGIGDYENQFLTPMSPEVWWCAAATGVVCALXXXXXXXXX
XXXXXXXXGVFSVLAAGFQQDYEDGHQTKKDSSSRKLALLVVGLTSMLMYNYYTSSVV
SWLLNAAAPSLDSLDGLIKSDFELVFEDIGYTRQWLDNPGFFYYMGYKNEKEDELRAKK
VTNVKRTLPLFESIEDGIELMRTGKCAFHTEPYTASQVISRTFADKDLCSLAGLQIMPPSYV
YVMGQKNSPYRQFFVWSMMRLLERGHTRATRARVGGQIPPCSGLTPRSFKIFSI
>CpomIR2
MEEETFTVASTPDCMTVLQDERNDPLWRETQVGCKVLLLLQEIHKFNITYSKGPYHYNER

GFYEYVDEVDIYAKPRSLWSPVMVNYTPIAPIMDWKFGYILRHPFNIQHFYSLAFSKPAWH
FIVAMILLVSVLFYILNRAEQKLTGENLKCYFWSELLIAFGIICQHYISINPMELTSRRIAFISF
FMFSYILYSYYTSTLLSDLVYDRDNEMDLETLAESDYEHAVLDSVTSVFKVLVEQLRNNN
KMFPQRQSFTENKLINHRVVNISTGLAEVKMSKTALLSDYVSIHSGVIQWFSESEVCDLIK
VDIFSNVLKYLVTSKKFKYIDEFKISTLRAYEAGVLQRLLSPHPIQHFSPTCVSSHFQADIGL
VRKPFILLALGYVLCGFILLVERVYYNRYKVWPYVN

>CpomIR3

LSAKMVGKSILFFLLSSVAGLEDRDIDFSVDFLKARDVKYICMLTCGDRTWNKKFAKNAS
KSSIAVSYVRIDDSLSDLDSVRVCLSPEFTDVGVLIDTKCPLYEEVLMYASENLLFDANHK
WLIIDIDTWISNISTVFNVETNENFSWLMNTLEKLNMSIDANVMLSLQKGSENNIYEVYNF
GKLRGGNVVVKKLGNWRNRADLIQHLNAYKYYRRWDFENFTINYVAVMSTPPKVFDVN
MLVGDTPAPGVAVMTTTVTRVLLEIAELHNIRYNYTIVDRWIGKFERNTTPVVATLLYFKE
QDITPVLRVTSEVFQRVDMVSPPITSIETRYYYRIPTTGPGKFENQFLRPLTKGAWGCVIAVI
SLCALVLFLTVRAETRPAALQYAVFSVAATFCQQFFEDGGYDDPRRESSARQLTILVTGASC
VLIYNYYTSSVVSWLLNGPPPSINSLQELLESPLSLIYQDIGYTRSWLQNPKYYYNKKNSE
VEDQLRKLKVFKKKKGEPLLVPLEEGIEMVKAGGYAYHTEVYNANMLISRSFNQEELCEL
GSLQSMEETPVYIAIPKDSPYKEFFNWKLVAKNELKFIGKMKHRILQGHE

>CpomIR4

MIIPAVAAFFKYKIVSSIIIFTCGNEFEQIRLVRQLSLQGMRATVSCDPGILNEEHKTLQGVLY
FNRPNDTLLDETSWEHFSMWYKWLIIGNEVPSRLNHTTRYDADITLLGLRQLGAIDAIDNS
SVAYHESILFEDLYVHLRDGVSRHPWAVWTPAGFQPLYELERIRRRHDLKRYTMRIPTPVG
HYDDSYEGTFADYVMDNSQPGRDSAIRCGYGTSSLILEWLQAKEVILQMEQWSTDAGNK
SMFTRLAQGTSELSGGILRMQHKRLLKLDYVIPLWIFKVGFTYVAERESSSNMFVIPFTGTT
WAACAVVTLVLAIAQRATAKQESEKEGAFVAVMATWLQQDASAVPEGASGRITFMALSIC
SMLVYAYYSSAIVSALMSAGSSGPTTLRALGDSRYRLASEDYEWIRAQMFDVYIPNWPEM
EYLKRKKLQSMANFYLDWQAGMQLVKSGTTAYHAEYNHVYPLMSVLSDDQVCKLQYV
DTVPPIMSWLVTTRRGQWTNLLRIGGDWLHETGLVKRMLSRWQLKPPPCRAALLAERVS
YGDVAPLIILTVVGLLTSVAVLFLERAVAKWRAKKTDKSS

>EposIgluR

MQGLTPSPMSILDSLCKEFLAVNVSAILYLMNHEQYGRSTASAQYFLQLAGYLGIPVISWN
ADNSGLEKRASHAALRLQLAPSIEHQTAAMLSILERYKWHQFSVVTSAIAGHDDFIQAVRE
RVTALQDRFKFTILNAVVVKKPADLNELVTSEARVMLLYATREEAADILSTAGDLHLTSENF
VWIVTQSVLGSMQQPNKFPVGMLGVHFDTSSSSLIAEIATAVKVFAYGVDSYVSEPENARH
PLGTRLSCSGAGAGEARWSTGERFYRHLRNVSVDSEAGRPSIEFTPDGELRAAELKIMNL
RPAIGEQLVWEEIGTWNSYPKERLDIKDIVWPGGLHTPPQGVPEKFHMRITFLEEPPYINLA
PPDPISGRCSLDRGVICRVAPEVDVAGLEAGTAHRNSSLYQCCSGFCIDLLQQLAEQLGFTY
ELSRVEDGRWGTLHNSKWNGLIADLVNKRTDMVLTSLIINSDREAVVDFSVPFMETGLAIV
VGKRTGIISPTAFLEPFDTASWMLVGAVAIQAATFSIFFFEWLSPSGFDCSTGQNSKRVPQNR
FSLCRTYWIVWAVLFQASVHVDSPRGFTARFMTNMWAMFAVVFLAIYTANLAAFMITREE
FHELSGLDDPRISRPLAIRPPLKFGTVPWSHTDATLAKYFREPHAYMGQYNRSTVSAGVSG
VLTAELDAFIYDGTVLDYLVSQDEDCRLLTVGSWYAMSGYGLAFTRNSKYLSMFNKRLL
DLRSNGDLERLRRYWMTGTCKPNKQEHKSSDPLALEQFLSAFLLLMAGILLAALLLLLEH
VYFRYMRAHLAASTVGPCCALVSLSMGQSLSFHGAVVEAAARGFGAGGRGHCRSAVCA
AQVWRARHERDAAMARARQLAATLAAHGLQPPPRRLASAAALLGEAHDATRPRTLHAP

ADLLPDLDRPLSCGDLRAKERTRVEMETVL
>EposIR1
MWLILLLLATAVDSRLPPGEMVTDYFLHKDAKYVAYLTCGASAEYKAVTSVLMSEGMRA
AVGRIDQETIDLDRLLYQWDAAVGVVLDGACRNTQNVLINASESMLFDAAHAWLVLADE
ADASARFEDLKLSVDADVVVATYFDEDKYQFTDVFNFGRIQGNALETKQLGTWTVDTGL
DLPLPRFKYYDRWDFHNLTLRAVSVMRTIPKVLDEKMLSEPVYTDGITKFTKISSMLLNQL
KDMHNFRFNYTIVDRWTGTPQRNSTPSLSNTLLWREQDISAASARLFPIWMNWVDPIFPP
VTQLESKFYYIIPDSGVGDYENRFLTPMSADVWWCSAAAGVVCALVLVAAAATEGRPEPG
SYGIFSVLAAGFQQDYEDGAQSQEDDSTQSRKLALLVVGLTSMLLYNYYTSSVVSWLLSA
KPPSLDSLDALIKSDFELIFEDIGYARGWLDNPGFYYFSGYVNKKENELRMKKVTSAKRS
VPLMQTVEAGIELVRTGSYAYHTEPYTASQIVSRTFVDEELCALGGLQMIKPAHVYIMAQK
RSPYKQFFVWSMMRLLERGHIKATRARIGGEVPPCSGQTPRALSLGQAAPAFLLLVEFMV
LALLMLVVEVWWYRYKKRTNKRGQAPLKVAQKSR
>EposIR3
MLRNILLIFLVTKVSGLQQNVKEFALDFFKTRDVQFICLLACGEKTWEQKFVNNASKLSIA
VSSVTIDDSSDYSDALRVCLTQKYTAVGVLIDTKCPVFEDVLLFASENSFFNGNHKWLIVD
NDVWMSIVTSDSDGNETNGNLFWLNSMFQKLNLSVDADVSLSLQKGSENNIYEVYSYGT
IRGGSTIVSKMGSWKNKSALLPQLNGYKYYRRWDFNQSSMNLVAVMSTPPEVFDLDMLI
GDQPKVGVAIITTTSLKVLEELKQLHNIRYNYTIVDRWIGDFDRNSSRVAANSLYFKEQDIT
PVLRVTREIFQKVDMLLPPLTAIETRYYYRIPTTGPGKFENQFLRPLTPGAWGCVCAVILLCS
FMLFLAAKAERRPSAVQYAVFSVMATFCQQFYEDNIGNEDPTRQSSARQLAVLVTGASCV
LIFNYYTSSVVSWLLNGPPPSINSLQELLESPLSLIFQDIGYTRSWLQNPTYYFNKKNAEVE
DKLRRYKVFNKKAGAPLLVPLNEGIEMVKAGGFAYHTEVYNANTRIAKTFTQSELCELGS
LQSMEKSQLYASVPKNSPYKEFFNWNLFRLHEAGIVSRIQRRTSSPEISCGGSSPRALALGG
AAPAFMLLAFGFFLSTIILLLERFIHRNNSRWENKFNALAP
>EposIR4
MTEIAGGSSELSGSILRMRLDRIDRLDYVVPVWPFLVGFTYLSERESSSNLFVMPFSRGVW
GACAAIALVLTLAQRATAKAPVERDGAYVAVVATWLQQDASAVPEGASGRLTSMMMSLC
SMLVYAYYSSAIVSALMAAGGGGPTTLRALADSRYALASEDYDWIRYMMFDEETGWDD
LEYLKRKKKTSSFYQSHERGMQLILEGNTAYHAEYNHIYPLMNIFSDDQICKLQYVYTVPP
VMTWLTTKKQSQWKEVMHSVGGWLHETGLAKRLVARWQLKQPPCRAALLAERVKYGD
VAPLVYLSTAGVIAAVVLLFLEIAVARWNARRSVESNGNDDTIIMEDDVA
>EposIR7d
MKVFSALTHSRLRCLVLPISSLHMDHFEPIPLADYFVAIIDSYDDFTRLASRLTRARSWNPK
ALFIFVFFGITSTDDENIKHAETMIHCLFKLNAINAVVIIPQANNIRRANVYGWKPYDPPEY
CGYSNESARRRLFVENVCDRGVVKYAKTVFEEKIPSDMKGCTFKMLALERQPFISTDPDD
PNIEQLLINDVAKRYNIKLEYNILNVFRGEKQINGHWDGALNDLILKKGELLLGGIFPDNE
VHEDFECSSTYLADSYTWVVPRAFQQPLWLAFFIIFKKIVWFSVVAVFIVAALTWKLLAKL
SGDPTYRKNLDHYFINTWISNLGFAAFSRPVTHSLRLFFIFLNLYCVLLLTAYQTKLIDVLTN
PNFEHQMSTVEELAASDLECGGSEELRDLFENSTDPMDKYFESKWQNILDIREAMIDVAL
HRNFSLLCSRLELAHIAAVVPELSDKYGNTKFYVFEHNVFTVPLEMVSLRGSPFLKKFSKT
LESFRQYGVNDKVRKYFAGYTLRKKAVLQTDLETENSSRDALSVITLQGGFMALLFGYVF
GSIFLVVELIMNTKLVKNIKIFKKSVYTLI
>EposIR8a

MDFCCIFWFIFVINLACVVSELSLRFVFIVEVHEQDLPPLVGRALKIAEDSQPETRLSDSIVL
LDRENEDESYRQLCSAVSEGVSTIIDLSWSPWDSADQLASDAGVPLVRTLLGSQQLLRALD
EHLESRNATDAALLLESEGDVDRTLYELLGESNIRVWVHAGLTRDSARALKAMRPEPSFY
AIIGGSAFVADTYKRAVKEKLVRRDYRWNLVQTDYSPPAVTAAAPAMALHVDPGECCRIL
AMKDGCSCSQDFERKLPILSALVQLLAETYSKLEDESALTARVDCEGVAPELNDTRAKLYR
QLAEDSGASNESVFYWDGNRYGLFLRSRFILSTLKPDAGLQTVATWTADEEYKLLPGVTL
EPLRQFFRIGTAPAVPWTMPKLDPATGEPMFNEDGQPLYEGYCIDLIQKLSESMDFDYEIVT
PKVGTFGRRLPNGTWDGVVGDLMRAETDMAVSALTMTAEREEVIDFVAPYFEQSGILIVI
RKPTRKTSLFKFMTVLRTEVWLSIVAALVLTGFMIWLLDKYSPYSAANSPHAYPYPCREFT
LKESFWFALTSFTPQGGGEAPKALSGRTLVAAYWLFVVLMLATFTANLAAFLTVERMQTP
VSSLEQLARQSRINYTVVEGSTIHQYFINMKFAEDTLYRVWKEITLNATSDQSQYRVWDYP
IREQYGHILLAINASIPVPDATTGFSQVDEHTDADFAFIHDSAEIKYEVTLNCNLTEVGDVF
AEQPYAIAVQQGSRLQEELSRALLDLQKERLLEQLAAKYWNETARQQCPDADESEGITLE
SLGGVFIATLFGLGLAMITLAWEVFYYKRKEKNKVQVLEDTEKKPKKAFEKEPHKKISDS
VARLRKGRKKPERVTIGDTFKPVSEKEGVSYISVYPKTEFKP
>EposIR21a
MLVTIKIVLLKIIILYAAGQDVEYYPSQSLLNTFDGKILPQQYKFKSKFGVKSKPYLDSHFE
YKREAQWRKFYKEDNVEKNNIRKRAVDPAFHGHPKTREELWNERFINKSTAFDQTLSLIG
LLRNITLTYLSDCIPVILYDSQVKSKESYLFQNLLKDFPIAFVHGYINEDDEVVEPKIIRATK
ECINFIAFLSDVTKSAKILGKQADSKVVIVARSSQWAVQEFLAGPQSRMFVNLIVIGQSFKD
GEDDSLEAPYILYTHKLYTDGLGASIPVVLNSWSHGKFSRSVNLFPAKMSQGYAGHRFVV
AAANQPPFIFRTIKTDLDGGNPRVVWEGVEMKILSLLAERNNFSIEVKEPQDLHLGSGDAV
TREVKSGRADIGVAGMYVTGERTRDMDLSYPHSQDCAVFVTLMSTALPRYRAILGPFHW
HVWLALTLTYLFGIFPLAFSDKHTLRHLLHNSGEVENMFWYVFGTFTNCFTFVGKNSWSK
TTKITTRLLIGWYWLFTIIITSCYTGSIIAFVTLPVFPETIDTIQQLLDGFYRVGTLDRGGWEK
WFLNSSDPQTNKLLKKLQLVPDVASGIRNTTKAFFLLPFAYLGSQAELEYIIQSNFTRNKKN
KRAMLHISNECFVPFGVSLSFPNNSIYSAKLSGDIARMVQSGLINKIMDEVRWEMQRGAIG
QRLAVSPGSINIVSVEEKGLTLADTQGMFLLLAAGFILGASALISEWMGGCTRKCRLTKKE
ETPSSANSREHLIPTPKSDMDAEIKIISNSGDSRFHLNPRGSSADSRDSLEGTIINLTTENISV
HDQFSVNGWDSRRSSSVDIDREVKEIFEKDESRRRAKSTAGVELTDSQRQATASKGAFGA
HLSEP
>EposIR25a
MSSLTILLLFLFVPVSLSQTTQNINVLLINEENNALAEKSFEVAKEYVRRNPTLGLAVDPVI
VVGNRTDAKAFLENVCRKYNDMLSAKKTPHVVLDFTMTGVGSETIKSFTAALALPTISGS
FGQAGDLRQWRNLNANQSKFLLQVMPPADILPESIRAIVTKQDITNAAIIFDEFFVMDHKY
KSLLQNIPTRHVITPIKSFNKDEIKTQLRSLRELDIVNFFVIGSLRTIKNVLDAADENQYFGR
KTAWFALSLDKGDISCGCKDATIVYMRPTPDAKSRDRLGKIKTTYSMNGEPEITSAFYFDL
SLRTFLTVKSLLDSGKWPNDMRYITCDDYDGKNTPNRTLDLKSAFQEIKETPTYAPFFIPED
DPMNGRSYMEFSTDLTAVTVKDGASIGSRSLGSWKAGLSNPLSLTDPENMSDYSAQLVFR
VVTIEQNPFIIRDDDAPKGFKGYCIDLIEEIRQIVKFDYEITLAPDGNFGVMDDNGNWNGII
KELIEKRADIGLTSLSVMAERENVVDFTVPYYDLVGITILMKLPRTPTSLFKFLTVLENDVW
LSILAAYFFTSFLMWVFDKWSPYSYQNNREKYKDDEEKREFNLKECLWFCMTSLTPQGG
GEAPKNLSGRLLAATWWLFGFIIIASYTANLAAFLTVSRLDTPIESLDDLSKQYKIQYAPLN
GSAAMTYFERMAHIEVRFYEIWKEMSLNDSLSDVERAKLAVWDYPVSDKYSKMWQAM

KEAGLPNSVEEAVQRVRDSKSSSEGFAWLGDATDVRYHVLTSCDLQMVGDEFSRKPYAIA
VQQGSPLKDQFNNAILQLLNKRKLEKLKENWWNNNPEAMKCEKQDDQSDGISIQNIGGV
FIVIFMGIGLACITLGVEYWWYKWRKRPLVGDVTQVEPSKSTRNNADHGTTKIGEGFTFR
SRNLGLSNLRSKF
>EposIR41a
MLEPPIILHPVEVLLQILINKYLLSSFCITMVTETELIIRPPSNLSFMYIYPEYNLTDQILDASE
KGCSDFIIQMNEPENFMDAFETVNHVGDIRRSDKKLIFLPRQDDNFNASVLMDILGLKASG
FVANILLVLPSINCTDDCNYYDLVTHNFVGTDDEVDQPLYLDRWHFSQEQVDNGVNWFP
HDMSNMNGKTLKVAAFTYKPYVLLDLDPSDNALGRDGIDVRIIDEFCRWVNCTVELVRD
DENEWGDIYDNLTGVGILGNVVEDRADMGITALYSWYGEYRVMDFSAAFVRTAITCIAPA
ARVLSSWDLPFLPFARLMWVCLIFTFFYASFALFIAQRSTDKIFMSTFRMMITQARDDTADS
WRIRSIAGWMLVTGLIIDNAYGGGLASSFTVPKYEASIDTVQDIVDRKMEWGATHDAWLF
SISLSEEPLVKELISQFKTYPEDELKRKSFTRSMAFSVEHLPAGSFAIGEYITKEAAEDLELM
LENFYYEQCVVMLRKSSPYTAKLSELVGRLHQSGLLLAWESQVALKYLDFKVQLEVKLS
RSRRDVDEVKPLNLKQLVGIFIIYFGGLSICLICFLVELLTRCGKASIVI
>EposIR68
MWITVLLVLLITGAIFYCLARFYTNLLQYQSDHSHVVVTAKDQDEKPVGMYLFGDIINSIL
YTYGMLLVVSLPKLPTGWAIRLLTGWYWLYCILLVVSYRASMTAILANPAPRVTIDTLQEL
VDSKVTCGGWGTETKHFFEESIDDIGQKIGERFEMIDDPNEAATKVAQGVYAYYENEYFL
KYLSVKRKNSDDKMSIETQNNSTNATVQVKLDSERNLHIMTDCVVNIPISLGFHKNSPLKP
LADIYMRRTVEVGLVEKWMNDVMYPIRALDTTDNEIKALMNLKKLYGAFIALAIGYFLSL
VCLLGEVIYWNCIVKRDPRFDKYAMDLYYEKKK
>EposIR75q.1
MKVVLIASVVLLCLSFVSMNDAKILKMVVNVIQSFNKPCHVVANLCWGSFEKKQLMDGL
GSVDNPKTVKFVQNMDLDHFVLREKTVFLVDLSCPSVTSFFNKANSSRIFNKPYRWLIVG
SSVDEGFPSELDNLQILPDSEVYLSQSNGNDSFIINLIYKIKLGRDWISEYYGTWTNNGVIK
SRISETSVAMRRKNLAQETIVTSMVVTDDSTITDLFELRYPLIDSVSKSLTQQLMPLYYFMN
ATARVIHSDTWGYFVNGSWSGMLGDMVEGRAELAGTLLFITEQRIEVQEFLTYPSSFPVRF
IFHEPPLSYQNNLYLLPFNTSVWYCCGSFVGVMIVVLLINAQWETKKLKKESANTVLFPSV
SEVTVFVVSAITQQGSTVELKGSLGRVVIFILFLAFLFLYTAYSASIVVLLQSSSNQIRTLSDL
LQSKLEIGAEDTPYNRFWFAAAKDPVRKAIYEKKLAPKGSIPKFYGMVEGIKMMQNKPFA
FHANLGVGYQIIQQYFQEHEKCGFQEITFLQDSTPWSSCYKLSPYKEIFKIGQIRIQEHGIAN
RINHLIFAKRPVCSVRGGRFVSVSIVDCYPSMLVLLYGLVLAVMILFVEILYDKRCSARKRV
SASIVSSSRGTVSSSSGLPSVSIDF
>EposIR75b
MKLLFLLNFALCLSSIFSLDSDDISMIVSFSQQDGRSTSVLAPYVCWSSYEVATLAKSLHEV
GVNAARSLQPRRTENYLQNLLILADLGCAGTDEFLIKANDEGFFKSPYRWLLITKDPEDLG
ILDRIVMLIDSDVVLAQKTSDGYKLIEVYKVIANSEVIFTTRAKWYPNGGTLKNTTTDGN
NTALTMDDKITSITVVEDKYGVLEDHRPSKILSTRRMDIRKHTLTMVNVITDSNETRKHM
DDRLQLHQDSITKMSYMVVKICFEMMNSTEKLIFTNTWGYVDKNGSWNGIIERLIKKEG
DIGTLTIFTQERMKNIDYIAMVGTTAVRFVFREPPLAYVSNIFALPFTGAVWLAILVCVLAC
ALFLYITSKWEATMGMHPLQLDGSWADVLILIIGAVLQQGCTLEPRHAAGRVVTLLLFVAL
TILYAAYSANIVVLLRAPSSSVRSLQDILNSPLKLGASDFAYNRYFFKKLNEPLRKTIYNKKI
APKGKKANFYTMKEGVEKIRRGLFAFHMELNPGYRLIQETYQEDEKCDLVEIDYINEIDP

WVPGQKRSPYKDLFKINFIKIRESGIQACIHQRLHVGKPRCLGAVNTFSSVGITDMYPAML
ATLYGMLLAPAVLLLEIAYKRLMEMREKRKAILQSDHDAT
>EposIR75q.2
MKTYCLMVSLIFFSGGCYAENETKLFMVSDVIQSMQRPSAVIAMLCWSSRMKMQLYSAL
GENDTEIKMMQFFKAGKVPQLDAQAQHVVFLADLDCPGISSYLTMSYLEKHFRSPFRWL
LIGTGSKNTGNEISIPEALASVDLLVDSEVILAQHLEHGSYELHLIYKVGPNTEWKKELYGT
WDKNKRLQTSLMEGELILRRLNLENYEIPISYVLTDNDSINHLFDNVNDHIDTITKVNFPTT
NHLLDFLNASRKYVFTNTWGYRVNGTWNGMTGYLVREEVEVGGSPMFFTSERVSIVDYI
SSPTPTRSKFVFRQPKLSYENNLFLLSFRAAVWYCTIALLNLLVLAVFIAAVWEWKKTHGH
ENEKSETDASILRPSLMDVTLLILGAACQQGSTVELKGSLGRVVMLVLFLALMFLYTSYSA
NIVALLQSSSSQIKTLDDLLHSRIKFGVHETVFNKYYFSTATEPVRKAIYETKVAPRGSKPRF
MSMEEGVKKMQKGLFAFHMETGVGYKFVGKYFQESEKCGLKEIQYLQVIDPWLAVRKN
TPFKEMFKIGTKRIQEHGLQSRETRLLYEKRPKCSGQGGSFVSVSMVDCYPALLVLSYGSI
VAIVLLFMEIMSAKKSFIYGKICRTTETED
>EposIR75e
MFLFVLMCICLANTRASHLNGDFVKDFLRREERPATIVSHLQLTKREQVHLSKTFFANNKQ
FQIALETPITNNEHVVHLADCRNNYTWEILQNAINDKSIRSPARWLLLLEDDLGNDLDSVQ
INDYKSDIERRLLDSYVFMDTEVYVACQRENHVELFSVYKTKPHLPLIWEEHGVWSQAGF
SKPRPAPLAMRRRSLGGAPVVAATVILDNRTLEHMPDYLHREVDTLTKLMYYMTMHLVE
WVNGTRVINRTGSWGYRQPDGRYDGVVREMQDGRADLSGTVMIPTIERSKYMNFVLPPA
PVEAKFIFKKPSLASVTNIYVLPFSIGVWLSILVLIFVSSLTLFLSYYREKVVNPRYNMSWPQ
KVSEGLFETLCLVFQQGTAGDPISIAGRQILLLGLMAFMFLYTAYSANVVALLQSPTNDISS
AETLLTSPLACGAQDVLYNRNMFLHETRPIHRALSSRKILPQGEKAFLSVDEGIRKVREGM
YAFHVELTAGYDQIQKTFLEDEKCNLGAIKFMSITYPYLALAEGSHIKEQLRIGANRIMEG
GVQRRTARRMLPAAPRCGAGAATFAAVRLRDVAPALRALLALYALALPVCALEVLARRRE
LRNAEKKADEEDTPEDISENFIS
>EposIR75d
MELASFITVYFAAKQLSLLTAFICWKPEELLVLQRETARAGLRLRTPRGAXALAPDGDRRE
AMLLDLXCPGAQLILDQASASRAFNLRHSWLLLDXGPFNASXVQXTLAXTLVLPDADVA
LAADDALVDVYRXRADQPLALAPLGAGPAEWAAXPAAPTRRKDLNNVYLKAATIISQPQ
HFKGWADLTMRHIDTFPKLTYPLMLLLAEDLHFRYNLKQVDLYGEERXGAFDGLAGQLQ
RAEIEVGITSMFMRXDRTRVLHYCSETVELRGAFIFRQPSKSSVSNVFLLPFSRGVWAATA
AVLAAATALLAALARQARLRAVDPMLEQLSLGETVIFTVGTVCQQGFNLVPAIASXRVVM
FCALMTSLFAFTSYSAKIVAILQTPSDAIRSIDDLTRSPMTLGVQETTYKRVYFAESTLPATQ
QLYRXKLLPLGERAYLSVVDGVERLRTGLFAFQVEEPSGYDIISKTFTEREKCGLQQIQAFK
LPMVAVPVRRHSGYKELFATRLRWQREVGLMERSRXMWLAARPRCDAGAGGFVSVGLI
DIISALHVLAAGMALSLVMLAAERAAQRCGPPRLRARRXEELVT
>EposIR75p
MSGRIIQWVMFASLMALYAAYAANIVVLLQAPSNSINNLAQLAKSKMTLAGYDADYNNF
LFKGTTDPVRRAIAKRVDPERGPKPFYSLEEGVERIRKGLFAFHVVSDPTYRQVEKTFFESE
KCDLVEIDYAGFKKFYMPAYKHSPYLELLRVVFKHIREVGIKSAINFRLETGKPSCKNAAA
MFVSVGVMEMRSVMLFMVYGVGISXAILLVEILVFHLDRYRQNRSAVRVGQVNQVCVGI
TWQESPKLGGKGAIFGRNRRSMDTEIQPCSTLVEG
>EposIR76b

MAGMELIISSFCNATFCDVIYDSPSPLTEHQAEVIATKNELNGKHLKVATYDNYPLSWVATT
ENSTLNGRGVAFVIFDILREQFNFTFDVITPSQNFEIGGSEPEQSLIGLVNSTKVDMAVAFVPI
LYKYQSMVDFSSILDEGVWNMMLRRPKESAAGSGLLAPFEVHVWYLILAAVLSYGPCITF
LTYVRSKMVKDDESHIPLSPSFWFVYGAFIKQGTTLAPEANTTRVLFTTWWLFIILLSAFYT
ANLTAFLTLSKFTLDIENPQDILKKNYRWVAPQGSTVQYTVGDVDSDLYYLNKMVANGRA
QFPSVNADKQFLPLVAGGAVLVKEQTAIDHLMFADYVRKTKDGVAEADRCTYVVAPHPF
MEKLRGFAFPRGSKLKPLFDPVLTYLLQSGIVKFLEHRDLPSTKICPLDLQSKDRKLRNSDL
TMTYMIMVTGLAAAIGVFVGEMMIRRYVRVKMKNMRGDTKTKLPKNAAMTRRFKLDD
SRPPPYDSLFGNNSRYKMTGKSKRKIINGREYWVVGTVNGESRLIPVRTPSAFLYQRDK
>EposIR87a
MRSRLFLLYLCFIHFAAAKTNPLLMPSEDSGKLVKAAECVVKMSAKYFVEHKALSGSIIIIS
MNSMVSIAQRAMIDTIHRGIKSTVMVKDSLFPHANASHFREIAKNYMFILESQQELTRDVI
QLNKLPTWNPLAKAVVFYQLQPGEDGEQISIDFINLMRGYKLFKSIIFMFSPENEEVISYSW
APYSDTNCGGKCESVYILDKCTNGKVRQVHSQIDMFPLNMKQCPLVTYAVVSEPYVMPP
VRKLTDTAYDDAYEFEKGGEIKLVKLISEFTNMSLIVRMSDVTENWGLIDANGTATGAFGV
LRNDSVDLVIGDIEVTRTIRKWFHPTISYTQDEMTWCVPKSAQASTWNNLVIIFQWSTWVA
TFLSIVIMGLIFHYFYYRENNRKVTKWPTNSWLYTLSMLLGWGASFNPKSATFRILIFAWL
FFGLIMGISYESFLRTFLMHPRYEKQISTASDLIRSEIPLGGRGIYRSYFETNNESSFYLYRKY
VETSFSDGIRRAALGRNFAAVSSRRQAEYQDQKLGKGRPLLYCFKEGNNLYKYGVVLVAK
RWYPMLDRLNNMIRRVSENGLIEKWNQELFIHSVSADSSGVVESLKIQHLLGAFMFIGLM
YAFSVLVLIGELAMGSLDKRRNNKTKEKAVYRVKLI
>EposIR93a
MRIWLLVFCIAGVRSEEFPSLITANASIAVVLDRQYLGEQYQALLEGLKDFIKELTRVDLKH
GGVVVYYYSWSTISLKKGFIAVFSIASCEDTWSLFSRIEEEELLLFALTEVDCPRLPPDSAIT
VTFTDPGQELPQLLLDLRTNKAFNWKSAIILHDDTLNRDMVSRVVQSLTSQIDDENIPTISL
TVFKMKHEINEYLRRKEMHRVLSKLPVKHIGENFIAIVTSDVMSTMAETARDLGMSHTQA
QWLYVISDTDNRRANLSMLINALYEGENIAYIYNITEDREDCRNGLMCYAEEMMTAFVSA
LDAAVQEEVDVAAQVSDEEWEAIRPSKLQRRDMLLKHMMQHITVNSKCGNCSTWRAIA
ADTWGSTYSGYVDTSDTGDNETTGVIEQINLLQVGYWRPIDGPRFTDYLFPHIAHGFRGK
VLPVITYHNPPWTILKANESGSISSYTGLIFDIVNQLAKNKNFTLKLLFPGDIKNALSNKTS
AEGTYSQSAMLTMMAVAKRQAAFAAASFTVLPDKNPGINYTVPVSTQPYAFMIARPRELS
RAMLFLLPFTTDTWLCLGFAVVLMGPTLYIVHRLSPYYEAMGVTRQGGLATIHNCLWYIY
GALLQQGGMYLPRADSGRLVVGTWWLVVLVVVTTYSGNLVAFLTFPKQEMPVTTVAELL
DNRALYTWSISKGSYLEMELQNSDEPKYVSLLKGAELTSESSGMEGHLQTRSPLLMRVRS
QRHVIIDWKLRLSYLMRAEHLATDTCGFALSTEEFLNEQVAMIVPAGSPYLPVFNKEINRM
QKAGLIAKWLSAYLPKRDRCWKTSSIAQEVDNHTVNLSDMQGSFFVLFLGFVTATSVLLI
EWFYHRRKSQKEDVAIKPYVE
>DmelIR25a
MILMNPKTSKILWLLGFLSLLSSFSLEIAAQTTQNINVLFINEVDNEPAAKAVEVVLTYLKK
NIRYGLSVQLDSIEANKSDAKVLLEAICNKYATSIEKKQTPHLILDTTKSGIASETVKSFTQA
LGLPTISASYGQQGDLRQWRDLDEAKQKYLLQVMPPADIIPEAIRSIVIHMNITNAAILYDD
SFVMDHKYKSLLQNIQTRHVITAIAKDGKREREEQIEKLRNLDINNFFILGTLQSIRMVLES
VKPAYFERNFAWHAITQNEGEISSQRDNATIMFMKPMAYTQYRDRLGLLRTTYNLNEEPQ
LSSAFYFDLALRSFLTIKEMLQSGAWPKDMEYLNCDDFQGGNTPQRNLDLRDYFTKITEP

TSYGTFDLVTQSTQPFNGHSFMKFEMDINVLQIRGGSSVNSKSIGKWISGLNSELIVKDEEQ
MKNLTADTVYRIFTVVQAPFIMRDETAPKGYKGYCIDLINEIAAIVHFDYTIQEVEDGKFG
NMDENGQWNGIVKKLMDKQADIGLGSMSVMAEREIVIDFTVPYYDLVGITIMMQRPSSP
SSLFKFLTVLETNVWLCILAAYFFTSFLMWIFDRWSPYSYQNNREKYKDDEEKREFNLKE
CLWFCMTSLTPQGGGEAPKNLSGRLVAATWWLFGFIIIASYTANLAAFLTVSRLDTPVESL
DDLAKQYKILYAPLNGSSAMTYFERMSNIEQMFYEIWKDLSLNDSLTAVERSKLAVWDYP
VSDKYTKMWQAMQEAKLPATLDEAVARVRNSTAATGFAFLGDATDIRYLQLTNCDLQVV
GEEFSRKPYAIAVQQGSHLKDQFNNAILTLLNKRQLEKLKEKWWKNDEALAKCDKPEDQ
SDGISIQNIGGVFIVIFVGIGMACITLVFEYWWYRYRKNPRIIDVAEANAERSNAADHPGKL
VDGVILGHSGEKFEKSKAALRPRFNQYPATFKPRF
>DmelIR10a
MAVLGTVFLLFMLDLKTLNLTRLNGLLVEPTRDLPQLELWLRAGSDHQDAENPYVQWFL
LRTEIPLSIVTYQENRYWMDDPFGRRNLVLVMSLDQLLTNRGAAAPIQKASTFFYILADQD
KDLSADEQLRLEGSCRQLWTQHKVYNRFFLTRDGVWIYDPFKRRDSAFGRLVRYYGSET
LDKLLFRDMAGYPLRIQMFRSVYTRPEFDKETGLLTRVTGVDFLVAQMLRERLNFTMLLQ
QPEKKYFGERSANGSYNGAIGSIIKDGLDICLTGFFVKDYLVQQYMDFTVAVYDDELCIYV
PKASRIPQSILPIFAVGYDIWLGFVLTAFACALIWLTLRVINLKLRIVSLGNQHIVGQALGIM
VDTWVVWVRLNLSHLPASYAERMFIGTLCLVSVIFGAIFESSLATVYIHPLYYKDINTMQE
LDESGLKVVYKYSSMADDLFFSETSPLFASLNKKLSWNRDLRADVIDEVARFRNKAGVSR
YTSLILESSHFTLLRKIWVVPECPKYYTISYVMPRDSPWEDAVNALLLRFLNAGLIVKWIQ
DEKSWVDIKMRSNILEADAESELVRVLTIGDLQLAFYVVIGGNLLAFLGFLAEHFRWKLQ
KKGV
>DmelIR52c
MVWLIIILFCLGNSSSQILDVTNNSHLDFDYRLFGLLQRLQVEKSYDTLLVYGEDCAIPSLF
ERLQVPAVLVSSGSTNFDWNFSSLTLILSCNFQDEREENYRTLMKLQTSRRLILLKGHIKPE
SVCDFYSKKEQHNVAMVKENFYQLEVVYSCRLFQDQNYEKLNLFDGKSIYKDQFRNMH
GAPIRTLSDKEPPRTIPYIDSKTGEEKFKGYVGMLISQFVKKVNATMQIREDLIKDDEEVSF
VDITNFTSNDILDIGICEARTLEMSNYDAISYPYLMSSYCFMAPLPDSLPFSDVYMAIVAPSI
LIMFLIIFCICSVLIIYIQERSYRSLTIRSVLMNDICLRGFLAQPFPFPRQYNRKLKLIFMLVCF
SSLISTTMYTAYLQAFLWGPPIEPRLTSFDDVKKSRYTMAINIYEREFLEALNVSLEDVEIYD
YGKFSKLRSTFNTNYLFPVTALQWFTINEEQKLFKYKIFYYCDAFCLNQFDILSIPLRRHLP
YRDIFEEHMLLQKEFGLTKYWIDQSYRDMIRANLTTFKDFSPLLENDYIEVHNLYWVFTM
YFVGMGMGLCFFILEILRPLRYWRNCKIKCEYCYAFLKNFAK
>DmelIR56a
MGSRFFIRNLILFGLLASSNMQIPFGELEKKFELDVDFLLGVTELVGHIQGLYSITVYADCID
IHPSIQQRIMDKFMVPVNTIGSNLSRPNYHKLDNSRIRIVLFTGLNDTILVNLNKTDVPYSD
NFYMLAYASAIKNKCIELDFIEEVFTLLWKMSIQNAILLIRGEFMMEMWSYLYMGKIHKIK
LTKPNSYLESLRKYNYRFSLEVINDPPAIFWYNSSEQADVTGGGNLSVSGPLGLIIINFLRHL
NVTIDIVPIPGKQTSQYELFQQPDNLRAENGVNMVGSALLKYSPMVTQSRMCLLVSNRRM
IPFSRFLDRLVSPGVHKLTFVSSIGIFVIKYFSHRPRSFVDAIFCTIRFFFAIPLPSIILNRLPVVD
RFIEVFIIIFVQILLSSNISITTSALTTGFWEPPIINVETMRASGLHILTEDPTILQAFKENILPSS
LADLVILVDEDTYFHHVTTLNNSYVYVVQAHNWQIFRLYQQQMTNEPFEIASEELCSKWR
ILGIPLNPKSPLRFMFKDYFYRILESGLREQWVHSGFKKFCEFNNLKKLPVDSVDSWQPLSI
EFYSNVIRAYIIGLVIATLAFVAELLHNGYRRKNVKKT

>DmelIR11a
MRFAILWLFSGCLLPGIQVGIWVVVRAQPTGRDVLLSRLGNQQNELNTRRLANASSYLTR
NYIANRINTLVVREICVECPYELSERQRQLVDQILASLAPELSVLLHKGTAEETTWEYTLFV
VNDHTAFTGQVFIFPDELLEREFFCIVVVSEIQSRQFVRQTVGSIVKSNLQMHFVNVVVVA
QLEDGTVGTYSYKLFKANCTPGITVRQINHFDRITGKPQQSMPDLYPVRNGHLGDCPFNV
GAAHMPPHLIYKRHKDPPPASNVSIPAEDLAGIDWDLLQLLAKALKFRIQLYMPQEPSQIF
GEGNVSGCFRQLADGTVSIAIGGLSGSDKRRSLFSKSTVYHQSNFVMVVRRDRYLGRLGP
LILPFRGKLWGVIIVILLLAVLSTCWLRSRLGLSHPIEDLLTVIVGNPIPDHRLPGKGFLRYLL
ASWMLLTLVLRCAYQARLFDVLRLSRHRPLPKDLSGLIKDNYTMVANGYHDFYPLELTCR
QPLDFSARFERVQRAAPDERLTTIALISNLAYWNHKHPNISRLTFVRQPIYMYHLVIYFPRR
FFLRPAIDRKIKQLLSAGVMAHIERRYMQYENKRKVASNDPVLLRRITKSIMNGAYRIHGL
VIVLATGMFILELLAGRSNGRLRRWMEWVHQ
>DmelIR7b
MKYWLYILSCCSLVASTMESSSDWDLAEALAQVVANSEMGRFKTLYIYTHTNSQSTGGHL
EELLDQVLMIVPNNLQARRLLLQQSMEYKPYVHAVLALVDGLPSLSAIYARIRATQDLSHT
LIYMSMPTDAYGEEMQATLRFLWRLSVLNVGVVLRPPGDHILMVSYFPFSALHGCQVISA
NVVNRYQVGTKRWASQDYFPSKLGNFYGCLLTCATWEDMPYLVWRPDGSGSFVGIEGAL
LQFMAENLNFTVGLYWMNKEEVLATFDESGRIFDEIFGHHADFSLGGFHFKPSAGSEIPYS
QSTYYFMSHIMLVTNLQSAYSAYEKLSFPFTPLLWRAIGLVLILACLLLMLLVRWRHHHEL
PRNPYYELLVLTMGGNLEDRWVPQRFPSRLVLLTWLFATLVLRSGYQSGMYQLLRQDTQR
NPPQTISEVLAQHFTIQLAEVNEARILASLPELRPEQLVYLEGSELQSFPALAQQSGSSARVA
ILTPYEYFGYFRKVHPMSRRLHLVRERIYTQQLAFYVRRHSHLVGVLNKQIQHAHTHGFL
EHWTRQYVSAVDEKDESVARIASTSYSTLDGIDGDPSLSESEEDQQVAPVRQNVLSMREL
AALFWLILWANLGAVVVFVLELLLPRIKLRKILRKMKSDIKKQISKLVRK
>DmelIR21a
MSYYWVALVLFTAQAFSIEGDRSASYQEKCISRRLINHYQLNKEIFGVGMCDGNNENEFR
QKRRIVPTFQGNPRPRGELLASKFHVNSYNFEQTNSLVGLVNKIAQEYLNKCPPVIYYDSF
VEKSDGLILENLFKTIPITFYHGEINADYEAKNKRFTSHIDCNCKSYILFLSDPLMTRKILGP
QTESRVVLVSRSTQWRLRDFLSSELSSNIVNLLVIGESLMADPMRERPYVLYTHKLYADGL
GSNTPVVLTSWIKGALSRPHINLFPSKFQFGFAGHRFQISAANQPPFIFRIRTLDSSGMGQLR
WDGVEFRLLTMISKRLNFSIDITETPTRSNTRGVVDTIQEQIIERTVDIGMSGIYITQERLMD
SAMSVGHSPDCAAFITLASKALPKYRAIMGPFQWPVWVALICVYLGGIFPIVFTDRLTLSH
LMGNWGEVENMFWYVFGMFTNAFSFTGKYSWSNTRKNSTRLLIGAYWLFTIIITSCYTGS
IIAFVTLPAFPDTVDSVLDLLGLFFRVGTLNNGGWETWFQNSTHIPTSRLYKKMEFVGSVD
EGIGNVTQSFFWNYAFLGSKAQLEYLVQSNFSDENISRRSALHLSEECFALFQIGFLFPRES
VYKIKIDSMILLAQQSGLIAKINNEVSWVMQRSSSGRLLQASSSNSLREIIQEERQLTTADT
EGMFLLMALGYFLGATALVSEIVGGITNKCRQIIKRSRKSAASSWSSASSGSMLRTNAEQL
SHDKRKANRREAAEVAQKMSFGMRELNLTRATLREIYGSYGAPETDHGQLDIVHTEFPNS
SAKLNNIEDEESREALESLQRLDEFMDQMDNDGNPSSHTFRIDN
>DmelIR60a
MWCNNPGLIIIIFLGQILNLCQGIVNLSNETANTVIFMLPEKDLGPDVWKAGVGCLDSFAQI
FFFRNPKERFTRAYNLMLVHAFHLSSPADQIQEGFSKLINEAVTNPGPPDREELFQMRVASD
YNITNGTEDKGELILADNYVIVVDSVDRLKELMKKKIVEMRSWNPGARFLVLFHNATCRN
RPLGVASNIFKDLMEMFYVHRVALLYANSTMNYNLLVNDYYSNVNCRILNVQSVGQCHD

GKLYPNNAVVKASMQDYVSGFSPRNCTFFACSSISAPFVEADCILGLEMRILGFMKNRLKF
DVNQTCSLESRGEMDGPANWTGLLGKVQNNECDFVFGGYYPDNEVADHFWGSDTYLQD
AHTWYIKMADRRPAWQALVGIFEAYTWIGFILILIISWLFWFTLVMILPEPKYYQQLSLTAI
NALAVTISIAVQERPICETTRLFFMALTLYGLNVVATYTSKMIATFQDPGYLHQLDELTEVV
AAGIPFGGHEESRDWFENDDDMWIFNGYNISPEFIPQSKNLEAVKWGQRCILSNRMYTMQ
SPLADVIYAFPNNVFSSPVQMIMKAGFPFLFEMNSIIRLMRDVGIFQKIDADFRYNNTYLNR
INKMRPQFPETAIVLTTEHLKGPFFILVVGSCWAALTFIGELIIHRWRTQLVSTSEQQDRRSD
KRRRRRRRRKPEKDNRWQRQVQVAPVVRFTPVKRRKVFQGQTSQK
>DmelIR68b
MKFLVGLLLQWYLPGIYALAEIACRIAVEQNVQVTYLYRCASCPASFDADYSALELDLYRC
VGSRLPVITRNMEAHELEPFRRTDSLSIFQIPAAEKGDSLVRRILDMLNPHQRRKHMHKYL
FVWPNAGRHQLLRLFRGSWAKKLLYGLAITGRENGTFDFDPFAWGGLQVIQRLDGEVPY
ARKVKDLRGYPLRFSMFTDPLMAMPRSPVETAGYQAVDGVAARVVGEMLNASVTYVFP
EDNESYGRCLPNGNYTGVVSDIVGGHTHFAPNSRFVLDCIWPAVEVLYPYTRRNLHLVVP
ASAIQPEYLIFVRVFRRTVWYLLLVTLLVVVLVFWVMQRLQRRIPRRGVIQFQATWYEILE
MFGKTHVGEPAGRLSSFSSMRTFLMGWILFSYVLSTIYFAKLESGFVRPSYEEQVDRVDDL
VHLDVHIYAVTTMYDAVRSALTEHQYGLLENRSRQLPLGIATSYYQPVVRRRDRRAAFIM
RDFHARDFLAITYDSQAERPAYHIAREYLRSMICTYILPRGSPFLHRLESLYSGFLEHGFFEH
WRQMDLITRVGASPDAEEFLEDLGDQTDTDSGSNELAIRNKKVVLTLDILQGAFYLWSVG
IGISCLGFAVEHAHWFWRRQTLRNAVEARTS
>DmelIR56d
MDNRAAELILRERNIFPTNGSDNITLLNNMFVLEMFYRITQLYHFKNFIFYISERLDLNNKD
SQEFFHNFWTYFPMAPNLIITREHHLGIPMMQFISTPSLVMVFTTGKDDPIMELASHNQQGI
HWLKTIFVLFPSLQSRDFETNPESLAQFTAEIKDVYDWVWRKQFINTFLITIKDNVFILDPY
PTPSIVNKTGVWQAEEFFHKYAKNMKGYLVRTPILYDMPRVFKSDRPTNRYEKNFIHGTS
GNLFLGFLEFVNATLMDTSANVTADYLNMTNLLDLVSQGVYETLIHSFTEITTKFVVSYSY
PIGINDCCIMVPYRNQSPADQYMHEALQENVWVLISLFTLYITVAIYLCSPLRPRDLSAAFL
QSICTLTYSVPTFIIRTPTLRMRYLYILLAIWGIVTSNLYISRMTSYFTTAPPVRQINTVQDVV
EANLRIKMLAIEYERMAKSPLQYPESYLNQVDLVDKHMLDLHRDPFNTSFGYTVSSDRW
RFLNLQQLHLRKPIFRLTEICEGPFYHVFPLHKDSHMRSVMTEYIMIAQQAGLMNHWERE
TFWEAVHLHRIHVHLFDDEPMALSLDFFSSLLRTWTLGLILAGLAFAAEMKWHEHVTFKR
RPVIRITRKPRSFLRRFMKL
>DmelIR51b
MCKVLTLLVVILLLALTNAAYNVTLLKSVLSLISTREPWINTPIFVGHNTQGGDLNDLIIWL
HQTMGVTSLTMNLFLQPEHIRPLGHFKITRYNGIALFFCHDKHDIMWLTLDRNLRKLRRIR
LIIILRNQRSGSQGAIKSIFNALWQYQFLNVLVLQRDQLYSYTPYPAMRFFKLDIHTEPLFPH
AARNFHGYVVSTPAENDIPRVFHVHDPLTKSRKVLGYAYRTFVEYLDHYNASLRLTNPDE
NLDPTTSVNMNHIVQLIIDGQLEISLHPYVFTPPTATKSYPLLIYPNCLIVPMRNEIPRHMYL
LRPFQLYSWYILLFAVFYITGILYCISPKLNKSSWPQRLGLNFLDAISKILFISPPITIYRPTWR
HLIIFLQLSVLGFMSTSWYNIELDSFFTTIVVGEQVNSMDQLVHQQQRVLVKEYEINTFLRH
VEPRLVEKVSRLLVPVNASEQVSALLSFNRSFAYPFTEERWQFFAMQQQYAFKPIFRFSSAC
LGSPHIGYPMRVDSHLETSLNHFILKIQDTGLLNHWVVSDFNDAMRAGYVRFVDNVLGY
QSIDVDTLRLGWCVLGIGWILSALVFSCEYWHLYPWRFIA
>DmelIR48b

MILQQSSNLLKLLLLLAISSVRTQGLNDIIIELNQRLLISNNFLYCNQSDKLNEYEIKYLQHM
PPISLMIFTSIESMNFTQVEYNLGADNKLFLIMGNEEPPYDFLHALNLHFQFAEYIIVIDEPV
DLKKSTKWLDFVNHLWQQGYVQLLIYTSYDEKLYHKIIFPETVIEETLVEQYISIRGSFNNL
YGYPVRVAAYNNAPRSMLYVNRWGKHIFAGFYMRFLRAFIDARNGSFVPVLTPSNSPGNC
TLNLVNETVDVCADALAANPAAFSLTHGFRIASANVLVTHAKPLHSYRYLTAPFQWSVWA
CLVIYVLLVVNFLSFIGWLRSGKWEFSKYLLEVFSSLLFSGFYLKEIRGRERYILFGVLFIAG
FVYSTEYLGLLKSMLISEVFEKQIDTFEALVESNITLMVDPYDKILFAKYNMPEILSPIMELV
SFETLLKHRNRFDQDYAYILFSDRMALYDYAQQFLKHPKLLRIPIDFSFLYTGIPMRKRWFL
KHHLGRAWYWAFESGLTRKLALDADFEAVRVGYLSFLITEHVEAQPLNVDYFVMPAIALA
IGYILALLSFVIEMTAWRIREFLGCRKATMTSTGCSEGGHVDVD
>DmelIR7a
MFHHLWLLMGLRSLAMGALHPPQPEAMTPLVAAALEILAEQVSPSQSTLAVMDLTQDAE
HRDERQEQLMTIILRSVGSEMALRTFQKPPAEVPASFVVFLVNSAQAFNTLGFHFTDIHSTR
EFNFLILLTHRMSSRAERLQVLRDISRTCVRFHTSNVILLTEKRDGVVLVYAYRLLNMDCD
LSVNLELIDIYKNGLFRHGHEARSFNRVLSLSGCPLQVSWYPLPPFVSFIGNSSDPEERAQI
WRLTGIDGELIKLLASIFDFRILLEEPCNKCLSPDIKDDCSGCFDQVIISNSSILIGAMSGSHQ
HRSHFSFTSSYHQSSLVFIMHMSSQFGAVAQLAVPFTVIVWLALVVSSLLLVLVLWMRNRL
VCGRSDLASHALQVLTTLMGNPLEARSLPRSSRLRILYAGWLLLVLVLRVVYQGKLFDSFR
LPYHKPLPTEISELIRSNYTLINQEYLDYYPRELTVLTRNGSKDRFDYIQGLGKEGKFTTTSL
IATMEYYNMMHWSTSRLTHIKEHIFLYQMVIYLRRHSLLKFAFDRKIKQLLSAGIIGYFVRE
FDACQYRKPFEEDYEVTPIPLDSFCGLYYISLIWLSAAVVAFILELLSQRIVWLRRIFE
>DmelIR64a
MHWWLLVFLPLSCQGLPEHELLELELDYGLAEPQRTSLLQSSLILQFSQDYKHIPRITYFTC
QKPHLQTPNQIPNAAEHRDAFAAKNFQLIKSLYESELFVRIVLLDVLAQSPSSGRPNRPGSG
PTGGFSQTPSQAQSNSEWLEGVLRMEALRQIAVVDLACGAVSRRFLELASAKMLYSEKFH
WLLIEDFAWHGRTQTAEGSGKRDDGEMEEEEPPGQQIQATDDEDLPSIESFLGGMNLYMN
TELTLAKRMSEAAHYTLFDVWNPGLNYGGHVNLTEIGSFTPTEGIQLHTWFRTTSTVRRR
MDMQHARVRCMVVVTNKNMTGTLMYYLTHTVSGHIDTMNRFNFNLLMAVRDMFNWT
FVLSRTTSWGYVKNGRFDGMIGALIRNETDIGGAPIFYWLERHKWIDVAGRSWLSRPCFIF
RHPRSTQKDRIVFLQPFTNDVWILIVGCGVLTVFILWFLTTIEWKLVPHDGSALIKPKGGAP
PRHHYQQQQQQEQVEAPVRPITAVSVVVSKEKVEEKQEEYEDSTPIDAGTLWQRCYQKL
NKYIKDRKAKQKKAPERVGLFLESVLFFVGIICQQGLGFSTSFVSGRCIVITSLLFSFCIYQF
YSASIVGTLLMEKPKTIKTLSDLVHSSLKVGMEDILYNRDYFLHTKDPVSMELYAKKITSV
PTTKENEADEDEPVDPNPASTDPAKSYRDIVHSHETGAHAKDNAASNWLDPETGLLRVKH
ERFAFHVDVAAAYKIIAETFSEQDICDLTEVSMFPPQKTVSIMQKNSPMRKVISYGLRRVTE
TGILTYHFNVWHSRKPPCVKKIETSDLHVDMDTVSSALLILLFSYAITLMILGTEILYSKWH
NRIQLKWVGAT
>DmelIR93a
MNPGEMRPSACLLLLAGLQLSILVPTEANDFSSFLSANASLAVVVDHEYMTVHGENILAH
FEKILSDVIRENLRNGGINVKYFSWNAVRLKKDFLAAITVTDCENTWNFYKNTQETSILLI
AITDSDCPRLPLNRALMVPIVENGDEFPQLILDAKVQQILNWKTAVVFVDQTILEENALLV
KSIVHESITNHITPISLILYEINDSLRGQQKRVALRQALSQFAPKKHEEMRQQFLVISAFHEDI
IEIAETLNMFHVGNQWMIFVLDMVARDFDAGTVTINLDEGANIAFALNETDPNCQDSLNC
TISEISLALVNAISKITVEEESIYGEISDEEWEAIRFTKQEKQAEILEYMKEFLKTNAKCSSCA

RWRVETAITWGKSQENRKFRSTPQRDAKNRNFEFINIGYWTPVLGFVCQELAFPHIEHHFR
NITMDILTVHNPPWQILTKNSNGVIVEHKGIVMEIVKELSRALNFSYYLHEASAWKEEDSL
STSAGGNESDELVGSMTFRIPYRVVEMVQGNQFFIAAVAATVEDPDQKPFNYTQPISVQKY
SFITRKPDEVSRIYLFTAPFTVETWFCLMGIILLTAPTLYAINRLAPLKEMRIVGLSTVKSCF
WYIFGALLQQGGMYLPTADSGRLVVGFWWIVVIVLVTTYCGNLVAFLTFPKFQPGVDYLN
QLEDHKDIVQYGLRNGTFFERYVQSTTREDFKHYLERAKIYGSAQEEDIEAVKRGERINID
WRINLQLIVQRHFEREKECHFALGRESFVDEQIAMIVPAQSAYLHLVNRHIKSMFRMGFIE
RWHQMNLPSAGKCNGKSAQRQVTNHKVNMDDMQGCFLVLLLGFTLALLIVCGEFWYR
RFRASRKRRQFTN
>DmelIR87a
MSTPEQRFWLAALLFLLSQHSEVRGFGINLMKVQTEDKGQEACILALLRKYFDSGDGLSG
SVLCINRNYQLPNIEEQLLRGVNNYENYPWSLLITNSREGPSPAKFLMNEKPQCYFLIVDN
LEDEDLDEVFEHWKGMVNWNPLAQFVVYLASLEETDEEMNDLMVELLLTFINKKIFNVN
VIGQSEENQFYYGKTVFPYHPDNNCGNRVISVELLDACDYPSEETDSEDENDEDEGDGAQ
EEDDGPQEEGDGEQEEEDGPQEQEDGDQAKGDEGQENDDGGLENKVENEFRIGASDDD
ELENDLSSNSSEPEAIIEEFFRAKFEDKFPRDLSGCPLTASFRPWEPYIFRNSEEQPVDDYYY
GLQGDEDDYNDTSPNYGESDDESYADPGEDGDGAIPDTETQSGGKLKLSGIEYEMVQTIA
ERLHVSIEMQGENSNLYHLFQQLIDGEIEMIVGGIDEDPSISQFVSSSIPYHQDELTWCVARA
KRRHGFFNFVATFNADAGFLIGIFVVTCSLVVWLAQRVSGFQLRNLNGYFPTCLRVLGILL
NQAIPAQDFPITLRQLFALSFLMGFFFSNTYQSFLISTLTTPRSSYQIHTLQEIYSNKMTVMG
TSEHVRHLNKDGEIFKYIREKFQMCYNLVDCLNDAAQNEHIAVAVSRQHSFYNPRIQRDRL
YCFDRRESLYVYLVTMLLPKKYHLLHQINPVIQHIIESGHMQKWARDLDMRRMIHEEITRV
REDPFKALTFDQFRGAIAFSGGLLLVASCVFAFELCYVKYVYRTEKRERKTKKITKKVHNI
KIQHD
>DmelIR7e
MNISALLNSYYDLSGEQMNHINEFVARAVLHVVHHYILSVTPSLVLTLCCRSNHTCNFYNK
MMSTLFREWGLAPLQIVNVLRGVPWHPVPGRRHFNVIFTDSFAAFEEIRMEYYSREYNYN
EHYFIFLQARDRLLQGEMRLIFDYCWRYRLIHCSIQVQKSNGDILFYSYYPFGEHGCSDME
PQLINRYNGSMLVEPDLFPRKLRNFFGCPLRCALWDVPPFLTLDEDQEEVLRVNGGYEGR
LLLALAEKMNFTIAVRKVHVNMRDEALEMLRRDEVDLTLGGIRQTVARGMVATSSHNYH
QTREVFGVLASSYELSSFDILFYPYRLQIWMGILGVVALSALIQLIVGRMLRERMGSRFWL
NLELVFVGMPLLECPRSHTARLYCVMLMMYTLIIRTIYQGLLYHLIRTHQLNRWPQTIESLV
QKNFTVVLTPIVQEVLDEIPSVQHMRFRLLEANSELDPLYFLEANHQLRQHVTASALDIFIH
FNRLSADKVHQRGEQGSGAHFEIVPEDIISMQLTMYLAKHSFLIDQLNEEIMWMRSVGLL
SVWSRWELSESYLRNEQSFQVLGTMELYAIFLMVLVGLIVGLLVFILELVSMRSIYLRKLFT
>DmelIR7f
MNTTSDSNAGSSLSSGSGYSIYKSYLENSRIDMQGEDANLYVARALRLVIENVLAQLSTTL
VVTISTRHLGTAHWFEYMMNILMDSWRMVAVQLLRIRPDLVVNPVPGRKRVSLLMVDSY
QGLLDTNITASNANFDDPDYYFIFLQARDHLIPKELQLILDHCLAHFWLHCNVMIQTAQVE
VLVYTYYPYTADACQKAYPIPVNTFDGRKWKASQMFPDKLSQMHGCPLTVLTWHQPPFV
ELVWDPKHNRSRGSGFEIQLVEHLARRMNFSLELVNIALLRPNAYRLAEGSSEGPIEKLLQ
RNVNISMGYFRKTARRNQLLTTPMSYYSANLVAVLQLERYRIGSLALLVFPFELSVWMLLL
LALLIHLGIHLPSARRGNEEDGGGGLQVVALLLGAALARLPRSWRHRFIAAHWLWASIPL
RISYQSLLFHLIRLQLYNTPSFSLDQLLAEGFQGICTANTQRLLLEMPQLARDPDSIQSVDTP

FDWDVLNVLTRNRNRKIFAVANQDVTLSFLHSSAHPNAFHVVKQPVNVEYAGMYMPKHS
FLYEKMDDDIRRLDASGFIHAWRRASFASVHRKEQVHMTSRRYINHAKLSGIYMVMAGL
YLLAGLLFAGEVLLRQRN
>DmelIR7c
MLHSAVHNVSLVYALVWAIDNYYGMATSTPLAVVQFPTSRESRRLHNDLIDAALGRSSGT
GRIQFLLEDDRVEMTETDTDPPPPSGLTGRPIAIWFLDSLRSYFRLEMYLNQLGSPYKRNGF
FLVIYTGLEDQPMESLKIMFRRLLNMYVLNVNVFLQRDGTVHLYTYYPYGPHHCQSSLPV
YYTAFQDLAAPANGFGLTKPLFPRKLTNMHGCEMVVATFEHRPYVIIEDDPKTPGGRSIHGI
EGLIFRSLAERMNFTIKLVEQKDKNRGEILPDGNFTGILKMMVDGEVNLTFVCFMYSKAR
SDLMLPSTSYTSFPIVLVVPSGGSISPMGRLTRPFRYIIWSCILVSLIFGFVLICLLKITALPGLR
NLVLGRRNRLPFMGMWASLLGGLALYNPQRNFARYILVMWLLQTLILRAAYTGQLYLLL
QDVEMRSPIKSLSEVLAKDYEFRILPALRTIFKDSMPTTNFHAVLSLEESLYRLRDEDDPGIT
VALLQPTVNQFDFRSGPNKRHLTVLPDPLMTAPLTFYMRPHSYFKRRIDRLIMAMMSSGIV
ARYRKMYMDRIKRVSKRRNLEPKPLSIWRLSGIFVCCAGLYLVALIVFILEILTTNHRRLRR
AFNVINRYAA
>DmelIR8a
MELPLLVLLLALRFAGSEVLKITFWIEPVQRAEFDTDIAMVLKELDALRLDVKVDDTTLTL
TRSEDGLDMQRFCEILSTVGASAVIDLTYSHWEEGYNLVRSLGIGYVRLERIMRPFLDMFG
DFMRQKRANNVAMVFMNARDAVEAMQQMLVGYPFRTLIMDASQTDPGQHFLERIRSLR
PAPTYIALFARAAAMNGIFEKVQKADLFQRPLEWHFVFLDTRDRVFKYRRQAELCTRFTL
NPRAICRSMPMPDLYCGSGFTMQRAMLLNVLRSLINAAQVSPGYPLAIYQDCNATASSSE
VSDPLEKDDYNWLDMVHWSNFLAYAPPLPHIQDQFQSPVPGLTFAVNISAGYYSSEHEAK
TDLAAWSSVGEMRLLNETISPARRFFRIGTAESIPWSYLRREEGTGELIRDRSGLPIWEGYCI
DFIIRLSQKLNFEFEIVAPEVGHMGELNELGEWDGVVGDLVRGETDFAIAALKMYSEREEV
IDFLPPYYEQTGISIAIRKPVRRTSLFKFMTVLRLEVWLSIVAALVGTAIMIWFMDKYSPYSS
RNNRQAYPYACREFTLRESFWFALTSFTPQGGGEAPKAISGRMLVAAYWLFVVLMLATFT
ANLAAFLTVERMQTPVQSLEQLARQSRINYTVVKDSDTHQYFVNMKFAEDTLYRMWKE
LALNASKDFKKFRIWDYPIKEQYGHILLAINSSQPVADAKEGFANVDAHENADYAFIHDSA
EIKYEITRNCNLTEVGEVFAEQPYAVAVQQGSHLGDELSYAILELQKDRFFEELKAKYWNQ
SNLPNCPLSEDQEGITLESLGGVFIATLFGLVLAMMTLGMEVLYYKKKQNALEITQVRPVN
DSSGSGGNSSTAPPTATSTTKQAWHIPVLEAEEKPAKVSPPPSFETATFRGKKLPARITLGDG
KFKPRHGLYARRNLGASDSHSGYME
>DmelIR20a
MLASLNRSTGLSAELLDLYGLVVHFLLSGEHTTLVYFNPAGLDCSWGVLWQRNLTAHPQI
VWQRNYSYPDLYYQFNAKLLVLACLPMDSRAAIQLEILANSLSHLRTVVRLLIEVAGPDQ
VTLARQYLSFCLRRSMLHVELYFRDYHHSLILYSFRAFPSFELVMRWISVGQGVKLFLHKL
DDLRGHRLRVIPDLSPPNTFFYRDARGDNQVTGYLWDFLATFAGRLNAGLEVVRPSWRA
GSASDSSYMLEYSAKGLIDVGLTTTLITKWNLWAIHQYTYPLLVSSWCTMLPVEKPLATPD
LFGRIVCPTLAMTLLLIILVTWLVFRQLRCLTRLKNSRPARIVPHLLTLLLLTTCSAQLLSLLI
FPPYHVRIASFEDLLRGDQKILGMRNEFYNFDGAFRARYAGVFYLIDDPNELYDLRNHFNT
TWAYTMPYIKWLVIKTQQRHFSKPLFRWSKDLCFFDFMPTSVIVAPDSIYWESIKDFTFRIH
QAGLMKHWIRKSFYDMIKAGKMSIKDYSDLETLKPLNIGDLEIVWRVCGAAIAVASAIFIM
ELLYFYINVFFNSL
>DmelIR31a

MNLLISMFILILAAGEGEIIPSMEESVVTNFVKSLVKTKQAIVFSCLFKDFKEISLALMRINQ
FVSVVNLNQSYSLTSILTRENYARTSVMVNARCSGSSELLFEASENRYFNKTYQWFLWGV
DLEVQSLFPLNLNYVGPNAQITYVNETADGYAYWDIHSKGRHLKSNLEINLIATLINDTLNI
ARDIFHLQSIDFRGQFNGLTLRGASVIDKEDIISNEQIESILSRPTKDAGVAAFIKYHYELLGL
LRERFNFTVNFRNSRGWAGRLGNTTFRLGLLGIVMRNEADIAASGAFNRINRFAEFDTIHQ
SWKFETAFLYRYTSDLDTHGKSGNFLSPFSDRVWLFCLLTLGAFSIIWVLFEIIDYKILRIRV
NSQKLEHLNQKSSVICIKTTCIERILQTFGACCQQGLDPNPVDRSVRFLVMTLFLFSLVMY
NYYTSSVVGGLLSSSDQGPSTVDEITASPLKISFEDIGYYKVLFRESQNRSITRLIEKKLSSS
RSLNELPIFSHIEDAVPYLKAGGFAFHCEVVDAYPVISEYFDANEICDLREVSGLMEVEILN
WILHKNSQYTEIFKTAMCNAQEKGFVERILRRRQIKKPACQSLYTVYPVSLSGVLPGFVILI
CKSINKFS
>DmelIR41a
MFIDLSWSLVLSAIVGKYLNESTICIFWNDKFEFQLLHKSDYISFVGINIKSFDDNGGHYIID
TGLKKKELQNKHLFLDELVIKIIISIEVTHCETFVVFDKDIDRFVNAFNKASVYSIWRSLHN
KFVFAHIANESPESRNHFFEDQPNILFVVRDHSSASSFDIKTNKFVGRKAENPSQMILVDRY
LASEQRFQFGKSLFADKLNNLQGREVIIAGFDYPPYTVIKHNMSTNAQDMGVSGESDFKN
VYIDGTETRIVLNFCEQFNCTIQIDSSAANDWGKVYPNMSGDGALGMLINRKADICIGAM
YSWYEDYTYLDLSMYLVRSGITCLVPAPLRLTSWYLPLEPFKETLWAAILLCLCAEATGLV
LAYKSEQALYVLPGYREGWWTCTSFGVCTTFKLFISQSGNSKAYSLTVRVLLFACFLNDLII
TSIYGGGLASILTIPSMDEAADTVTRLRFHRLQWAANSEAWVSAIRASDEALVKDILYNFHI
YSDDELLRLAQDQHMRIGFTVERLPFGHFAIGNYLGPQAIDQLVIMKDDIYFQYTVAFVPR
LWPLLDKLNTLIYSWHSSGFDKYWEYRVVADNLNLKIQQQVQETMTGTKDIGPVPLGMS
NFAGFIIVWILGSAIATLTFLLELSLTYILKQSNLK
>DmelIR47a
MRQIKLLVWLLVVGVVSSTEQLQFLKNFLEAVHKERSISTILLIQRKVHKNDFLHGLYPIF
WPIICLDETKRVELVNNFNKDFLALVYMESEADTLLLSALAADLNHIRDARIMIWLQMSPS
ENFLDRIVFQASKQKFLNLVVIENTLKTRRFYPFPQPKVQVIDKPFEEKEIYPALWRNFMG
KNAIAVPDLVPPRSFNSFDPKTGHRRESGSIYNVFKAFTQRYNITMLLKWPLIRNTTQEEIIG
KSVRGEIDLPITGQLISFRHPNGSRSQPLLGMTALSIAVPCGPELPMFDRFFLFYGLATPITIT
GYYVLLNTIEIILGTLSDRIKRHPRRKKILNLVLNLRVFSCILSLPTPQGNRLRSVKGQLTMV
MSITGLILSCIVAAQTSTILTMKPQYRHIKNFQELSDSNITVVCNHLNYLTIKQQMDPKFMA
KFMQNIWIVNSIEQMKMIFDLNTSYAYQTFSYKKDPFTLLQMHTTRKAFCRTPGLDLVSG
LAYTAVLEKNSIYALALQDYTLKAFSAGLVYYWAEESIRDLISTVGRTQFEKLPIVIGYQSL
KLQDYNVCWKILLIGGALAFCVFIVEVVVGLINRRI
>DmelIR48c
MSLLRIILIIIFLRIVSSIPDTIISHLSAELQIKIQIYFGLGNDLYDFSRLDGNYQKIIISHNISEEF
KTYHDEPVLIIIRLERDLNLNLATLDVLRSYLTDRQYNDILLIDNDEENLNSYVDIRKAYW
NAGFSQVLIYNSQQRTWSIKPYPYLQIRPTSLKEYIENRNTRNLMGYPLRVLVTNDPPHCF
VDKDELPGSPNRYKGSIVTMLKIFADQLNATFQANPFREFRRYSTADCVQMVSDDEIDAC
GSIFIRTYTYATSQPVRLNRVVIMAPFGNPIEKFYYFFRPFDLYVWIGTGIIVVYIAVMGSLL
HRWHFKEWNVGQYLLLAVQTLLNRELSLPQSSSGSKFMLLLLLFAIGFILSNLYVALLSMM
LTTKLYQRPIENLADLKAANVNILLQTHNIRPNSVYGSSEELRERFLLVEESQHLEKRNGLD
PSYAYVDSEDRMDFYLYQQKFLRRRRMKKLSNPVGYTWAVQVIKQNWVLEKHYNDHVQ
RFFETGLQNKLVDDVHELAVKAGFLHFFPTQTQTIEPLRLEDIVMAAMVLGGGHALAVIC

FLVELFA
>DmelIR52a
MALGWSVIILGFIGQLSAQILNYTQSRDLELLEGSLFRVLSRLNLEEEYNTLLIYGKECVFH
SLLRKLEISAVTVPSGSTDYDWSFSTAILILSCGYDAENEENSYTLMKLQRTRRLIYLEDNS
EPESVCMRYSLKEQHNIAMVKSDFDQSDTFYSCRLFQTPNYVEGHFFKDQPIYIENFQNM
RGATIRTVADSLVPRTILYRDEKSGETKMMGYLGHMINTYAQKLNAKLHFIDTSKLGAKKP
SVLDIMNWVNEDIVDIGTALASSLQFKNMDSVWYPYLLTGYCLMVPVPAKMPYNLVYSM
IVDPLVLSIIFVMLCLFSVLIIYTQHLSWKNLTLANILLNDKSLRGLLGQSFPFPPNPSKHLK
LIIFVLCFASVMITTMYEAYLQSYFTQPPSEPYIRSFRDIGNSSLKMAISRLEVNVLTSLNNS
HFREISEDHLLIFDDLSEYLVLRDSFNTSFIFPVSVDRWNGYEEQQKLFAEPAFYLATNLCF
NQFMLFSPPLRRYLPHRHLFEDHMMRQHEFGLVTFWKSQSFIEMVRLGLASMEDLSRKR
NEEVSLLLDDISWILKLYLGAMFISSFCFILEILRCGERCKRLWRCRW
>DmelIR52d
MVRIIIILLCLGYTKARILDATNTNHTDLEERLLSLLLRLQQEQFFNTLLIYGEDCAFSSLSR
RLQVPTILVSSGSTSFEWNYSSLALILTCEFKAEREENYQTLKKLQMNRRLILLNGNIKPDS
VCDFYSKKDQYNIAMVNNNFHQVGIIYACRLFQERNYEKVYLSEGNPIYVDQFRNMQGA
LLKSITFNLIPGSMAYRDPKTGQEKHIGYVANLLNNFVEKVNATLDMQVKLHKAGKKTSF
YNITKWASEDLVDIGMSYAAYFEMTNFDTISYPYLMTSTCFMVPLPDMMPNSEIYMGIVD
PPVLVVLIAIFCIFSVMLNYIKQRSWRSLSLVNVLLNDICLRGFLAQPFPFPRQSNRKLKLIS
MLVCFFSVITTTMYTSYLQSFMWGPPIDPKMCSFADLENSRYKLAIRRYDIEMLRPFNVSM
DHVVVFDESSQLEYLRDSFDDNYMYPMSALSWSAFKEQQKLFAFPLFYYSEKLCLKPISF
FSFPIRRHLPYRDLFEEHMLQQNEFGLSTYWIDRSFSDMVRLKLATMNDFSPPRLEDYIEV
SDLSWVFGMYFTGLGISCCCFGLELLGLPSWTRRLRLTNWLRVRN
>DmelIR54a
MWTVITGIVLWAPVLVAGSAVDFIFRAAAEHSLSVIMIRIDYCPYNWAKDIFENQTIPVVVL
SDSETFINIRMFSRPLHVACLPGHELQKDLALLENFTSSLMDFPSQKKIVYISNNFSDPTRM
DYIFETCYHRRIWNIVGLLASDEHRYFYRYHLYPSFRTEYRSLESSTIFDKDFPNMHGHPLT
VMPDQWLPRSVLYVDRRTGKQILAGSVGRFFHVLSWKLNATLQLSKKVTTGRFLNATAL
KELSESFSVDVPASLTIMERVEQLASTSYPMEVTHVCLMVPVARRIPIKDIYFILSSASNMFL
AIVIVSSYGLALNLLRNMTHRDVRLVDFVLNDKALRGILGQSFNLPLSRSFSTRLIFLMLGI
VGLNVSSIFGAGLDTLMAHPPRQFQARSFAGLRRTKIPLVTTEEDFPTWMKLRVPMLVVN
VSEYNHLRNGRNTSNAYFASRLYWNLFSEQQKRFTRELFIYSTDDCLWSLALLSFQWPQN
SLFTEPVSQLILEVNANGLYDFWVGMHYYDMTAAGLSGLEDPSLQLKEREHPTSLRIVDF
QWMWQAYGTFMVIAILVFLLEVSWHRITSLFVSLVY
>DmelIR60d
MRLAIYVAFLSSIGNRSGFLSSLLMSLGKELHYKTILLVGGSSTCWSLEPFETGVPILNLRG
ENNAYPQDTFNSQMLALACLQTESEDAVKLLYRSLKDMRDTPTLLFASSEEHIHDTLFLGC
FRENMLNVLALTASSKEFIYSYQAFPTFRVIKRKLVEIHRYFEPQLKDLGGHIVSALPGNIM
PRTMCYRNAEGERQLAGYLNTFIRNYVESINGTLRISWGLVPEDDMRHLTISRLSKIQHVD
FPLGIIPLYNKTDKQHVYMEISSWFLMLPMETSVPRAHLFVKLGLERLLPIIVVVGAVLGN
AHRIEVGLGPSWRCYYLADKVLRGALAQPIVLPRRLSPKLMLIYSLLLLSGFFLSNYYMAS
LTTWLVHPPASDRILEWDQLRYLHLKVLTIPEEFKYMSLILGTDFMTAYGSIFQLTNSTDFQ
RRRISMDPSYAYPVTTSLWPFLELSQVRLRRPLFRRSYDMVLQPFQVMSLPLPRNSIFHKSL
LRYAALTRETGLYYYWFRRSYYELVALGKISYKEEEGNPYCDLKWNDFRIVWLAFLGGTII

SCLALLLEVAHYRWHLGNSSL
>DmelIR60e
MVIKMISFLLVSVLLCLVGASDSESMQVQVLQDLNLALQTELNVFIDFECCATSEILHKLD
SPRILLSSNSREARDLRIRGNFTESTLIIVSVMDSDLNPLVASLLPRLLDELHELHIVFLSNEE
PGFPKQDLYTYCFKEGFVNVILMSGKGLYSYLPYPSIQPISLSNVSEYFDRARIIRNFQGFPV
RILRSTLAPRDFEYSNEQGGLVRAGYLFTAVKELTYRYNATIESVPIPDLPEYDVYLAVAEM
LHTKKIDIVCYFKDFSLEVAYTAPLSIIREYFMAPHARPISSYLYYSKPFGWTLWAVVISTVL
YGTVMLHLAARGARVEIGKCLLYSLSHILYNCHQKIRVAGWRDVAIHGILTIGGFILTNVYL
ATLSSILTSGLYDEEYNTLEDLARAPYPSLHDEYYRSQMKAKTFLPERLRRNSLSLNATLL
KAYRDGLNQSYIYILYEDRLELILMQQYLLKTPRFNMIRQAVGFTLESYCVSNSLPYLAMT
SEFMRRLQEHGISIKMKADTFRELIHQGIYTLMRDDEPPAKAFDLDYYFFAFVLWTVGLIS
SLLVFFAELVSGHL
>DmelIR67a
MLPILVPVLLLFNETSWINPILTSIYKDRHHETVLLLQHSQHGNASGLERFPWPVFSFNEQM
DFYVRGKYNSEMLVLIWQTGNSDWDLDLWQALDRSLLNMRKVRVLLLRKWEKIPTADV
AATAEHLLFLHVAVIGQGNRIYRLQPYAPQSWLQVDPIESPIFIKIRNYFGRYIVTLPDQFPPR
SIVYRNPKTDEIQMTGYVYKFLLEFIRIYNFTFRWQRPIVQGERMNLILLRNMTLNGTINLA
ISLCGFETPSELGVFSDVYDMEEWYIMVPRAQEISIADVYVVMVSGNFLIVLIIFYFIFTILD
TCFGPLLLKERVDWSNLMLNERMISGIMGQSFNMSARNTISSKVTNATLFLLGLVLSTLYA
AHLKTLLTKRPTSQQISNFKQLRDSPVTVFFEEAERFYLKHAWDRPIRYIKDQLNFRETIEY
NALRMGLNRSNAFSALTSEWMIVAKRQELFKQPIFTVQPELRVIQTSVLLSLVMQSNSIYE
DHINDLIHRVQSAGIVEYWKHQTLREMITMGMISQKDPFPYVAFREFKVGDLFWIWLLWV
SFLFMSFVIFLCELLVDCFISKTLIRNKRPH
>DmelIR75a
MQLVQLANFVLDNLVQSRIGFIVLFHCWQSDESLKFAQQFMKPIHPILVYHQFVQMRGVL
NWSHLELSYMGHTQPTLAIYVDIKCDQTQDLLEEASREQIYNQHYHWLLVGNQSKLEFY
DLFGLFNISIDADVSYVKEQIQDNNDSVAYAVHDVYNNGKIIGGQLNVTGSHEMSCDPFVC
RRTRHLSSLQKRSKYGNREQLTDVVLRVATVVTQRPLTLSDDELIRFLSQENDTHIDSLARF
GFHLTLILRDLLHCKMKFIFSDSWSKSDVVGGSVGAVVDQTADLTATPSLATEGRLKYLSA
IIETGFFRSVCIFRTPHNAGLRGDVFLQPFSPLVWYLFGGVLSLIGVLLWITFYMECKRMQK
RWRLDYLPSLLSTFLISFGAACIQSSSLIPRSAGGRLIYFALFLISFIMYNYYTSVVVSSLLSS
PVKSKIKTMRQLAESSLTVGLEPLPFTKSYLNYSRLPEIHLFIKRKIESQTQNPELWLPAEQG
VLRVRDNPGYVYVFETSSGYAYVERYFTAQEICDLNEVLFRPEQLFYTHLHRNSTYKELFR
LRFLRILETGVYRKQRSYWVHMKLHCVAQNFVITVGMEYVAPLLLMLICADILVVVILLV
ELAWKRFFTRHLTFHP
>DmelIR75c
MTSWPLYRLIVFNLLEINLSNLMVFHCWSIKEAFPLVEMLNQNGIFSQYIDVQNPDNLANV
HKEYLDSDLVRLGVFLDLGCDKAELVTNQSSRARLYNQNLHWLLYDEAGNFTKLTQLFE
GANLSLNADVTYVSREDEERFILHDVYNKGSHLGGKLNITVDQTLQCNRSHCQVKEYLS
ELHLRPRLQHRMDLSSVTFRLAALVSVLPINSSEEELLEFLNSDRDSHMDSISRIGNRLIMH
TQEILGFKLHYIWCGTWSVQDAFGGAIGMLTNESAELCTTPFVPSWNRLHYLHPMTEQA
QFRAVCMFRTPHNAGIKAAVFLEPFMPSVWFAFAGLLIFAGVLLWMIFHLERHWMQRCLD
FIPSLLSSCLISFGAACIQGSYLMPKSAGGRLAFIAVMLTSFLMYNYYTSIVVSTLLGSPVRS
NIRTIQQLADSSLDVGFDTVPFTKTYLVSSPRPDIRSLYKQKVESKRDPNSVWLSPEEGVIR

VRDQPGFVYTSEASFMYHFVEKHYLPREISDLNEIILRPESAVYGMVHLNSTYRQLLTQLQ
VRMLETGITSKQSRFFSKTKLHTFSNSFVIQVGMEYAAPLFISLLVAYFLALLILILEICWARY
AKKKFSTIIPQNQ
>DmelIR75d
MKVQVAHWLPLIFFLLVSGTPRVAGSWRSEYSRQDPDPKTRWGNQLPDMLVAYYRHHGV
HSLMLVVCHTDIADFRLWKLWQHFNLNNFYVQVSTESSLRDLQHVDALDEHKDAPPPKS
FHANNSTHWETSFLLPALPYKMGILLLEFSSECALNLLRWSAASEHNYFTTNRFWLLLTED
PGDIDLLEDPEIFIPPDSELRVLHYENVGNFSCSLIDLYKVAAWKPLKRTLVGHNIRNSRHVI
HALQHFGSAITYRQDLEGIVFNSAIVIAFPDLFTNIEDLSLRHIDTISKVNHRLMLELANRLN
MSYNTYQTVNYGWRQPNGSFDGLMGRFQRYELDLAQLAIFMRLDRIALVDFVAETYRVR
AGIMFRQPPLSAVANIFAMPFENDVWVSILMLLIITTVVLVLELFFSPHNHDMSYMDTLNF
VWGAMCQQGFYVEVRNRSARIIVFTTFVAALFLFTSFSANIVALLQSPSDAIQSLSDLGQSP
LEIGVQDTQYNKIYFTESTDPVTKNLYHKKIASKGENIYMRPLLGMEKMRTGLFAYQVEL
QAGYQIVSDTFSEPEKCGLMELEPFQLPMLAIPTRKNFPYKELIRRQLRWQREVSLVNREE
RKWIPQKPKCEGGVGGFVSIGITECRYALGIFGCGAAVSFVLFLFEFIFRHFKQVYRIIKGYR
EVQR
>DmelIR76a
MENLLVESYYFSTVLSFFAQQFFADSHATCIFWHPAFDFRLETVHPMPLIIMDWHRWANRS
DQDVYDYKIKEDEFEGKGIPYNDWTLRLTVAIERSHCETFIAFQEQIPEFARYFYHASIYSI
WRSLRNRFMFVYTKEFEDKKDSYLSGYIFQDQPNILVITSQYLNSSTFEIKTNRFVGPRNFN
KNPEPVEFYILQRFDAKGTKATWETQSAMSSKMRNLKGREVVIGIFDYKPFMLLDYEKPP
LYYDRFMNTTDVTIDGTDIQLMLIFCELYNCTIQVDTSEPYDWGDIYLNASGYGLVGMILD
RRNDYGVGGMYLWYEAYEYMDMTHFLGRSGVTCLVPAPNRLISWTLLLRPFQFVLWMC
VMLCLLLESLALGITRRWEHSSVAAGNSWISSLRFGCISTLKLFVNQSTNYVTSSYALRTVL
VASYMIDIILTTVYSGGLAAILTLPTLEEAADSRQRLFDHKLIWTGTSQAWITTIDERSADPV
LLGLMEHYRVYDANLISAFSHTEQMGFVVERLQFGHLGNTELIENDALKRLKLMVDDIYF
AFTVAFVPRLWPHLNAYNDFILAWHSSGFDKFWEWKIAAEYMNAHRQNRIVASEKTNLDI
GPVKLGIDNFIGLILLWCFGMICSLLTFLGELWRGQG
>DmelIR94e
MDCPKWILSGLCLISLVSGATVIELLGTLKLELDFEYVLLMKNRNFSLSDQVWNGTSLTKD
VMDEVQVPVLQFNENVSYFLHNSISRRLVTLGFMSDANLDEHRGLLTALVANLRHMTTSR
VIFLVQSKASTDFLYELFRNCWRKKLLNVIVIFQDFETTSTFYSYSNFPILQIEERIYETSLQT
LPIFPDRLRNLHGYEMPVILGGTAPRMIAYRNKKGNVVYDGTVGHFMTAFQQKYNVKFV
QPLQAKNPLDFAPSMQTVGAVRNETVEISISLTFPTIPPFGFSYPYEQMNWCVMLPVEADV
PPFEYYTRVFELAAFLLTLGTLVLISCLLASALSLHGYATNISEFLLHDSCLRGVLGQSFVEV
FRAPTLVRGIYLEICVLGILITAWYNSYFSSYVTSAPKQPPFRTYDDILASKLKVVAWKPEY
AELVGRLLEFRKYETMFLVEPDFNRYLALRDTLDTRYGYMITTNRWVLINEQQKVFSRPL
FQKRDDFCFFNNIPFGFPLHENSVFMEPVQKLIMELAETGLYYHWITTGFSELIDAGEMHF
VDLSPHREFRAMQIQDLQYVWYGYAFMVVLSSLVWLLENLAYTVKSKTIFPTHFMQRNK
K
>DmelIR62a
MYLQFLFALFLSRYQIVATENFDRAFELALFLDRIGRVHRLHAITIVNSLGSVDPSYLDDLH
RGLMCNSSNHFYMLPQMTATDKDSSHVHFSSLQDEETIYLVFARDSKDAVIYLQAERARG
RRYTRTMFLLRKQESQKDIKYFFELLWKLQFRSALVVVAARNFYQMDPYPTVRVIRMRRL

SSYDPHHVFPPANRKNFRGYRMRLPVQQDVPNTFWYKNRRTKAWELAGLGGILINQLM
MHLNVTMDLFRFEVNGSSLLNMAALTDLIVKGKVELSPHLYDTLQSNTSVDYSYPTQVAP
RCFMIPLDNEISRSLYVFLPFSLTMWLCLLFVLLVVHFVYVRRLIPDGHFWAILGVPGAGQ
VRYGNRKPVRRFSTFLILFGIFILGQTYSTKLTSSLTVTLIRRPDNSLEELFLLPYRILVLPTD
VYAIVDSLGHAEQFSTKFSCTDAENFSQKRISMHPEYIYPISTIRWRFFDMQQRFLRKKRFY
FSKICHGSFPYQYQLRVDSHLKDALHRFLLHVQQAGLHDLWLDTCYRKAHRMGYLKDFS
TLAELEEKLRLRPLALNLLVPAFSLFLCGMLGSGIAFLVEIRHSFGCRQKPPSINRNPGD
>DmelIR76b
MATGIELLVAAALCVACPPLNDSPPTNLIQMGENGTLSPVTELPMDVDASEAGFDADAPV
ETLETINRKKPKLREMLDWIGGKHLRIATLEDFPLSYTEVLENGTRVGHGVSFQIIDFLKKK
FNFTYEVVVPQDNIIGSPSDFDRSLIEMVNSSTVDLAAAFIPSLSDQRSFVYYSTTTLDEGE
WIMVMQRPRESASGSGLLAPFEFWVWILILVSLLAVGPIIYALIILRNRLTGDGQQTPYSLG
HCAWFVYGALMKQGSTLSPIADSTRLLFATWWIFITILTSFYTANLTAFLTLSKFTLPYNTV
NDILTKNKHFVSMRGGGVEYAIRTTNESLSMLNRMIQNNYAVFSDETNDTYNLQNYVEKN
GYVFVRDRPAINIMLYRDYLYRKTVSFSDEKVHCPFAMAKEPFLKKKRTFAYPIGSNLSQL
FDPELLHLVESGIVKHLSKRNLPSAEICPQDLGGTERQLRNGDLMMTYYIMLAGFATALAV
FSTELMFRYVNSRQEANKWARHGIGRTPNGQSVAPSRWLRGWRRLNSGHGQLLGASTHG
QNVTPPPPYQSIFNGGSHGDPLNRWRRPLANGNALGNGVLLGGDSEGGVRRLINGRDYM
VFRNPNGQSQLVPVRSPSAALFQYSYTE
>DmelIR85a
MSIQWLKHILLLAILVNLAGTRENHIPLDLKKSSIVMVKMSQILCKARIKVLFVYFENQTS
HEHTGQILKEVTKCDISNQNTPLEAVKDDGILMYMVMITTNISQPLELSLIRKKSAAKHRS
HVFLLVRDADTVSDAWMRASFRQFWKIWLLNIVILYWRDGRLNAYRYNPFMDNYLIPVD
NKPNEVPTLEQLFPKTIPNMQRKPLRMCIYKDDVRAIFWRQGTILGTDGLLAAYVAERLN
ATMMITRPHSYNNHNLSSDICFLEVAKEYVDVAMNIRFLVPDTFRKQAESTVSHTRDDLC
VIVPKAKTAPTFWNIFRSFGSLVWALILVSVLVANVFCYILKSEVGRVPMQLFAGALTMPM
TQIPPNHSIRLFLIFWLYFGLLICSAFKGNLTSMMVFQPYLPDINQLGALARSHYHIIIRPRH
VKHIQHFLTLGHKHESRIREQMLEVSDTQMYEMMRNNDIRFAYLEKYHIARFQVNSRVH
MHLGRPLFHLMNSCLVPFHAVYIVPYGSPYLGFLDSLIRSSHEFGFERYWDRIMNSAFIKSG
VKVVNRRRGSGNDEPVVLKLQHFHAVFALWLVGIGMACIVLAWEHLTHNYNLAVTKRRD
>DmelIR56c
MQHLLNLLAPFGRMNVFQEIVWFVSPHQRLDQLDEFIMRIDEAFGKSATQTVVNNNTEM
RMIYSSARRNHMSFVFTTGAEDPIMKVFSKVLLGRHFYVSMVIYVDKVGDMHPIYDLLTF
AYNQQFFNSMVHFESMEGVNQLFGVSKFPVMSFENRTDFLKYMGKIWKQVQNARSDVG
GFGFTTPLRQDLPHLFQSQGHYDGSTYRIIETFVRFINGSFKELIMPPDSLGGQVINMKDAL
QLIRERKMEFCAHAYALFMSDEELEKSYPLLVVQWCLMVPLYNSVSTYFYPLQPFDWNV
WFFALGALLALVLLELMWLRMFGGWSGYRGAVLNSFCYIINVPIEGQLQQPCLLRFLLLA
TVFFHGFFLSAYYTSNLGSILTVNLFHAQINTMNDIVSAQLPVMIIDYEMEFLLNLNKELPQ
EFLELLRPVDSAVFSEHQTSFNSSFAYFVTEDHWEFLDEQQKHLKQRLFKLSSICFGSYHLA
FPLQMDSSLWRDIEYFTFRIHSSGLLNFYARSSFGSALHAGLVQRMPDTQEYTSAGLQHLA
IAFILLLVMSFLAGIVFVLETLSR
>DmelIR75b
MLQLHNLILHNLIHMAKLSHVLILHCSLSHLALLAQSKNIFTQFQPLHSDIQLNDDFLNHNI
LKLGVFLDINCDKSGTVLDMASAKRFFSHRYHWLIYDRSMNFSVLESHFKEAQIFVDADV

TYVTHDPFSKNFLLYDVYNKGRQLGGELNITADREIFCNKTNCRVERYLSELYTRSALQHR
KSFTGLTMRATAVVTALPLNVSIKEIFDFMNSKYRIQLDTYARLGYQARQPLRDMLDCKFK
YIFRDRWSDGNATGGMIGDLILDKADLAIAPFIYSFDRALFLQPITKFSVFREICMFRNPRSV
SAGLSATEFLQPFSGGVWLTFALLLLLAGCLLWVTFILERRKQWKPSLLTSCLLSFGAGCIQ
GAWLTPRSMGGRMAFFALMVTSYLMYNYYTSIVVSKLLGQPIKSNIRTLQQLADSNLDV
GIEPTVYTRIYVETSEEPDVRDLYRKKVLGSKRSPDKIWIPTEAGVLSVRDQEGFVYITGVA
TGYEFVRKHFLAHQICELNEIPLRDASHTHTVLAKRSPYAELIKLSELRMLETGVHFKHER
SWMETKLHCYQHNHTVAVGLEYAAPLFIILLGAIILCMGILGLEVIWHRHCTLH
>DmelIR84a
MIKLQVKVISWPLIILTAFLRVLQIESINTNFLELAAFEDFLRSEHLSHVLVVRGDDADGDW
KIECHQKLLANYRVQFYRPEMSANFEDLMFYGSPRTAVLVLNSEHVLVRRQVFGVASEAG
YFNNSLAWFILGSGRESLPVEQLIDQLLSGYRMGIDADITVALRGPDNASMLFYDVYRISR
QANTPLIIEKKGLWTHSGGYQKFGNFKNTWVIRRRNFLNVTLIGSTVLTEKPPGFGDMEY
LADDKQLQQLDPMQRKTYQLFQLVERMFNLSLAISLTDKWGELLDNGSWSGVMGQVTS
READFAVCPIRFVLDRQPYVQYSAVLHTQNIHFLFRHPRRSHIKNIFFEPLSNQVWWCVLA
LVTGSTILLLFHVRLERMLSNMENRFSFVWFTMLETYLQQGPANEIFRLFSTRLLISLSCIFS
FMLMQFYGAFIVGSLLSESARSIVNLQALYDSNLAIGMENISYNFPIFTNTSNQLVRDVYV
KKICKSGEHNIMSLQQGAERIIQGRFAFHTAIDRMYRLLLELQMDEAEFCDLQEVMFNLPY
DSGSVMPKGSPWREHLAHALLHFRATGLLQYNDKKWMVRRPDCSLFKTSQAEVDLEHF
APALFALALAMVASALVFLLELFLHWLPDFRRRLGTMST
>DmelIR92a
MLLQPLVMHLSQLLRIIVGQYFAEFPSILIVYNNSASTTPLQLEYLSALELVLRELSKPIRLQ
WINVAFLKDLNDLEDQVMGALNSSVTEGFITILSQTHHFIHARYYATRNANVRLKDKRYL
FLCEDESPAELLCMDILQFYPHHLMVRPGTETAPTGPTGPHPDPRRGGGASVSTKNKDDG
EGGAGNKTTSPYRDINFELWTQKFVGAVGNLDALLLDAFLPNETFANRVELYPNKLLNLQ
RRSLLVGSITYVPYTITNYVPAGQGDVDPIHPQWPNRSLTFDGAEANVMKTFCQVHNCHL
RVEAYGADNWGGIYDNESSDGMLGDIYEQRVEMAIGCIYNWYDGITETSHTIARSSVTILG
PAPAPLPSWRTNIMPFNNRAWLVLISTLVICGTFLYFMKYVSYRLRYSGTQVKFHHSRKLE
KSMLDIFALFIQQPSAPLSFDRFAPRFFLATILCATITLENIYSGQLKSMLTFPFYSAPVDTIEK
WAQSGWKWSAPSIIWVHTVQSSDLETEQILARNFEVHDYSYLSNVSFMPNYGFGIERLSS
GSLSVGDYVSTEALENRIVLHDDLYFDYTRAVSIRGWILMPELNKHIRTCQETGLYFHWEL
EFIDKYMDKKKQEVLMDLANGHKVKGAPQALDVRNIAGALFVLAFGVAFAGCALVAELL
IHRMDLSK
>DmelIR94b
MSLIFNLLFILILSQAVSQETEFLQLKYLNNIVRSMIKLHKMETLVIVKHHLDNNCSLQNW
NAHGMGIIRTNDQGKLIMKDTFNSRTLAIICIGQNSHITLLRNVFETFGKVQQKKIILWTQM
ELKEKFFQEISKKSRDLKLLNLLVLKAVTKDKLLIYRLNPFPSPHFKRIENIWTPNDTLFMD
TKFNFHGMTAVVKHDYNWTIQMGNIRKFPISRIEDKEVIEFALKYNLTLQFFNDVERFDIEL
RKRIILKSNSTQPIDSGIPMVFSSLLIVVPCGNYLSIQDVIKVSGIEKWIFYIILVYVIFVLIEIT
FLGVTILISRQSRHQMIPNTLVNLCAFRAILGLPFPETRRTSLSLRQLFLAIALFGMIFSIFINC
KLSSMLTNPCPRPQVNNFEELKTSGLTVVMDHDAENFIEKEIGVDFFNQYMPRKVTLTFTE
RAKLLFSLKGNHAFTLFSESFAIIESYQRSKGLRAHCTSEDLIVAERVPRIYILENNSILDRPL
RRFIRQMQESGITNHWLKNIPSSLEKNLMQITIPYDRERVHPLSIEHLTWLWCILILGYSISM
IVFFVEMSLKRRKKNLENRAPNICIC

>DmelIR94c
MSKVFKLLVLPLIYLSLTKGSKNPQLKFLRELINVIEEGREIRTIMVIKHSRDEYCHLDQWN
PRGSPILRTNEMGSIRISGYFNDQAVILACMGENSDYGLLKSLANAMDNMRQERIILWSER
EPTKMLMDYISQQADRYNFAQIIIVTMNEDVDAVPSLHQLNPYPTPRFRQITNISNIRRTSFF
GCGLSFQGKTAILKESVVSNIRFKVWSPSGPIPLSELKDYEIVQFAVKYNLSLKLYDQNESK
SDHFDIQLGPLFITKDFPTQMAFVSPNTACSLIVIVPCSPKWRFMDVLHKLGVLKLIGCLLI
AYAVFVLIETLILWLTHRISGREVRLTSLNQLLNPRAFRGILGLPFPEFRRSSISLRQLFLVISV
FGLVYSNFVSCTLSALLTKPAQNPQVRNFKELRDSGLITIMDKYTHSFIEKHIDPEFFDHVL
PHYLILQKKEALRMIWNFNDSYSYVMYTTTWKSLNTVQKSFDERVFCESESLTIAWNLPR
MYVLGNNSVLKWMLSRYITYMPQTGIPDSWTEQLPKVLKLLYNVTSPRRIKEGAVPLSIQ
HLSWIWHLLFIGESIATLVFIVEILLQKSNQHTSNMRERSSEDDDFV
>DmelIR94h
MLSNISFSSAPELVDLYGLVLKFLVSSETTLFYFNPTGQKCSWETLPRTILSNHPQIIWFREE
TYPGLYKRHSSNLFVMACLSSTSYDGQLQLLAESLTRYRSVRVLIEVQDKEGSFLASQILLL
CQQHSMLNVVLYFSRWTRTLNVFSYLAFPYFKLLKQRLSGSLRPKIFINQLKDLQGYKIRV
QPDLSPPNSFSYRDRHGECQVGGFLWRIVENFSKSLKGDTQVLYPTWAKAKVSAAEYMIQ
FTRNGSSDIGVTTTMITFKHEERYRDYSYPMYDISWCTMLPVEKPLSVEILFSHVLSPGSAL
LLILAFILFFLIVPQLIKCLGITFRGRLIGMASRIFALVMLCSSSAQLLSLLMSPPLHTRIKSFD
DLLTSGLKIFGIRSELYFLDGGFRAKYASAFHLTENPNELYDNRNYFNTSWAYTITSVKWN
VIEAQQRHFAHPVFRYSTDLCFSSETPWGLLIAPESFYREPLQHFTLKINQAGLITQWMTQS
FHEMVRAGRMTIKDYSRTNLMKPLRIQDLRKCWVIFAVGLGTSTVVFTIELLLIYTNVFLN
SL
>DmelIR94g
MSTAVNSVHSKLVSLISRGQELTSIFFYAPAKEKCHLEDTISSATWGLPLVIWRTDRTVILNG
FIGEGLLVLACLPGFHWRALLGSLARSLKYLRQARILIELMQDRDEFLVSEVLQFCLSQDM
INVNAIFDDFPETENLSSFEAYPSFEVVNQTFTPDTQVSDLYPNKMLNLRGGVIRTMPDYSE
PNTILYQDKEGNKEILGYLWDLLEAYAHKHNAQLQVVNKYADDRPLNFIELLDAAQSGIID
VGASIQPMSMGSLSRMHEMSYPVNQASWCTMLPVERQLHVSELLTRVIPYPTLALLLLLW
IFYEVLRGRWRRHSRLQSIGWLVLATLVSSNYVGKLLNLFTDPPSLPPVNSLAALMESPVRI
ISIRSEYSAIEFTQRTKYSAAFHLALHASILIGLRNAFNTSYGYTITSEKWKIYEEQQKRSSK
PVFRYSKDLCFYEMIPFGLVIPENSPHRAPLHSYTLLLRQAGLHDFWVNRGFSYMVKAGKI
NFTAVGERYEAKTLTITDLRNVFIIYVSVLLISLILFTCELFVSWVNYWLGF
>DmelIR94fMWQQVLLAETSNWFRSDVLQRFWTHLRVEIRFRTMLNYRLESCDCWFDNV
LGSDNSTALLWNDQTYPHYLRRRQDTDILVVSCLRFHQYQEVLLALSLMLDQMRSMPVV
LQLCGDEDSMQELNSARLLLKHSQDLKMPNVVLLSSTFFTSATLYSYEMFPEFNVQKLVY
QAYLTLFPYKLGNLKGHPIRTVPDNSEPLTIVRKTLNGSIAIDGLVWQFMIEFAKHINATLQL
PIEPHPEKSIKLVQILDLVRNQTVDIAASLRPYSLNVQRSSTHIYGSPMMVGNWCMMLPTE
RVIGSHEALTRLMKSPWTWLILLLFYSVHRFLAQKTRLRSSLIHLIKLLINLSLICFLQAQLS
AYFIGPQKVNHISNMQQVEESGLKIRGMRGEFMEYPIDMRSRYASSFLLHDLFFDLAQYR
NSLNTSYGYTVTSVKWELYKEAQRHFRRPLFRYSEEICVQKLSLFSLIQQSNCIYCYRSRIFI
LRMHEAGLIRLWYRRSYYVMVTAGRFPIGDLSTVHRAQPIRWTEWQNVVLLHGVGLLFS
VVVFVIELTVHYANVCLNNL
>DmelIR100a
MATTLQLIMLALVGGTLGQANNTDHKQVLTSIVKQLEGGLELHLRTSEDGGNDLVQFLM

QEKSSIIISAKQEEVPSRAKIMRHHFFIFDGVHQMQEIRTSLFNTDGFYILALENNTIEDDVL
LMEFAADVWLQHGHSRIYYVQLSKKSVLLFNPFLQRLVVVQDSKTYSRIYKDLEGYHLRI
YIFDSVYSSVIGDGENKVLSVTGADAKLAKTVARQLNFTADFVWPDDEFFGGRLANGEYS
GGVGRAHRGEVDIIFAGFFIKDYLTTHIQFSAAVYMDELCLYVKKAQRIPQSILPLFAVHMD
VWLCFLLVGLLGALVWLILRAVNLILGIEGVPDGSRATRISYFGAARRIFVDTWVIWVRVN
VGRFPPFHSERIFVASLCLVSVIFGALLESSLATVYIRPLYYRDVNTLRELDESGQPIYIKHPA
FKDDLFYGHNSEVYRRLDAKMMLVAEGEERLIEMVSKRGGFAGVTRSASLQLSDIRYVM
TKKVHKIPECPKNYHIAYVLPRPSPYLEEVNRIVLRLVAGGIVGLWTGEAKERAKWSIQRF
PEYLAELDVGRWKVLTLSDVQLAFYALTIGCLLSAIVCMAEILLGRQRRLHSPK
>DmelIR40a
MHKFLALGLLPYLLGLLNSTRLTFIGNDESDTAIALTQIVRGLQQSSLAILALPSLALSDGV
CQKERNVYLDDFLQRLHRSNYKSVVFSQTELFFQHIEENLQGANECISLILDEPNQLLNSL
HDRHLGHRLSLFIFYWGARWPPSSRVIRFREPLRVVVVTRPRKKAFRIYYNQARPCSDSQL
QLVNWYDGDNLGLQRIPLLPTALSVYANFKGRTFRVPVFHSPPWFWVTYCNNSFEEDEEF
NSLDSIEKRKVRVTGGRDHRLLMLLSKHMNFRFKYIEAPGRTQGSMRSEDGKDSNDSFTG
GIGLLQSGQADFFLGDVGLSWERRKAIEFSFFTLADSGAFATHAPRRLNEALAIMRPFKQD
IWPHLILTIIFSGPIFYGIIALPYIWRRRWANSDVEHLGELYIHMTYLKEITPRLLKLKPRTVL
SAHQMPHQLFQKCIWFTLRLFLKQSCNELHNGYRAKFLTIVYWIAATYVLADVYSAQLTS
QFARPAREPPINTLQRLQAAMIHDGYRLYVEKESSSLEMLENGTELFRQLYALMRQQVIND
PQGFFIDSVEAGIKLIAEGGEDKAVLGGRETLFFNVQQYGSNNFQLSQKLYTRYSAVAVQIG
CPFLGSLNNVLMQLFESGILDKMTAAEYAKQYQEVEATRIYKGSVQAKNSEAYSRTESYD
STVISPLNLRMLQGAFIALGVGSLAAGVILLLEIVFIKLDQARLWMLCSRLQWIRYDRKVT

>ApolPBP1
SPEIMKNLSNNFGKAMDQCKDELSLPDSVVADLYNFWKDDYVMTDRLAGCAINCLATKL
DVVDPDGNLHHGNAKDFAMKHGADETMAQQLVDIIHGCEKSAPPNDDKCMKTIDVAMC
FKKEIHKLNWVPNMDLVIGEVLAEV*
>ApolPBP2
SPEIMKNLCMNYGKTMDQCKQELGLPDSVINDLYNFWKDDYVMTDRLAGCAINCLSTK
LDIVDPDGNLHHGNAKEFAMKHGADDGMAQQLVDIIHRCEKSTPPNDDKCTKTMDIAM
CFKKEIHKLNWVPNMDLVVCEVLAVV*
>ApolPBP3
SQEIMKTMTLTFTKGLDACKKEMDLPDTVDVDFNNFWKEDYVVTNRDAGCAIVCLASKI
NLVDSMGILIHGSAHEFAKQHGADDNMAKQLSDTLHTCETIIGTGNDECTRALHVANCFK
VEMHKLDWAPSMDLIIGELLAEI*
>AperGOBP1
DVNVMKDVTLGFGQALEKCREESQLTEEKMEEFFHFWSEDFKFEHRELGCAILCMSRHF
NLLTDSSRMHHENTDKFIKSFPNNEVLSKHMVNLIHSCEQQHDADLDHCWRILRVAECFK
RSCQEAGVAPSMELLMAEFIMESEIN*
>AperGOBP2
TAEVMSHVTAHFGKALEECRDESGLSPEILNEFKHFWSEDFDVVHRELGCAIICMSNKFSL
LKDDTRIHHVNMHDYVKSFPNGEVLSAKMVNLIHNCEKQYDDITDECDRVVKVAACFKV
DAKKEGIAPEVAMIEAVIEKY*
>AperPBP1

SPEIIKNLSQNFCKAMDQCKQELNIPDSVIADLYNFWKDDYVMTDRLAGCAINCMATKLD
VVDPDGNLHHGNAKEFAMKHGADASMAQQLVDIIHGCEKSAPPNDDKCMKTIDVAMCF
KKEIHKLNWVPDMDVVLGEVLAEV*
>AperPBP2
SPEVMKNLCMNYGKAMDQCKQELNLPDSVIADLYNFWKDDYVMTDRLAGCAINCLSTK
LDIVDPDGNLHHGNAKEFAMKHGADDGMAHELVDIIHGCEKSSPPNDDKCIKTMDIAMC
FKKEIHKLNWVPNMDLVVGEVLAEV*
>AperPBP3
MIAKTFNLLVIVYLSTNTAVDSSQDVMKSMTLTFTKGLDACKKEMDLPDTIDVDFNNFWK
EDYVVTNRNAGCAIMCLASKVDLVDSMGILIHGSSHEFAKQHGADDNMAKQLSDTLHSC
EKTIGTLNDECLRALNVANCFKVEIHKLDWAPSMDLIIGEILAEI*
>BmorGOBP1
DVYVMKDVTLGFGQALEQCREESQLTEEKMEEFFHFWNDDFKFEHRELGCAIQCMSRHF
NLLTDSSRMHHENTDKFIKSFPNGEILSQKMIDMIHTCEKKFDSEPDHCWRILRVAECFKD
ACNKSGLAPSMELILAEFIMESEADK*
>BmorGOBP2
TAEVMSHVTAHFGKTLEECREESGLSVDILDEFKHFWSDDFDVVHRELGCAIICMSNKFSL
MDDDVRMHHVNMDEYIKGFPNGQVLAEKMVKLIHNCEKQFDTETDDCTRVVKVAACF
KKDSRKEGIAPEVAMIEAVIEKY*
>BmorPBP1
MSIQGQIALALMVYMAVGSVDASQEVMKNLSLNFGKALDECKKEMTLTDAINEDFYNF
WKEGYEIKNRETGCAIMCLSTKLNMLDPEGNLHHGNAMEFAKKHGADETMAQQLIDIV
HGCEKSTPANDDKCIWTLGVATCFKAEIHKLNWAPSMDVAVGEILAEV*
>BmorPBP2
SRDVMTNLSIQFAKPLEACKKEMGLTETVLKDFYNFWIEDYEFTDRNTGCAILCMSKKLE
LMDGDYNLHHGKAHEFARKHGADETMAKQLVDLIHGCSQSVATMPDECERTLKVAKCFI
AEIHKLKWAPDVELLMAEVLNEVSWKS*
>BmorPBP4
ESGVDVVKNLSLSFARFFLECDEERHFQPEVRLKVMTFWYSESSTWDRDVGCAFLCIFKK
MEIDNPQDPSYRTHLELLSFANSEDNKIANQMVEIFYACGENTETDPCLWALEQVKCYKN
RINQLGLTPTF*
>BmorPBP3
SSEAMRHIATGFIRVLDECKQELGLTDHILTDMYHFWKLDYSMMTRETGCAIICMSKKLD
LIDGDGKLHHGNAQAYALKHGAATEVAAKLVEVIHGCEKLHESIDDQCSRVLEVAKCFRT
GVHELHWAPKLDVIVGEVMTEI*
>MsexGOBP1
DVQVMKDVTLGFGQALEQCREESQLTEEKMEEFFHFWREDFKFEHRELGCALQCMSRHF
NLLTDSSRMHHENTDKFIKSFPNGAVLSKTMVELIHNCELQHDAEEDHCWRILRVAECFKI
SCTKAGIAPSMEVMMAEFIMETENK*
>MsexGOBP2
TAEVMSHVTAHFGKALEECREESGLPVEVMDEFKHFWREDFEVVHRELGCAIICMSNKFE
LLQDDTRIHHVNMHDYIKSFPNGQVLSEKMVQLIHNCEKQYDDIADDCDRVVKVAACFK
KDAKKEGIAPEVAMIEAVIEKY*
>MsexPBP1

SPDVMKNLCLNFGKALDECKAEMNLSDSIKDDFANFWVEGYEVSNRDTGCAILCLSKKL
DMIDPDGKLHHGNAMEFAKKHGADEAMAKQLLDIVHNCENSTPPNDDACLKTLDIAKC
FKKEIHKLNWAPNMDLVVGEVLAEV*
>MsexPBP4
NFKGKQIMRSVAETFGRTVFECQNEVLMKFGSGILNDIFRYWHEGQPLEDRDLGCIFRCIL
LKLELVNDNGRLIDANADGFFQANGADESMTKHLIELYHSCYQTMRFPQDDCMLILEIGK
CCREGVRNAHWTPGSK*
>MsexPBP3
IAPSSDAMRHIANGFLKVLDQCKHELGLTDQIVVDLYQFWKLQYALLNRDTGCAIICMSK
KLDLLDGTGRMHHGNTQEFAVSHGATDEVASKVVVIIRDCEKQQEGEQDDCVRVLEVAK
CFRTAIHELNWAPNMEVVVDELLTEI*
>MsexPBP2
SQEVLKQMSVGFSKVLQTCKTELSVGDHIIQDFYNYWREDYDLLNRDFGCMVICMAVKH
DLINDQLTMHHGNAHAFAKTHGADDDTAQQLVTILRECEAKHQSVEDVCNRALEMAKC
FRTKIHELKWAPAMEVVLEEIMTSV*
>PxylGOBP1
TVEVMKDVTLGFGEALEQCREQSQLTEEMMEEFYHFWREDFKFEARAVGCAIHCMSRYF
NLLGEQQRMHHDNTHKFIQSFPNGEVLSHQMVGIIHTCEQQHDAETDHCWRILRVAECFK
RESQAQGLAPSMEMLMAEFIMEADV*
>PxylGOBP2
TAEVMSHVTAHFGKTLEECREESGLSGEIMEEFHHFWREDFEVVHRELGCAIICMSNKFQ
LMQDDARMHHENMHDYIKSFPKGDLLSETMVRLIHNCEKKYDDIDDECSRVVKTAACFK
KDAQAEGIAPELTMIEAVLEKY*
>PxylPBP2
SADVMKGLSENFGKALGDCKKELDLPDSIMTEFYNFWKDDYVLSDRSTGCAIICLSSKLD
LLDPDGNLHHGNAKDFALKHGADEGMAGQLVGMIHECEKAAPDNPDACLKVLDIANCF
KKKIHELKWAPSMDVVVAEVLADV*
>PxylPBP1
SKETMKDITSGFFKVLNECKHELNLPDHLVGDFYHYWRQEYALLDRDLGCAILCMSRKL
ELIDASGKLHHGNTQEFAEKHGADNSMASKLVEVLHSCEKQHEAVSDECQRALEVAKCF
RSSVHELGWAPTIDVIIEEVLTDM*
>AselPBP
RECSQEVMHNLSKGFAEVLEDCKKQENVGDHIMQDFYNFWHEEYSLVNREMGCIILCMA
GKLDLLDGDTMHHGNAHEFAKKHGADDALAKQLVGLVHECEQASASVEERCARALETT
KCFRGKIHGLKWAPSMRVVMEEVMADM*
>AselPBP2
SQDVMKTLTINFGKPMEICKKELDLPEAVTKEFLNFWRDGYEVTNRLTGCAIMCISEKLEL
LDEGYKLHHGNAKDFAMKHGADAGMAQQLVDIIHGCNESTPDNTDHCLKTVAVAMCFK
HKIHELDWAPNADLIIAEVLAEV*
>HvirGOBP1
DVNVMKDVTLGFGQALDKCREESQLTEEKMEEFFHFWRDDFKFEHRELGCAIQCMSRHF
NLLTDSSRMHHDNTEKFIQSFPNGEVLARQMVELIHSCEKQFDHEEDHCWRISHLADCFK
SSCVQRGIAPSMELMMTEFIMEAEAR*
>HvirGOBP2

TAEVMSHVTAHFGKALEECREESGLSAEVLEEFQHFWREDFEVVHRELGCAIICMSNKFS
LLQDDSRMHHVNMHDYVKSFPNGHVLSEKLVELIHNCEKKYDTMTDDCDRVVKVAACF
KVDAKAAGIAPEVTMIEAVMEKY*
>HvirPBP1
SQDVMKNLSMNFAKPLEDCKKEMDLPDSVTTDFYNFWKEGYEFTNRHTGCAILCLSSKL
ELLDQEMKLHHGKAQEFAKKHGADDAMAKQLVDMIHGCSQSTPDATDDPCMKALNVA
KCFKAKIHELNWAPSMELVVGEVLAEV*
>HvirPBP2
SKELLTKMTGGFTKVVDHCKTELNVGDHIMQDMYNFWREEYQLVNRDLGCMIMCMTA
KLDLVGDDQKMHHGKAEEFAKSHGADDALAKQLVGLIHGCETQHQAIEDHCSRTLEVAK
CFRTKIHELKWAPSMEVIMEEIMTAA*
>SlitGOBP1
DVNVMKDVTLGFGQALDKCRQESQLTEEKMEEFFHFWRDDFKFEHRELGCAIQCMSRHF
NLLTDSSRMHHENTEQFIQSFPNGEVLARQMVELIHACEKQHDHEDDHCWRILHVAECFK
QACVQRGIAPSMEMMITEFIMEAEAR*
>SlitGOBP2
MATVTSSVMGTAEVMSHVTAHFGKALEECREESGLSAEVLEEFQHFWRDDFEVVHRELG
CAIICMSNKFSLLQDDSRMHHVNMHDYVKSFPNGHVLSEKLVGLIHNCEKQFDSMTDDC
ERVVKVAACFKVDAKAAGIAPEVAMIEAVMEKY*
>SlitPBP1
SQDLMAKMTKGFTRVVDDCKTELNVGDHIMQDMYNYWREDYQLINRDMGCMLLCMA
KKLDLMDDQTMHHGKTEDFAKSHGADDDVAKKLVSVIHECEQQHTGIADDCMRVLEVA
KCFRTKIHELKWAPSMEVIMEEVMTAV*
>SlitPBP2
SQDVMKNLAINFAKPLDDCKKEMDLPDSVTTDFYNFWKEGYELTNRQTGCAILCLSSKLE
ILDQELNLHHGRAQEFAMKHGADEAMAKQIVDMIHTCAQSTPDVAADPCMKTLNVAKC
FKLKVHELNWAPSVELIVGEVLAEV*
>SlitPBP3
SKDPMKYIASGFVKVLEECKHELNMNDHLIADLFHYWKLEYTLLNRDTGCAIICMGKKL
DLLDANGRMHHGNAQEFAKKHGAGDEVASQIVQIIHECEKKHERDDDECLRVLEVAKCF
RTGIHELNWQPNVEVIVSEVLTEI*
>DpleGOBP1
NVDVMKDVTLGFGEALKLCREESQLTEEKMEEFFHFWRDDFKFDDRAVGCAIKCMSSHF
DLLTDSHRMHHRNMDNFIKSFPNGEVLSQQMVTLIHECEQQHDSEEDHCWRILRVAECFK
SSCKKHGIAPTMELLMAEFVMESEAN*
>DpleGOBP2
TAEVMSHVTAHYGKSLEECRKETGLSKEILEEFKHFWSDDFEIVHRPFGCTLICMSNKFAL
LQDDARMHHINMNDYVKGFPEGDVLADKLVQMIHKCEKEYDDIKDDCDRVVKVAACFR
ADAKKEGIAPEVAMIEAVMEQY*
>DplePBP1
NQDVMKSLSYKFGTKLFECGERTNYTRAMARDILHIWEESYDLNHDETGCLVLCAMVRL
ELLDQQGNMIVENTEGFIRANGGDDSMVSFLIQLYSMCREKTSSISNGCKAAIELSKCFRA
AIQQIGWVPDTTSLLVISYD*
>DplePBP2

NEVMKGITSSFFKVLDECKRELGLTDNVLTDLYYFWKQDHPLMHRDTGCAIVCMSQKLN
LLDTIGKLHHGNAQEFAINHGAGEQMAKKLVTMVHECEQQFMEQEDSCLRALDVAKCFR
TAMHDVNWAPKFDIIVTEVLTEVK*
>DplePBP3
SQEIMKKLTTGFVKAMEECKAELNLGDHIIQDFMNYWREEYELLNRDTGCAIMCMASKH
DLITEDMKIHHENAHEFAKSHGADDDLAKQLVQMIHDCEKQFTDITDDCSKTLEISKCFRT
KIHELKWAPSMETILEELMTET*
>HmelGOBP1
TMEVMKDVTLGFGEALQSCREESQLTEDKMEEFFHFWRDDFKFEDREVGCAMKCMSSH
FNLLTDSHRMHHENTDKFIKSFPNGEVLSKRMISIIHTCEQQFDALEDHCWRILRIAECFKV
ACKKEGIAPTMELLMAEFIMEADPS*
>HmelGOBP2
TAEVMSHVTAHFGKSLEECREESGLSSDILDEFQHFWSEEFQVVHRELGCAIICMSNKFLL
MHDDARMHHVNMHDYIKSFPQGELLSEKMVNLIHNCEKQFDDIEDECSRVVKVAACFKE
SAKEEGIAPEVAMIEAVLEKY*
>HmelPBP1
NETLRGITASFLKVLEECRQELNIGDNVLADMYYFWKLDRTLIHRDTGCAIVCMSKKLNL
LDTSGKLHHGNAEEFALQHGAADDMAKKLVTTVHECEQKHELEEDQCLRALEIAKCFRG
AMHEINWAPKVDVAISEILTEV*
>HmelPBP2
SQEIMHKLTKGFATAFEQCKQELNLGDNIMQDFLNYWREEYELLNRDTGCAIMCMAQKH
DLLTEDGIHHEKVHGFTKSHGADDELAKQLVTMIHECEKSNAGVSDECMKTLEVAKCFRT
KIHELKWAPDMETILEEIMTDI*
>EoblGOBP1
DVQVMKDVTLGFGQALEVCREESQLSQDVMEEFFHFWREDFKFESRAVGCALQCMSRH
FNLLTDSSRMHHENTHRFIESFPNGSVLAKQMVSLIHGCEQQHEAEPDHCWRILRVAECFK
RRCQEAGIAPSMEIIMAEFIMETEAK*
>EoblGOBP2
TAEVMSHVTAHFGKALSECREESGLTPEVLEEFQHFWREDFEVVHRELGCAIICMSNKFSL
LQEDSRIHHVNMHDYVKGFPNGQVLSAKMVELIHNCEQQYDDITDDCARVVKVAACFK
RDAKKEGIAPEVTMIEAVMEKY*
